# Supplementary material for: Site-selective remote C(sp3)–H heteroarylation of amides via organic photoredox catalysis
Source: Nat Commun. 2019 Oct 18;10:4743. doi: 10.1038/s41467-019-12722-4 (PMC6800443; doi:10.1038/s41467-019-12722-4)
Supplement: Supplementary file 2 — Supplementary Information [file 41467_2019_12722_MOESM2_ESM.pdf]

# Supplementary Information

## Site-Selective Remote C(sp$^3$)–H Heteroarylation of Amides *via* Organic Photoredox Catalysis

*Chen et al.*

**Supplementary Methods**

**General Information.** The reagents (chemicals) were purchased from commercial sources, and used without further purification unless otherwise specified. Thin layer chromatography (TLC) was performed on EMD precoated plates (silica gel 60 F254, Art 5715) and visualized by fluorescence quenching under UV light and by staining with phosphomolybdic acid. Column chromatography was performed on EMD Silica Gel 60 (300–400 Mesh) using a forced flow of 0.5–1.0 bar. $^{1}$H, $^{13}$C and $^{19}$F NMR spectra were recorded on a Bruker AVANCE III–400 spectrometer (100 Hz and 376 Hz for $^{13}$C and $^{19}$F, respectively) spectrometer at ambient temperature. $^{1}$H and $^{13}$C NMR spectra are internally referenced to residual solvent signals ($CDCl_3$, δ 7.26 and 77.16 ppm; DMSO-$d_6$, δ 2.50 and 39.52 ppm). Data for $^{1}$H and $^{13}$C NMR are reported as follows: chemical shift (δ ppm), multiplicity (s = singlet, d = doublet, t = triplet, q = quartet, m = multiplet), coupling constant (Hz), integration. Data for $^{19}$F NMR are reported as follows: chemical shift (δ ppm). $^{13}$C and $^{19}$F NMR spectra are fully decoupled by broad band proton decoupling. High Resolution Mass spectra were obtained from micrOTOF-Q 134 High-resolution MS. The substrates **1a-1s**,[1] **2a**,[2] **2l**,[3] **2m**,[4] **2q**,[5] **2r**,[5] redox active ester **55**[6] and hydroxylamine derivative **58**[7] were synthesized according to the literature's procedure. Other substrates were purchased from commercial sources and used without further purification unless otherwise noted.

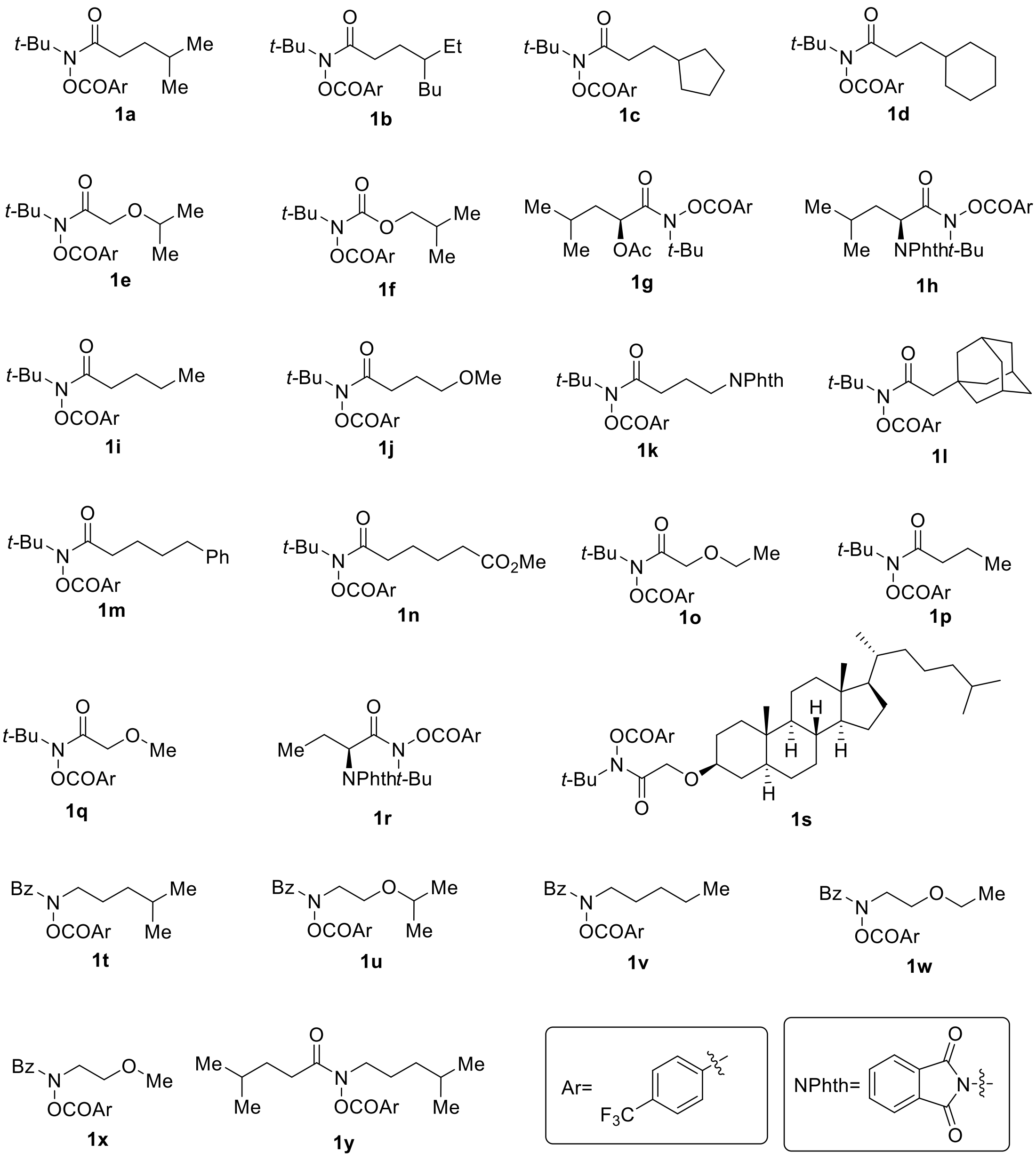


**Supplementary Figure 1.** Structures of hydroxylamine derivatives

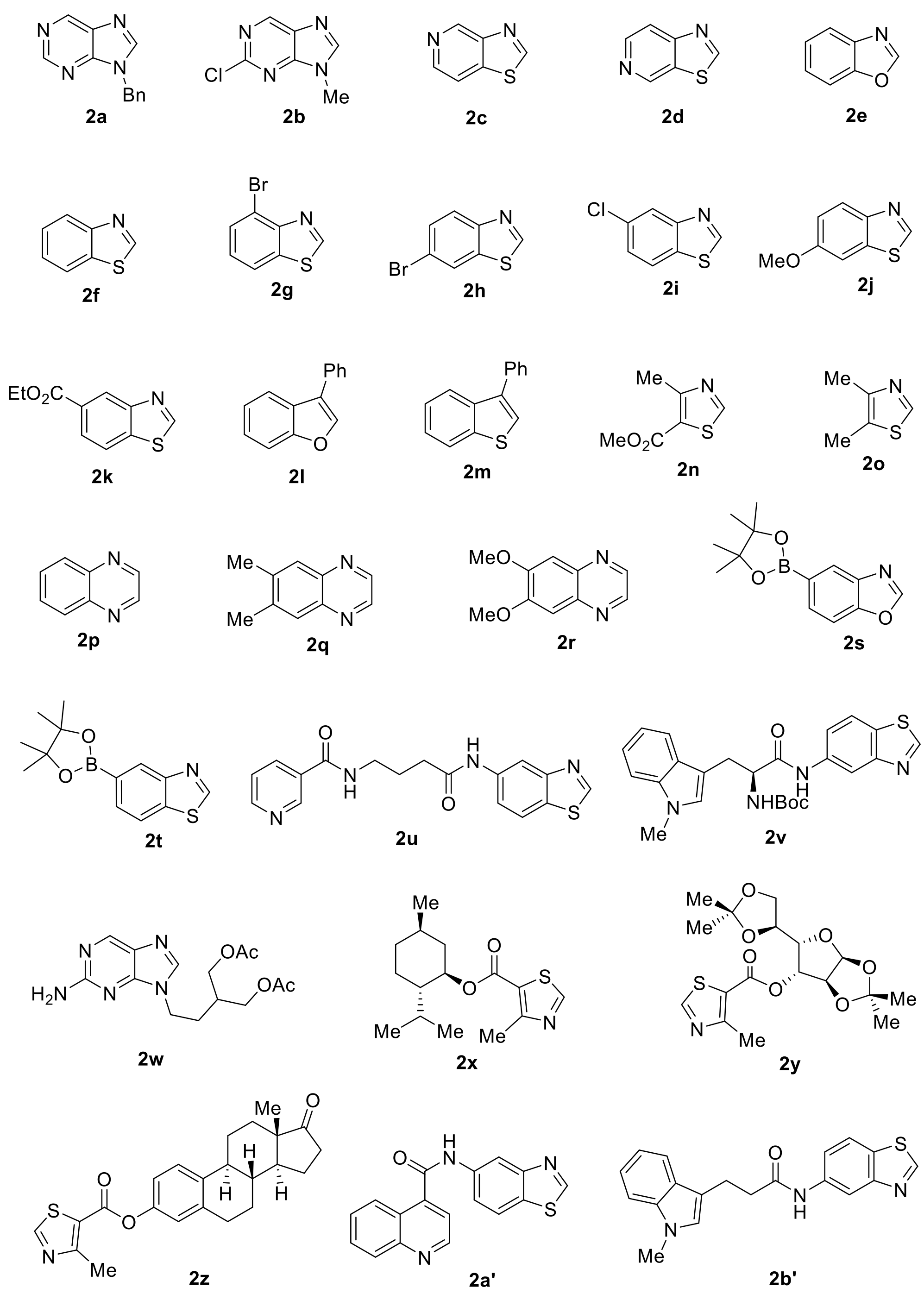


**Supplementary Figure 2.** Structures of heteroarenes

**Supplementary Table 1. Optimization of Reaction Conditions**[a]

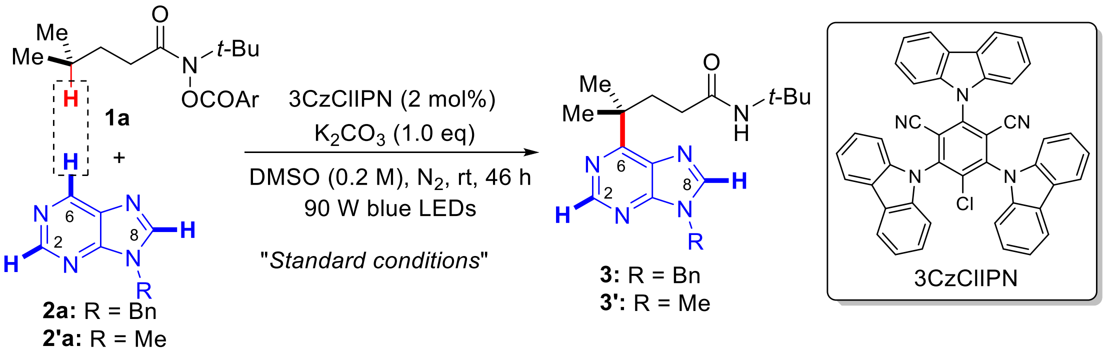


| Entry | Variation of reaction conditions | Yield(%)[b] |
|---|---|---|
| 1 | none | 89 |
| 2 | without light | 0 |
| 3 | air instead of $N_2$ | 0 |
| 4 | without $K_2CO_3$ | 16 |
| 5 | without 3CzClIPN | 65 |
| 6 | $Ir(ppy)_3$ instead of 3CzClIPN | 46 |
| 7 | $Ru(bpy)_3Cl_2$ instead of 3CzClIPN | 27 |
| 8 | Eosin Y instead of 3CzClIPN | 54 |
| 9 | 4CzIPN instead of 3CzClIPN | 67 |
| 10 | $Na_2HPO_4$ instead of $K_2CO_3$ | 78 |
| 11 | DIEA instead of $K_2CO_3$ | 61 |
| 12 | $CH_3CN$ instead of DMSO | 27 |
| 13 | MeOH instead of DMSO | 0 |
| 14 | 45 W CFL instead of 90 W blue LEDs | 10 |
| 15[c] | **2'a** instead of **2a** | 76(**3'**) |

[a]Reaction conditions: **1a** (0.2 mmol, 1.0 equiv), **2a** (0.5 mmol), base (0.2 mmol), photocatalyst (2 mol%), solvent (1.0 mL), rt, 90 W blue LEDs.

[b]Isolated yield.

[c]Together with 8% yield of C8 alkylated regioisomer. Ar = *p*-$CF_3C_6H_4$

**Supplementary Table 2. Examination of Photocatalysts**[a]

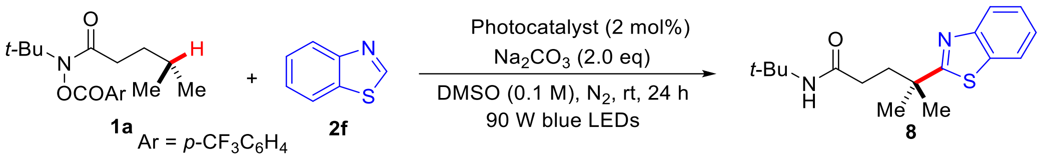


| Entry | Photocatalyst | Conv. (%) | Yield(%)[b] |
|---|---|---|---|
| 1 | $Acr^{+}$-Mes $ClO_4^{-}$ | 100 | 50 |
| 2 | $Ru(bpy)_3Cl_2$ | 100 | 40 |
| 3 | $Ir(ppy)_3$ | 100 | 22 |
| 4 | $[Ir(ppy)_2(dtbbpy)]PF_6$ | 100 | 43 |
| 5 | $Ir(dFCF_3ppy)_2(dtbbpy)PF_6$ | 100 | 51 |
| 6 | Eosin Y | 100 | 52 |
| 7 | Eosin B | 99 | 46 |
| 8 | Fluorescein | 100 | 39 |
| 9 | Rose bengal | 100 | 52 |
| 10 | 4CzIPN | 100 | 64 |
| 11[c] | 4CzIPN | 100 | 74 |
| 12[c] | 3DPA2FBN | 100 | 58 |
| 13[c] | 3CzClIPN | 100 | 79 |

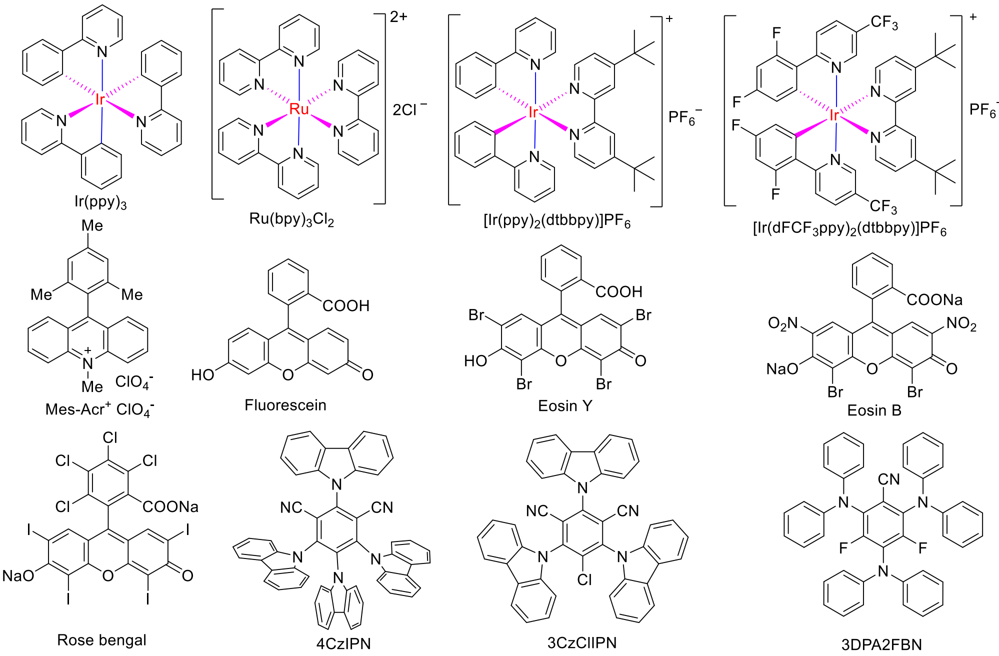

[a]**1a** (0.1 mmol, 1.0 equiv), **2f** (3.0 equiv), $Na_2CO_3$ (2.0 equiv), Photocatalyst (2 mol%), DMSO (1.0 mL), 90 W blue LEDs.

[b]The yield was determined by GC in the presence of tetradecane as an internal standard.

[c]**1a** (0.1 mmol, 1.0 equiv), **2f** (3.0 equiv), $Na_2HPO_4$ (2.0 equiv), Photocatalyst (2% mol), DMSO (1.0 mL), 90 W blue LEDs.

**Supplementary Table 3. Examination of Solvents[a]**

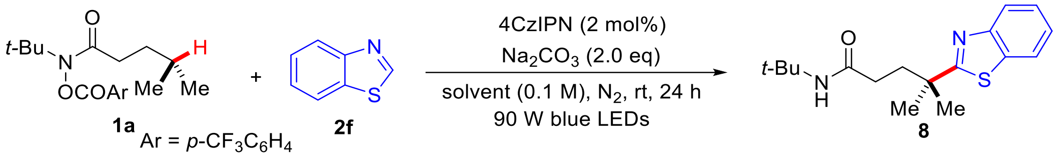


| Entry | Solvent | Conv. (%) | Yield(%)[b] |
|---|---|---|---|
| 1 | DCE | 5 | 0 |
| 2 | EA | 20 | 11 |
| 3 | $PhCF_3$ | 7 | 0 |
| 4 | THF | 85 | 32 |
| 5 | 1,4-dioxane | 11 | 5 |
| 6 | $CH_3CN$ | 16 | 7 |
| 7 | DMF | 100 | 62 |
| 8 | DMSO | 100 | 64 |
| 9 | NMP | 99 | 53 |
| 10 | MeOH | 100 | 2 |
| 11 | HFIP | 14 | 0 |

[a]**1a** (0.1 mmol, 1.0 equiv), **2f** (3.0 equiv), $Na_2CO_3$ (2.0 equiv), 4CzIPN (2 mol%), Solvent (1.0 mL), 90 W blue LEDs.

[b]The yield was determined by GC in the presence of tetradecane as an internal standard.

**Supplementary Table 4. Examination of Additives[a]**

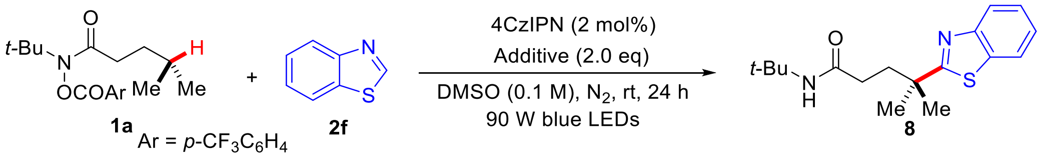


| Entry | Additive | Conv. (%) | Yield(%)[b] |
|---|---|---|---|

| | | | |
|---|---|---|---|
| 1 | $NaHCO_3$ | 99 | 75 |
| 2 | $K_2HPO_4$ | 100 | 77 |
| 3 | $Na_2HPO_4$ | 100 | 78 |
| 4 | $Na_2CO_3$ | 100 | 74 |
| 5 | $K_2CO_3$ | 100 | 77 |
| 6 | $Cs_2CO_3$ | 100 | 57 |
| 7 | $K_3PO_4$ | 100 | 77 |
| 8 | *t*-BuOK | 100 | 3 |
| 9 | CsF | 100 | 66 |
| 10 | NaOAc | 100 | 70 |
| 11 | NaOH | 100 | 12 |
| 12 | TFA | 24 | 8 |
| 13 | TsOH | 14 | 3 |

[a]**1a** (0.1 mmol, 1.0 equiv), **2f** (3.0 equiv), Additive (2.0 equiv), 4CzIPN (2 mol%), DMSO (1.0 mL), 90 W blue LEDs.
[b]The yield was determined by GC in the presence of tetradecane as an internal standard.

**Supplementary Table 5. Examination of Temperatures[a]**

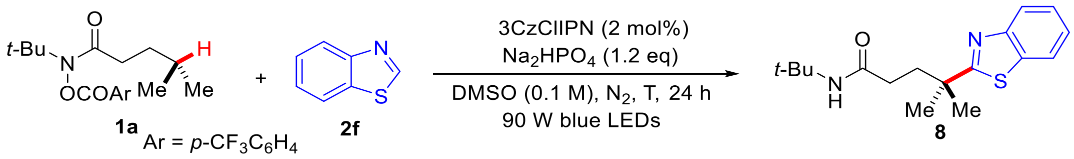


| Entry | T (ºC) | Conv. (%) | Yield(%)[b] |
|---|---|---|---|
| 1 | rt (24±2) | 100 | 76 |
| 2 | 35 | 100 | 72 |
| 3 | 50 | 100 | 67 |

[a]**1a** (0.1 mmol, 1.0 equiv), **2f** (3.0 equiv), $Na_2HPO_4$ (1.2 equiv), 3CzClIPN (2 mol%), DMSO (1.0 mL), 90 W blue LEDs.
[b]The yield was determined by GC in the presence of tetradecane as an internal standard.

**Supplementary Table 6. Examination of Light sources[a]**

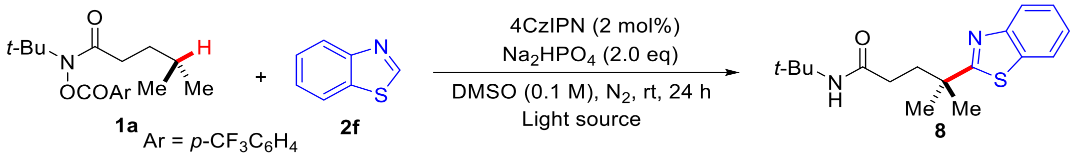


| Entry | Light source | Conv. (%) | Yield(%)[b] |
|---|---|---|---|
| 1 | 26 W blue LED strips | 10 | 3 |
| 2 | 90 W blue LEDs | 100 | 78 |
| 3 | 90 W white LEDs | 100 | 76 |
| 4 | 45 W CFL | 58 | 41 |

[a]**1a** (0.1 mmol, 1.0 equiv), **2f** (3.0 equiv), $Na_2HPO_4$ (2.0 equiv), 4CzIPN (2 mol%), DMSO (1.0 mL).

[b]The yield was determined by GC in the presence of tetradecane as an internal standard.

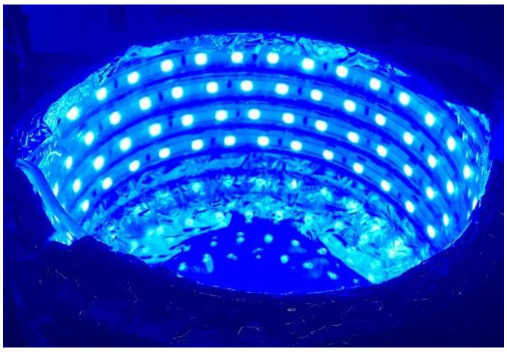

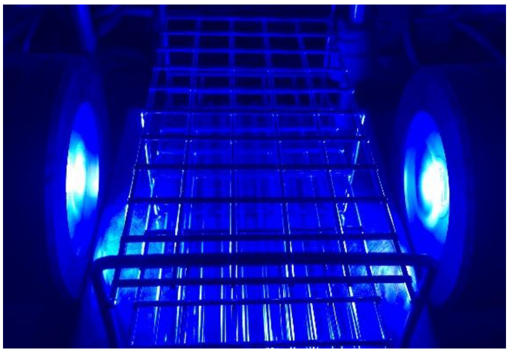

**26 W blue LED strips** **90 W blue LEDs**

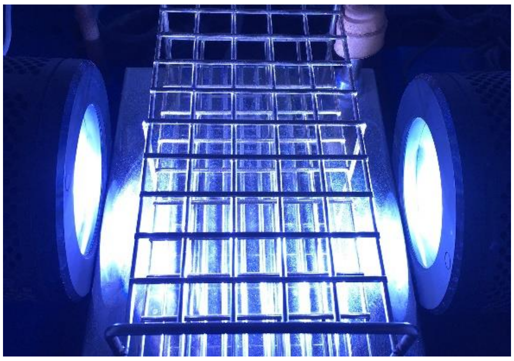

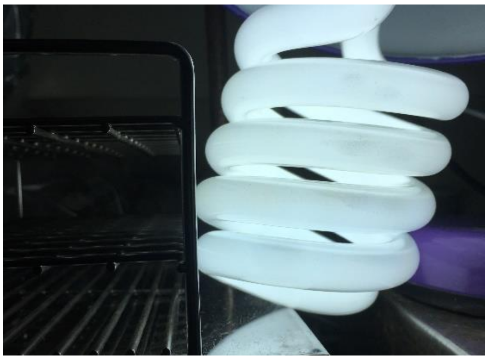

**90 W white LEDs** **45 W CFL**

**Supplementary Figure 3.** Light sources examined for the interrupted HLF reaction

**General Procedure for the Preparation of Substrates**

**General Procedure A:**

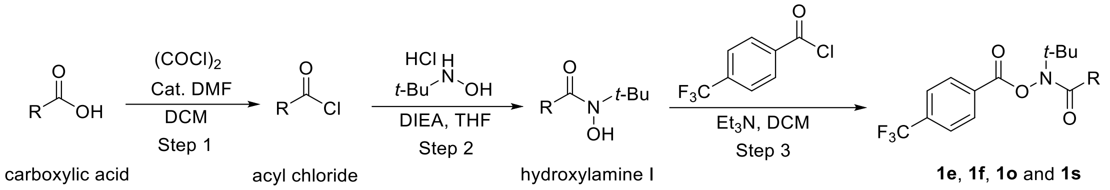


**Step 1:** To a solution of carboxylic acid (1.0 equiv) and 3-5 drops of anhydrous DMF in anhydrous $CH_2Cl_2$ (0.5 M) at 0 ˚C, oxalyl chloride (1.5 equiv) was added dropwise over 10 minutes. The reaction was vigorously stirred at room temperature for 3 h. The solvent was removed in vacuum. Anhydrous $CH_2Cl_2$ was added to remove the residual of oxalyl chloride in vacuum. Then the resulting acyl chloride was redissolved in anhydrous acetonitrile and used directly for the next step without further purification.

**Step 2:** A solution of the *N*-(*tert*-butyl)hydroxylamine hydrochloride in anhydrous THF (0.4 M) was cooled to 0 ºC, treated with DIPEA (2.0 equiv) and stirred for 15 minutes. The acyl chloride (1.0 equiv) in anhydrous acetonitrile was added dropwise over 15 minutes and the mixture was allowed to warm to room temperature overnight. The mixture was diluted with saturated $NaHCO_3$ and EtOAc and the layers were separated. The aqueous layer was extracted with EtOAc (2 x) and the combined organic layers were washed with 1 M HCl, saturated $NaHCO_3$ and brine, successively, and then evaporated. Purification by column chromatography on silica gel eluting with Petroleum ether and EtOAc gave the hydroxylamine I.

**Step 3:** To a solution of hydroxylamine I (1.05 equiv) in anhydrous $CH_2Cl_2$ (0.35 M) at 0 ˚C, $Et_3N$ (1.5 equiv) was added dropwise. 4-trifluoromethyl-benzoyl chloride (1.0 equiv) was then added dropwise over 5 minutes. The reaction was vigorously stirred at room temperature for 2 h. After removal of the solvent, the resulting residue was added saturated $NaHCO_3$ and THF, and stirred for 30 minutes. Then, the layers were separated. The aqueous layer was extracted with EtOAc again and the combined organic layers were washed with 1 M HCl, saturated $NaHCO_3$ and brine, successively, and then evaporated. Purification by column chromatography on silica gel gave **1e**, **1f**, **1o** and **1s**.

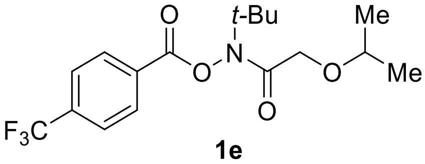

**1e**

***N*-(*tert*-butyl)-2-isopropoxy-*N*-((4-(trifluoromethyl)benzoyl)oxy)acetamide (1e)**: Prepared according to **General Procedure A** from commercially available 2-isopropoxyacetic acid. 34% yield for 3 steps, white solid. Flash column chromatography conditions (for step 3): Petroleum ether/ $Et_2O$ = 10/1 to 5/1. Mp 56-58 °C. $^1$H NMR (400 MHz, $CDCl_3$) δ 8.20 (d, *J* = 8.1 Hz, 2H), 7.79 (d, *J* = 8.2 Hz, 2H), 4.06 (d, *J* = 14.9 Hz, 1H), 3.93 (d, *J* = 14.9 Hz, 1H), 3.68 – 3.56 (m, 1H), 1.49 (s, 9H), 1.10 (d, *J* = 5.1 Hz, 6H). $^{13}$C NMR (100 MHz, $CDCl_3$) δ 171.69, 164.48, 135.94 (q, *J* = 33.1 Hz), 130.52, 130.18, 126.15 (q, *J* = 3.7 Hz), 123.44 (q, *J* = 272.9 Hz), 72.49, 67.31, 63.49, 27.31, 21.91, 21.70. $^{19}$F NMR (376 MHz, $CDCl_3$) δ -63.39. HRMS (ESI) *m/z* calcd. for $C_{17}H_{22}F_3NO_4Na$ $[M+Na]^+$ 384.1399, found 384.1392.

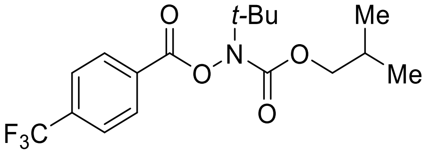

**1f**

**Isobutyl *tert*-butyl((4-(trifluoromethyl)benzoyl)oxy)carbamate (1f)**: Prepared according to **General Procedure A** from commercially available isobutyl chloroformate. 68% yield for 2 steps, colorless oil. Flash column chromatography conditions (for step 3): Petroleum ether/ $Et_2O$ = 50/1 to 20/1. $^1$H NMR (400 MHz, $CDCl_3$) δ 8.19 (d, *J* = 8.2 Hz, 2H), 7.75 (d, *J* = 8.3 Hz, 2H), 3.90 (s, 2H), 1.93 – 1.80 (m, 1H), 1.48 (s, 9H), 0.83 (d, *J* = 6.7 Hz, 6H). $^{13}$C NMR (100 MHz, $CDCl_3$) δ 163.87, 155.96, 135.32 (q, *J* = 32.8 Hz), 131.18, 130.36, 125.86 (q, *J* = 3.8 Hz), 123.59 (q, *J* = 272.6 Hz), 72.47, 61.97, 27.94, 27.74, 19.06. $^{19}$F NMR (376 MHz, $CDCl_3$) δ -63.29. HRMS (ESI) *m/z* calcd. for $C_{17}H_{22}F_3NO_4Na$ $[M+Na]^+$ 384.1399, found 384.1400.

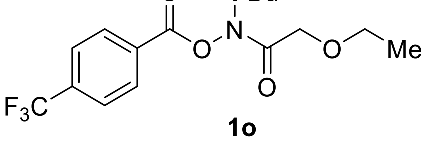

**1o**

***N*-(*tert*-butyl)-2-ethoxy-*N*-((4-(trifluoromethyl)benzoyl)oxy)acetamide (1o)**: Prepared according to **General Procedure A** from commercially available 2-ethoxyacetic acid. 41% yield for 3 steps, colorless oil. Flash column chromatography conditions (for step 3): Petroleum ether/ $Et_2O$ = 10/1 to 2/1. $^1$H NMR (400 MHz, $CDCl_3$) δ 8.20 (d, *J* = 8.1 Hz, 2H), 7.79 (d, *J* = 8.2 Hz, 2H), 4.06 (d, *J* = 14.9 Hz, 1H), 3.94 (d, *J* = 14.9 Hz, 1H), 3.50 (p, *J* = 6.8 Hz, 2H), 1.49 (s, 9H), 1.14 (t, *J* = 7.0 Hz, 3H). $^{13}$C NMR (100 MHz, $CDCl_3$) δ 171.31, 164.48, 135.95 (q, *J* = 33.2 Hz), 130.50, 130.13, 126.15 (q, *J* = 4.0 Hz), 123.43 (q, *J* = 273.1 Hz), 69.87, 67.24, 63.52, 27.29, 15.05. $^{19}$F NMR (376 MHz, $CDCl_3$) δ -63.39. HRMS (ESI) *m/z* calcd. for $C_{16}H_{20}F_3NO_4Na$ $[M+Na]^+$ 370.1242, found 370.1245.

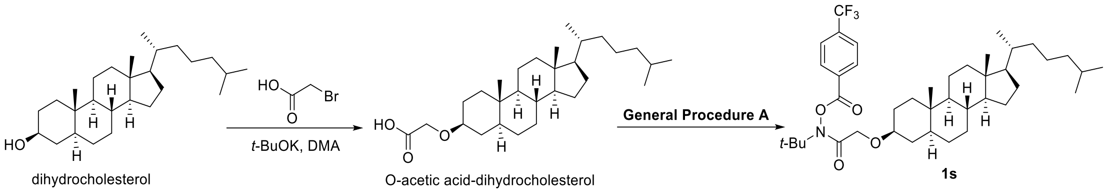


***N*-(*tert*-butyl)-2-(((*3S,5S,8R,9S,10S,13R,14S,17R*)-10,13-dimethyl-17-((*R*)-6-methylheptan-2-yl)hexadecahydro-1*H*-cyclopenta[*a*]phenanthren-3-yl)oxy)-*N*-((4-(trifluoromethyl)benzoyl)oxy)acetamide (1s)**: A stirred solution of dihydrocholesterol (5 g, 12.86 mmol, 1 equiv) in anhydrous DMA (40 mL) was added *t*-BuOK (7.21 g, 64.32 mmol, 5 equiv), and then the mixture was rose to 50°C. Bromoacetic acid (3.57 g, 25.73 mmol, 2 equiv) in anhydrous DMA (15 mL) was then added dropwise and stirred at 70 °C for another 4 h. The mixture was quenched by pouring into ice water slowly. After adjusting to pH 1-2 with 6 M HCl, the mixture was extracted with EtOAc (3 x). The combined organic phases were then dried over $Na_2SO_4$ and concentrated under vacuum. The residue was recrystallized with EtOAc and n-hexane, afforded O-acetic acid-dihydrocholesterol (3.91g, 68%) as a white solid. **1s** was then synthesized according to **General Procedure A.** 27% yield for 3 steps, white solid. Flash column chromatography conditions (for step 3): Petroleum ether/ $Et_2O$ = 15/1 to 5/1. Mp 140-141 °C. $^1$H NMR (400 MHz, $CDCl_3$) δ 8.21 (d, *J* = 8.1 Hz, 2H), 7.79 (d, *J* = 8.3 Hz, 2H), 4.15 – 4.04 (m, 1H), 4.03 – 3.92 (m, 1H), 3.32 – 3.20 (m, 1H), 1.93 (dt, *J* = 12.5, 3.4 Hz, 1H), 1.85 – 1.73 (m, 2H), 1.49 (s, 16H), 1.37 – 1.23 (m, 6H), 1.23 –

0.91 (m, 14H), 0.88 (d, $J$ = 6.5 Hz, 3H), 0.86 (d, $J$ = 1.8 Hz, 3H), 0.84 (d, $J$ = 1.8 Hz, 3H), 0.71 (s, 3H), 0.62 (s, 3H), 0.56 (td, $J$ = 12.1, 4.0 Hz, 1H). $^{13}C$ NMR (100 MHz, $CDCl_3$) δ 171.84, 164.46, 135.93 (q, $J$ = 32.9 Hz), 130.55, 130.23, 126.14 (q, $J$ = 3.8 Hz), 123.45 (q, $J$ = 273.0 Hz), 79.36, 67.09, 63.49, 56.61, 56.41, 54.49, 44.81, 42.72, 40.17, 39.65, 36.98, 36.31, 35.92, 35.79, 35.60, 34.41, 34.25, 32.20, 28.89, 28.38, 28.15, 27.97, 27.76, 27.34, 24.34, 23.96, 22.95, 22.70, 21.34, 18.80, 12.32, 12.20. $^{19}F$ NMR (376 MHz, $CDCl_3$) δ -63.36. HRMS (ESI) *m/z* calcd. for $C_{41}H_{63}F_3NO_4$ $[M+H]^+$ 690.4709, found 690.4667.

**General Procedure B:**

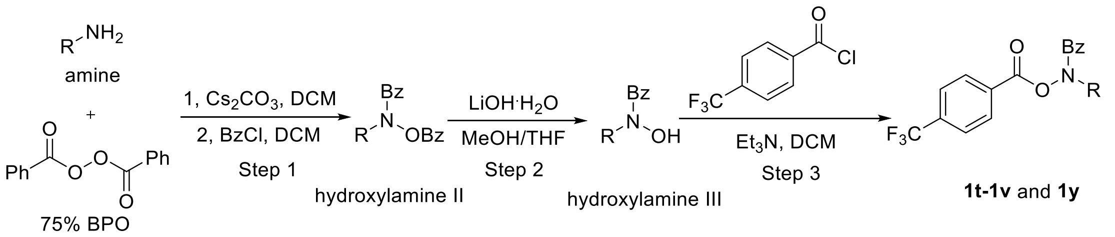


**Step 1:** Following the literature's procedure[8], BPO (wetted with ca. 25% $H_2O$) (2.0 equiv) and $Cs_2CO_3$ (3.0 equiv) were taken in a round bottom flask equipped with a magnetic stir bar. DCM (0.1 M) was added to it and the heterogeneous mixture was stirred for 2 h at room temperature. After that a solution of amine (1.0 equiv, in DCM (0.25 M)) was then added and the mixture was further vigorously stirred for 14 h. Then a solution of BzCl (2.0 equiv, in DCM (0.5 M)) was added to it and stirred continued for another 6 h. Then water was added to the reaction mixture and stirred for 15 min and extracted with DCM. The organic layer was washed with sat. $NaHCO_3$ solution, brine, and concentrated to get crude product. The crude product was purified by silica gel column chromatography using petroleum ether/ EtOAc as eluent gave the hydroxylamine II.

**Step 2:** Following the literature's procedure[8] with slight modifications, to a stirred solution of the hydroxylamine II (1.0 equiv) in MeOH (0.5 M) and THF (1.0 M) was added $LiOH{\cdot}H_2O$ (3.0 equiv). After 0.5-1 h, the reaction mixture was concentrated under reduced pressure and then to this water was added and extracted with EtOAc,

washed with brine, and concentrated under reduced pressure to get crude product. The crude product was purified by silica gel column chromatography using petroleum ether/ EtOAc as eluent gave the hydroxylamine III.

**Step 3:** To a solution of the hydroxylamine III (1.0 equiv) in anhydrous $CH_2Cl_2$ (0.35 M) at 0 ˚C, $Et_3N$ (1.5 equiv) was added dropwise. 4-trifluoromethyl-benzoyl chloride (for **1y**, using 4-methylpentanoyl chloride generated from step 1 of **General Procedure A**) (1.0 equiv) was then added dropwise over 5 minutes. The reaction was vigorously stirred at room temperature for 2 h. After removal of the solvent, the resulting residue was added saturated $NaHCO_3$ and THF and stirred for 30 minutes. Then, the layers were separated. The aqueous layer was extracted with EtOAc again and the combined organic layers were washed with 1 M HCl, saturated $NaHCO_3$ and brine, successively, and then evaporated. Purification by column chromatography on silica gel eluting with Petroleum ether/ EtOAc gave **1t-1v** and **1y**.

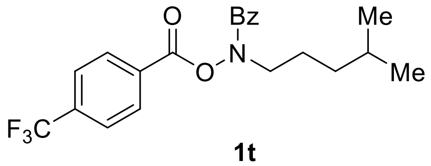


**1t**

***N*-(4-methylpentyl)-*N*-((4-(trifluoromethyl)benzoyl)oxy)benzamide (1t)**: Prepared according to **General Procedure B** from commercially available 4-methylpentan-1-amine. 43% yield for 3 steps, colorless oil. Flash column chromatography conditions (for step 3): Petroleum ether/ EtOAc = 20/1 to 10/1. $^1$H NMR (400 MHz, $CDCl_3$) δ 8.08 (d, *J* = 8.2 Hz, 2H), 7.71 (d, *J* = 8.3 Hz, 2H), 7.64 – 7.59 (m, 2H), 7.45 – 7.33 (m, 3H), 3.86 (t, *J* = 7.3 Hz, 2H), 1.79 – 1.69 (m, 2H), 1.61 – 1.50 (m, 1H), 1.31 – 1.22 (m, 2H), 0.89 (s, 3H), 0.87 (s, 3H). $^{13}$C NMR (100 MHz, $CDCl_3$) δ 170.46, 163.36, 135.60 (q, *J* = 32.9 Hz), 133.78, 131.12, 130.49, 130.40, 128.45, 127.80, 125.86 (q, *J* = 3.8 Hz), 123.47 (q, *J* = 272.7 Hz), 50.91, 35.78, 27.81, 25.23, 22.60. $^{19}$F NMR (376 MHz, $CDCl_3$) δ -63.35. HRMS (ESI) *m/z* calcd. for $C_{21}H_{23}F_3NO_3$ $[M+H]^+$ 394.1630, found 394.1632.

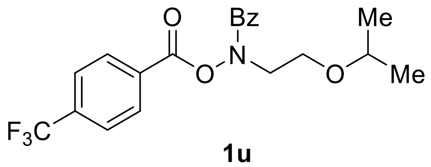


**1u**

***N*-(2-isopropoxyethyl)-*N*-((4-(trifluoromethyl)benzoyl)oxy)benzamide (1u)**: Prepared according to **General Procedure B** from commercially available 2-isopropoxyethan-1-amine. 45% yield for 3 steps, white solid. Flash column chromatography conditions (for step 3): Petroleum ether/ EtOAc = 10/1 to 5/1. Mp 103-105 °C. $^{1}$H NMR (400 MHz, $CDCl_3$) δ 8.10 (d, *J* = 8.2 Hz, 2H), 7.71 (d, *J* = 8.3 Hz, 2H), 7.69 – 7.65 (m, 2H), 7.45 – 7.33 (m, 3H), 4.06 (t, *J* = 5.3 Hz, 2H), 3.71 (t, *J* = 5.3 Hz, 2H), 3.64 – 3.53 (m, 1H), 1.10 (s, 3H), 1.08 (s, 3H). $^{13}$C NMR (100 MHz, $CDCl_3$) δ 170.65, 163.33, 135.48 (q, *J* = 32.7 Hz), 133.57, 131.17, 130.75, 130.45, 128.39, 128.17, 125.78 (q, *J* = 3.8 Hz), 123.50 (q, *J* = 273.0 Hz), 72.24, 64.72, 51.12, 22.04. $^{19}$F NMR (376 MHz, $CDCl_3$) δ -63.31. HRMS (ESI) *m/z* calcd. for $C_{20}H_{21}F_3NO_4$ $[M+H]^+$ 396.1422, found 396.1433.

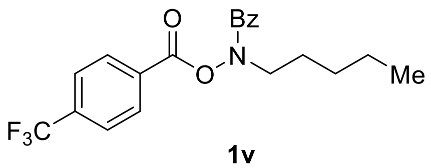


**1v**

***N*-pentyl-*N*-((4-(trifluoromethyl)benzoyl)oxy)benzamide (1v)**: Prepared according to **General Procedure B** from commercially available pentan-1-amine. 37% yield for 3 steps, colorless oil. Flash column chromatography conditions (for step 3): Petroleum ether/ EtOAc = 20/1 to 10/1. $^{1}$H NMR (400 MHz, $CDCl_3$) δ 8.08 (d, *J* = 8.2 Hz, 2H), 7.71 (d, *J* = 8.3 Hz, 2H), 7.64 – 7.59 (m, 2H), 7.44 – 7.33 (m, 3H), 3.87 (t, *J* = 7.3 Hz, 2H), 1.74 (p, *J* = 7.4 Hz, 2H), 1.41 – 1.28 (m, 4H), 0.89 (t, *J* = 7.0 Hz, 3H). $^{13}$C NMR (100 MHz, $CDCl_3$) δ 170.45, 163.37, 135.59 (q, *J* = 32.8 Hz), 133.78, 131.12, 130.50, 130.40, 128.45, 127.80, 125.85 (q, *J* = 3.7 Hz), 123.47 (q, *J* = 273.0 Hz), 50.69, 28.86, 27.06, 22.40, 14.04. $^{19}$F NMR (376 MHz, $CDCl_3$) δ -63.35. HRMS (ESI) *m/z* calcd. for $C_{20}H_{21}F_3NO_3$ $[M+H]^+$ 380.1473, found 380.1487.

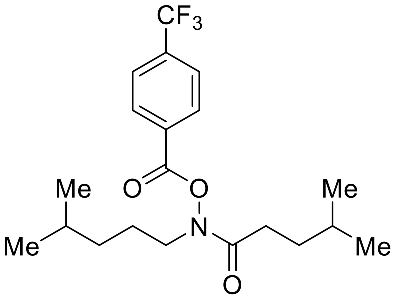

**1y**

**4-methyl-*N*-(4-methylpentyl)-*N*-((4-(trifluoromethyl)benzoyl)oxy)pentanamide (1y)**: Prepared according to **General Procedure B** from commercially available 4-methylpentan-1-amine. 43% yield for 3 steps, colorless oil. Flash column chromatography conditions (for step 3): Petroleum ether/ EtOAc = 20/1 to 10/1. $^{1}$H NMR (400 MHz, $CDCl_3$) δ 8.22 (d, *J* = 8.2 Hz, 2H), 7.79 (d, *J* = 8.2 Hz, 2H), 3.79 (t, *J* = 7.3 Hz, 2H), 2.29 (t, *J* = 7.2 Hz, 2H), 1.69 – 1.49 (m, 6H), 1.29 – 1.19 (m, 2H), 0.91 – 0.80 (m, 12H). $^{13}$C NMR (100 MHz, $CDCl_3$) δ 163.52, 135.87 (q, *J* = 33.7 Hz), 130.51, 126.06 (q, *J* = 4.3 Hz), 123.47 (q, *J* = 273.1 Hz), 35.83, 33.38, 30.59, 27.84, 27.77, 25.14, 22.61, 22.43. $^{19}$F NMR (376 MHz, $CDCl_3$) δ -63.37. HRMS (ESI) *m/z* calcd. for $C_{20}H_{29}F_3NO_3$ $[M+H]^+$ 388.2099, found 388.2106.

**General Procedure C:**

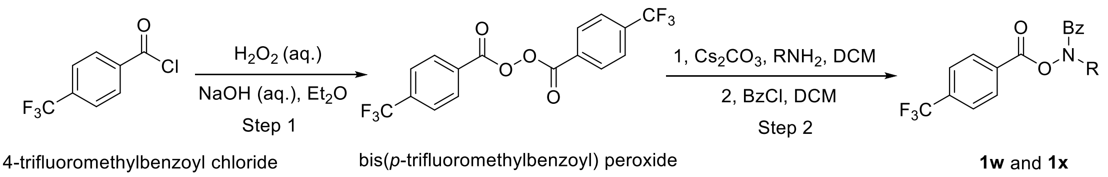


**Step 1:** Following the literature's procedure[9], hydrogen peroxide (13.87 g, 30 wt. % in $H_2O$, 122.36 mmol) was added dropwise over 10 min to a cold solution of 4-trifluoromethylbenzoyl chloride (44.0 g, 210.97 mmol) in diethyl ether (44 mL). This was followed by the dropwise addition of an aqueous solution of NaOH (10.63 g, 265.82 mmol, in 70 mL $H_2O$) over 20 min. The resulting white precipitate was collected by filtration. After washing with water (2 × 25 mL) and diethyl ether (2 × 25 mL), the solid was crystallized from a cold acetone / water mixture (200 mL, 1:1 v/v) to give the desired the bis(*p*-trifluoromethylbenzoyl) peroxide as a white solid (33.28 g, 83% yield).

**Step 2:** Following the literature's procedure[8] with slight modifications, bis(p-trifluoromethylbenzoyl) peroxide (2.0 equiv) and $Cs_2CO_3$ (3.0 equiv) were taken in a round bottom flask equipped with a magnetic stir bar. DCM (0.1 M) and $H_2O$ (13.9 equiv) was added to it and the heterogeneous mixture was stirred for 2 h at room temperature. After that a solution of amine (1.0 equiv, in DCM (0.25 M)) was then added and the mixture was further vigorously stirred for 14 h. Then a solution of BzCl (2.0 equiv, in DCM (0.5 M)) was added to it and stirred continued for another 6 h. Then water was added to the reaction mixture and stirred for 15 min and extracted with DCM. The organic layer was washed with sat. $NaHCO_3$ solution, brine, and concentrated to get crude product. The crude product was purified by silica gel column chromatography using petroleum ether/ EtOAc as eluent gave **1w** and **1x**.

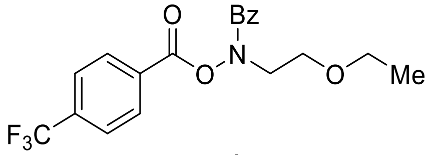


**1w**

***N*-(2-ethoxyethyl)-*N*-((4-(trifluoromethyl)benzoyl)oxy)benzamide (1w)**: Prepared according to **General Procedure C** from commercially available 2-ethoxyethan-1-amine. 65% yield, white solid. Flash column chromatography conditions: Petroleum ether/ EtOAc = 20/1 to 5/1. Mp 68-70 °C. $^1$H NMR (400 MHz, $CDCl_3$) δ 8.09 (d, *J* = 8.2 Hz, 2H), 7.71 (d, *J* = 8.3 Hz, 2H), 7.68 – 7.64 (m, 2H), 7.44 – 7.33 (m, 3H), 4.08 (t, *J* = 5.3 Hz, 2H), 3.72 (t, *J* = 5.3 Hz, 2H), 3.46 (q, *J* = 7.0 Hz, 2H), 1.08 (t, *J* = 7.0 Hz, 3H). $^{13}$C NMR (100 MHz, $CDCl_3$) δ 170.73, 163.40, 135.50 (q, *J* = 33.0 Hz), 133.50, 131.20, 130.69, 130.43, 128.39, 128.13, 125.79 (q, *J* = 3.8 Hz), 123.49 (q, *J* = 272.9 Hz), 67.25, 66.73, 50.56, 15.13. $^{19}$F NMR (376 MHz, $CDCl_3$) δ -63.33. HRMS (ESI) *m/z* calcd. for $C_{19}H_{19}F_3NO_4$ $[M+H]^+$ 382.1266, found 382.1274.

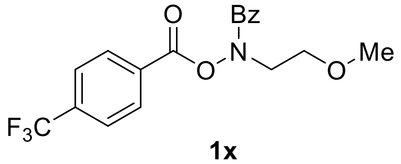


**1x**

***N*-(2-methoxyethyl)-*N*-((4-(trifluoromethyl)benzoyl)oxy)benzamide (1x)**: Prepared according to **General Procedure C** from commercially available 2-methoxyethan-1-

amine. 65% yield, colorless oil. Flash column chromatography conditions: Petroleum ether/ EtOAc = 10/1 to 3/1. $^{1}H$ NMR (400 MHz, $CDCl_3$) δ 8.08 (d, *J* = 8.2 Hz, 2H), 7.70 (d, *J* = 8.3 Hz, 2H), 7.68 – 7.63 (m, 2H), 7.44 – 7.33 (m, 3H), 4.08 (t, *J* = 5.3 Hz, 2H), 3.69 (t, *J* = 5.3 Hz, 2H), 3.31 (s, 3H). $^{13}C$ NMR (100 MHz, $CDCl_3$) δ 170.79, 163.48, 135.51 (q, *J* = 32.9 Hz), 133.40, 131.23, 130.58, 130.39, 128.39, 128.11, 125.81 (q, *J* = 3.8 Hz), 123.47 (q, *J* = 272.9 Hz), 69.28, 58.96, 50.27. $^{19}F$ NMR (376 MHz, $CDCl_3$) δ -63.34. HRMS (ESI) *m/z* calcd. for $C_{18}H_{17}F_3NO_4$ $[M+H]^+$ 368.1109, found 368.1118.

**General Procedure D:**

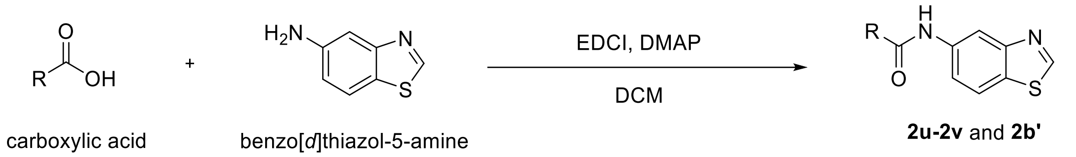


To a solution of EDCI (1.3 equiv) and DMAP (1.4 equiv) in DCM (0.2 M) was added carboxylic acid (1.0 equiv) at room temperature. After the mixture was stirred for 10 min, benzo[*d*]thiazol-5-amine (1.2 equiv) was added. The reaction was vigorously stirred at room temperature overnight. Then, the mixture was poured into saturated $NaHCO_3$ and extracted with DCM twice. The combined organic phases were then dried over $Na_2SO_4$ and concentrated under vacuum. Purification by column chromatography on silica gel or crystallization gave **2u-2v** and **2b'**.

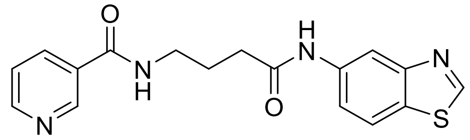


**2u**

***N*-(4-(benzo[*d*]thiazol-5-ylamino)-4-oxobutyl)nicotinamide (2u)**: Prepared according to **General Procedure D** from commercially available pikamilone. 66% yield, white solid. Crystallization conditions: EtOAc. Mp 205-206 °C. $^{1}H$ NMR (400 MHz, DMSO-$d_6$) δ 10.22 (s, 1H), 9.35 (s, 1H), 9.06 – 8.99 (m, 1H), 8.75 (t, *J* = 5.4 Hz, 1H), 8.68 (dd, *J* = 4.8, 1.6 Hz, 1H), 8.50 (d, *J* = 1.8 Hz, 1H), 8.20 (dt, *J* = 7.9, 1.9 Hz, 1H), 8.04 (d, *J* = 8.7 Hz, 1H), 7.61 (dd, *J* = 8.7, 1.9 Hz, 1H), 7.51 – 7.45 (m, 1H), 3.37

(q, $J$ = 6.9 Hz, 2H), 2.46 (t, $J$ = 7.4 Hz, 2H), 1.91 (p, $J$ = 7.2 Hz, 2H). $^{13}$C NMR (100 MHz, DMSO-$d_6$) δ 171.15, 164.80, 156.78, 153.58, 151.70, 148.38, 137.93, 134.90, 130.01, 127.59, 123.36, 122.21, 117.96, 112.62, 38.85, 33.91, 24.96. HRMS (ESI) *m/z* calcd. for $C_{17}H_{17}N_4O_2S$ $[M+H]^+$ 341.1072, found 341.1062.

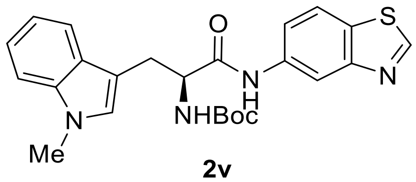


**2v**

***Tert*-butyl (*S*)-(1-(benzo[d]thiazol-5-ylamino)-3-(1-methyl-1*H*-indol-3-yl)-1-oxopropan-2-yl)carbamat*e* (2v)**: Prepared according to **General Procedure D** from *N*-(*tert*-butoxycarbonyl)-1-methyl-*L*-tryptophan. 59% yield, white solid. Flash column chromatography conditions: n-hexane/ THF = 2/1. Mp 101-104 °C. $^1$H NMR (400 MHz, $CDCl_3$) δ 8.95 (s, 1H), 8.17 (s, 1H), 8.09 (d, $J$ = 1.9 Hz, 1H), 7.75 (d, $J$ = 8.6 Hz, 1H), 7.67 (d, $J$ = 7.9 Hz, 1H), 7.37 (dd, $J$ = 8.6, 1.7 Hz, 1H), 7.29 (d, $J$ = 8.2 Hz, 1H), 7.22 (t, $J$ = 7.3 Hz, 1H), 7.10 (t, $J$ = 7.4 Hz, 1H), 6.93 (s, 1H), 5.37 (s, 1H), 4.66 (s, 1H), 3.68 (s, 3H), 3.39 (dd, $J$ = 14.2, 5.3 Hz, 1H), 3.27 (dd, $J$ = 14.3, 7.1 Hz, 1H), 1.44 (s, 9H). $^{13}$C NMR (100 MHz, $CDCl_3$) δ 170.53, 155.06, 153.79, 137.13, 136.23, 129.35, 128.16, 127.88, 122.08, 121.85, 119.54, 119.07, 119.03, 114.66, 109.51, 108.96, 80.63, 56.11, 32.78, 28.43, 28.28. HRMS (ESI) *m/z* calcd. for $C_{24}H_{27}N_4O_3S$ $[M+H]^+$ 451.1804, found 451.1825.

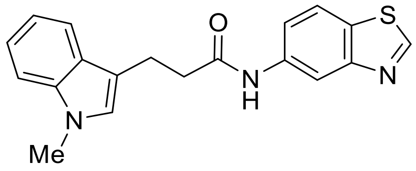


**2b'**

***N*-(benzo[*d*]thiazol-5-yl)-3-(1-methyl-1*H*-indol-3-yl)propanamide (2b')**: Prepared according to **General Procedure D** from 3-(1-methyl-1*H*-indol-3-yl)propanoic acid. 53% yield, white solid. Flash column chromatography conditions: DCM/ EtOAc = 10/1 to 5/1. Mp 165-167 °C. $^1$H NMR (400 MHz, DMSO-$d_6$) δ 10.18 (s, 1H), 9.36 (s, 1H), 8.53 (d, $J$ = 1.8 Hz, 1H), 8.05 (d, $J$ = 8.7 Hz, 1H), 7.65 – 7.59 (m, 2H), 7.36 (d, $J$ = 8.2 Hz, 1H), 7.16 – 7.11 (m, 2H), 7.05 – 7.00 (m, 1H), 3.71 (s, 3H), 3.06 (t, $J$ = 7.6 Hz, 2H), 2.74 (t, $J$ = 7.6 Hz, 2H). $^{13}$C NMR (100 MHz, DMSO-$d_6$) δ 171.13, 156.80, 153.60,

137.93, 136.65, 127.62, 127.32, 126.71, 122.23, 121.07, 118.63, 118.28, 117.99, 113.04, 112.67, 109.52, 37.46, 32.21, 20.64. HRMS (ESI) *m/z* calcd. for $C_{19}H_{18}N_3OS$ $[M+H]^+$ 336.1170, found 336.1162.

**General Procedure E:**

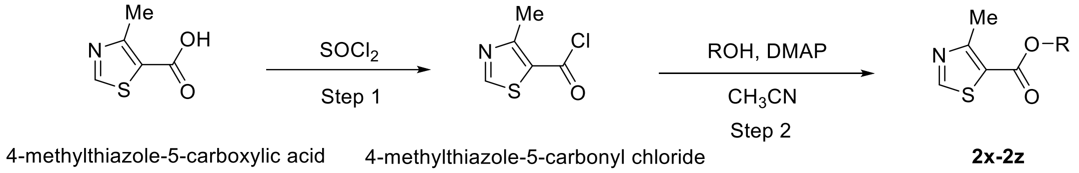


Step 1: 4-Methylthiazole-5-carboxylic acid (2.5 equiv) was added to $SOCl_2$ (1.2 M). After refluxing for 3 hours, the excess $SOCl_2$ was distilled off under reduced pressure. Then the resulting 4-methylthiazole-5-carbonyl chloride was redissolved in anhydrous acetonitrile and used directly for the next step without further purification.

Step 2: To a solution of the alcohol or phenol (1.0 equiv) in anhydrous acetonitrile (0.4 M) was added DMAP (2.5 equiv). The 4-methylthiazole-5-carbonyl chloride in anhydrous acetonitrile was added dropwise and the mixture was allowed to warm to room temperature overnight. The mixture was diluted with saturated $NaHCO_3$ and EtOAc and the layers were separated. The aqueous layer was extracted with EtOAc (2 x) and the combined organic layers were washed with saturated $NaHCO_3$ and brine, and then evaporated. Purification by column chromatography on silica gel eluting with Petroleum ether/ EtOAc gave **2x-2z**.

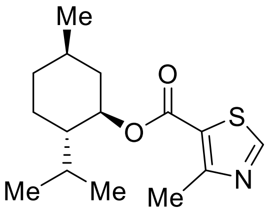


**2x**

**(*1R,2S,5R*)-2-isopropyl-5-methylcyclohexyl 4-methylthiazole-5-carboxylate (2x)**: Prepared according to **General Procedure E** from commercially available *L*-Menthol. 97% yield for 2 steps, colorless oil. Flash column chromatography conditions: Petroleum ether/ EtOAc = 20/1 to 10/1. $^1H$ NMR (400 MHz, $CDCl_3$) δ 8.74 (s, 1H),

4.86 (td, $J$ = 10.9, 4.4 Hz, 1H), 2.76 (s, 3H), 2.15 – 2.06 (m, 1H), 1.97 – 1.85 (m, 1H), 1.75 – 1.66 (m, 2H), 1.60 – 1.44 (m, 2H), 1.16 – 1.03 (m, 2H), 0.94 – 0.87 (m, 7H), 0.78 (d, $J$ = 7.0 Hz, 3H). $^{13}$C NMR (100 MHz, $CDCl_3$) δ 161.85, 160.39, 155.18, 122.89, 75.72, 47.21, 41.10, 34.31, 31.56, 26.66, 23.73, 22.12, 20.86, 17.48, 16.65. HRMS (ESI) *m/z* calcd. for $C_{15}H_{24}NO_2S$ $[M+H]^+$ 282.1527, found 282.1540.

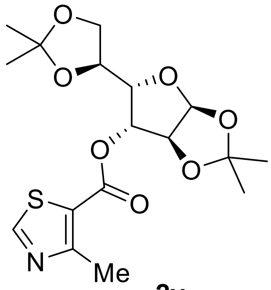


**2y**

**(*3aS,5S,6R,6aS*)-5-((*S*)-2,2-dimethyl-1,3-dioxolan-4-yl)-2,2-dimethyltetrahydro-furo[2,3-*d*][1,3]dioxol-6-yl 4-methylthiazole-5-carboxylate (2y)**: Prepared according to **General Procedure E** from commercially available diacetone-D-glucose. 95% yield for 2 steps, colorless oil. Flash column chromatography conditions: Petroleum ether/ EtOAc = 5/1 to 1/1. $^{1}$H NMR (400 MHz, $CDCl_3$) δ 8.79 (s, 1H), 5.92 (d, $J$ = 3.7 Hz, 1H), 5.41 (d, $J$ = 2.0 Hz, 1H), 4.63 (d, $J$ = 3.7 Hz, 1H), 4.31 – 4.24 (m, 2H), 4.14 – 4.09 (m, 1H), 4.06 – 4.01 (m, 1H), 2.78 (s, 3H), 1.53 (s, 3H), 1.40 (s, 3H), 1.31 (s, 3H), 1.27 (s, 3H). $^{13}$C NMR (100 MHz, $CDCl_3$) δ 161.91, 160.84, 155.91, 121.34, 112.56, 109.59, 105.22, 83.40, 79.92, 77.33, 72.65, 67.55, 26.96, 26.83, 26.33, 25.30, 17.56. HRMS (ESI) *m/z* calcd. for $C_{17}H_{24}NO_7S$ $[M+H]^+$ 386.1273, found 386.1256.

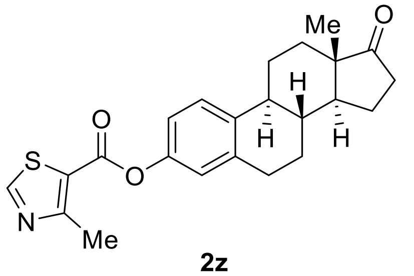


**2z**

**(*8R,9S,13S,14S*)-13-methyl-17-oxo-7,8,9,11,12,13,14,15,16,17-decahydro-6*H*-cyclopenta[a]phenanthren-3-yl 4-methylthiazole-5-carboxylate (2z)**: Prepared according to **General Procedure E** from commercially available estrone. 18% yield for 2 steps, white solid. Flash column chromatography conditions: Petroleum ether/

EtOAc = 10/1 to 1/1. Mp 238-239 °C. $^1$H NMR (400 MHz, $CDCl_3$) δ 8.85 (s, 1H), 7.33 (d, *J* = 8.5 Hz, 1H), 6.99 – 6.92 (m, 2H), 2.93 (dd, *J* = 8.7, 3.9 Hz, 2H), 2.83 (s, 3H), 2.51 (dd, *J* = 18.8, 8.5 Hz, 1H), 2.46 – 2.38 (m, 1H), 2.31 (td, *J* = 10.7, 3.5 Hz, 1H), 2.20 – 1.95 (m, 4H), 1.67 – 1.43 (m, 6H), 0.92 (s, 3H). $^{13}$C NMR (100 MHz, $CDCl_3$) δ 220.77, 162.37, 160.91, 156.18, 148.18, 138.31, 137.97, 126.63, 121.68, 118.85, 50.53, 48.04, 44.28, 38.11, 35.96, 31.66, 29.53, 26.43, 25.89, 21.70, 17.64, 13.95. HRMS (ESI) *m/z* calcd. for $C_{23}H_{26}NO_3S$ $[M+H]^+$ 396.1633, found 396.1611.

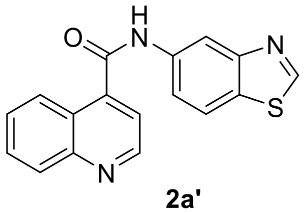


**2a'**

***N*-(benzo[*d*]thiazol-5-yl)quinoline-4-carboxamide (2a')**:

To a solution of quinoline-4-carboxylic acid (2.25 g, 12.98 mmol, 1.3 equiv) and 3-5 drops of anhydrous DMF in anhydrous $CH_2Cl_2$ (30 mL) at 0 ˚C, oxalyl chloride (2.54 g, 19.97 mmol, 2 equiv) was added dropwise over 10 minutes. The reaction was vigorously stirred at room temperature for 3 h. The solvent was removed in vacuum. Anhydrous $CH_2Cl_2$ was added to remove the residual of oxalyl chloride in vacuum. Then the resulting quinoline-4-carbonyl chloride was redissolved in anhydrous acetonitrile and used directly for the next step without further purification.

A solution of the benzo[*d*]thiazol-5-amine (1.5 g, 9.99 mmol, 1 equiv) in anhydrous THF (30 mL) was cooled to 0 ºC, treated with $Et_3N$ (3.03 g, 29.96 mmol, 3 equiv) and stirred for 15 minutes. The quinoline-4-carbonyl chloride in anhydrous acetonitrile was added dropwise over 15 minutes and the mixture was allowed to warm to room for 2 h. The mixture was diluted with saturated $NaHCO_3$ and EtOAc and the layers were separated. The aqueous layer was extracted with EtOAc (2 x) and the combined organic layers were washed saturated $NaHCO_3$ and brine, dried over $Na_2SO_4$, and then evaporated. The residue was crystallized with acetone, afforded **2a'** (2.26g, 74%) as a light pink solid. Mp 204-205 °C. $^1$H NMR (400 MHz, DMSO-$d_6$) δ 11.05 (s, 1H), 9.44 (s, 1H), 9.08 (d, *J* = 4.2 Hz, 1H), 8.75 – 8.67 (m, 1H), 8.25 – 8.13 (m, 3H), 7.88 – 7.78

(m, 3H), 7.71 (t, $J$ = 7.5 Hz, 1H). $^{13}$C NMR (100 MHz, DMSO-$d_6$) δ 165.49, 157.25, 153.56, 150.35, 147.99, 141.83, 137.36, 129.99, 129.50, 128.88, 127.66, 125.27, 123.97, 122.52, 119.30, 118.60, 113.68. HRMS (ESI) $m/z$ calcd. for $C_{17}H_{12}N_3OS$ $[M+H]^+$ 306.0701, found 306.0693.

## General Procedure for remote C(sp$^3$)–H heteroarylation

### Photochemical Reaction Apparatus

Photochemical reaction was carried out under visible light irradiation by two 90 W blue lamps (Kessil A360W E-SERIES TUNA BLUE) at room temperature.

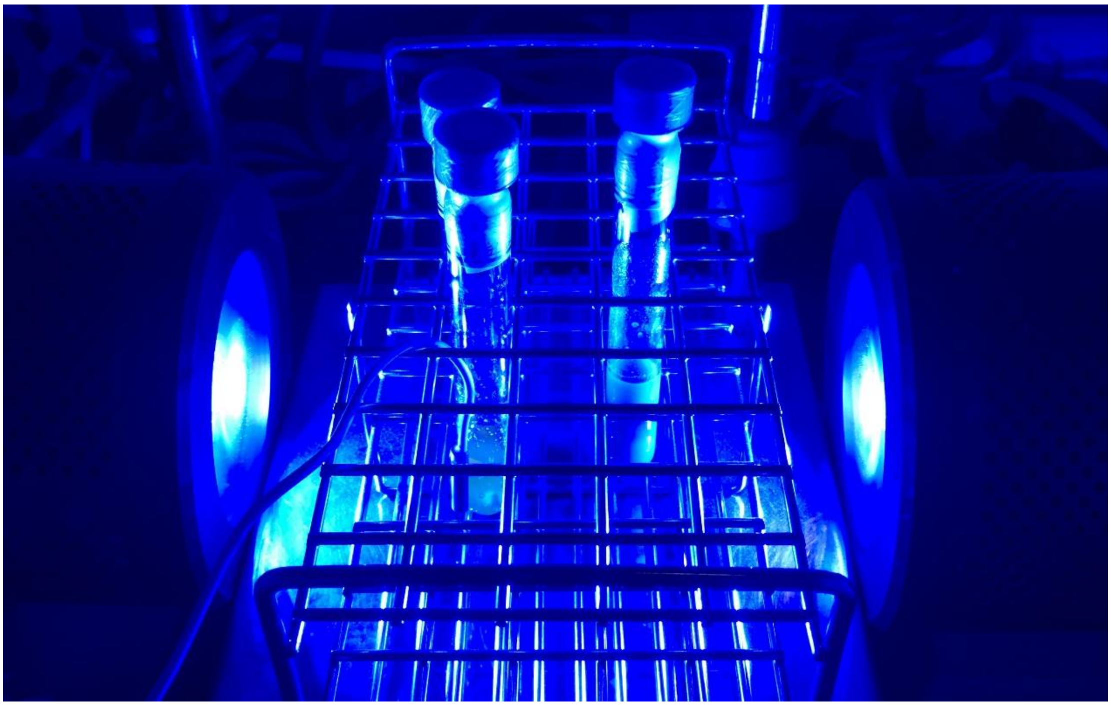

**Supplementary Figure 4.** Reaction set-up for the interrupted HLF reaction

### General Procedure F:

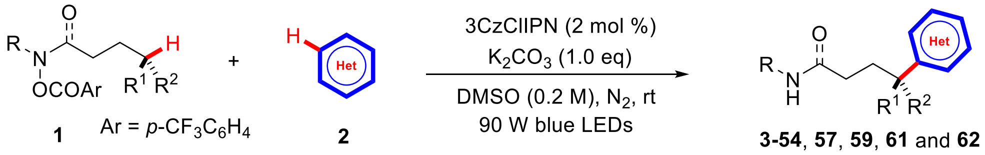


To a mixture of the hydroxamide **1** (1.0 equiv), heteroarene **2** (2.5 equiv), $K_2CO_3$ (1.0 equiv) and 3CzClIPN (2 mol%) in a vial. The vial was evacuated and backfilled with nitrogen for 3-5 times, DMSO (0.2 M) was added with a syringe under nitrogen. The mixture was then irradiated by two 90 W blue lamps. After the reaction was complete

as judged by TLC analysis, the mixture was quenched by adding 20 mL NaCl and 20 mL of EtOAc. The layers were separated and the aqueous layer was extracted with EtOAc (2 × 20 mL). The combined organic layers were washed with saturated $NaHCO_3$ (20 mL) and brine (20 mL), successively, and then evaporated. The crude product was purified by column chromatography on silica gel to afford the desired product **3-54**, **57**, **59**, **61** and **62**.

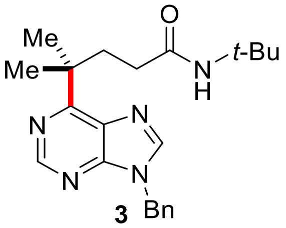

**3**

**4-(9-benzyl-9*H*-purin-6-yl)-*N*-(*tert*-butyl)-4-methylpentanamide (3)**: Prepared according to **General Procedure F** (Reaction time: 46 h). White solid (67.4 mg, 89% yield). Flash column chromatography conditions: EtOAc. Mp 149-150 °C. $^{1}$H NMR (400 MHz, $CDCl_3$) δ 8.90 (s, 1H), 7.97 (s, 1H), 7.39 – 7.28 (m, 5H), 5.85 (s, 1H), 5.42 (s, 2H), 2.43 – 2.37 (m, 2H), 2.00 – 1.94 (m, 2H), 1.54 (s, 6H), 1.29 (s, 9H). $^{13}$C NMR (100 MHz, $CDCl_3$) δ 172.97, 167.87, 151.96, 151.63, 142.60, 135.24, 131.62, 129.24, 128.70, 128.10, 50.84, 47.35, 41.85, 37.91, 34.26, 28.87, 27.57. HRMS (ESI) *m/z* calcd. for $C_{22}H_{30}N_5O$ $[M+H]^+$ 380.2450, found 380.2459.

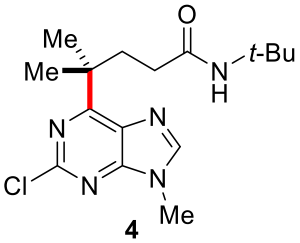

**4**

***N*-(*tert*-butyl)-4-(2-chloro-9-methyl-9*H*-purin-6-yl)-4-methylpentanamide (4)**: Prepared according to **General Procedure F** (Reaction time: 24 h). White solid (64.2 mg, 95% yield). Flash column chromatography conditions: EtOAc. Mp 140-142 °C. $^{1}$H NMR (400 MHz, $CDCl_3$) δ 7.96 (s, 1H), 5.71 (s, 1H), 3.85 (s, 3H), 2.38 – 2.31 (m, 2H), 1.98 – 1.91 (m, 2H), 1.51 (s, 6H), 1.29 (s, 9H). $^{13}$C NMR (100 MHz, $CDCl_3$) δ 172.73, 170.30, 153.70, 153.60, 144.06, 130.82, 50.96, 42.03, 37.79, 34.23, 30.08, 28.87, 27.39. HRMS (ESI) *m/z* calcd. for $C_{16}H_{25}ClN_5O$ $[M+H]^+$ 338.1747, found

338.1736.

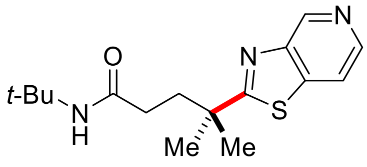


**5**

***N*-(*tert*-butyl)-4-methyl-4-(thiazolo[4,5-*c*]pyridin-2-yl)pentanamide (5)**: Prepared according to **General Procedure F** (Reaction time: 24 h). White solid (56.4 mg, 92% yield). Flash column chromatography conditions: Petroleum ether/ EtOAc = 2/1. Mp 97-98 °C. $^{1}H$ NMR (400 MHz, $CDCl_3$) δ 9.20 (d, *J* = 1.0 Hz, 1H), 8.43 (d, *J* = 5.4 Hz, 1H), 7.76 (dd, *J* = 5.4, 1.0 Hz, 1H), 5.46 (s, 1H), 2.17 – 2.10 (m, 2H), 2.07 – 2.00 (m, 2H), 1.49 (s, 6H), 1.26 (s, 9H). $^{13}C$ NMR (100 MHz, $CDCl_3$) δ 181.78, 171.70, 149.84, 144.73, 143.37, 143.23, 116.53, 51.18, 41.61, 39.23, 33.01, 28.83, 28.65. HRMS (ESI) *m/z* calcd. for $C_{16}H_{24}N_3OS$ $[M+H]^+$ 306.1640, found 306.1644.

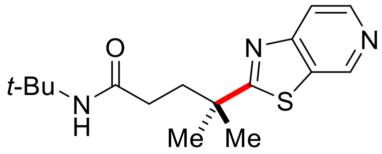


**6**

***N*-(*tert*-butyl)-4-methyl-4-(thiazolo[5,4-*c*]pyridin-2-yl)pentanamide (6)**: Prepared according to **General Procedure F** (Reaction time: 24 h). White solid (57.4 mg, 94% yield). Flash column chromatography conditions: Petroleum ether/ EtOAc = 2/1. Mp 118-120 °C. $^{1}H$ NMR (400 MHz, $CDCl_3$) δ 9.09 (d, *J* = 1.0 Hz, 1H), 8.57 (d, *J* = 5.6 Hz, 1H), 7.81 (dd, *J* = 5.6, 1.0 Hz, 1H), 5.44 (s, 1H), 2.19 – 2.13 (m, 2H), 2.06 – 2.00 (m, 2H), 1.50 (s, 6H), 1.26 (s, 9H). $^{13}C$ NMR (100 MHz, $CDCl_3$) δ 186.35, 171.65, 158.08, 145.50, 144.11, 132.28, 117.18, 51.20, 41.82, 39.26, 33.03, 28.84, 28.67. HRMS (ESI) *m/z* calcd. for $C_{16}H_{24}N_3OS$ $[M+H]^+$ 306.1640, found 306.1653.

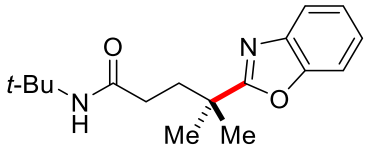


**7**

**4-(benzo[*d*]oxazol-2-yl)-*N*-(*tert*-butyl)-4-methylpentanamide (7)**: Prepared according to **General Procedure F** (Reaction time: 147 h). White solid (32.3 mg, 56%

yield). Flash column chromatography conditions: Petroleum ether/ EtOAc = 3/1.

Mp 135-137 °C. $^{1}$H NMR (400 MHz, $CDCl_3$) δ 7.72 – 7.63 (m, 1H), 7.53 – 7.44 (m, 1H), 7.34 – 7.27 (m, 2H), 5.40 (s, 1H), 2.18 – 2.09 (m, 2H), 2.07 – 1.99 (m, 2H), 1.47 (s, 6H), 1.28 (s, 9H). $^{13}$C NMR (100 MHz, $CDCl_3$) δ 172.17, 171.85, 150.90, 141.11, 124.70, 124.21, 119.76, 110.65, 51.20, 37.43, 37.15, 33.26, 28.85, 26.47. HRMS (ESI) *m/z* calcd. for $C_{17}H_{25}N_2O_2$ $[M+H]^+$ 289.1916, found 289.1928.

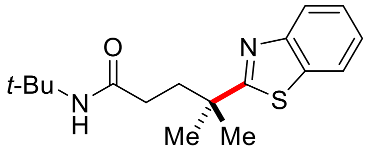


**8**

**4-(benzo[*d*]thiazol-2-yl)-*N*-(*tert*-butyl)-4-methylpentanamide (8)**: Prepared according to **General Procedure F** (Reaction time: 24 h). White solid (54.2 mg, 89% yield). Flash column chromatography conditions: Petroleum ether/ EtOAc = 10/1 to 2/1. Mp 104-106 °C. $^{1}$H NMR (400 MHz, $CDCl_3$) δ 7.97 (d, *J* = 8.1 Hz, 1H), 7.85 (d, *J* = 7.9 Hz, 1H), 7.44 (t, *J* = 7.4 Hz, 1H), 7.34 (t, *J* = 7.5 Hz, 1H), 5.31 (s, 1H), 2.19 – 2.11 (m, 2H), 2.07 – 1.99 (m, 2H), 1.50 (s, 6H), 1.28 (s, 9H). $^{13}$C NMR (100 MHz, $CDCl_3$) δ 180.36, 172.08, 153.18, 135.17, 125.92, 124.81, 122.80, 121.68, 51.15, 41.34, 39.42, 33.33, 28.86, 28.77. HRMS (ESI) *m/z* calcd. for $C_{17}H_{25}N_2OS$ $[M+H]^+$ 305.1687, found 305.1701.

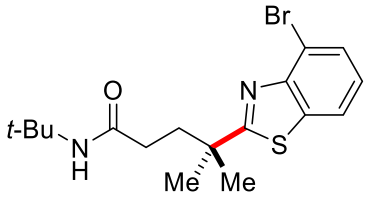


**9**

**4-(4-bromobenzo[*d*]thiazol-2-yl)-*N*-(*tert*-butyl)-4-methylpentanamide (9)**: Prepared according to **General Procedure F** (Reaction time: 25 h). Colorless oil (72.7 mg, 95% yield). Flash column chromatography conditions: Petroleum ether/ EtOAc = 4/1. $^{1}$H NMR (400 MHz, $CDCl_3$) δ 7.77 (dd, *J* = 8.0, 1.0 Hz, 1H), 7.62 (dd, *J* = 7.8, 1.0 Hz, 1H), 7.18 (t, *J* = 7.9 Hz, 1H), 5.33 (s, 1H), 2.19 – 2.13 (m, 2H), 2.13 – 2.07 (m, 2H), 1.50 (s, 6H), 1.29 (s, 9H). $^{13}$C NMR (100 MHz, $CDCl_3$) δ 180.85, 172.24, 151.28, 136.13, 129.33, 125.65, 120.87, 116.50, 51.17, 41.64, 39.31, 33.50, 28.88. HRMS (ESI)

*m/z* calcd. for $C_{17}H_{24}BrN_2OS$ $[M+H]^+$ 383.0792, found 383.0775.

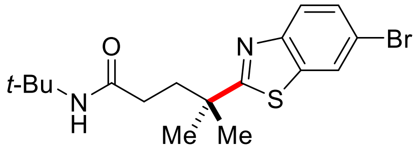

**10**

**4-(6-bromobenzo[*d*]thiazol-2-yl)-*N*-(*tert*-butyl)-4-methylpentanamide (10)**: Prepared according to **General Procedure F** (Reaction time: 30 h). White solid (71.0 mg, 93% yield). Flash column chromatography conditions: Petroleum ether/ EtOAc = 3/1. Mp 89-91 °C. $^1$H NMR (400 MHz, $CDCl_3$) δ 7.96 (d, *J* = 1.8 Hz, 1H), 7.79 (d, *J* = 8.7 Hz, 1H), 7.52 (dd, *J* = 8.7, 1.8 Hz, 1H), 5.31 (s, 1H), 2.17 – 2.07 (m, 2H), 2.06 – 1.96 (m, 2H), 1.47 (s, 6H), 1.27 (s, 9H). $^{13}$C NMR (100 MHz, $CDCl_3$) δ 180.98, 171.83, 152.03, 136.86, 129.37, 124.17, 123.91, 118.30, 51.17, 41.39, 39.28, 33.16, 28.85, 28.63. HRMS (ESI) *m/z* calcd. for $C_{17}H_{24}BrN_2OS$ $[M+H]^+$ 383.0792, found 383.0782.

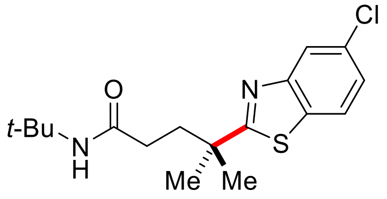

**11**

***N*-(*tert*-butyl)-4-(5-chlorobenzo[*d*]thiazol-2-yl)-4-methylpentanamide (11)**: Prepared according to **General Procedure F** (Reaction time: 30 h). White solid (67.3 mg, 99% yield). Flash column chromatography conditions: Petroleum ether/ EtOAc = 4/1. Mp 106-107 °C. $^1$H NMR (400 MHz, $CDCl_3$) δ 7.94 (d, *J* = 1.9 Hz, 1H), 7.73 (d, *J* = 8.5 Hz, 1H), 7.30 (dd, *J* = 8.5, 2.0 Hz, 1H), 5.31 (s, 1H), 2.17 – 2.07 (m, 2H), 2.07 – 1.98 (m, 2H), 1.48 (s, 6H), 1.28 (s, 9H). $^{13}$C NMR (100 MHz, $CDCl_3$) δ 182.43, 171.85, 154.04, 133.42, 131.89, 125.24, 122.70, 122.36, 51.18, 41.47, 39.28, 33.16, 28.85, 28.65. HRMS (ESI) *m/z* calcd. for $C_{17}H_{24}ClN_2OS$ $[M+H]^+$ 339.1298, found 339.1306.

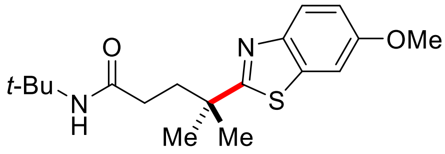

**12**

***N*-(*tert*-butyl)-4-(6-methoxybenzo[*d*]thiazol-2-yl)-4-methylpentanamide (12)**: Prepared according to **General Procedure F** (Reaction time: 24 h). White solid (56.3

mg, 84% yield). Flash column chromatography conditions: Petroleum ether/ EtOAc = 2.5/1. Mp 98-100 °C. $^1$H NMR (400 MHz, $CDCl_3$) δ 7.83 (d, *J* = 8.9 Hz, 1H), 7.29 (d, *J* = 2.5 Hz, 1H), 7.03 (dd, *J* = 8.9, 2.6 Hz, 1H), 5.35 (s, 1H), 3.85 (s, 3H), 2.14 – 2.07 (m, 2H), 2.06 – 1.98 (m, 2H), 1.47 (s, 6H), 1.28 (s, 9H). $^{13}$C NMR (100 MHz, $CDCl_3$) δ 177.71, 172.12, 157.42, 147.58, 136.40, 123.20, 115.07, 104.28, 55.91, 51.11, 41.16, 39.41, 33.30, 28.85, 28.72. HRMS (ESI) *m/z* calcd. for $C_{18}H_{27}N_2O_2S$ [M+H]$^+$ 335.1793, found 335.1812.

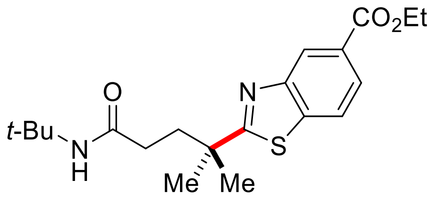


**13**

**Ethyl 2-(5-(*tert*-butylamino)-2-methyl-5-oxopentan-2-yl)benzo[*d*]thiazole-5-carboxylate (13)**: Prepared according to **General Procedure F** (Reaction time: 24 h). White solid (68.7 mg, 91% yield). Flash column chromatography conditions: Petroleum ether/ EtOAc = 3/1. Mp 93-95 °C. $^1$H NMR (400 MHz, $CDCl_3$) δ 8.64 (d, *J* = 1.3 Hz, 1H), 8.00 (dd, *J* = 8.4, 1.5 Hz, 1H), 7.86 (d, *J* = 8.4 Hz, 1H), 5.37 (s, 1H), 4.39 (q, *J* = 7.1 Hz, 2H), 2.18 – 2.09 (m, 2H), 2.08 – 1.99 (m, 2H), 1.49 (s, 6H), 1.39 (t, *J* = 7.1 Hz, 3H), 1.27 (s, 9H). $^{13}$C NMR (100 MHz, $CDCl_3$) δ 181.72, 171.85, 166.49, 152.94, 139.92, 128.56, 125.43, 124.27, 121.47, 61.24, 51.16, 41.48, 39.24, 33.13, 28.83, 28.64, 14.42. HRMS (ESI) *m/z* calcd. for $C_{20}H_{29}N_2O_3S$ [M+H]$^+$ 377.1899, found 377.1899.

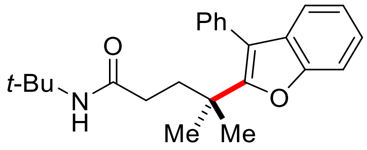


**14**

***N*-(*tert*-butyl)-4-methyl-4-(3-phenylbenzofuran-2-yl)pentanamide (14)**: Prepared according to **General Procedure F** (Reaction time: 49 h). White solid (45.1 mg, 62% yield). Flash column chromatography conditions: Petroleum ether/ EtOAc = 5/1. Mp 162-164 °C. $^1$H NMR (400 MHz, $CDCl_3$) δ 7.47 – 7.43 (m, 2H), 7.43 – 7.39 (m, 2H), 7.38 – 7.35 (m, 2H), 7.28 – 7.24 (m, 1H), 7.15 (d, *J* = 4.1 Hz, 2H), 5.04 (s, 1H), 2.04 – 1.94 (m, 4H), 1.27 (s, 15H). $^{13}$C NMR (100 MHz, $CDCl_3$) δ 172.31, 158.59, 152.86,

133.73, 131.47, 130.74, 128.32, 127.64, 123.87, 122.53, 119.71, 117.10, 110.70, 51.10, 38.41, 37.80, 33.78, 28.85, 28.13. HRMS (ESI) *m/z* calcd. for $C_{24}H_{30}NO_2$ $[M+H]^+$ 364.2276, found 364.2273.

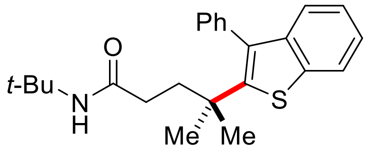


**15**

***N*-(*tert*-butyl)-4-methyl-4-(3-phenylbenzo[*b*]thiophen-2-yl)pentanamide (15)**: Prepared according to **General Procedure F** (Reaction time: 49 h). White solid (26.3 mg, 35% yield). Flash column chromatography conditions: Petroleum ether/ EtOAc = 5/1. Mp 150-153 °C. $^1$H NMR (400 MHz, $CDCl_3$) δ 7.78 (d, *J* = 7.9 Hz, 1H), 7.48 – 7.40 (m, 3H), 7.32 – 7.27 (m, 3H), 7.24 – 7.19 (m, 1H), 7.01 (d, *J* = 7.8 Hz, 1H), 5.10 (s, 1H), 2.07 – 2.02 (m, 2H), 1.90 – 1.85 (m, 2H), 1.30 (s, 6H), 1.28 (s, 9H). $^{13}$C NMR (100 MHz, $CDCl_3$) δ 172.27, 149.14, 143.05, 137.48, 136.64, 133.12, 130.64, 128.34, 127.69, 124.09, 124.06, 122.97, 121.53, 51.13, 40.18, 39.11, 33.81, 30.93, 28.90. HRMS (ESI) *m/z* calcd. for $C_{24}H_{30}NOS$ $[M+H]^+$ 380.2048, found 380.2033.

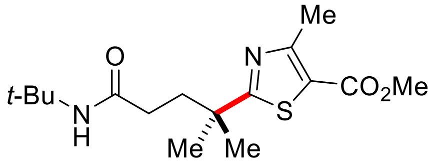


**16**

**Methyl 2-(5-(*tert*-butylamino)-2-methyl-5-oxopentan-2-yl)-4-methylthiazole-5-carboxylate (16)**: Prepared according to **General Procedure F** (Reaction time: 43 h). White solid (57.3 mg, 88% yield). Flash column chromatography conditions: Petroleum ether/ EtOAc = 3/1. Mp 90-92 °C. $^1$H NMR (400 MHz, $CDCl_3$) δ 5.31 (s, 1H), 3.82 (s, 3H), 2.67 (s, 3H), 2.07 – 2.01 (m, 2H), 2.00 – 1.93 (m, 2H), 1.38 (s, 6H), 1.29 (s, 9H). $^{13}$C NMR (100 MHz, $CDCl_3$) δ 182.55, 171.93, 162.86, 160.13, 120.68, 52.12, 51.18, 40.82, 39.10, 33.22, 28.87, 28.54, 17.58. HRMS (ESI) *m/z* calcd. for $C_{16}H_{27}N_2O_3S$ $[M+H]^+$ 327.1742, found 327.1740.

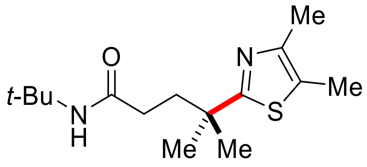


**17**

***N*-(*tert*-butyl)-4-(4,5-dimethylthiazol-2-yl)-4-methylpentanamide (17)**: Prepared according to **General Procedure F** (Reaction time: 25 h). White solid (23.7 mg, 42% yield). Flash column chromatography conditions: Petroleum ether/ EtOAc = 2.5/1. Mp 86-88 °C. $^{1}$H NMR (400 MHz, $CDCl_3$) δ 5.38 (s, 1H), 2.31 – 2.25 (m, 6H), 1.99 (s, 4H), 1.35 (s, 6H), 1.30 (s, 9H). $^{13}$C NMR (100 MHz, $CDCl_3$) δ 174.72, 172.55, 147.23, 124.98, 51.08, 40.20, 39.48, 33.59, 28.90, 14.93, 11.37. HRMS (ESI) *m/z* calcd. for $C_{15}H_{27}N_2OS$ $[M+H]^+$ 283.1844, found 283.1841.

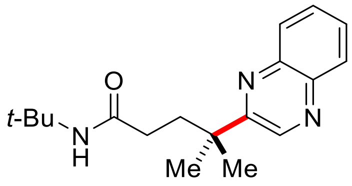


**18**

***N*-(*tert*-butyl)-4-methyl-4-(quinoxalin-2-yl)pentanamide (18)**: Prepared according to **General Procedure F** (Reaction time: 43 h). White solid (47.8 mg, 80% yield). Flash column chromatography conditions: Petroleum ether/ EtOAc = 5/1 to 1/1. Mp 105-108 °C. $^{1}$H NMR (400 MHz, $CDCl_3$) δ 8.93 (s, 1H), 8.11 – 7.96 (m, 2H), 7.78 – 7.62 (m, 2H), 5.22 (s, 1H), 2.24 – 2.15 (m, 2H), 1.97 – 1.88 (m, 2H), 1.48 (s, 6H), 1.25 (s, 9H). $^{13}$C NMR (100 MHz, $CDCl_3$) δ 172.12, 162.19, 143.77, 141.63, 140.85, 129.84, 129.33, 129.19, 129.06, 51.12, 40.07, 38.20, 33.24, 28.83, 27.53. HRMS (ESI) *m/z* calcd. for $C_{18}H_{26}N_3O$ $[M+H]^+$ 300.2076, found 300.2090.

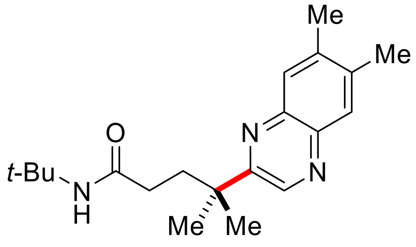


**19**

***N*-(*tert*-butyl)-4-(6,7-dimethylquinoxalin-2-yl)-4-methylpentanamide (19)**: Prepared according to **General Procedure F** (Reaction time: 25 h). White solid (64.4

mg, 98% yield). Flash column chromatography conditions: Petroleum ether/ EtOAc = 3/1. Mp 124-125 °C. $^{1}$H NMR (400 MHz, $CDCl_3$) δ 8.81 (s, 1H), 7.77 (s, 2H), 5.23 (s, 1H), 2.46 (s, 6H), 2.20 – 2.12 (m, 2H), 1.95 – 1.87 (m, 2H), 1.46 (s, 6H), 1.26 (s, 9H). $^{13}$C NMR (100 MHz, $CDCl_3$) δ 172.26, 161.19, 142.73, 140.55, 140.27, 139.78, 139.52, 128.40, 128.08, 51.09, 39.87, 38.37, 33.33, 28.85, 27.59, 20.34. HRMS (ESI) *m/z* calcd. for $C_{20}H_{30}N_3O$ [M+H]$^+$ 328.2389, found 328.2406.

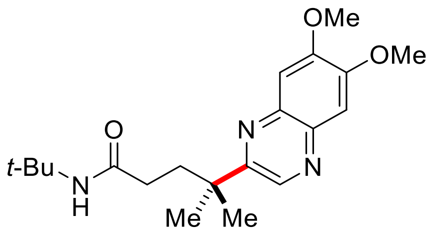


**20**

***N*-(*tert*-butyl)-4-(6,7-dimethoxyquinoxalin-2-yl)-4-methylpentanamide (20)**: Prepared according to **General Procedure F** (Reaction time: 25 h). White solid (64.4 mg, 90% yield). Flash column chromatography conditions: EtOAc. Mp 65-67 °C. $^{1}$H NMR (400 MHz, $CDCl_3$) δ 8.70 (s, 1H), 7.27 (d, *J* = 3.5 Hz, 2H), 5.24 (s, 1H), 4.03 (s, 3H), 4.01 (s, 3H), 2.18 – 2.11 (m, 2H), 1.95 – 1.87 (m, 2H), 1.45 (s, 6H), 1.25 (s, 9H). $^{13}$C NMR (100 MHz, $CDCl_3$) δ 172.26, 159.84, 152.63, 152.16, 140.85, 138.83, 137.79, 106.91, 106.54, 56.40, 56.37, 51.08, 39.63, 38.53, 33.31, 28.85, 27.68. HRMS (ESI) *m/z* calcd. for $C_{20}H_{30}N_3O_3$ [M+H]$^+$ 360.2287, found 360.2297.

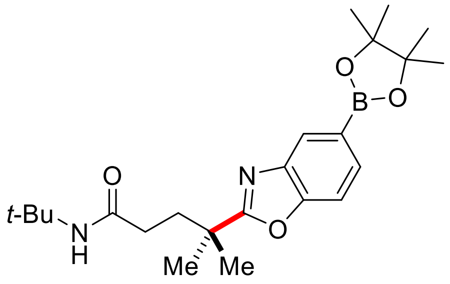


**21**

***N*-(*tert*-butyl)-4-methyl-4-(5-(4,4,5,5-tetramethyl-1,3,2-dioxaborolan-2-yl)benzo[*d*]oxazol-2-yl)pentanamide (21)**: Prepared according to **General Procedure F** (Reaction time: 24 h). Colorless oil (53.8 mg, 65% yield). Flash column chromatography conditions: Petroleum ether/ EtOAc = 2/1. $^{1}$H NMR (400 MHz, $CDCl_3$) δ 8.11 (s, 1H), 7.75 (dd, *J* = 8.1, 1.0 Hz, 1H), 7.46 (d, *J* = 8.1 Hz, 1H), 5.45 (s, 1H),

2.15 – 2.08 (m, 2H), 2.06 – 2.00 (m, 2H), 1.46 (s, 6H), 1.34 (s, 12H), 1.28 (s, 9H). $^{13}$C NMR (100 MHz, $CDCl_3$) δ 172.04, 153.09, 140.90, 131.38, 126.57, 110.10, 84.01, 51.21, 37.44, 37.19, 33.29, 28.85, 26.44, 24.98. HRMS (ESI) *m/z* calcd. for $C_{23}H_{36}BN_2O_4$ $[M+H]^+$ 415.2768, found 415.2753.

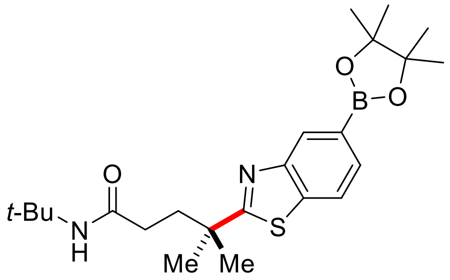


**22**

***N*-(*tert*-butyl)-4-methyl-4-(5-(4,4,5,5-tetramethyl-1,3,2-dioxaborolan-2-yl)benzo[*d*]thiazol-2-yl)pentanamide (22)**: Prepared according to **General Procedure F** (Reaction time: 30 h). White solid (65.0 mg, 76% yield). Flash column chromatography conditions: Petroleum ether/ EtOAc = 3/1. Mp 54-57 °C. $^1$H NMR (400 MHz, $CDCl_3$) δ 8.45 (s, 1H), 7.86 – 7.81 (m, 1H), 7.74 (dd, *J* = 8.0, 1.0 Hz, 1H), 5.33 (s, 1H), 2.16 – 2.10 (m, 2H), 2.04 – 2.00 (m, 2H), 1.48 (s, 6H), 1.35 (s, 12H), 1.28 (s, 9H). $^{13}$C NMR (100 MHz, $CDCl_3$) δ 179.79, 172.15, 152.87, 138.29, 130.36, 129.61, 121.06, 84.03, 51.15, 41.30, 39.31, 33.28, 28.86, 28.75, 25.01. HRMS (ESI) *m/z* calcd. for $C_{23}H_{36}BN_2O_3S$ $[M+H]^+$ 431.2539, found 431.2532.

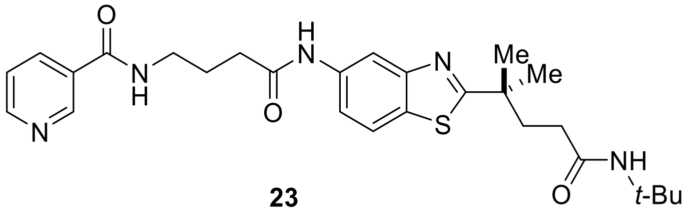


**23**

***N*-(4-((2-(5-(*tert*-butylamino)-2-methyl-5-oxopentan-2-yl)benzo[*d*]thiazol-5-yl)amino)-4-oxobutyl)nicotinamide (23)**: Prepared according to **General Procedure F** (Reaction time: 96 h). White solid (45.4 mg, 89% yield). Flash column chromatography conditions: DCM/ MeOH = 10/1. Mp 95-98 °C. $^1$H NMR (400 MHz, $CDCl_3$) δ 9.43 (s, 1H), 9.05 (s, 1H), 8.59 (d, *J* = 3.9 Hz, 1H), 8.16 (d, *J* = 1.9 Hz, 1H), 8.11 (d, *J* = 7.9 Hz, 1H), 7.94 (t, *J* = 5.8 Hz, 1H), 7.64 (d, *J* = 8.6 Hz, 1H), 7.51 (dd, *J* = 8.6, 2.0 Hz, 1H), 7.28 – 7.26 (m, 1H), 5.86 (s, 1H), 3.56 (q, *J* = 6.0 Hz, 2H), 2.51 (t,

*J* = 6.6 Hz, 2H), 2.15 – 1.95 (m, 6H), 1.43 (s, 6H), 1.27 (s, 9H). $^{13}$C NMR (100 MHz, $CDCl_3$) δ 181.52, 172.36, 172.22, 166.35, 153.56, 151.74, 148.23, 136.69, 135.48, 130.49, 130.10, 123.56, 121.58, 118.32, 114.12, 51.18, 41.31, 39.81, 39.42, 35.15, 33.15, 28.85, 28.70, 25.71. HRMS (ESI) *m/z* calcd. for $C_{27}H_{35}N_5O_3S$ $[M+H]^+$ 510.2539, found 510.2519.

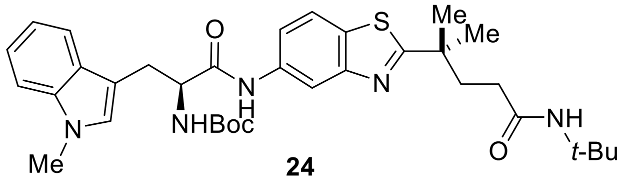


**24**

***Tert*-butyl (*S*)-(1-((2-(5-(*tert*-butylamino)-2-methyl-5-oxopentan-2-yl)benzo[*d*]thiazol-5-yl)amino)-3-(1-methyl-1*H*-indol-3-yl)-1-oxopropan-2-yl)carbamate (24)**: Prepared according to **General Procedure F** (Reaction time: 24 h). White solid (55.7 mg, 90% yield). Flash column chromatography conditions: Toluene/ EtOAc = 2/1. Mp 102-104 °C. $^1$H NMR (400 MHz, $CDCl_3$) δ 8.10 (s, 2H), 7.65 (dd, *J* = 12.4, 8.3 Hz, 2H), 7.29 (d, *J* = 8.2 Hz, 1H), 7.22 (t, *J* = 7.4 Hz, 1H), 7.16 – 7.07 (m, 2H), 6.93 (s, 1H), 5.45 (s, 1H), 5.40 – 5.28 (m, 1H), 4.63 (s, 1H), 3.69 (s, 3H), 3.38 (dd, *J* = 14.1, 4.8 Hz, 1H), 3.26 (dd, *J* = 14.5, 7.1 Hz, 1H), 2.15 – 2.08 (m, 2H), 2.04 – 2.00 (m, 2H), 1.47 (s, 6H), 1.43 (s, 9H), 1.30 (s, 9H). $^{13}$C NMR (100 MHz, $CDCl_3$) δ 181.47, 172.19, 170.40, 153.65, 137.10, 135.79, 130.81, 128.15, 127.94, 122.03, 121.50, 119.49, 119.02, 118.06, 114.32, 109.47, 108.97, 56.07, 51.15, 41.36, 39.31, 33.25, 32.79, 28.87, 28.73, 28.43. HRMS (ESI) *m/z* calcd. for $C_{34}H_{46}N_5O_4S$ $[M+H]^+$ 620.3270, found 620.3251.

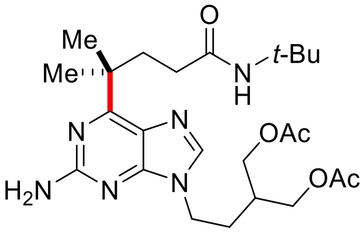


**25**

**2-(2-(2-amino-6-(5-(*tert*-butylamino)-2-methyl-5-oxopentan-2-yl)-9*H*-purin-9-yl)ethyl)propane-1,3-diyl diacetate (25)**: Prepared according to **General Procedure F** (Reaction time: 25 h). Colorless oil (50.0 mg, 51% yield). Flash column

chromatography conditions: EtOAc. $^{1}$H NMR (400 MHz, $CDCl_3$) δ 7.67 (s, 1H), 5.91 (s, 1H), 4.93 (s, 2H), 4.17 (t, *J* = 7.1 Hz, 2H), 4.11 (d, *J* = 5.5 Hz, 4H), 2.37 – 2.30 (m, 2H), 2.04 (s, 6H), 2.03 – 1.90 (m, 5H), 1.44 (s, 6H), 1.30 (s, 9H). $^{13}$C NMR (100 MHz, $CDCl_3$) δ 173.22, 171.01, 169.01, 159.21, 153.43, 139.65, 125.68, 63.80, 50.83, 41.64, 40.98, 37.88, 35.09, 34.37, 28.90, 28.84, 27.52, 20.97. HRMS (ESI) *m/z* calcd. for $C_{24}H_{39}N_6O_5$ $[M+H]^+$ 491.2982, found 491.2969.

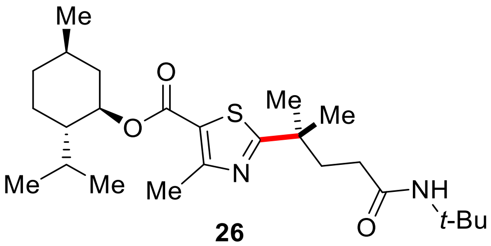


**26**

**(*1R,2S,5R*)-2-isopropyl-5-methylcyclohexyl 2-(5-(*tert*-butylamino)-2-methyl-5-oxopentan-2-yl)-4-methylthiazole-5-carboxylate (26)**: Prepared according to **General Procedure F** (Reaction time: 24 h). Colorless oil (43.9 mg, 97% yield). Flash column chromatography conditions: Petroleum ether/ EtOAc = 3/1. $^{1}$H NMR (400 MHz, $CDCl_3$) δ 5.29 (s, 1H), 4.83 (td, *J* = 10.9, 4.4 Hz, 1H), 2.69 (s, 3H), 2.11 – 1.88 (m, 6H), 1.76 – 1.65 (m, 2H), 1.55 – 1.43 (m, 2H), 1.40 (s, 6H), 1.31 (s, 9H), 1.06 (q, *J* = 11.9 Hz, 2H), 0.91 (d, *J* = 6.7 Hz, 7H), 0.78 (d, *J* = 6.9 Hz, 3H). $^{13}$C NMR (100 MHz, $CDCl_3$) δ 182.27, 172.05, 162.14, 159.65, 121.60, 75.34, 51.21, 47.22, 41.18, 40.82, 39.12, 34.34, 33.33, 31.57, 28.90, 28.65, 28.58, 26.53, 23.62, 22.14, 20.94, 17.66, 16.56. HRMS (ESI) *m/z* calcd. for $C_{25}H_{43}N_2O_3S$ $[M+H]^+$ 451.2994, found 451.2991.

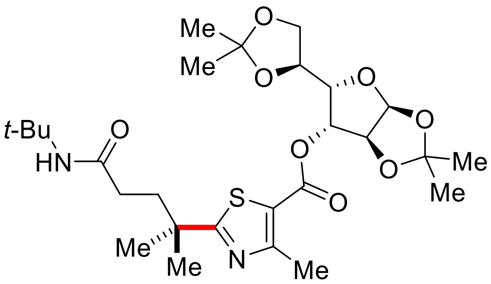


**27**

**(*3aS,5S,6R,6aS*)-5-((*S*)-2,2-dimethyl-1,3-dioxolan-4-yl)-2,2-dimethyltetrahydro-furo[2,3-*d*][1,3]dioxol-6-yl 2-(5-(*tert*-butylamino)-2-methyl-5-oxopentan-2-yl)-4-methylthiazole-5-carboxylate (27)**: Prepared according to **General Procedure F**

(Reaction time: 24 h). White solid (54.9 mg, 99% yield). Flash column chromatography conditions: DCM/ Acetone = 20/1. Mp 60-63 °C. $^1$H NMR (400 MHz, $CDCl_3$) δ 5.90 (d, *J* = 3.7 Hz, 1H), 5.38 (d, *J* = 1.9 Hz, 1H), 5.26 (s, 1H), 4.59 (d, *J* = 3.7 Hz, 1H), 4.31 – 4.23 (m, 2H), 4.16 – 4.09 (m, 1H), 4.08 – 4.01 (m, 1H), 2.70 (s, 3H), 2.08 – 2.02 (m, 2H), 2.01 – 1.95 (m, 2H), 1.53 (s, 3H), 1.40 (d, *J* = 2.5 Hz, 9H), 1.29 (d, *J* = 6.5 Hz, 15H). $^{13}$C NMR (100 MHz, $CDCl_3$) δ 183.33, 171.83, 161.25, 161.03, 120.02, 112.51, 109.53, 105.17, 83.43, 79.83, 76.97, 72.66, 67.42, 51.24, 40.94, 39.04, 33.19, 28.90, 28.58, 28.44, 26.99, 26.83, 26.32, 25.40, 17.74. HRMS (ESI) *m/z* calcd. for $C_{27}H_{43}N_2O_8S$ [M+H]$^+$ 555.2740, found 555.2722.

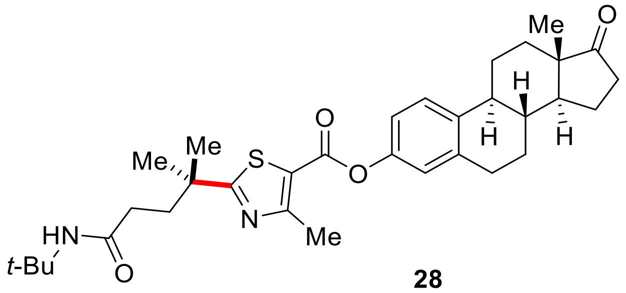


**28**

**(*8R,9S,13S,14S*)-13-methyl-17-oxo-7,8,9,11,12,13,14,15,16,17-decahydro-6*H*-cyclopenta[a]phenanthren-3-yl 2-(5-(*tert*-butylamino)-2-methyl-5-oxopentan-2-yl)-4-methylthiazole-5-carboxylate (28)**: Prepared according to **General Procedure F** (Reaction time: 24 h). White solid (34.6 mg, 61% yield). Flash column chromatography conditions: DCM/ Acetone = 20/1. Mp 87-90 °C. $^1$H NMR (400 MHz, $CDCl_3$) δ 7.31 (d, *J* = 8.5 Hz, 1H), 6.94 (dd, *J* = 8.4, 2.5 Hz, 1H), 6.90 (d, *J* = 2.3 Hz, 1H), 5.30 (s, 1H), 2.96 – 2.88 (m, 2H), 2.75 (s, 3H), 2.51 (dd, *J* = 18.8, 8.5 Hz, 1H), 2.45 – 2.38 (m, 1H), 2.35 – 2.25 (m, 1H), 2.20 – 1.94 (m, 8H), 1.70 – 1.46 (m, 6H), 1.44 (s, 6H), 1.32 (s, 9H), 0.91 (s, 3H). $^{13}$C NMR (100 MHz, $CDCl_3$) δ 220.85, 183.54, 171.90, 161.73, 161.15, 148.29, 138.25, 137.82, 126.60, 121.74, 118.91, 51.25, 50.55, 48.07, 44.29, 41.00, 39.13, 38.13, 35.98, 33.27, 31.68, 29.54, 28.91, 28.58, 26.45, 25.90, 21.72, 17.82, 13.96. HRMS (ESI) *m/z* calcd. for $C_{33}H_{45}N_2O_4S$ [M+H]$^+$ 565.3100, found 565.3076.

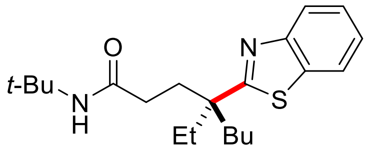

**29**

**4-(benzo[*d*]thiazol-2-yl)-*N*-(*tert*-butyl)-4-ethyloctanamide (29)**: Prepared according to **General Procedure F** (Reaction time: 24 h). Colorless oil (37.5 mg, 52% yield). Flash column chromatography conditions: Petroleum ether/ EtOAc = 5/1. $^{1}$H NMR (400 MHz, $CDCl_3$) δ 7.99 (d, *J* = 7.9 Hz, 1H), 7.89 – 7.83 (m, 1H), 7.48 – 7.42 (m, 1H), 7.38 – 7.32 (m, 1H), 5.28 (s, 1H), 2.19 – 2.11 (m, 2H), 1.99 – 1.76 (m, 6H), 1.29 (s, 13H), 0.93 – 0.86 (m, 3H), 0.86 – 0.79 (m, 3H). $^{13}$C NMR (100 MHz, $CDCl_3$) δ 180.28, 172.25, 152.98, 135.06, 125.80, 124.77, 122.87, 121.67, 51.19, 47.70, 37.03, 34.38, 32.51, 30.14, 28.91, 25.75, 23.38, 14.16, 8.16. HRMS (ESI) *m/z* calcd. for $C_{21}H_{33}N_2OS$ $[M+H]^+$ 361.2313, found 361.2318.

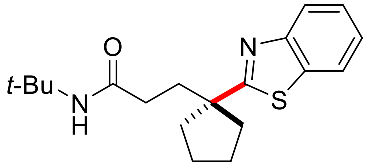

**30**

**3-(1-(benzo[*d*]thiazol-2-yl)cyclopentyl)-*N*-(*tert*-butyl)propanamide (30)**: Prepared according to **General Procedure F** (Reaction time: 24 h). White solid (48.2 mg, 73% yield). Flash column chromatography conditions: Petroleum ether/ EtOAc = 3/1. Mp 114-116 °C. $^{1}$H NMR (400 MHz, $CDCl_3$) δ 7.96 (d, *J* = 8.1 Hz, 1H), 7.84 (d, *J* = 7.9 Hz, 1H), 7.47 – 7.40 (m, 1H), 7.37 – 7.30 (m, 1H), 5.31 (s, 1H), 2.39 – 2.27 (m, 2H), 2.23 – 2.13 (m, 2H), 2.02 – 1.94 (m, 2H), 1.92 – 1.82 (m, 2H), 1.82 – 1.66 (m, 4H), 1.26 (s, 9H). $^{13}$C NMR (100 MHz, $CDCl_3$) δ 180.05, 172.05, 153.15, 135.35, 125.87, 124.78, 122.74, 121.66, 53.42, 51.10, 39.37, 37.72, 34.07, 28.82, 24.36. HRMS (ESI) *m/z* calcd. for $C_{19}H_{27}N_2OS$ $[M+H]^+$ 331.1844, found 331.1829.

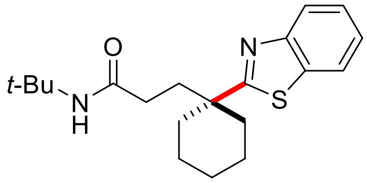

**31**

**3-(1-(benzo[*d*]thiazol-2-yl)cyclohexyl)-*N*-(*tert*-butyl)propanamide (31)**: Prepared

according to **General Procedure F** (Reaction time: 24 h). White solid (50.2 mg, 73% yield). Flash column chromatography conditions: Petroleum ether/ EtOAc = 4/1. Mp 147-149 °C. $^1$H NMR (400 MHz, $CDCl_3$) δ 7.99 (d, *J* = 8.1 Hz, 1H), 7.86 (d, *J* = 7.9 Hz, 1H), 7.48 – 7.40 (m, 1H), 7.38 – 7.30 (m, 1H), 5.19 (s, 1H), 2.32 – 2.21 (m, 2H), 2.09 – 2.02 (m, 2H), 1.97 – 1.87 (m, 2H), 1.74 – 1.59 (m, 4H), 1.55 – 1.34 (m, 4H), 1.23 (s, 9H). $^{13}$C NMR (100 MHz, $CDCl_3$) δ 179.39, 172.13, 153.12, 135.27, 125.74, 124.75, 122.81, 121.71, 51.08, 45.05, 38.85, 37.11, 32.11, 28.78, 25.94, 22.52. HRMS (ESI) *m/z* calcd. for $C_{20}H_{29}N_2OS$ $[M+H]^+$ 345.2000, found 345.1999.

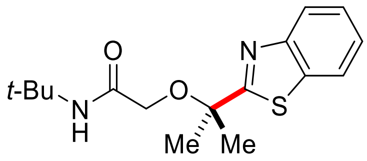


**32**

**2-((2-(benzo[*d*]thiazol-2-yl)propan-2-yl)oxy)-*N*-(*tert*-butyl)acetamide (32)**: Prepared according to **General Procedure F** (Reaction time: 24 h). Colorless oil (53.0 mg, 86% yield). Flash column chromatography conditions: Petroleum ether/ EtOAc = 3/1. $^1$H NMR (400 MHz, $CDCl_3$) δ 7.99 (d, *J* = 8.1 Hz, 1H), 7.88 (d, *J* = 8.0 Hz, 1H), 7.51 – 7.43 (m, 1H), 7.42 – 7.34 (m, 1H), 6.53 (s, 1H), 3.87 (s, 2H), 1.75 (s, 6H), 1.40 (s, 9H). $^{13}$C NMR (100 MHz, $CDCl_3$) δ 176.60, 168.52, 153.13, 135.23, 126.22, 125.42, 123.35, 121.84, 79.18, 64.01, 51.02, 28.92, 27.20. HRMS (ESI) *m/z* calcd. for $C_{16}H_{23}N_2O_2S$ $[M+H]^+$ 307.1480, found 307.1475.

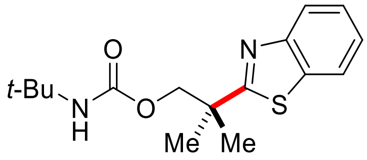


**33**

**2-(benzo[*d*]thiazol-2-yl)-2-methylpropyl *tert*-butylcarbamate (33)**: Prepared according to **General Procedure F** (Reaction time: 24 h). White solid (26.7 mg, 44% yield). Flash column chromatography conditions: Petroleum ether/ EtOAc = 10/1. Mp 79-81 °C. $^1$H NMR (400 MHz, $CDCl_3$) δ 8.00 (d, *J* = 8.1 Hz, 1H), 7.89 – 7.83 (m, 1H), 7.48 – 7.42 (m, 1H), 7.38 – 7.32 (m, 1H), 4.62 (s, 1H), 4.29 (s, 2H), 1.53 (s, 6H), 1.26

(s, 9H). $^{13}$C NMR (100 MHz, $CDCl_3$) δ 177.92, 153.19, 135.15, 125.93, 124.84, 123.00, 121.58, 71.66, 50.44, 42.17, 28.97, 25.80. HRMS (ESI) *m/z* calcd. for $C_{16}H_{23}N_2O_2S$ $[M+H]^+$ 307.1480, found 307.1477.

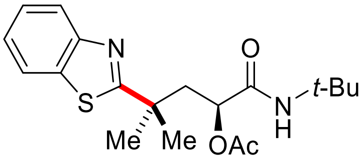


**34**

**(*S*)-4-(benzo[*d*]thiazol-2-yl)-1-(*tert*-butylamino)-4-methyl-1-oxopentan-2-yl acetate (34)**: Prepared according to **General Procedure F** (Reaction time: 24 h). Colorless oil (70.8 mg, 98% yield). Flash column chromatography conditions: Petroleum ether/ EtOAc = 3/1. $^1$H NMR (400 MHz, $CDCl_3$) δ 7.94 (d, *J* = 8.1 Hz, 1H), 7.89 – 7.82 (m, 1H), 7.49 – 7.41 (m, 1H), 7.39 – 7.32 (m, 1H), 6.25 (s, 1H), 5.07 (dd, *J* = 8.7, 2.1 Hz, 1H), 2.58 (dd, *J* = 15.1, 2.1 Hz, 1H), 2.27 (dd, *J* = 15.1, 8.7 Hz, 1H), 1.80 (s, 3H), 1.51 (s, 6H), 1.36 (s, 9H). $^{13}$C NMR (100 MHz, $CDCl_3$) δ 180.62, 170.05, 169.34, 152.83, 134.98, 126.13, 125.04, 122.60, 121.72, 71.85, 51.38, 44.51, 41.05, 30.26, 28.87, 28.83, 20.67. HRMS (ESI) *m/z* calcd. for $C_{19}H_{27}N_2O_3S$ $[M+H]^+$ 363.1742, found 363.1745.

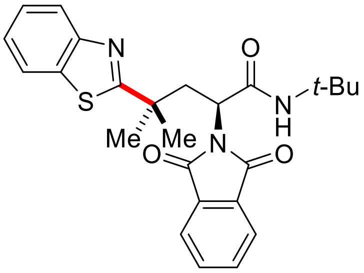


**35**

**(*S*)-4-(benzo[*d*]thiazol-2-yl)-*N*-(*tert*-butyl)-2-(1,3-dioxoisoindolin-2-yl)-4-methylpentanamide (35)**: Prepared according to **General Procedure F** (Reaction time: 24 h). White solid (33.4 mg, 74% yield). Flash column chromatography conditions: Petroleum ether/ EtOAc = 5/1 to 3/1. Mp 107-108 °C. $^1$H NMR (400 MHz, $CDCl_3$) δ 7.79 (d, *J* = 8.0 Hz, 1H), 7.77 – 7.73 (m, 1H), 7.71 – 7.65 (m, 2H), 7.61 – 7.55 (m, 2H), 7.41 – 7.34 (m, 1H), 7.31 – 7.26 (m, 1H), 7.04 (s, 1H), 4.85 (dd, *J* = 9.3, 1.6 Hz, 1H), 3.10 (dd, *J* = 15.4, 9.3 Hz, 1H), 2.85 (dd, *J* = 15.4, 1.4 Hz, 1H), 1.52 (s, 3H), 1.46 (s, 3H), 1.40 (s, 9H). $^{13}$C NMR (100 MHz, $CDCl_3$) δ 180.67, 168.43, 168.39, 152.69,

135.00, 133.98, 131.74, 126.06, 124.93, 123.36, 122.38, 121.70, 52.68, 51.73, 41.85, 41.08, 30.65, 29.01. HRMS (ESI) *m/z* calcd. for $C_{25}H_{28}N_3O_3S$ $[M+H]^+$ 450.1851, found 450.1851.

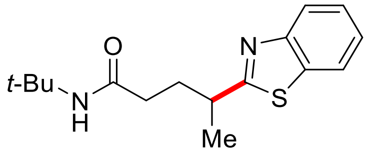


**36**

**4-(benzo[*d*]thiazol-2-yl)-*N*-(*tert*-butyl)pentanamide (36)**: Prepared according to **General Procedure F** (Reaction time: 24 h). White solid (43.5 mg, 75% yield). Flash column chromatography conditions: Petroleum ether/ EtOAc = 2/1. Mp 114-116 °C. $^1$H NMR (400 MHz, $CDCl_3$) δ 7.95 (d, *J* = 8.0 Hz, 1H), 7.85 (d, *J* = 7.8 Hz, 1H), 7.45 (t, *J* = 7.5 Hz, 1H), 7.35 (t, *J* = 7.4 Hz, 1H), 5.68 (s, 1H), 3.41 – 3.26 (m, 1H), 2.20 – 1.99 (m, 4H), 1.47 (d, *J* = 6.8 Hz, 3H), 1.32 (s, 9H). $^{13}$C NMR (100 MHz, $CDCl_3$) δ 177.11, 171.76, 152.99, 134.74, 126.04, 124.90, 122.64, 121.80, 51.24, 38.75, 35.25, 33.59, 28.89, 21.58. HRMS (ESI) *m/z* calcd. for $C_{16}H_{23}N_2OS$ $[M+H]^+$ 291.1531, found 291.1527.

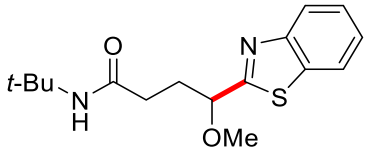


**37**

**4-(benzo[*d*]thiazol-2-yl)-*N*-(*tert*-butyl)-4-methoxybutanamide (37)**: Prepared according to **General Procedure F** (Reaction time: 27 h). White solid (53.0 mg, 86% yield). Flash column chromatography conditions: Petroleum ether/ EtOAc = 1/1. Mp 103-104 °C. $^1$H NMR (400 MHz, $CDCl_3$) δ 7.98 (d, *J* = 8.0 Hz, 1H), 7.89 (d, *J* = 7.6 Hz, 1H), 7.50 – 7.43 (m, 1H), 7.42 – 7.34 (m, 1H), 5.62 (s, 1H), 4.64 (t, *J* = 6.1 Hz, 1H), 3.45 (s, 3H), 2.35 – 2.17 (m, 4H), 1.32 (s, 9H). $^{13}$C NMR (100 MHz, $CDCl_3$) δ 174.41, 171.35, 153.14, 135.00, 126.11, 125.27, 123.09, 122.04, 81.08, 58.14, 51.23, 32.99, 32.21, 28.89. HRMS (ESI) *m/z* calcd. for $C_{16}H_{23}N_2O_2S$ $[M+H]^+$ 307.1480, found 307.1472.

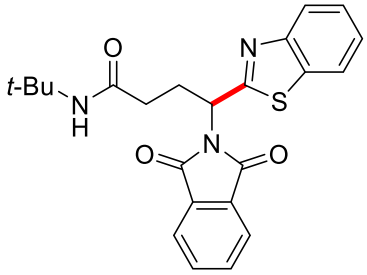

**38**

**4-(benzo[*d*]thiazol-2-yl)-*N*-(*tert*-butyl)-4-(1,3-dioxoisoindolin-2-yl)butanamide (38)**: Prepared according to **General Procedure F** (Reaction time: 142 h). White solid (54.0 mg, 64% yield). Flash column chromatography conditions: Petroleum ether/ EtOAc = 1.5/1. Mp 66-68 °C. $^{1}$H NMR (400 MHz, $CDCl_3$) δ 7.97 (d, *J* = 8.0 Hz, 1H), 7.90 – 7.84 (m, 2H), 7.83 – 7.79 (m, 1H), 7.78 – 7.72 (m, 2H), 7.47 – 7.40 (m, 1H), 7.38 – 7.32 (m, 1H), 5.79 (dd, *J* = 9.4, 6.1 Hz, 1H), 5.48 (s, 1H), 3.01 – 2.81 (m, 2H), 2.29 (t, *J* = 7.2 Hz, 2H), 1.30 (s, 9H). $^{13}$C NMR (100 MHz, $CDCl_3$) δ 170.46, 169.00, 167.72, 152.74, 135.43, 134.46, 131.86, 126.22, 125.46, 123.80, 123.47, 121.74, 52.80, 51.47, 34.44, 28.84, 27.61. HRMS (ESI) *m/z* calcd. for $C_{23}H_{24}N_3O_3S$ $[M+H]^+$ 422.1538, found 422.1540.

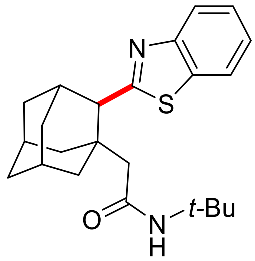

**39**

**2-((*1R,2R,3S,5R,7S*)-2-(benzo[*d*]thiazol-2-yl)adamantan-1-yl)-*N*-(*tert*-butyl)acetamide (39)**: Prepared according to **General Procedure F** (Reaction time: 24 h). White solid (51.3 mg, 67% yield). Flash column chromatography conditions: Petroleum ether/ EtOAc = 7/1. Mp 149-151 °C. $^{1}$H NMR (400 MHz, $CDCl_3$) δ 7.96 (d, *J* = 8.1 Hz, 1H), 7.88 (d, *J* = 8.0 Hz, 1H), 7.50 – 7.44 (m, 1H), 7.41 – 7.35 (m, 1H), 6.65 (s, 1H), 3.67 (s, 1H), 2.39 – 2.32 (m, 2H), 2.31 – 2.22 (m, 2H), 2.12 – 2.05 (m, 2H), 2.03 – 1.96 (m, 1H), 1.95 – 1.90 (m, 2H), 1.86 – 1.81 (m, 1H), 1.79 – 1.74 (m, 3H), 1.71 – 1.63 (m, 1H), 1.50 – 1.44 (m, 1H), 1.29 (s, 9H). $^{13}$C NMR (100 MHz, $CDCl_3$) δ 174.41, 170.26, 152.13, 134.84, 126.09, 125.09, 122.53, 121.52, 53.06, 51.05, 49.13,

43.66, 39.11, 38.80, 37.13, 36.35, 34.54, 32.17, 28.92, 28.47, 28.23. HRMS (ESI) *m/z* calcd. for $C_{23}H_{31}N_2OS$ $[M+H]^+$ 383.2157, found 383.2142.

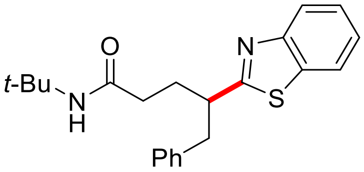

**40**

**4-(benzo[*d*]thiazol-2-yl)-*N*-(*tert*-butyl)-5-phenylpentanamide (40)**: Prepared according to **General Procedure F** (Reaction time: 24 h). Colorless oil (38.1 mg, 52% yield). Flash column chromatography conditions: Petroleum ether/ EtOAc = 2/1. $^1H$ NMR (400 MHz, $CDCl_3$) δ 7.97 (d, *J* = 8.1 Hz, 1H), 7.86 – 7.80 (m, 1H), 7.49 – 7.42 (m, 1H), 7.38 – 7.33 (m, 1H), 7.25 – 7.19 (m, 2H), 7.19 – 7.12 (m, 3H), 5.44 (s, 1H), 3.60 – 3.46 (m, 1H), 3.21 (dd, *J* = 13.8, 7.7 Hz, 1H), 3.07 (dd, *J* = 13.8, 7.2 Hz, 1H), 2.30 – 2.20 (m, 1H), 2.14 – 1.98 (m, 3H), 1.30 (s, 9H). $^{13}C$ NMR (100 MHz, $CDCl_3$) δ 175.17, 171.50, 153.04, 138.90, 134.82, 129.12, 128.58, 126.56, 126.06, 124.97, 122.78, 121.83, 51.26, 46.22, 42.64, 35.19, 31.55, 28.89. HRMS (ESI) *m/z* calcd. for $C_{22}H_{27}N_2OS$ $[M+H]^+$ 367.1844, found 367.1836.

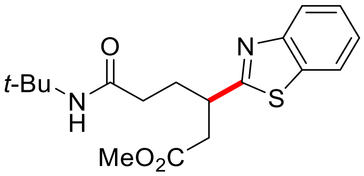

**41**

**Methyl-3-(benzo[*d*]thiazol-2-yl)-6-(*tert*-butylamino)-6-oxohexanoate (41)**: Prepared according to **General Procedure F** (Reaction time: 24 h). Colorless oil (42.5 mg, 61% yield). Flash column chromatography conditions: Petroleum ether/ EtOAc = 1/1. $^1H$ NMR (400 MHz, $CDCl_3$) δ 7.96 (d, *J* = 8.1 Hz, 1H), 7.84 (d, *J* = 7.7 Hz, 1H), 7.48 – 7.42 (m, 1H), 7.38 – 7.32 (m, 1H), 5.52 (s, 1H), 3.77 – 3.70 (m, 1H), 3.63 (s, 3H), 2.99 (dd, *J* = 16.3, 8.2 Hz, 1H), 2.78 (dd, *J* = 16.4, 6.4 Hz, 1H), 2.23 – 2.04 (m, 4H), 1.30 (s, 9H). $^{13}C$ NMR (100 MHz, $CDCl_3$) δ 173.55, 171.77, 171.20, 153.07, 134.83, 126.13, 125.10, 122.90, 121.77, 51.98, 51.31, 40.19, 39.80, 34.72, 31.47, 28.85. HRMS (ESI) *m/z* calcd. for $C_{18}H_{25}N_2O_3S$ $[M+H]^+$ 349.1586, found 349.1576.

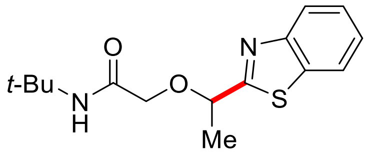

**42**

**2-(1-(benzo[*d*]thiazol-2-yl)ethoxy)-*N*-(*tert*-butyl)acetamide (42)**: Prepared according to **General Procedure F** (Reaction time: 24 h). Colorless oil (48.0 mg, 82% yield). Flash column chromatography conditions: Petroleum ether/ EtOAc = 1.5/1. $^{1}$H NMR (400 MHz, $CDCl_3$) δ 7.99 (d, *J* = 8.1 Hz, 1H), 7.90 (d, *J* = 7.9 Hz, 1H), 7.52 – 7.45 (m, 1H), 7.43 – 7.36 (m, 1H), 6.50 (s, 1H), 4.91 (q, *J* = 6.5 Hz, 1H), 4.07 – 3.92 (m, 2H), 1.71 (d, *J* = 6.5 Hz, 3H), 1.38 (s, 9H). $^{13}$C NMR (100 MHz, $CDCl_3$) δ 173.27, 168.01, 153.11, 134.74, 126.38, 125.52, 123.34, 121.97, 77.04, 69.51, 51.13, 28.89, 21.87. HRMS (ESI) *m/z* calcd. for $C_{15}H_{21}N_2O_2S$ $[M+H]^+$ 293.1323, found 293.1319.

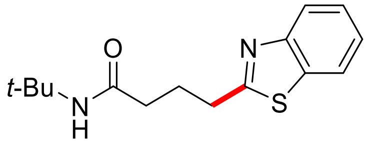

**43**

**4-(benzo[*d*]thiazol-2-yl)-*N*-(*tert*-butyl)butanamide (43)**: Prepared according to **General Procedure F** (Reaction time: 72 h). White solid (15.8 mg, 29% yield). Flash column chromatography conditions: Petroleum ether/ EtOAc = 1.5/1. Mp 83-85 °C. $^{1}$H NMR (400 MHz, $CDCl_3$) δ 7.95 (d, *J* = 8.1 Hz, 1H), 7.87 – 7.82 (m, 1H), 7.48 – 7.42 (m, 1H), 7.39 – 7.32 (m, 1H), 5.67 (s, 1H), 3.20 – 3.14 (m, 2H), 2.23 – 2.16 (m, 4H), 1.35 (s, 9H). $^{13}$C NMR (100 MHz, $CDCl_3$) δ 171.54, 171.35, 153.31, 135.29, 126.09, 124.95, 122.61, 121.72, 51.36, 36.24, 33.13, 28.95, 25.58. HRMS (ESI) *m/z* calcd. for $C_{15}H_{21}N_2OS$ $[M+H]^+$ 277.1374, found 277.1368.

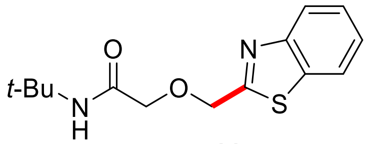

**44**

**2-(benzo[*d*]thiazol-2-ylmethoxy)-*N*-(*tert*-butyl)acetamide (44)**: Prepared according to **General Procedure F** (Reaction time: 24 h). Colorless oil (33.4 mg, 60% yield). Flash column chromatography conditions: Petroleum ether/ EtOAc = 1.5/1. $^{1}$H NMR

(400 MHz, $CDCl_3$) δ 8.01 (d, *J* = 8.1 Hz, 1H), 7.91 (d, *J* = 7.9 Hz, 1H), 7.54 – 7.46 (m, 1H), 7.45 – 7.38 (m, 1H), 6.47 (s, 1H), 4.96 (s, 2H), 4.05 (s, 2H), 1.38 (s, 9H). $^{13}C$ NMR (100 MHz, $CDCl_3$) δ 167.72, 153.12, 134.99, 126.48, 125.62, 123.38, 121.93, 71.11, 70.66, 51.22, 28.90. HRMS (ESI) *m/z* calcd. for $C_{14}H_{19}N_2O_2S$ $[M+H]^+$ 279.1167, found 279.1158.

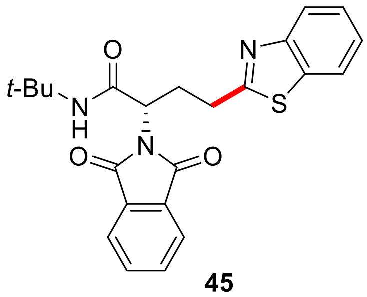


**45**

**(*S*)-4-(benzo[*d*]thiazol-2-yl)-*N*-(*tert*-butyl)-2-(1,3-dioxoisoindolin-2-yl)butanamide (45)**: Prepared according to **General Procedure F** (Reaction time: 67 h). Colorless oil (34.6 mg, 41% yield). Flash column chromatography conditions: Petroleum ether/ EtOAc = 2/1. $^1H$ NMR (400 MHz, $CDCl_3$) δ 7.85 (d, *J* = 8.1 Hz, 1H), 7.81 (dd, *J* = 5.5, 3.1 Hz, 2H), 7.80 – 7.76 (m, 1H), 7.72 – 7.66 (m, 2H), 7.44 – 7.38 (m, 1H), 7.35 – 7.30 (m, 1H), 6.35 (s, 1H), 4.86 (dd, *J* = 9.1, 6.2 Hz, 1H), 3.19 (t, *J* = 7.2 Hz, 2H), 2.91 – 2.82 (m, 2H), 1.36 (s, 9H). $^{13}C$ NMR (100 MHz, $CDCl_3$) δ 170.00, 168.34, 167.53, 153.09, 134.33, 131.78, 126.13, 125.04, 123.67, 122.59, 121.66, 54.93, 51.93, 31.35, 28.83, 28.41. HRMS (ESI) *m/z* calcd. for $C_{23}H_{24}N_3O_3S$ $[M+H]^+$ 422.1538, found 422.1535.

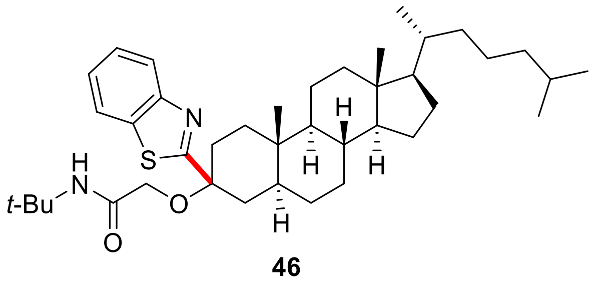


**46**

**2-(((*5S,8R,9S,10S,13R,14S,17R*)-3-(benzo[*d*]thiazol-2-yl)-10,13-dimethyl-17-((*R*)-6-methylheptan-2-yl)hexadecahydro-1*H*-cyclopenta[*a*]phenanthren-3-yl)oxy)-*N*-(*tert*-butyl)acetamide (46)**: Prepared according to **General Procedure F** (Reaction time: 48 h). White solid (42.5 mg, 67% yield, dr 4:1). Flash column chromatography

conditions: Petroleum ether/ EtOAc = 1.7/1. Mp (the major isomer) 61-64 °C. $^1$H NMR (400 MHz, $CDCl_3$, the major isomer) δ 8.00 (d, *J* = 8.0 Hz, 1H), 7.87 (d, *J* = 7.8 Hz, 1H), 7.47 (t, *J* = 7.5 Hz, 1H), 7.38 (t, *J* = 7.4 Hz, 1H), 6.63 (s, 1H), 3.83 – 3.69 (m, 2H), 2.24 – 2.10 (m, 2H), 2.09 – 1.96 (m, 2H), 1.95 – 1.77 (m, 2H), 1.77 – 1.64 (m, 3H), 1.61 – 1.47 (m, 4H), 1.42 (s, 9H), 1.39 – 1.24 (m, 9H), 1.19 – 1.07 (m, 5H), 1.06 – 0.96 (m, 3H), 0.93 – 0.73 (m, 13H), 0.66 (s, 3H). $^{13}$C NMR (100 MHz, $CDCl_3$, the major isomer) δ 176.56, 168.63, 153.04, 135.10, 126.21, 125.47, 123.43, 121.88, 80.47, 63.64, 56.68, 56.40, 54.54, 51.04, 42.76, 41.30, 40.16, 39.65, 37.45, 36.31, 35.94, 35.80, 35.63, 34.21, 32.23, 32.12, 29.03, 28.35, 28.16, 24.33, 23.99, 22.97, 22.71, 21.18, 18.82, 12.24, 11.98. HRMS (ESI, the major isomer) *m/z* calcd. for $C_{40}H_{63}N_2O_2S$ $[M+H]^+$ 635.4610, found 635.4574.

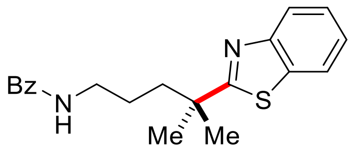


**47**

***N*-(4-(benzo[*d*]thiazol-2-yl)-4-methylpentyl)benzamide (47)**: Prepared according to **General Procedure F** (Reaction time: 25 h). Colorless oil (52.0 mg, 77% yield). Flash column chromatography conditions: Petroleum ether/ EtOAc = 2/1. $^1$H NMR (400 MHz, $CDCl_3$) δ 7.89 (d, *J* = 8.1 Hz, 1H), 7.83 (d, *J* = 7.8 Hz, 1H), 7.79 – 7.74 (m, 2H), 7.50 – 7.45 (m, 1H), 7.44 – 7.37 (m, 3H), 7.36 – 7.30 (m, 1H), 6.49 (s, 1H), 3.39 (q, *J* = 6.8 Hz, 2H), 1.98 – 1.90 (m, 2H), 1.64 – 1.54 (m, 2H), 1.50 (s, 6H). $^{13}$C NMR (100 MHz, $CDCl_3$) δ 180.91, 167.71, 153.16, 135.00, 134.92, 131.39, 128.60, 127.05, 125.91, 124.77, 122.74, 121.62, 41.49, 40.45, 40.19, 29.00, 25.02. HRMS (ESI) *m/z* calcd. for $C_{20}H_{23}N_2OS$ $[M+H]^+$ 339.1531, found 339.1520.

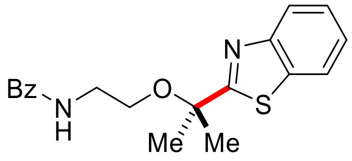


**48**

***N*-(2-((2-(benzo[*d*]thiazol-2-yl)propan-2-yl)oxy)ethyl)benzamide (48)**: Prepared according to **General Procedure F** (Reaction time: 24 h). White solid (58.9 mg, 87%

yield). Flash column chromatography conditions: Petroleum ether/ EtOAc = 2/1. Mp 73-75 °C. $^{1}H$ NMR (400 MHz, CDCl) δ 7.88 – 7.81 (m, 3H), 7.79 (d, *J* = 7.8 Hz, 1H), 7.55 – 7.49 (m, 1H), 7.48 – 7.38 (m, 3H), 7.37 – 7.31 (m, 1H), 7.07 (s, 1H), 3.71 – 3.61 (m, 4H), 1.74 (s, 6H). $^{13}C$ NMR (100 MHz, $CDCl_3$) δ 177.83, 167.66, 152.99, 135.25, 134.85, 131.46, 128.63, 127.19, 126.08, 125.25, 123.17, 121.75, 78.26, 62.63, 40.37, 27.71. HRMS (ESI) *m/z* calcd. for $C_{19}H_{21}N_2O_2S$ $[M+H]^+$ 341.1323, found 341.1313.

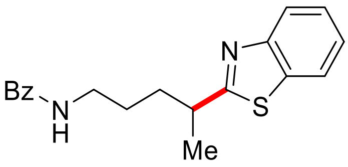


**49**

***N*-(4-(benzo[*d*]thiazol-2-yl)pentyl)benzamide (49)**: Prepared according to **General Procedure F** (Reaction time: 25 h). Colorless oil (22.5 mg, 35% yield). Flash column chromatography conditions: Petroleum ether/ EtOAc = 1.5/1. $^{1}H$ NMR (400 MHz, $CDCl_3$) δ 7.93 (d, *J* = 8.1 Hz, 1H), 7.85 (d, *J* = 7.6 Hz, 1H), 7.81 – 7.76 (m, 2H), 7.50 – 7.45 (m, 1H), 7.45 – 7.38 (m, 3H), 7.37 – 7.32 (m, 1H), 6.56 (s, 1H), 3.54 – 3.42 (m, 2H), 3.41 – 3.31 (m, 1H), 2.04 – 1.92 (m, 1H), 1.88 – 1.81 (m, 1H), 1.77 – 1.62 (m, 2H), 1.47 (d, *J* = 7.0 Hz, 3H). $^{13}C$ NMR (100 MHz, $CDCl_3$) δ 177.49, 167.71, 153.03, 134.83, 134.72, 131.44, 128.62, 127.06, 126.06, 124.89, 122.67, 121.77, 40.03, 39.17, 34.90, 27.23, 21.59. HRMS (ESI) *m/z* calcd. for $C_{19}H_{21}N_2OS$ $[M+H]^+$ 325.1374, found 325.1364.

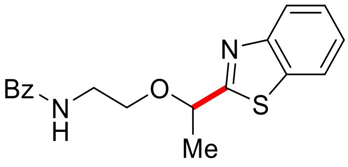


**50**

***N*-(2-(1-(benzo[*d*]thiazol-2-yl)ethoxy)ethyl)benzamide (50)**: Prepared according to **General Procedure F** (Reaction time: 24 h). White solid (53.2 mg, 81% yield). Flash column chromatography conditions: Petroleum ether/ EtOAc = 1/1. Mp 78-80 °C. $^{1}H$ NMR (400 MHz, $CDCl_3$) δ 7.92 (d, *J* = 7.9 Hz, 1H), 7.86 – 7.77 (m, 3H), 7.53 – 7.33 (m, 5H), 6.87 (s, 1H), 4.90 (q, *J* = 6.6 Hz, 1H), 3.83 – 3.77 (m, 1H), 3.76 – 3.67 (m,

3H), 1.66 (d, *J* = 6.6 Hz, 3H). $^{13}$C NMR (100 MHz, $CDCl_3$) δ 175.03, 167.61, 153.06, 134.75, 134.66, 131.52, 128.62, 127.11, 126.21, 125.32, 123.14, 121.92, 76.50, 68.53, 40.03, 22.52. HRMS (ESI) *m/z* calcd. for $C_{18}H_{19}N_2O_2S$ $[M+H]^+$ 327.1167, found 327.1164.

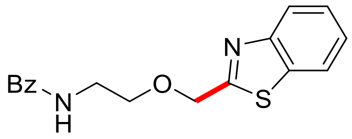


**51**

***N*-(2-(benzo[*d*]thiazol-2-ylmethoxy)ethyl)benzamide (51)**: Prepared according to **General Procedure F** (Reaction time: 24 h). White solid (19.5 mg, 31% yield). Flash column chromatography conditions: Petroleum ether/ EtOAc = 1/1. Mp 64-66 °C. $^{1}$H NMR (400 MHz, Chloroform-*d*) δ 7.95 (d, *J* = 8.1 Hz, 1H), 7.89 – 7.84 (m, 1H), 7.84 – 7.79 (m, 2H), 7.53 – 7.49 (m, 1H), 7.49 – 7.41 (m, 3H), 7.41 – 7.36 (m, 1H), 6.83 (s, 1H), 4.96 (s, 2H), 3.87 – 3.83 (m, 2H), 3.77 – 3.72 (m, 2H). $^{13}$C NMR (100 MHz, $CDCl_3$) δ 169.47, 167.69, 153.12, 134.96, 134.61, 131.62, 128.69, 127.15, 126.34, 125.43, 123.22, 121.91, 70.46, 70.31, 39.95. HRMS (ESI) *m/z* calcd. for $C_{17}H_{17}N_2O_2S$ $[M+H]^+$ 313.1010, found 313.1001.

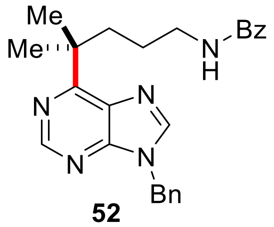


**52**

***N*-(4-(9-benzyl-9*H*-purin-6-yl)-4-methylpentyl)benzamide (52)**: Prepared according to **General Procedure F** (Reaction time: 24 h). Colorless oil (52.9 mg, 64% yield). Flash column chromatography conditions: EtOAc. $^{1}$H NMR (400 MHz, $CDCl_3$) δ 8.88 (s, 1H), 7.82 – 7.73 (m, 3H), 7.48 – 7.42 (m, 1H), 7.39 – 7.32 (m, 4H), 7.31 – 7.27 (m, 2H), 7.07 (s, 1H), 5.36 (s, 2H), 3.38 (q, *J* = 6.3 Hz, 2H), 2.32 – 2.26 (m, 2H), 1.55 (s, 6H), 1.53 – 1.47 (m, 2H). $^{13}$C NMR (100 MHz, $CDCl_3$) δ 168.21, 167.85, 152.05, 151.56, 142.63, 135.43, 135.09, 131.41, 131.19, 129.24, 128.70, 128.46, 128.11, 127.07, 47.34, 42.01, 40.51, 38.42, 27.97, 24.74. HRMS (ESI) *m/z* calcd. for $C_{25}H_{28}N_5O$ $[M+H]^+$ 414.2294, found 414.2301.

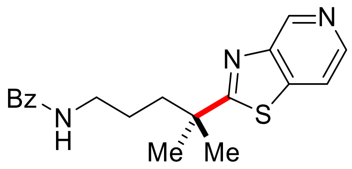

**53**

***N*-(4-methyl-4-(thiazolo[4,5-*c*]pyridin-2-yl)pentyl)benzamide (53)**: Prepared according to **General Procedure F** (Reaction time: 24 h). Colorless oil (44.6 mg, 66% yield). Flash column chromatography conditions: Petroleum ether/ EtOAc = 1/1. $^1$H NMR (400 MHz, $CDCl_3$) δ 9.16 (s, 1H), 8.43 (d, *J* = 5.4 Hz, 1H), 7.80 – 7.68 (m, 3H), 7.48 – 7.43 (m, 1H), 7.41 – 7.35 (m, 2H), 6.58 (s, 1H), 3.39 (q, *J* = 6.9 Hz, 2H), 1.96 – 1.88 (m, 2H), 1.61 – 1.53 (m, 2H), 1.49 (s, 6H). $^{13}$C NMR (100 MHz, $CDCl_3$) δ 182.29, 167.72, 149.88, 144.70, 143.37, 143.11, 134.78, 131.46, 128.60, 126.97, 116.51, 41.80, 40.58, 40.15, 28.84, 25.11. HRMS (ESI) *m/z* calcd. for $C_{19}H_{22}N_3OS$ $[M+H]^+$ 340.1483, found 340.1471.

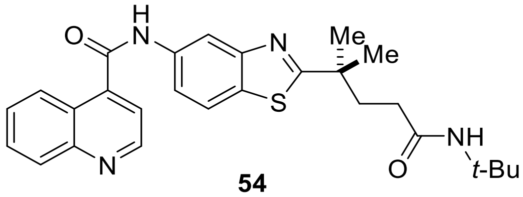

**54**

***N*-(2-(5-(*tert*-butylamino)-2-methyl-5-oxopentan-2-yl)benzo[*d*]thiazol-5-yl)quinoline-4-carboxamide (54)**: Prepared according to **General Procedure F** (Reaction time: 24 h). White solid (192.2 mg, 81% yield). Flash column chromatography conditions: DCM/ EtOAc = 2/1. Mp 273-275 °C. $^1$H NMR (400 MHz, DMSO-*d*$_6$) δ 10.98 (s, 1H), 9.07 (d, *J* = 4.3 Hz, 1H), 8.55 – 8.49 (m, 1H), 8.19 – 8.16 (m, 1H), 8.14 (d, *J* = 8.4 Hz, 1H), 8.05 (d, *J* = 8.7 Hz, 1H), 7.89 – 7.83 (m, 1H), 7.77 (d, *J* = 4.3 Hz, 1H), 7.76 – 7.68 (m, 2H), 7.37 (s, 1H), 1.98 (s, 4H), 1.45 (s, 6H), 1.20 (s, 9H). $^{13}$C NMR (100 MHz, DMSO-*d*$_6$) δ 181.37, 171.16, 165.40, 153.08, 150.36, 147.97, 141.88, 137.12, 130.00, 129.84, 129.50, 127.67, 125.22, 123.95, 122.08, 119.24, 117.81, 113.33, 49.75, 40.90, 38.96, 31.60, 28.48, 28.00. HRMS (ESI) *m/z* calcd. for $C_{27}H_{31}N_4O_2S$ $[M+H]^+$ 475.2167, found 475.2143.

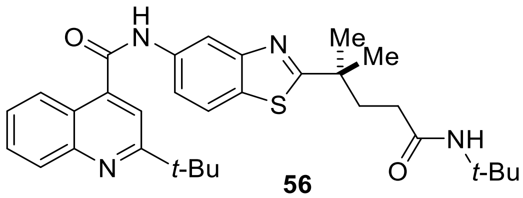

56

**2-(*tert*-butyl)-N-(2-(5-(*tert*-butylamino)-2-methyl-5-oxopentan-2-yl)benzo[*d*]thiazol-5-yl)quinoline-4-carboxamide (56)**: **54** (0.1 mmol, 1.0 equiv), redox active ester **55** (0.2 mmol, 2.0 equiv), Ir(dFCF$_3$ppy)$_2$(dtbbpy)PF$_6$ (2.0 mol %) were placed in vial equipped with a stirring bar. The vial was evacuated and backfilled with nitrogen for 3-5 times. DMA (1.0 mL) and TFA (0.2 mmol, 2.0 equiv) was added via a syringe. The mixture was then irradiated by two 90 W blue lamps at room temperature for 21 h. The mixture was quenched with 0.1 mL Et$_3$N and 20 mL water, then extracted with ethyl acetate (3 x 20 mL). The organic layers were combined and concentrated under vacuo. The product was purified by flash column chromatography on silica gel (petroleum ether/ EtOAc = 2.5/1) to afford **56** (45.2 mg, 85% yield) as a white solid. Mp 140-143 °C. $^1$H NMR (400 MHz, CDCl$_3$) δ 8.59 (s, 1H), 8.41 (d, *J* = 1.7 Hz, 1H), 8.10 (dd, *J* = 14.3, 8.1 Hz, 2H), 7.75 (d, *J* = 8.6 Hz, 1H), 7.72 – 7.65 (m, 2H), 7.59 (dd, *J* = 8.6, 2.1 Hz, 1H), 7.52 – 7.46 (m, 1H), 5.50 (s, 1H), 2.07 – 2.02 (m, 2H), 1.99 – 1.93 (m, 2H), 1.45 (s, 9H), 1.43 (s, 6H), 1.25 (s, 9H). $^{13}$C NMR (100 MHz, CDCl$_3$) δ 181.89, 172.16, 168.96, 166.53, 153.72, 148.04, 142.01, 136.01, 131.49, 130.03, 129.78, 126.99, 124.64, 122.59, 121.86, 118.36, 115.97, 114.72, 51.19, 41.38, 39.29, 38.45, 33.17, 30.16, 28.82, 28.68. HRMS (ESI) *m/z* calcd. for $C_{31}H_{39}N_4O_2S$ $[M+H]^+$ 531.2793, found 531.2760.

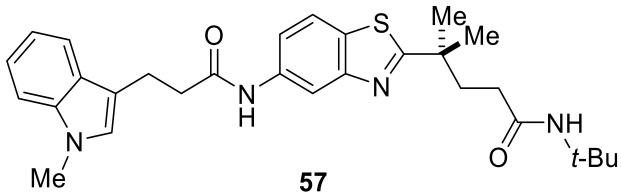

57

***N*-(tert-butyl)-4-methyl-4-(5-(3-(1-methyl-1*H*-indol-3-yl)propanamido)benzo-[*d*]thiazol-2-yl)pentanamide (57)**: Prepared according to **General Procedure F** (Reaction time: 24 h). White solid (201.9 mg, 80% yield). Flash column

chromatography conditions: Toluene/ EtOAc = 2/1. Mp 86-89 °C. $^1$H NMR (400 MHz, $CDCl_3$) δ 8.11, 8.10, 7.74, 7.64, 7.62, 7.61, 7.59, 7.29, 7.28, 7.27, 7.26, 7.25, 7.24, 7.22, 7.20, 7.12, 7.10, 7.08, 6.86, 5.54, 3.67, 3.20, 3.18, 3.16, 2.76, 2.74, 2.72, 2.12, 2.11, 2.10, 2.10, 2.09, 2.07, 2.04, 2.02, 2.01, 2.00, 2.00, 1.98, 1.45, 1.29. $^{13}$C NMR (100 MHz, $CDCl_3$) δ 181.38, 172.25, 171.47, 153.66, 137.15, 136.32, 130.57, 127.56, 126.81, 121.77, 121.47, 118.96, 118.87, 118.23, 114.34, 113.33, 109.43, 51.15, 41.32, 39.34, 38.48, 33.21, 32.68, 28.86, 28.70, 21.21. HRMS (ESI) *m/z* calcd. for $C_{29}H_{37}N_4O_2S$ $[M+H]^+$ 505.2637, found 505.2611.

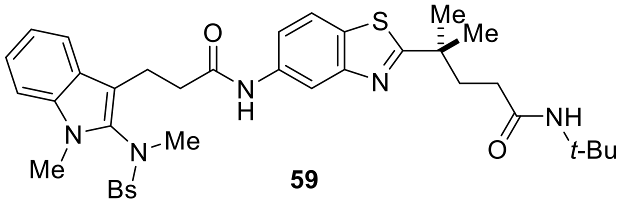


**59**

**4-(5-(3-(2-((4-bromo-*N*-methylphenyl)sulfonamido)-1-methyl-1*H*-indol-3-yl)propanamido)benzo[*d*]thiazol-2-yl)-*N*-(*tert*-butyl)-4-methylpentanamide (59)**:

Route A: **57** as the starting material, to a mixture of the **57** (0.1 mmol, 1.0 equiv), hydroxylamine derivative **58** (0.2 mmol, 2.0 equiv), $NaHCO_3$ (10.1 mg, 1.2 equiv) and $Ir(ppy)_3$ (1.3 mg, 2 mol %) in a vial. The vial was evacuated and backfilled with nitrogen for 3-5 times, DMF (1.5 mL) was added with a syringe under nitrogen. The mixture was then irradiated by 16 W white LED strips. After 24 h, the mixture was quenched by adding 20 mL NaCl and 20 mL of EtOAc. The layers were separated and the aqueous layer was extracted with EtOAc (2 × 20 mL). The combined organic layers were washed with saturated $NaHCO_3$ (20 mL) and brine (20 mL), successively, and then evaporated. The crude product was purified by column chromatography on silica gel (n-hexane/ THF = 1.5/1) to afford the desired product **59** (66.7 mg, 99% yield) as a white solid.

Route B: **60** as the starting material, prepared according to **General Procedure F** (Reaction time: 24 h). White solid (34.4 mg, 51% yield). Flash column chromatography conditions: Toluene/ EtOAc = 2/1. Mp 114-117 °C. $^1$H NMR (400 MHz, $CDCl_3$) δ 8.12 (d, *J* = 1.7 Hz, 1H), 7.86 – 7.81 (m, 2H), 7.76 (s, 1H), 7.68 – 7.60 (m, 2H), 7.60 – 7.51 (m, 3H), 7.29 (dd, *J* = 8.6, 1.9 Hz, 1H), 7.27 – 7.25 (m, 2H), 7.13 – 7.07 (m, 1H), 5.46

(s, 1H), 3.44 (s, 3H), 3.27 (s, 3H), 2.83 – 2.66 (m, 3H), 2.62 – 2.51 (m, 1H), 2.15 – 2.08 (m, 2H), 2.05 – 1.99 (m, 2H), 1.46 (s, 6H), 1.29 (s, 9H). $^{13}$C NMR (100 MHz, $CDCl_3$) δ 181.41, 172.21, 171.12, 153.70, 138.78, 136.52, 135.15, 133.50, 131.73, 130.42, 129.49, 127.67, 125.57, 123.16, 121.53, 119.80, 119.68, 117.86, 113.85, 111.24, 109.95, 51.15, 41.35, 39.40, 39.35, 38.18, 33.27, 29.29, 28.87, 28.72, 20.76. HRMS (ESI) *m/z* calcd. for $C_{36}H_{44}N_5O_4S_2$ $[M+H]^+$ 674.2834, found 674.2805.

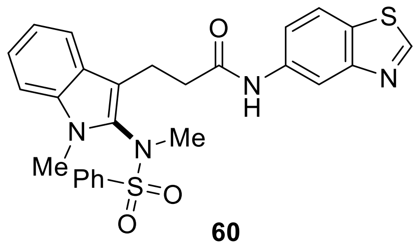


**60**

***N*-(benzo[*d*]thiazol-5-yl)-3-(1-methyl-2-(*N*-methylphenylsulfonamido)-1*H*-indol-3-yl)propanamide (60)**: To a mixture of the **2b’** (0.1 mmol, 1.0 equiv), hydroxylamine derivative **58** (0.2 mmol, 2.0 equiv), $NaHCO_3$ (10.1 mg, 1.2 equiv) and $Ir(ppy)_3$ (1.3 mg, 2 mol%) in a vial. The vial was evacuated and backfilled with nitrogen for 3-5 times, DMSO (1.0 mL) was added with a syringe under nitrogen. The mixture was then irradiated by 16 W white LED strips. After 24 h, the mixture was quenched by adding 20 mL NaCl and 20 mL of EtOAc. The layers were separated and the aqueous layer was extracted with EtOAc (2 × 20 mL). The combined organic layers were washed with saturated $NaHCO_3$ (20 mL) and brine (20 mL), successively, and then evaporated. The crude product was purified by column chromatography on silica gel (DCM/ EtOAc = 5/1) to afford the desired product **60** (49.9 mg, 99% yield) as a light yellow oil. $^1$H NMR (400 MHz, $CDCl_3$) δ 8.95 (s, 1H), 8.16 (d, $J$ = 1.7 Hz, 1H), 7.88 – 7.81 (m, 2H), 7.81 – 7.75 (m, 2H), 7.66 – 7.60 (m, 1H), 7.59 – 7.47 (m, 4H), 7.30 – 7.25 (m, 2H), 7.14 – 7.05 (m, 1H), 3.43 (s, 3H), 3.26 (s, 3H), 2.86 – 2.68 (m, 3H), 2.64 – 2.53 (m, 1H). $^{13}$C NMR (100 MHz, $CDCl_3$) δ 171.23, 155.10, 153.80, 138.76, 136.94, 135.14, 133.50, 131.74, 129.48, 128.97, 127.65, 125.55, 123.19, 121.90, 119.83, 119.65, 118.86, 114.16, 111.18, 109.94, 39.34, 38.19, 29.28, 20.73. HRMS (ESI) *m/z* calcd. for $C_{26}H_{25}N_4O_3S_2$ $[M+H]^+$ 505.1368, found 505.1364.

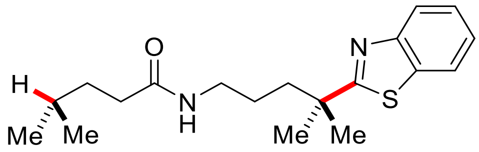

**61**

***N*-(4-(benzo[*d*]thiazol-2-yl)-4-methylpentyl)-4-methylpentanamide (61)**: Prepared according to **General Procedure F** (Reaction time: 25 h). Colorless oil (36.6 mg, 55% yield). Flash column chromatography conditions: Petroleum ether/ EtOAc = 2/1. $^1$H NMR (400 MHz, $CDCl_3$) δ 7.99 – 7.94 (m, 1H), 7.87 – 7.82 (m, 1H), 7.47 – 7.41 (m, 1H), 7.37 – 7.31 (m, 1H), 5.63 (s, 1H), 3.19 (q, $J$ = 6.9 Hz, 2H), 2.17 – 2.10 (m, 2H), 1.89 – 1.81 (m, 2H), 1.59 – 1.40 (m, 11H), 0.88 (d, $J$ = 6.3 Hz, 6H). $^{13}$C NMR (100 MHz, $CDCl_3$) δ 180.87, 173.37, 153.21, 135.03, 125.93, 124.78, 122.73, 121.64, 41.44, 40.73, 39.67, 34.98, 34.75, 28.86, 27.95, 25.09, 22.45. HRMS (ESI) *m/z* calcd. for $C_{19}H_{29}N_2OS$ $[M+H]^+$ 333.2000, found 333.1998.

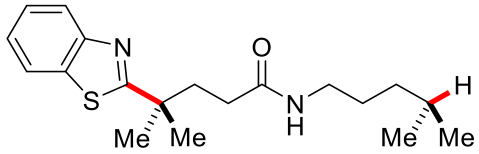

**62**

**4-(benzo[*d*]thiazol-2-yl)-4-methyl-*N*-(4-methylpentyl)pentanamide (62)**: Prepared according to **General Procedure F** (Reaction time: 25 h). Colorless oil (18.0 mg, 27% yield). Flash column chromatography conditions: Petroleum ether/ EtOAc = 2/1. $^1$H NMR (400 MHz, $CDCl_3$) δ 8.00 – 7.95 (m, 1H), 7.88 – 7.83 (m, 1H), 7.48 – 7.42 (m, 1H), 7.38 – 7.32 (m, 1H), 5.51 (s, 1H), 3.19 – 3.11 (m, 2H), 2.22 – 2.15 (m, 2H), 2.15 – 2.07 (m, 2H), 1.51 (s, 6H), 1.47 – 1.38 (m, 2H), 1.19 – 1.11 (m, 2H), 0.85 (d, $J$ = 6.6 Hz, 6H). $^{13}$C NMR (100 MHz, $CDCl_3$) δ 180.28, 172.70, 153.18, 135.15, 125.97, 124.87, 122.82, 121.70, 41.39, 39.93, 39.46, 36.17, 32.59, 28.79, 27.88, 27.59, 22.65. HRMS (ESI) *m/z* calcd. for $C_{19}H_{29}N_2OS$ $[M+H]^+$ 333.2000, found 333.1995.

**Scale-up Experiment**

A 50 mL round bottom flask was equipped with a rubber septum and magnetic stir bar and was charged with **1a** (1.80 g, 5 mmol), benzothiazole **2f** (1.69 g, 12.5 mmol), $K_2CO_3$ (0.69 g, 5 mmol) and 3CzClIPN (65.8 mg, 0.1 mmol, 2 mol %). The flask was

evacuated and backfilled with nitrogen for 3-5 times, DMSO (25 mL, 0.2 M) was added with a syringe under nitrogen. The mixture was then irradiated by two 90 W blue lamps at room temperature. After the reaction was complete (24 h), the mixture was poured into a separatory funnel containing 50 mL of saturated NaCl and 50 mL of EtOAc. The layers were separated and the aqueous layer was extracted with EtOAc (2 × 50 mL). The combined organic layers were washed with saturated $NaHCO_3$ (50 mL) and brine (50 mL), successively, and then evaporated. The crude product was purified by column chromatography on silica gel eluting with Petroleum ether/ EtOAc (10/1 to 2/1) to afford the desired product **8** in 96% yield (1.46 g).

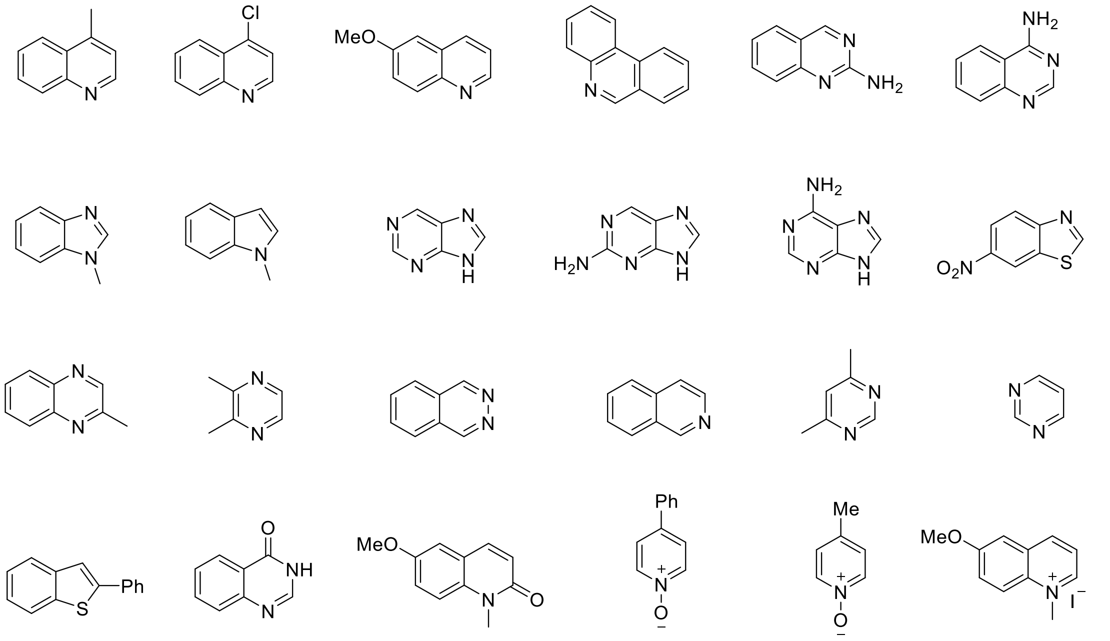

**Supplementary Figure 5. Unsuccessful heteroarenes**

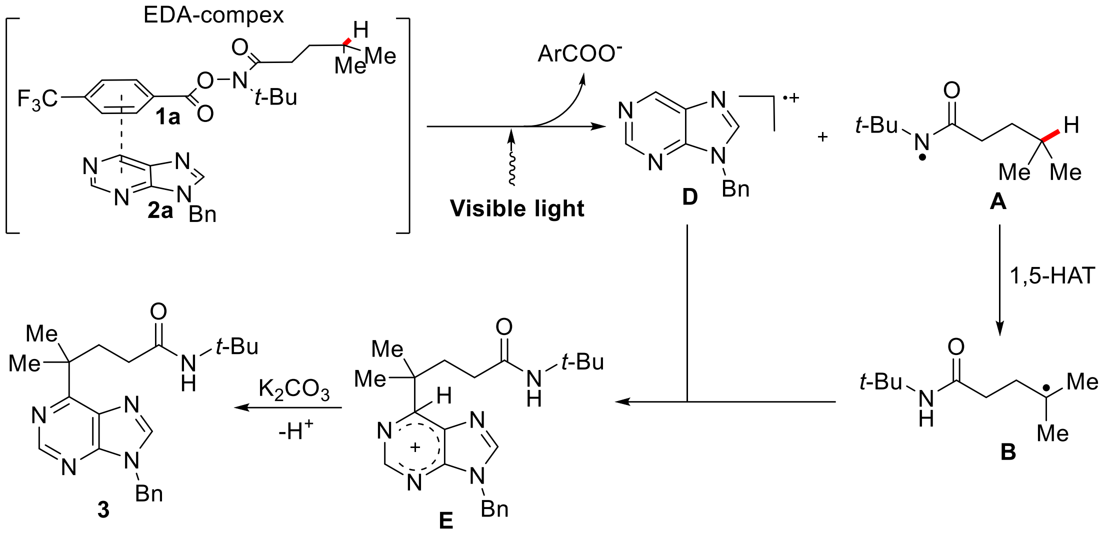


**Supplementary Figure 6. Proposed mechanism via EDA complex**

## Computational Details of Mechanistic Studies

All the calculations were conducted by using the Gaussian 09 packages[10] in solution-phase with SMD solvent model[11] (solvent = DMSO). M06-2X functional[12] with an ultrafine integration grid and the def2-SVP[13] basis set were used for the density functional theory (DFT) and time-dependent DFT (TDDFT) calculations. The TDDFT method was applied to optimize the first singlet excited electronic state geometry of photocatalyst **3CzClIPN**. Frequency calculations at the same level of theory were employed to verify all the stationary points as an intermediate or transition state. The intrinsic reaction coordinate (IRC)[14,15] analysis was carried out to confirm that all the saddle point connected the correct reactant and product on the potential energy surface. Dispersion corrections were added by using the D3 version of Grimme's dispersion with Becke-Johnson damping to give a better description of long-range weak interactions.[16,17] 1.9 kcal/mol was added to the Gibbs free energies of all species to account for the standard state change from 1 atm to 1 mol/L at 298.15 K.[18-20] To identify the reactive sites, atomic dipole corrected Hirshfeld atomic charges (ADCH) were adopted to calculate the Fukui functions indices.[21,22] The ADCH atomic charges were resolved from the wavefunction file from Gaussian 09 calculation with multiwfn 3.6.[23] Fukui indices $f_A^+$ was calculated as $f_A^+ = q_N^A - q_{N+1}^A$ for nucleophilic attack.

**Estimation of activation barriers of the SET process according to Marcus theory:**[24-35]

For a single electron transfer (SET) process, the outer-sphere ET model is applicable and the activation barrier may be estimated from the outer-sphere Marcus-Hush model. Marcus theory of outer-sphere electron transfer can be applied:

$$\Delta G_{ET}^{\ddagger} = \Delta G_0^{\ddagger}\left(1 + \frac{\Delta G_r}{4\Delta G_0^{\ddagger}}\right)^2 \qquad (1)$$

where $\Delta G_0{}^{\ddagger}$ is the intrinsic barrier which is related to the reorganization energy ($\lambda$) by:

$$\Delta G_0^{\ddagger} = \frac{\lambda}{4} \qquad (2)$$

$$\lambda_0 = (332\text{kcal/mol})\left(\frac{1}{2a_1} + \frac{1}{2a_2} - \frac{1}{R}\right)\left(\frac{1}{\varepsilon_{op}} - \frac{1}{\varepsilon}\right) \qquad (3)$$

Since the data are insufficient to calculate the inner reorganization energy for the reactants, $\lambda_i$, and the inner reorganization energies in electron transfer reactions are usually small, $\lambda_i$ could be neglected. Thus, the total reorganization energy $\lambda \approx \lambda_0$.

**Supplementary Table 7.** Estimation of the activation barriers for the SET between **1a** and **3CzClIPN**.

| a1/ Å | a2/ Å | R/ Å | $\varepsilon_{op}$ | $\varepsilon$ | $\lambda_0$/ kcal/mol | $\Delta G_r$/ kcal/mol | $\Delta G_{ET}^{\ddagger}$/ kcal/mol |
|---|---|---|---|---|---|---|---|
| 7.30 | 8.36 | 15.66 | 2.01 | 46.83 | 10.20 | 1.40 | 3.30 |

a1, the radii of the oxidant; a2, the radii of the reactant; R, a1 + a2; $\varepsilon_{op}$, the optical dielectric constant; $\varepsilon$, the static dielectric constant for the DMSO solvent; $\lambda_0$, the solvent reorganization energy; $\Delta G_r$, the reaction energy; $\Delta G_{ET}^{\ddagger}$, calculated activation barriers for ET.

**Computed Gibbs free energy profile**

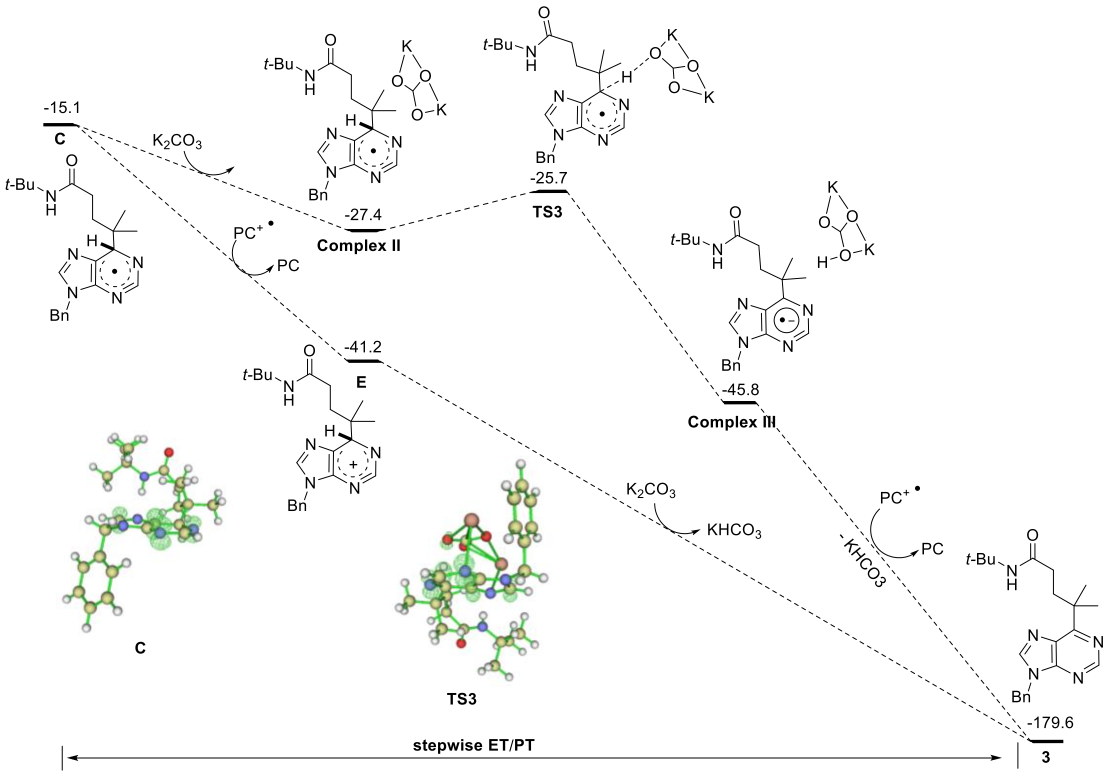


**Supplementary Figure 7.** Computed Gibbs free energy profile for the H-atom loss process in redox neutral coupling reaction of **1a** and **2a** and spin density structure of intermediate **C** and transition state **TS3**. Energies are given in kcal/mol.

**Supplementary Table 8.** Estimation of the activation barriers for the ET process for the equation (a) and (b) according to Marcus theory.

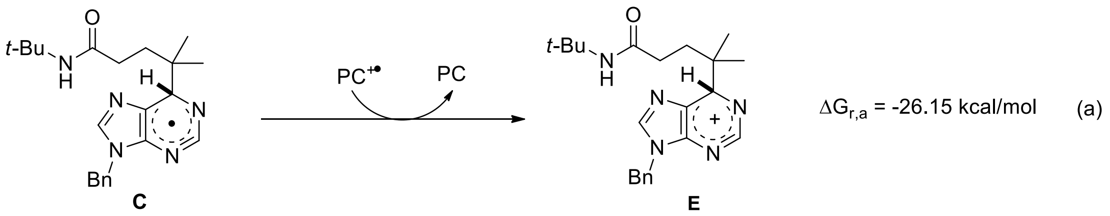


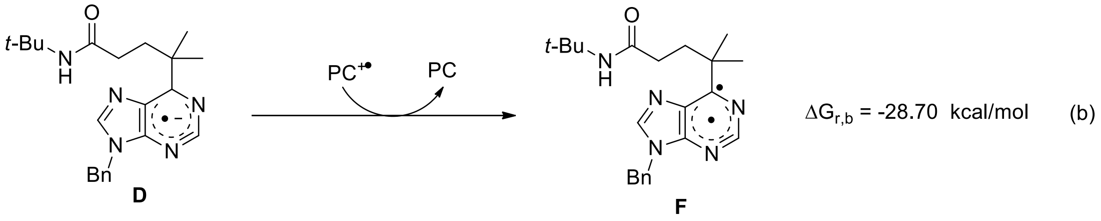


| | a1/ Å | a2/ Å | R/ Å | $\varepsilon_{op}$ | $\varepsilon$ | $\lambda_0$/ kcal/mol | $\Delta G_r$/ kcal/mol | $\Delta G_{ET}^{\ddagger}$/ kcal/mol |
|---|---|---|---|---|---|---|---|---|
| (a) | 8.44 | 7.90 | 16.34 | 2.01 | 46.83 | 9.70 | -26.15 | 6.98 |
| (b) | 8.44 | 7.44 | 15.88 | 2.01 | 46.83 | 10.03 | -28.70 | 9.31 |

Proton Transfer between **E** and $K_2CO_3$:

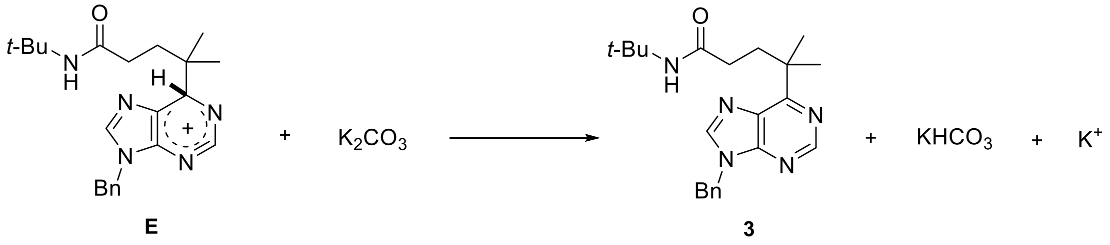


Relaxed PES scan along the C-H bond on mixed systems of **E** and $K_2CO_3$

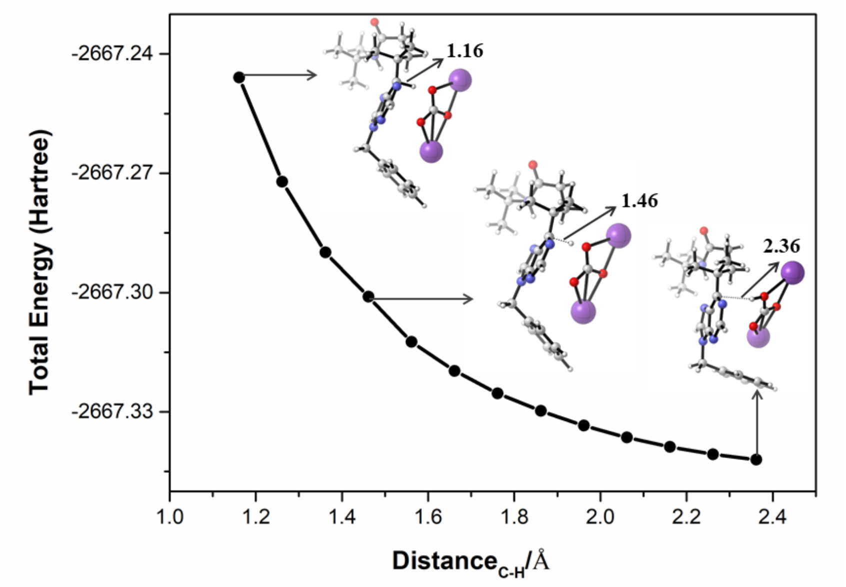


**Supplementary Figure 8.** Relaxed potential energy surface scan along the C-H bond on mixed systems of **E** and $K_2CO_3$.

**Supplementary Table 9.** The selected bond lengths in purine ring of **Complex III** and **3**.

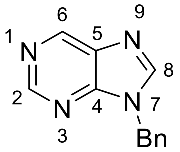


| Susbstance/distance(Å) | C6-N1 | N1-C2 | C2-N3 | N3-C4 | C4-N7 | N7-C8 | C8-N9 | N9-C5 | C5-C6 | C5-C4 |
|---|---|---|---|---|---|---|---|---|---|---|
| **Complex III** | 1.37 | 1.31 | 1.38 | 1.35 | 1.37 | 1.37 | 1.33 | 1.37 | 1.44 | 1.40 |
| **3** | 1.33 | 1.34 | 1.33 | 1.33 | 1.37 | 1.37 | 1.31 | 1.38 | 1.41 | 1.41 |

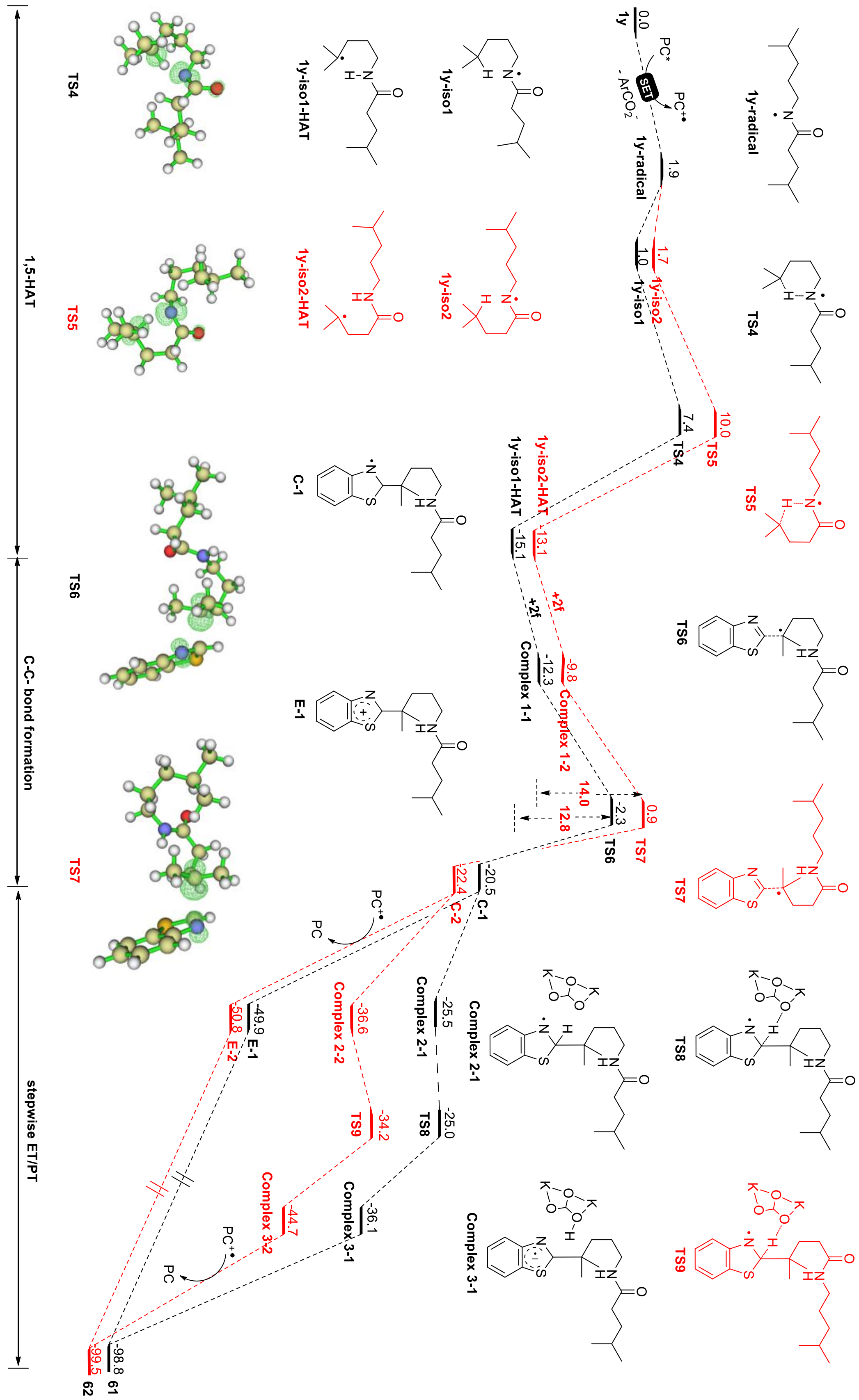


**Supplementary Figure 9.** Computed Gibbs free energy profile for the coupling reaction of **1y** and **2f** generating **61** and **62**, and spin density structure of transition states **TS4**, **TS5**, **TS6** **and TS7**. Energies are given in kcal/mol.

**Supplementary Table 10.** Estimation of the activation barriers for the ET process of the equations (c)-(f) according to Marcus theory.

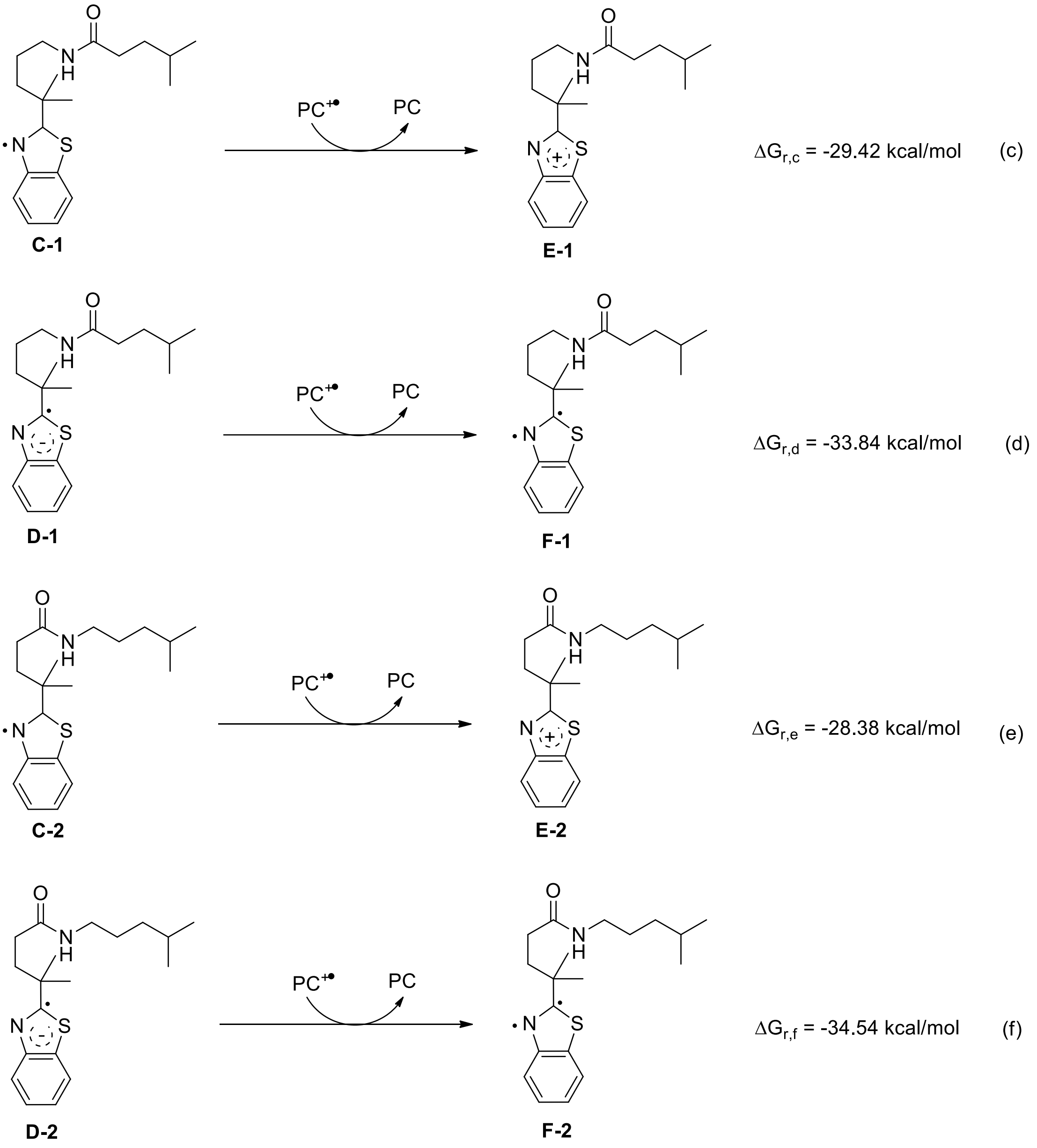


| | a1/ Å | a2/ Å | R/ Å | $\varepsilon_{op}$ | $\varepsilon$ | $\lambda_0$/ kcal/mol | $\Delta G_r$/ kcal/mol | $\Delta G_{ET}^{\ddagger}$/ kcal/mol |
|---|---|---|---|---|---|---|---|---|
| (c) | 8.44 | 8.78 | 17.22 | 2.01 | 46.83 | 9.19 | -29.42 | 11.14 |
| (d) | 8.44 | 9.20 | 17.64 | 2.01 | 46.83 | 9.00 | -33.84 | 17.16 |
| (e) | 8.44 | 8.65 | 17.09 | 2.01 | 46.83 | 9.25 | -28.38 | 9.88 |
| (f) | 8.44 | 7.79 | 16.23 | 2.01 | 46.83 | 9.77 | -34.54 | 15.70 |

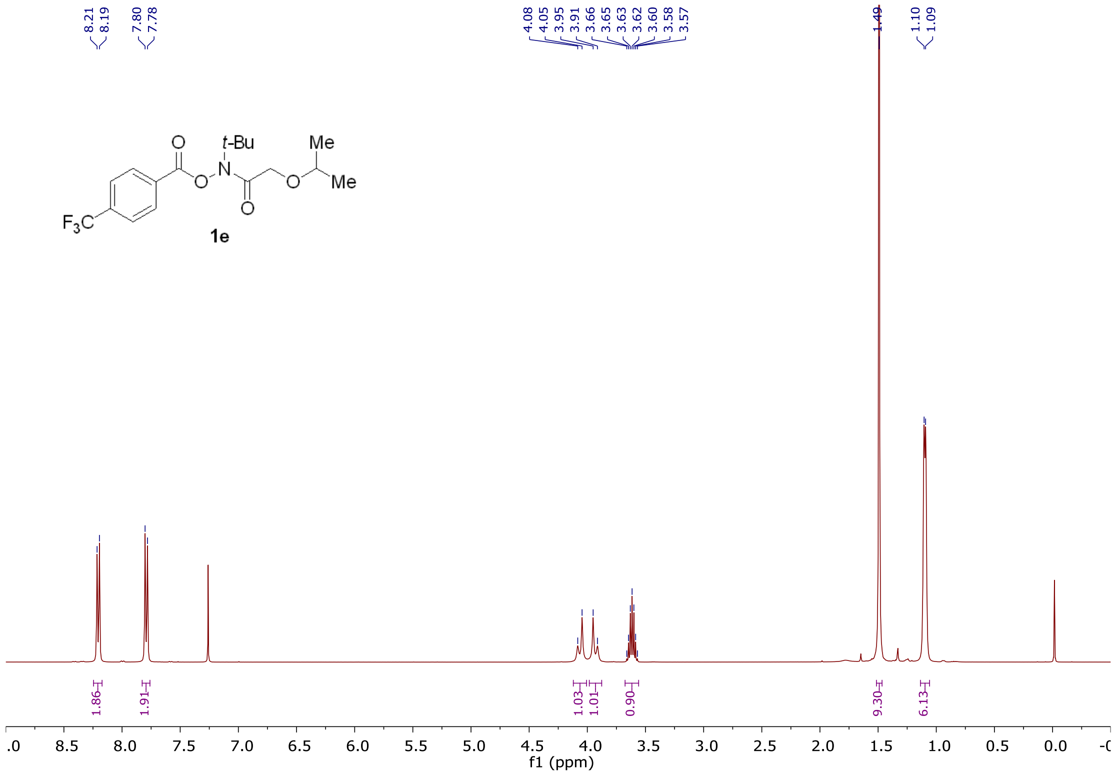


**Supplementary Figure 10.** $^{1}$H NMR spectra for **1e**

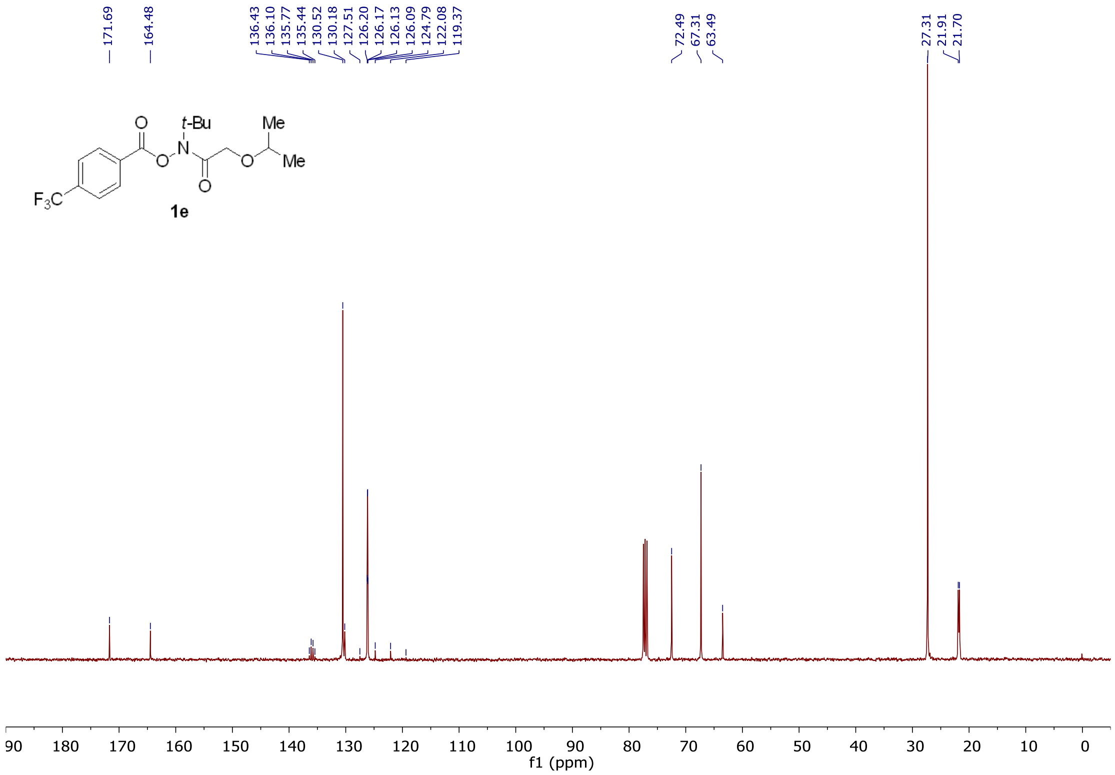


**Supplementary Figure 11.** $^{13}$C NMR spectra for **1e**

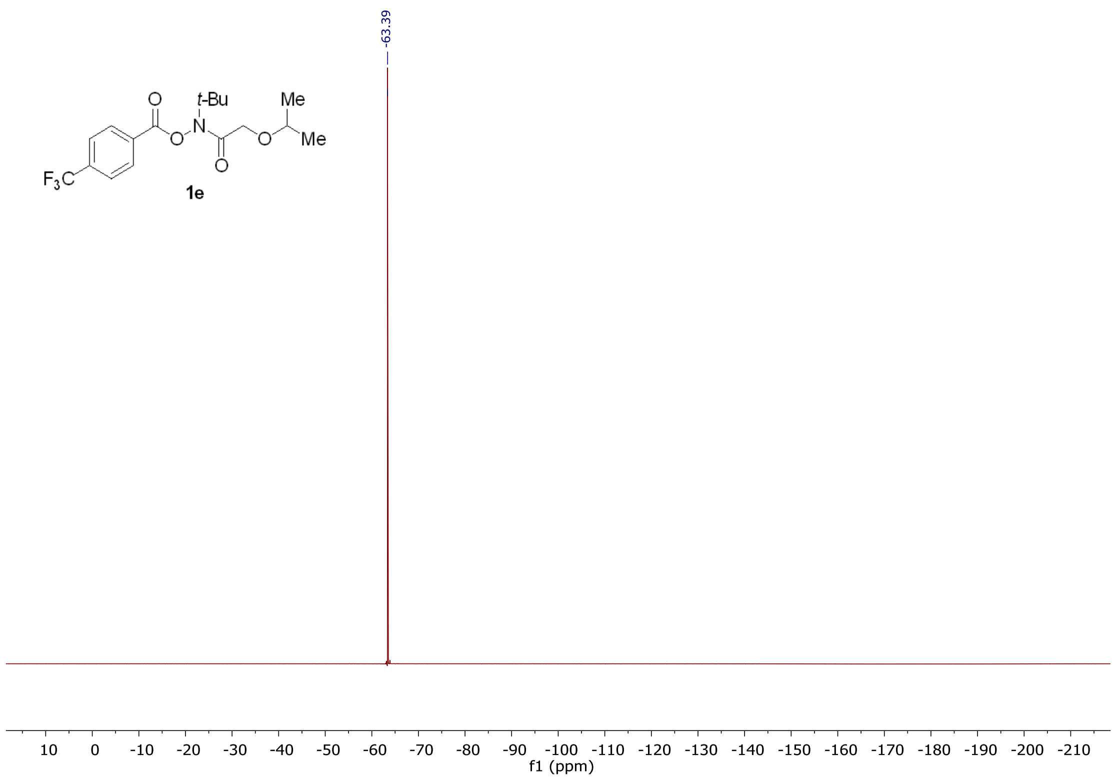


**Supplementary Figure 12.** $^{19}$F NMR spectra for **1e**

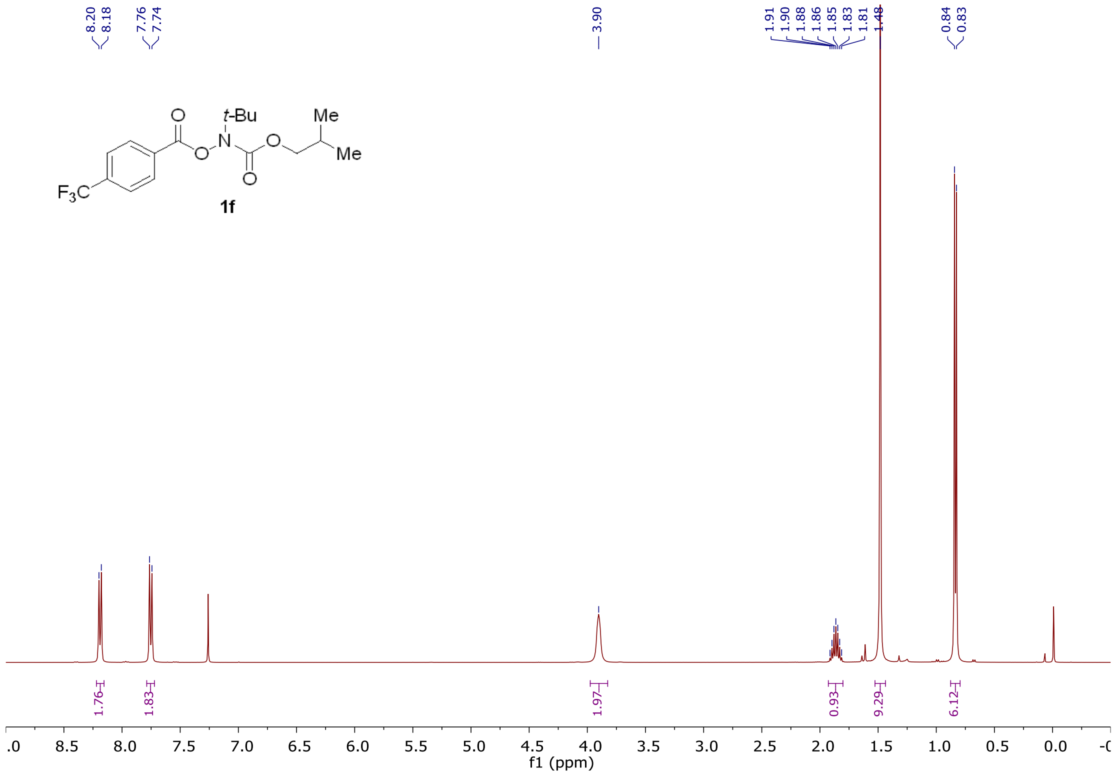


**Supplementary Figure 13.** $^{1}$H NMR spectra for **1f**

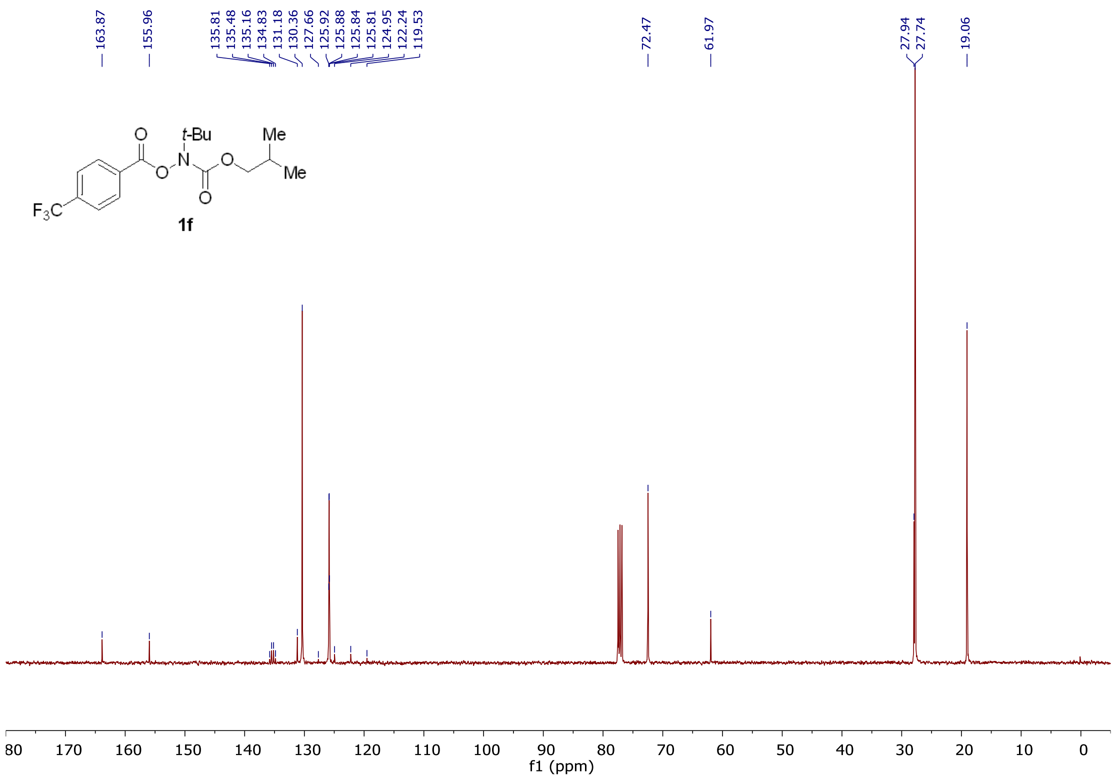


**Supplementary Figure 14.** $^{13}$C NMR spectra for **1f**

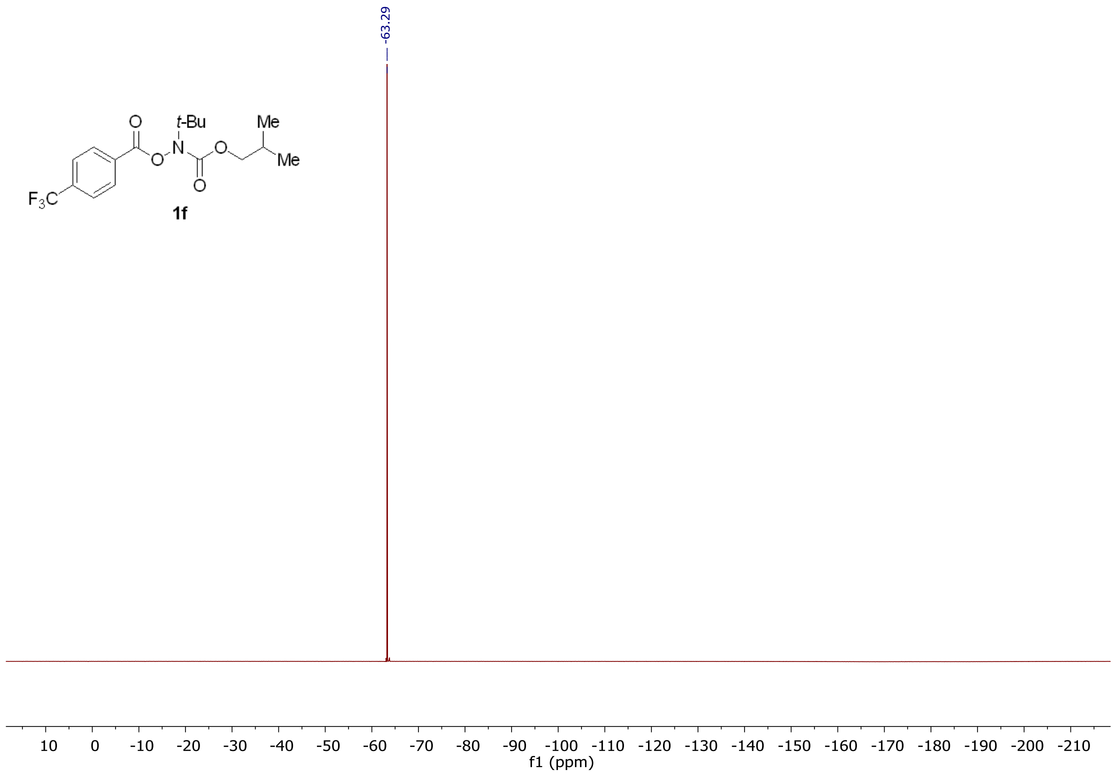


**Supplementary Figure 15.** $^{19}$F NMR spectra for **1f**

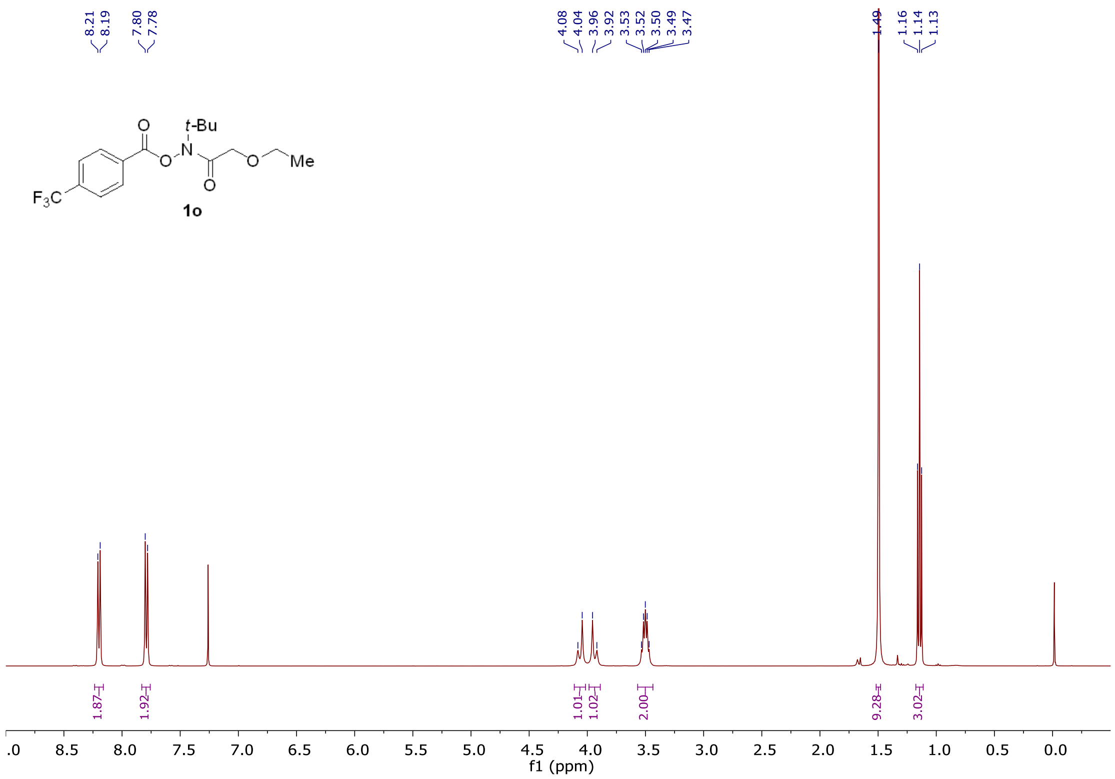


**Supplementary Figure 16.** $^{1}$H NMR spectra for **1o**

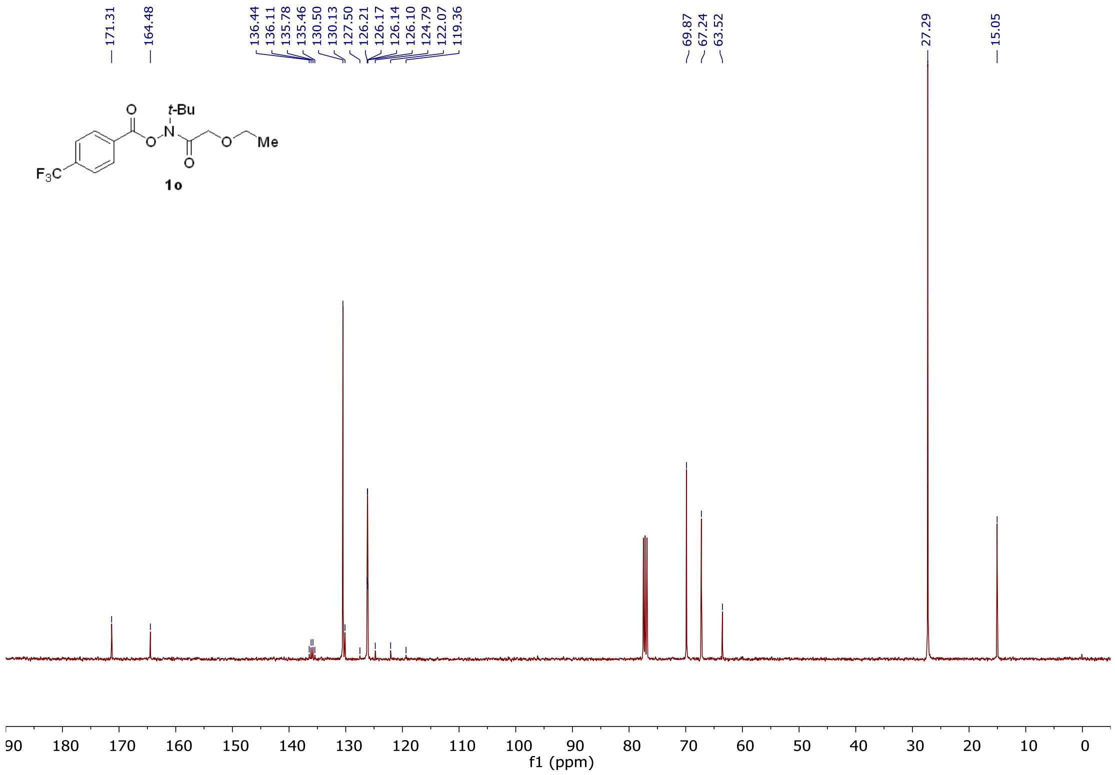


**Supplementary Figure 17.** $^{13}$C NMR spectra for **1o**

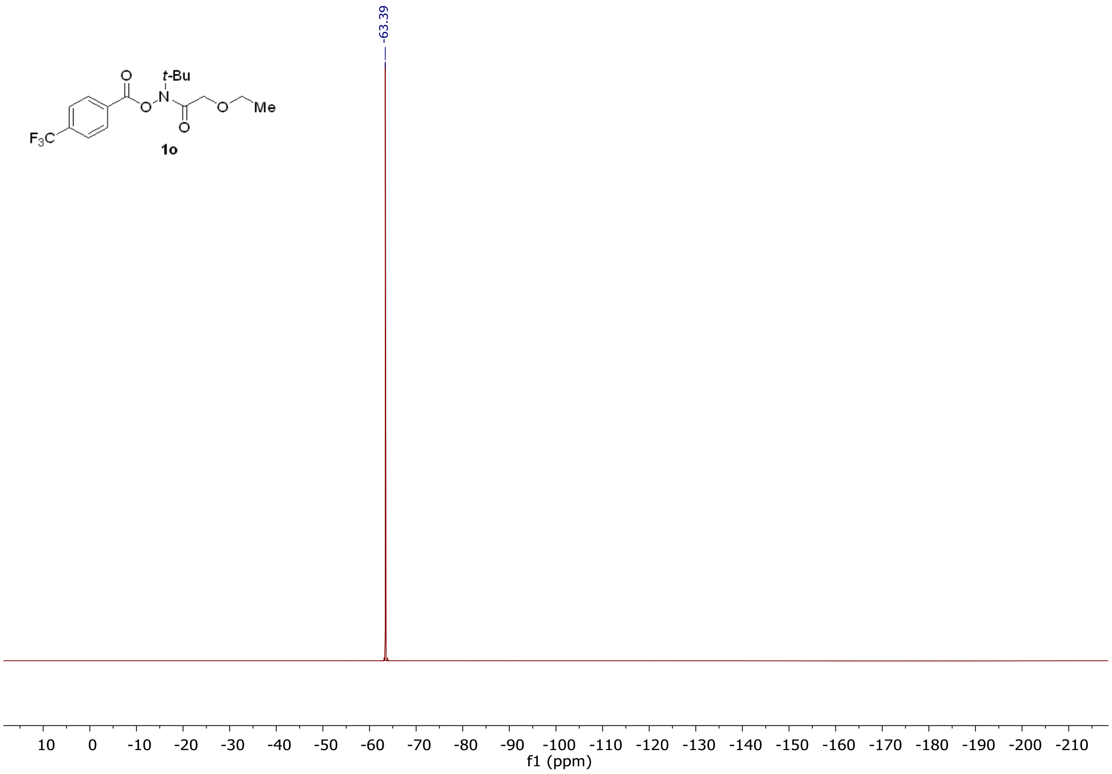


**Supplementary Figure 18.** $^{19}$F NMR spectra for **1o**

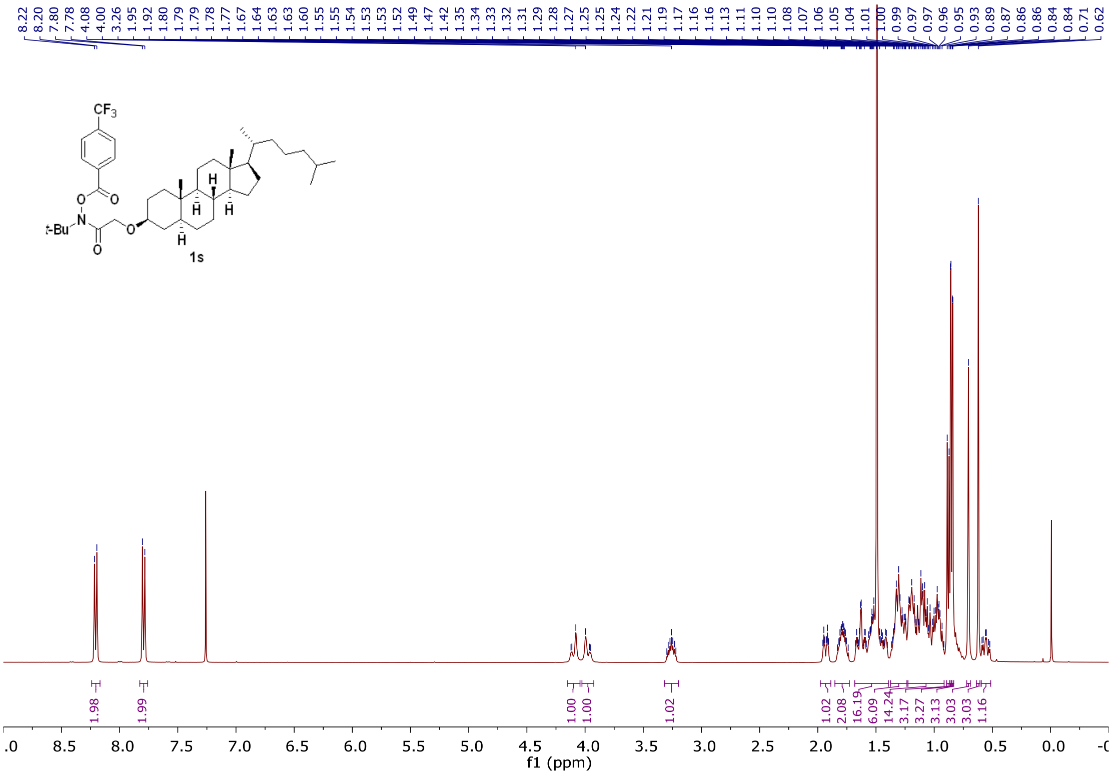


**Supplementary Figure 19.** $^{1}$H NMR spectra for **1s**

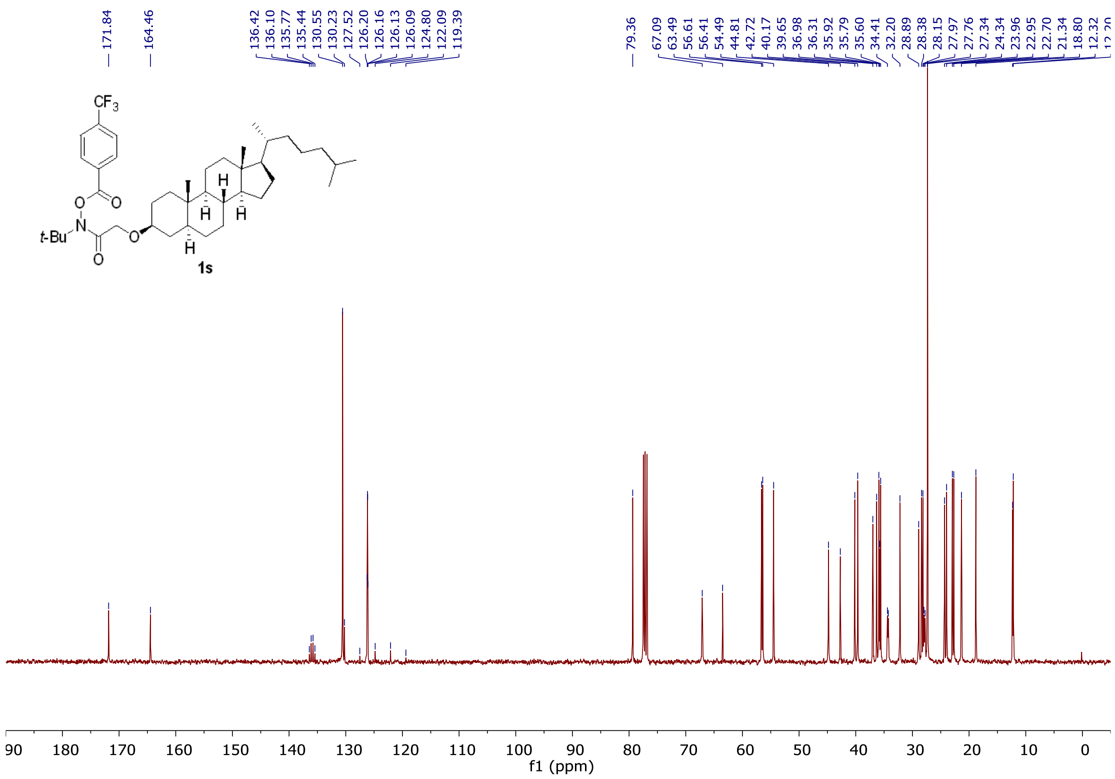

**Supplementary Figure 20.** $^{13}$C NMR spectra for **1s**

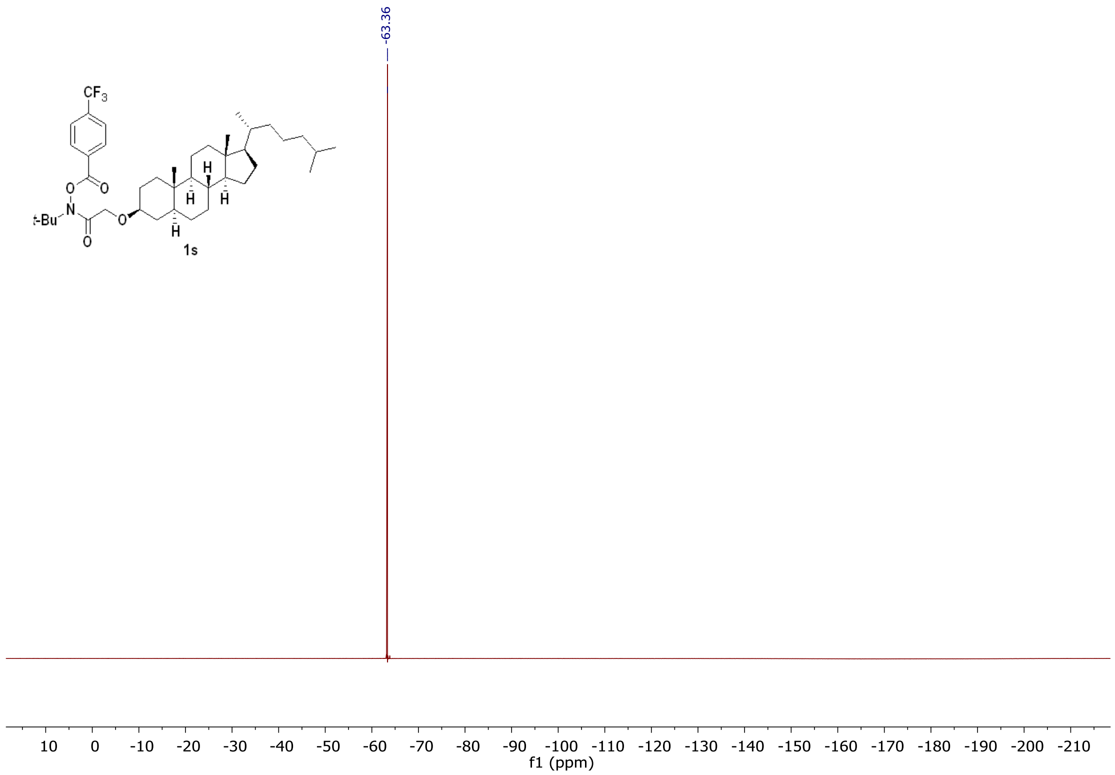

**Supplementary Figure 21.** $^{19}$F NMR spectra for **1s**

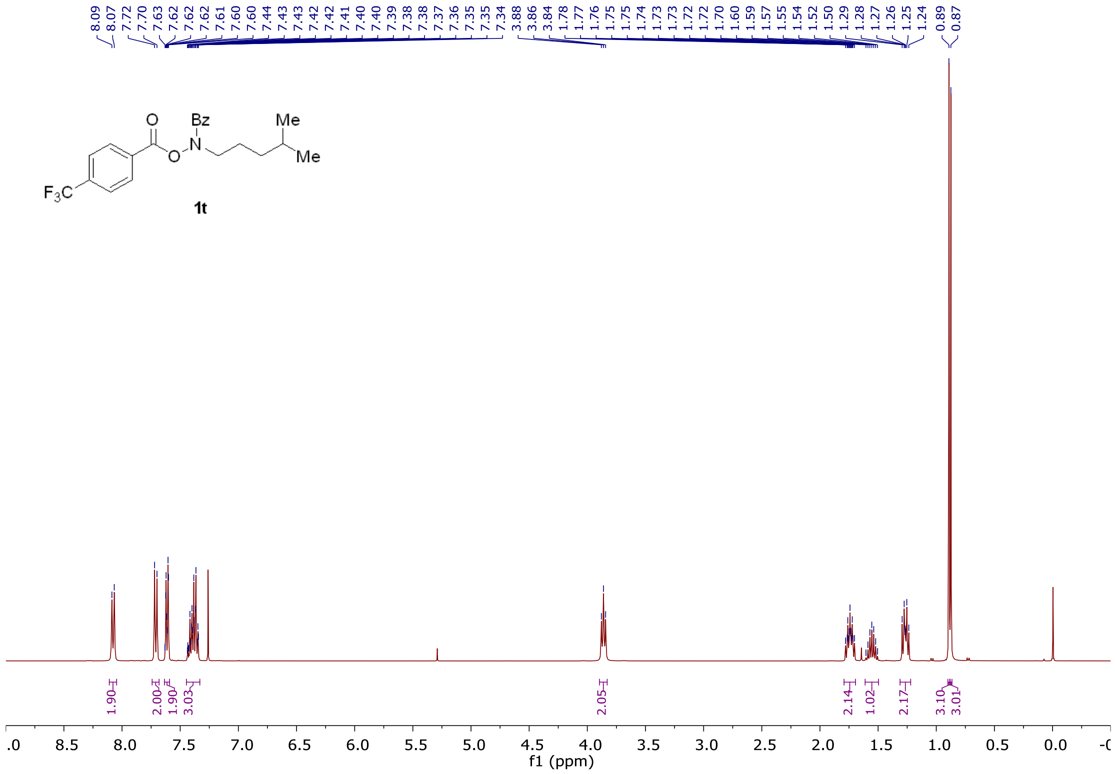

**Supplementary Figure 22.** $^{1}$H NMR spectra for **1t**

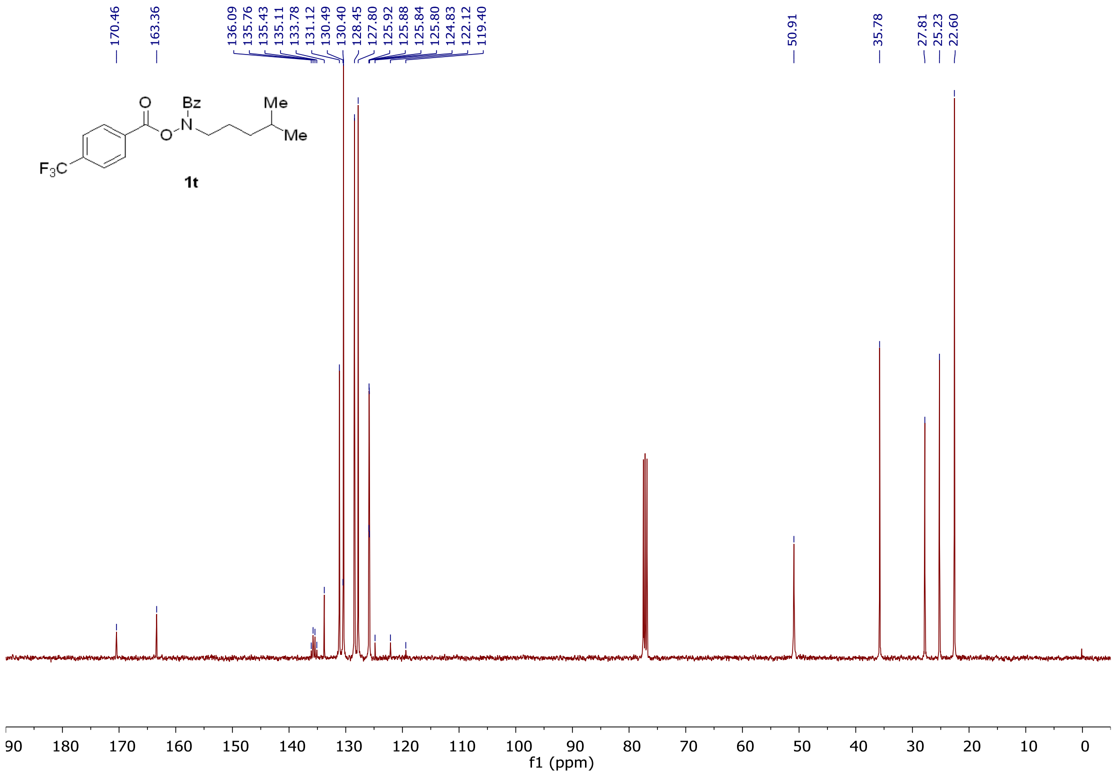

**Supplementary Figure 23.** $^{13}$C NMR spectra for **1t**

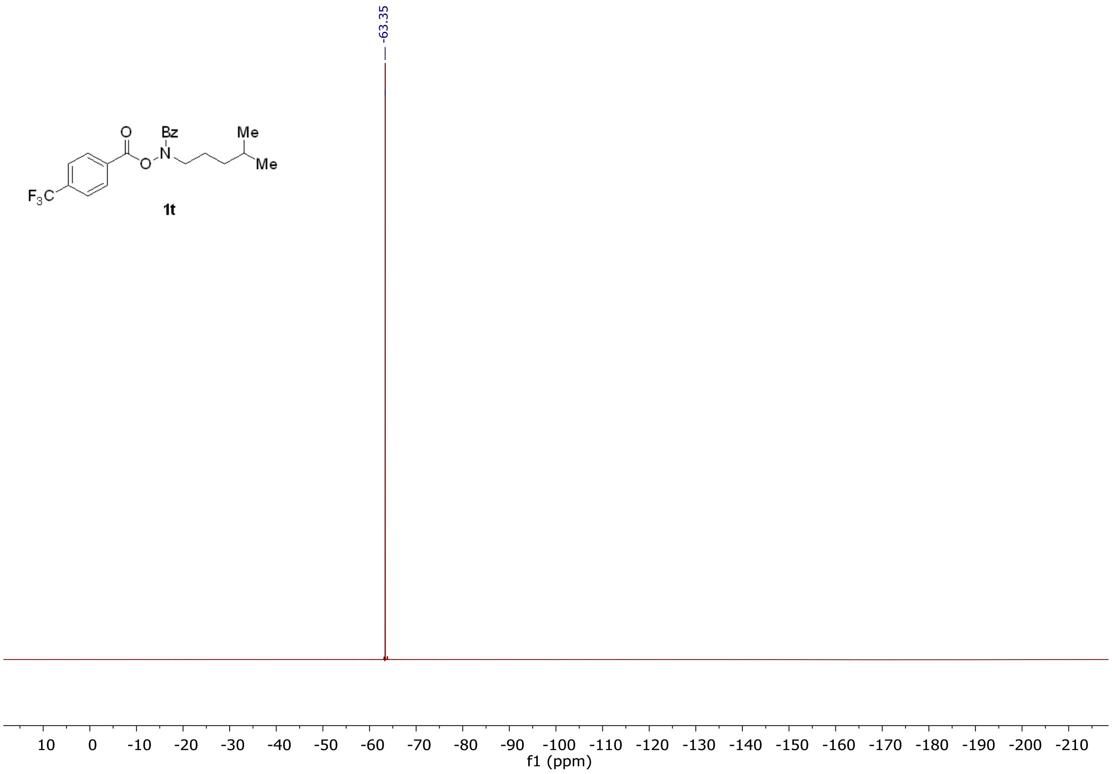


**Supplementary Figure 24.** $^{19}$F NMR spectra for **1t**

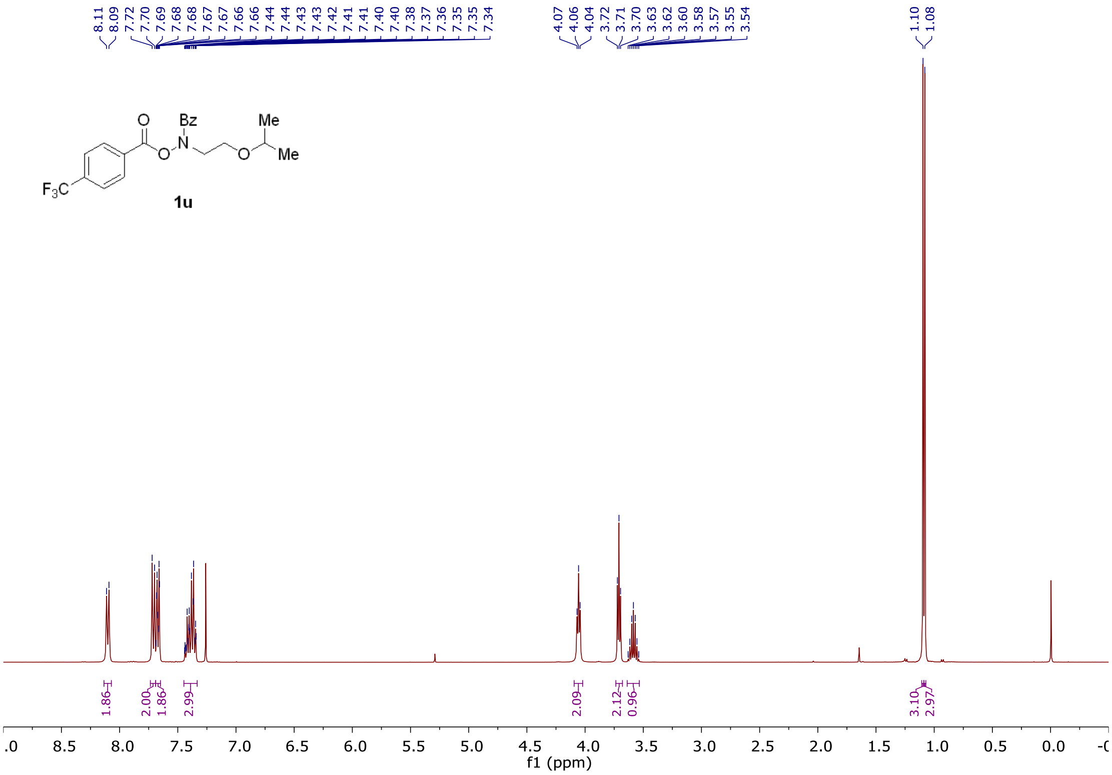


**Supplementary Figure 25.** $^{1}$H NMR spectra for **1u**

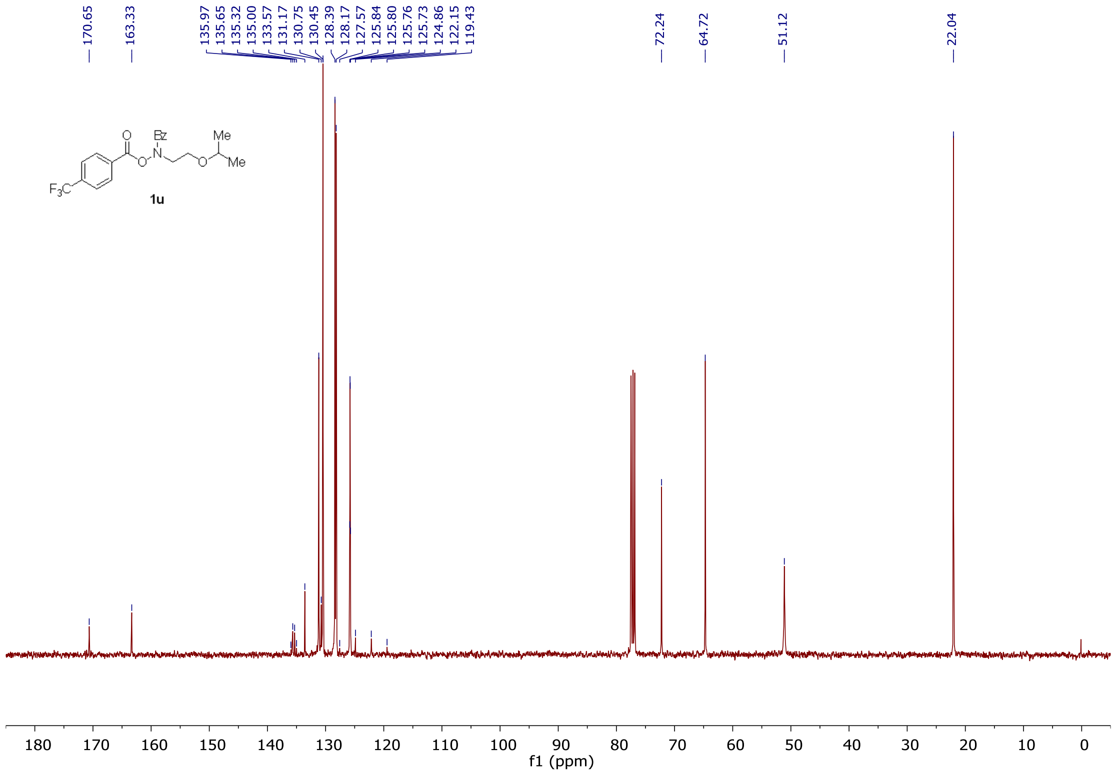


**Supplementary Figure 26.** $^{13}$C NMR spectra for **1u**

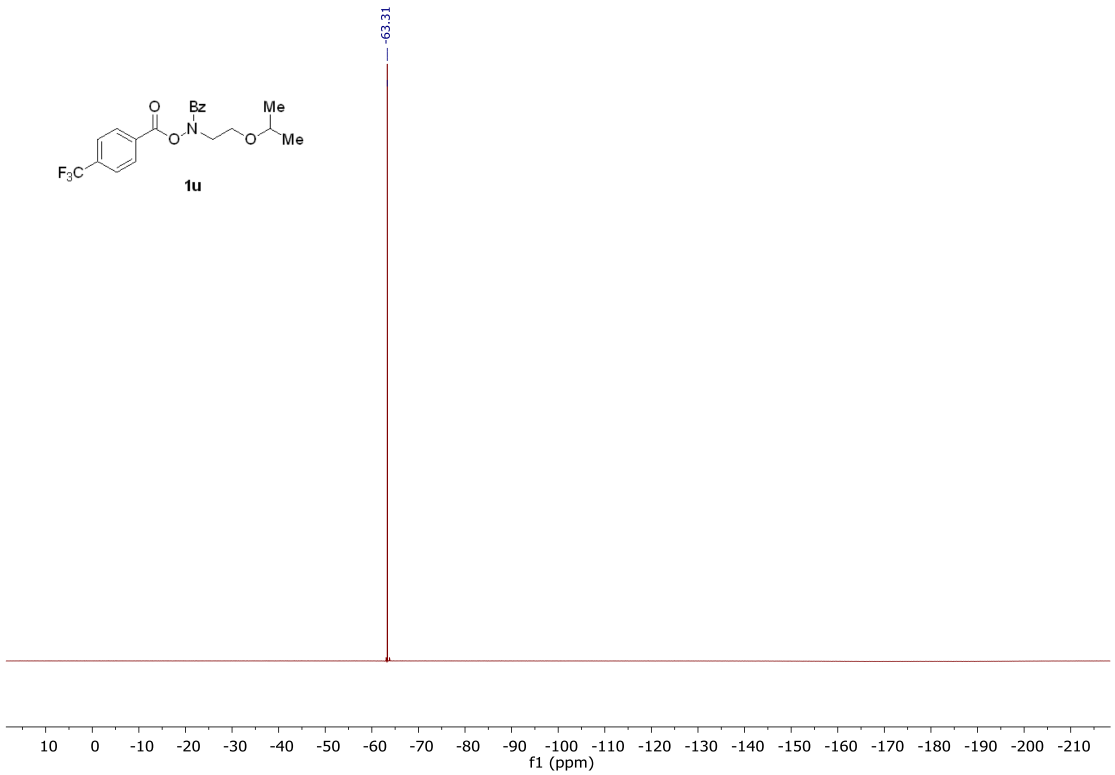


**Supplementary Figure 27.** $^{19}$F NMR spectra for **1u**

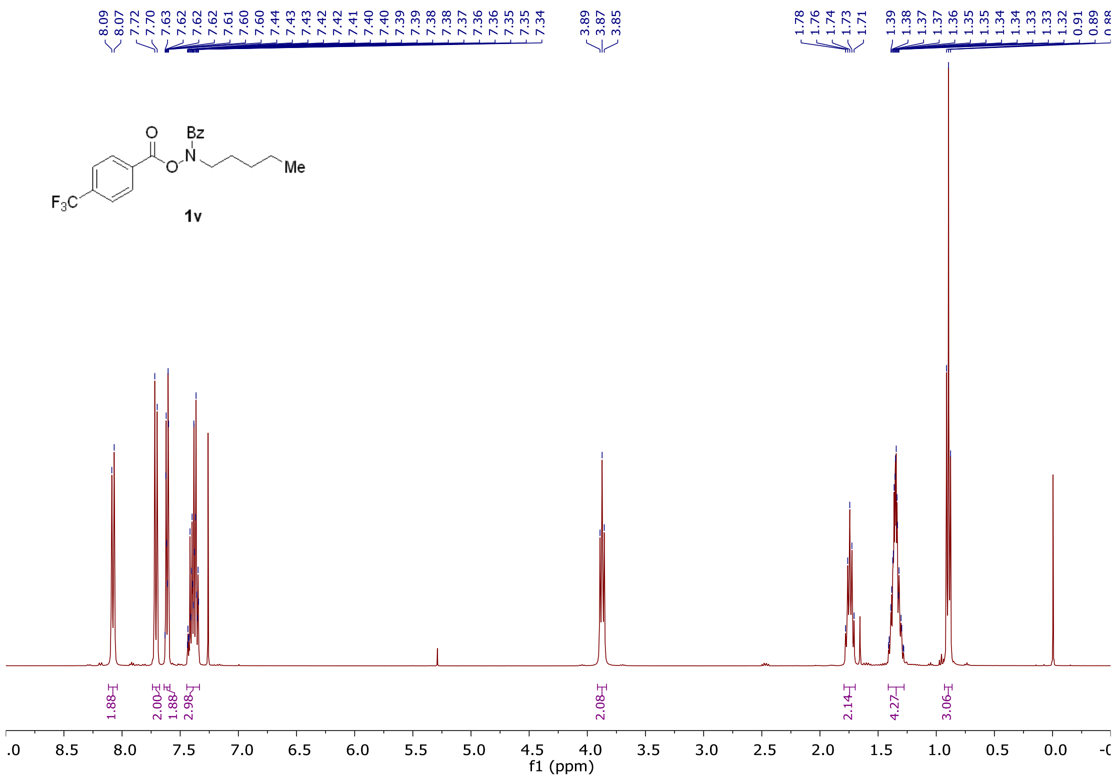

**Supplementary Figure 28.** $^{1}$H NMR spectra for **1v**

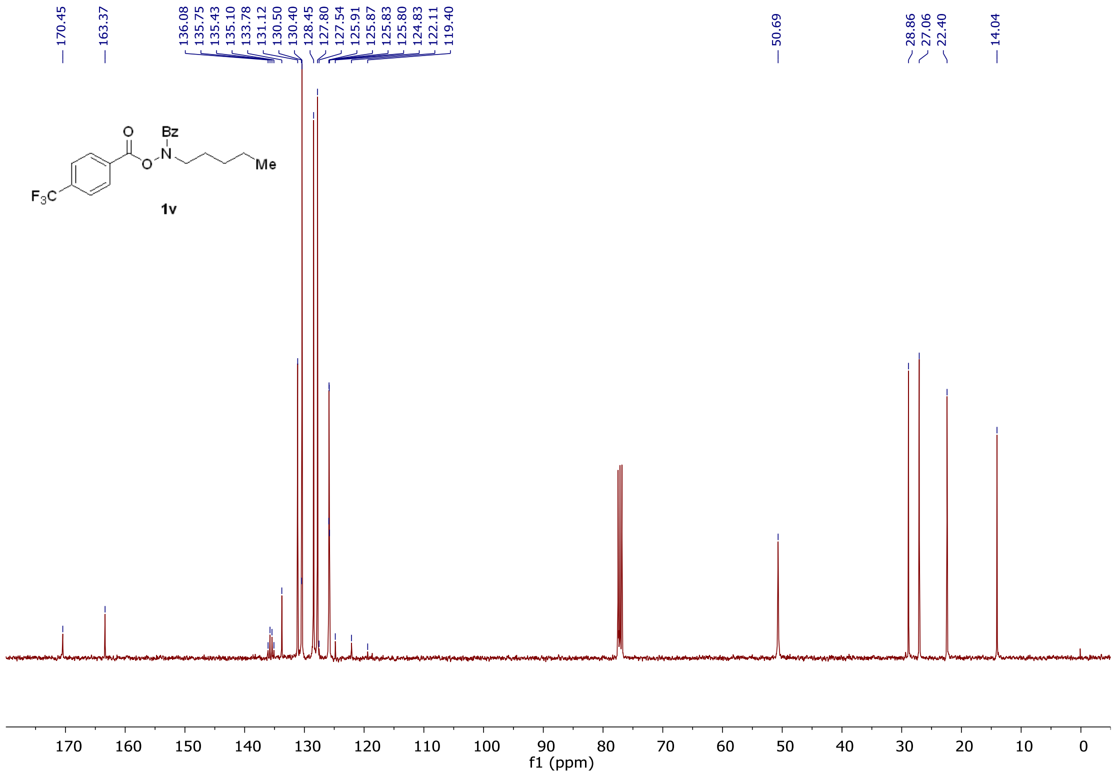

**Supplementary Figure 29.** $^{13}$C NMR spectra for **1v**

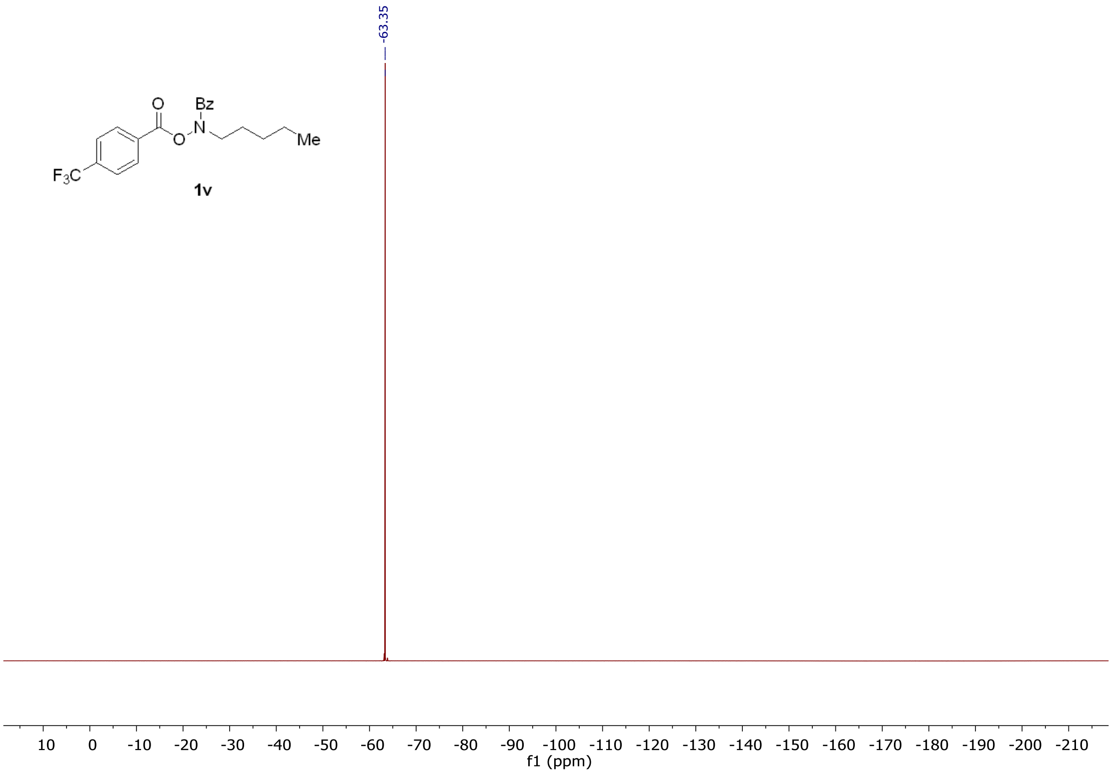


**Supplementary Figure 30.** $^{19}$F NMR spectra for **1v**

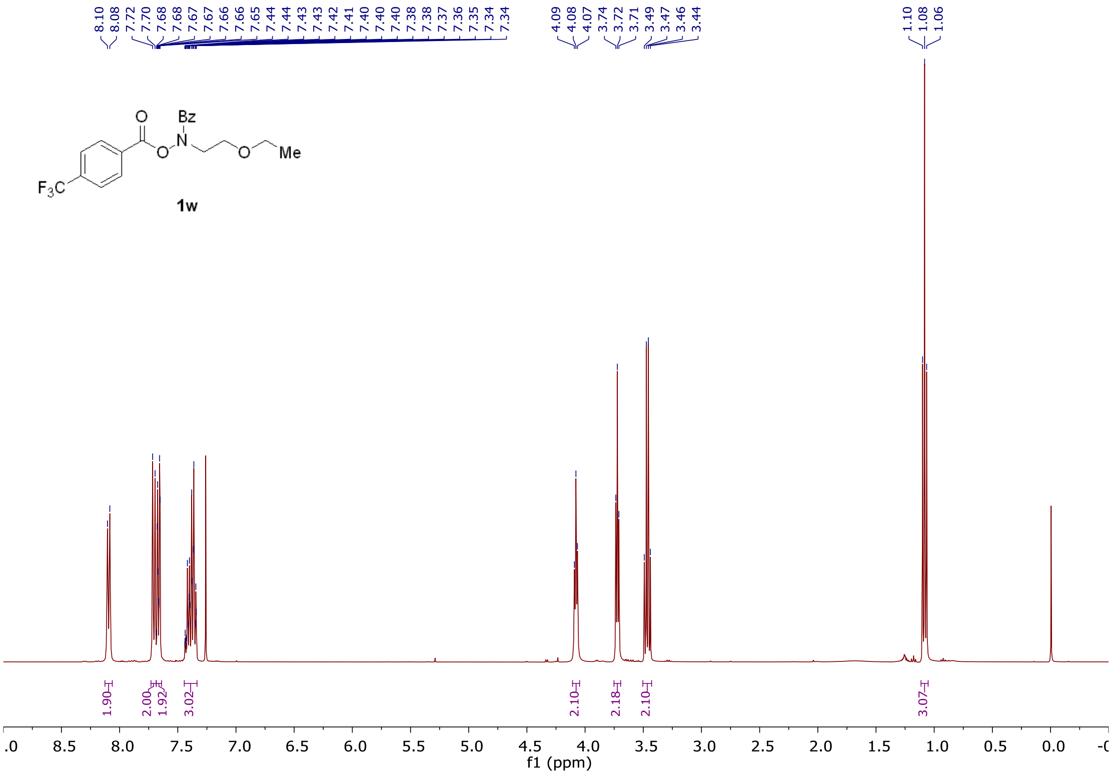


**Supplementary Figure 31.** $^{1}$H NMR spectra for **1w**

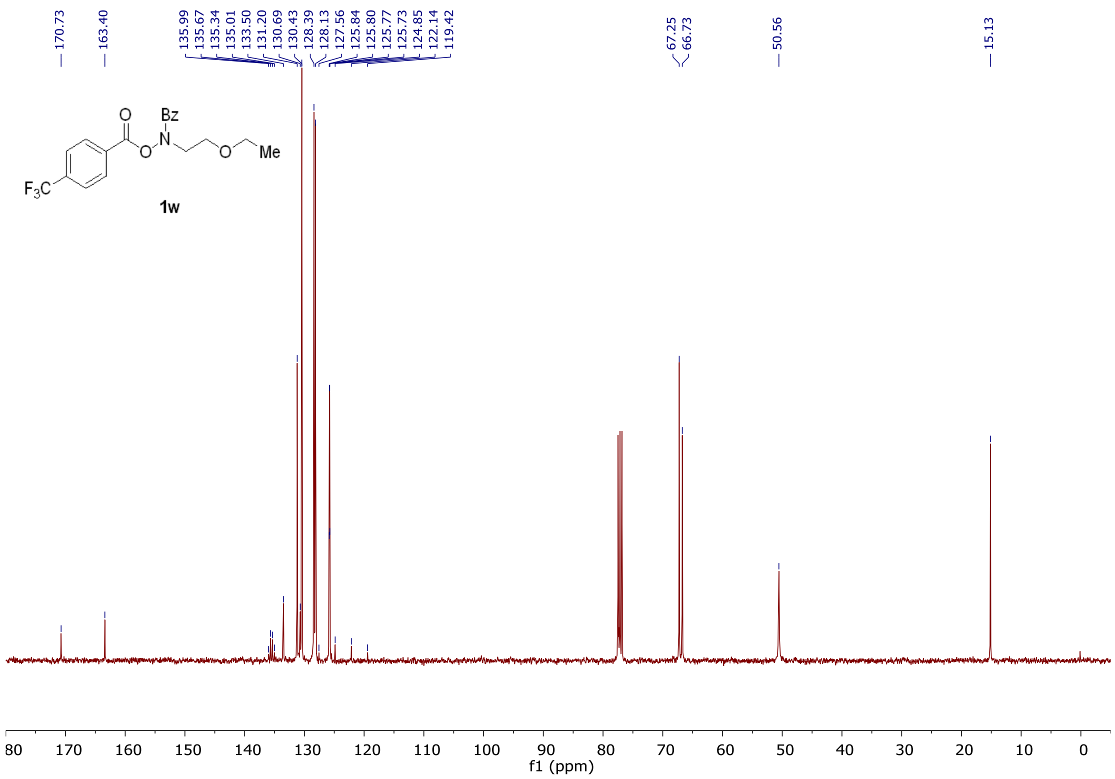


**Supplementary Figure 32.** $^{13}C$ NMR spectra for **1w**

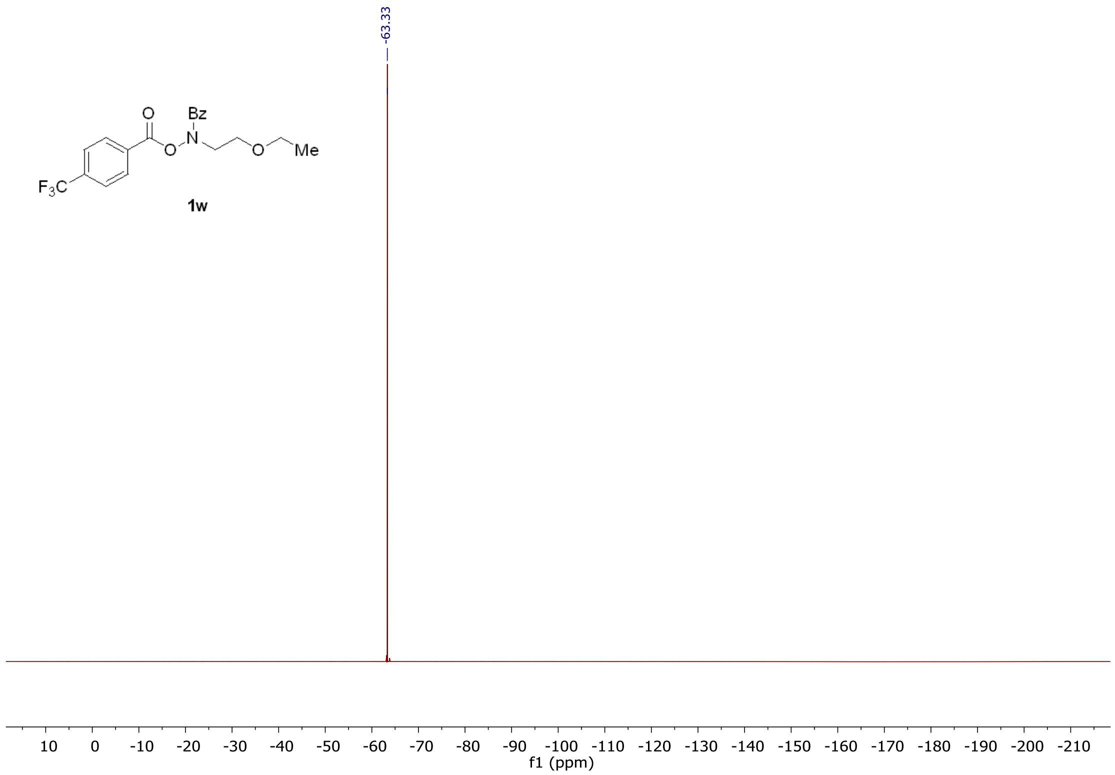


**Supplementary Figure 33.** $^{19}F$ NMR spectra for **1w**

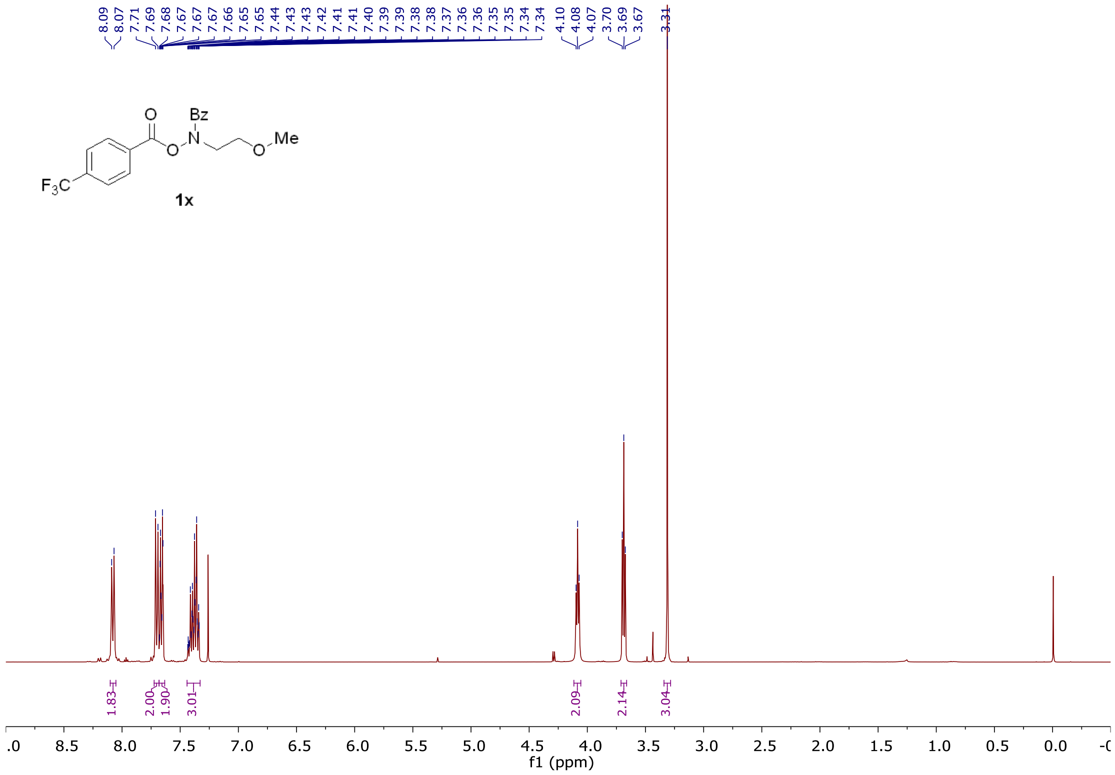


**Supplementary Figure 34.** $^{1}$H NMR spectra for **1x**

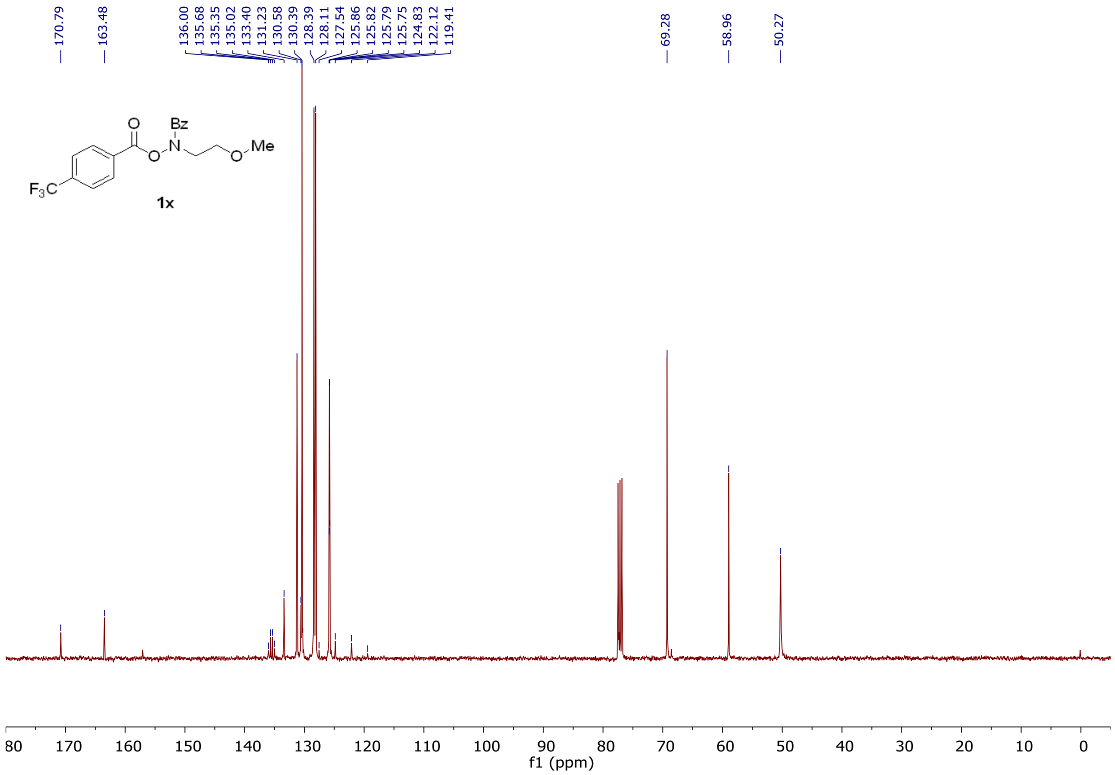


**Supplementary Figure 35.** $^{13}$C NMR spectra for **1x**

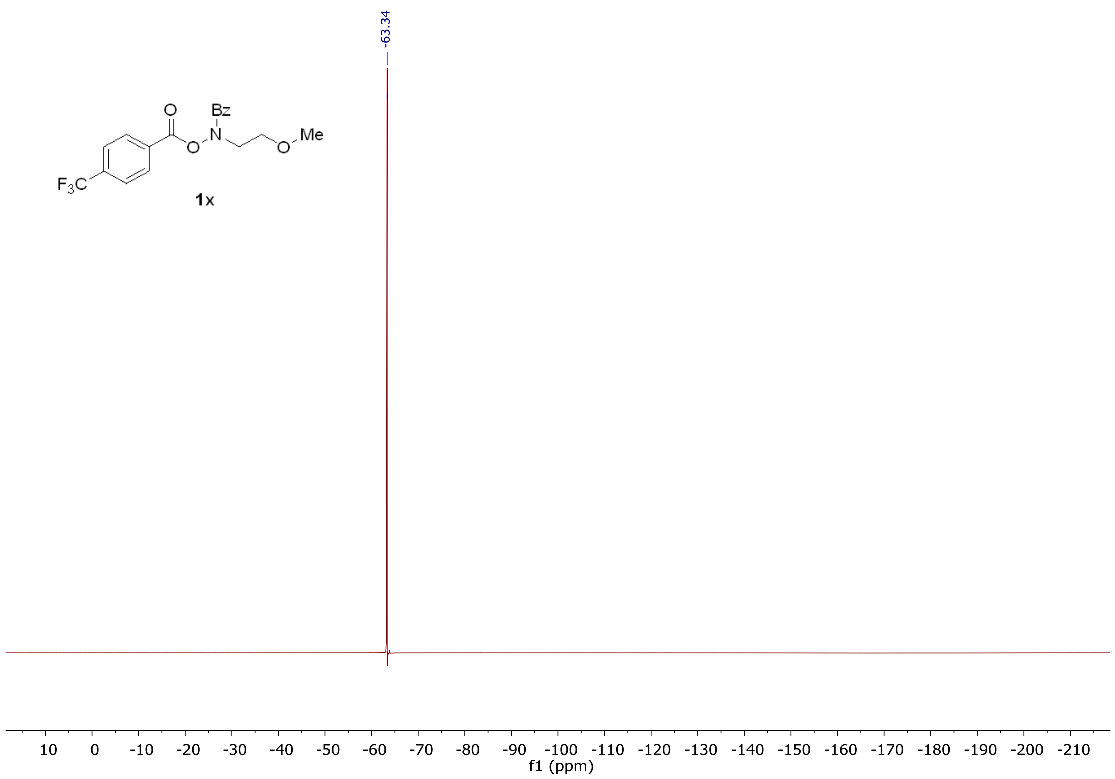

**Supplementary Figure 36.** $^{19}$F NMR spectra for **1x**

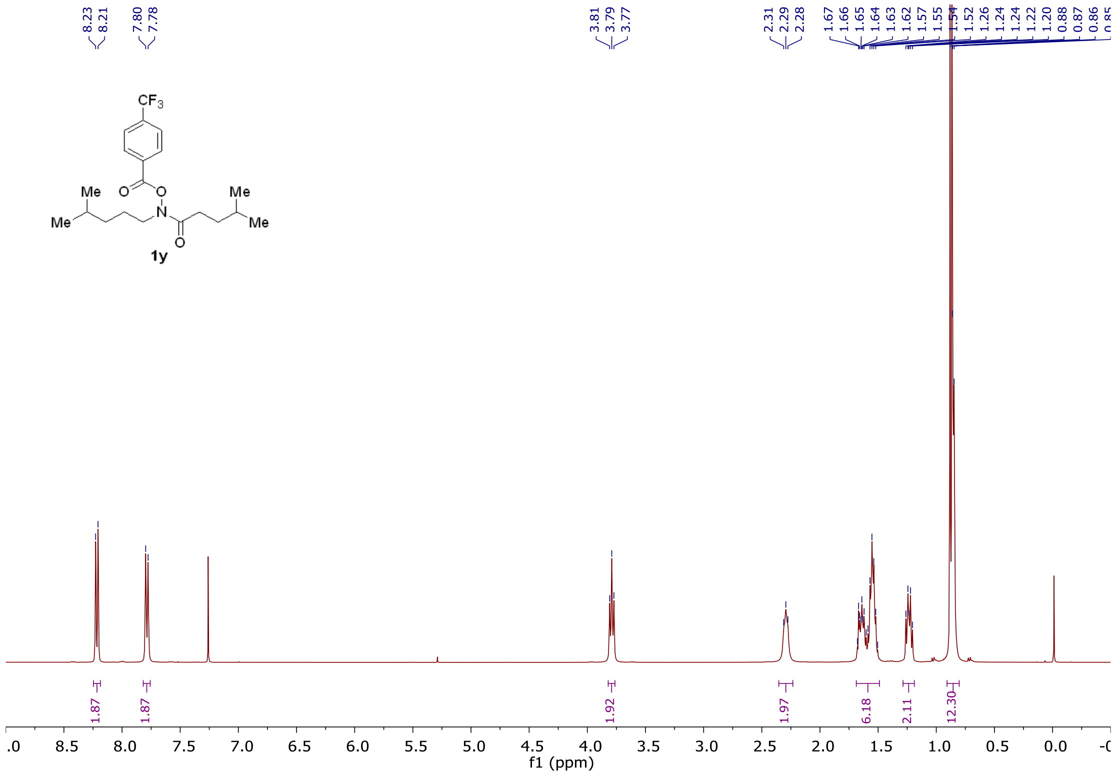

**Supplementary Figure 37.** $^{1}$H NMR spectra for **1y**

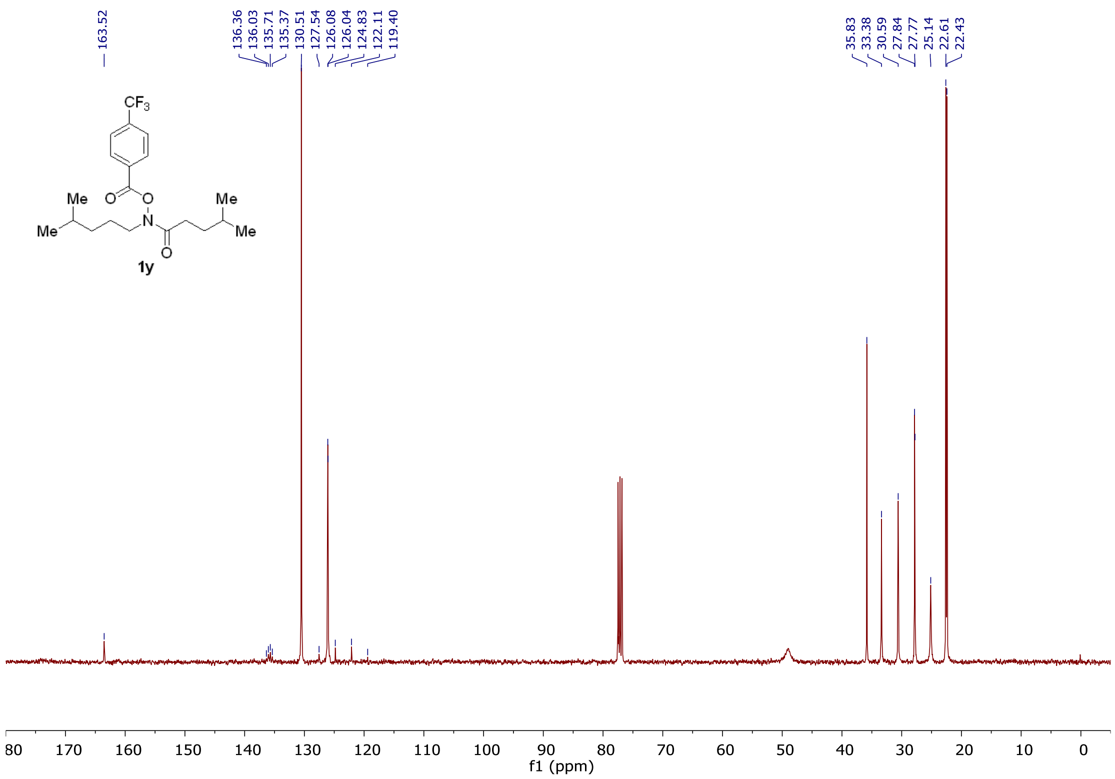


**Supplementary Figure 38.** $^{13}$C NMR spectra for **1y**

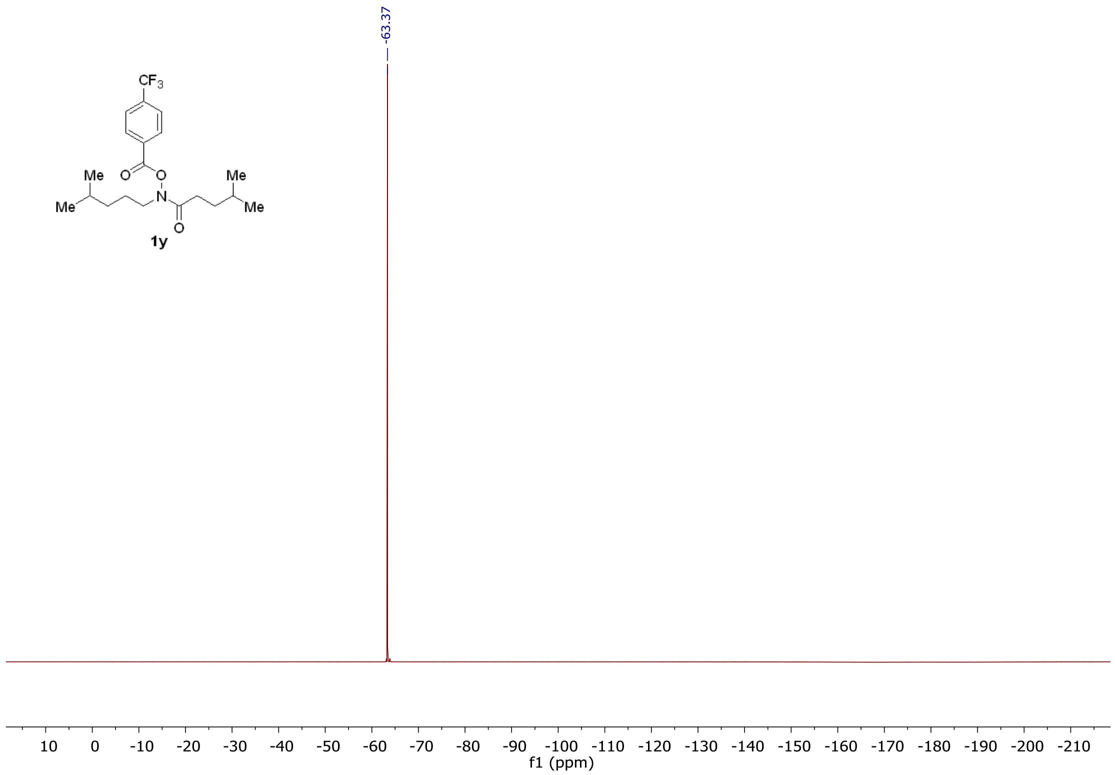


**Supplementary Figure 39.** $^{19}$F NMR spectra for **1y**

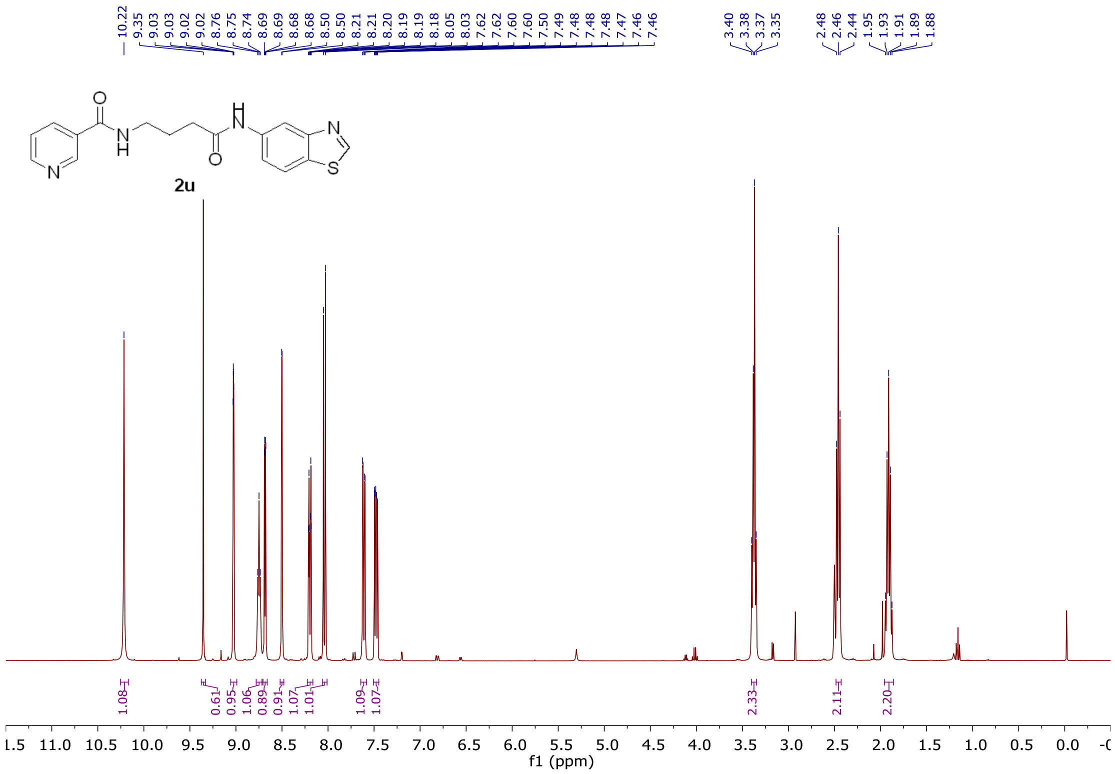


**Supplementary Figure 40.** $^{1}$H NMR spectra for **2u**

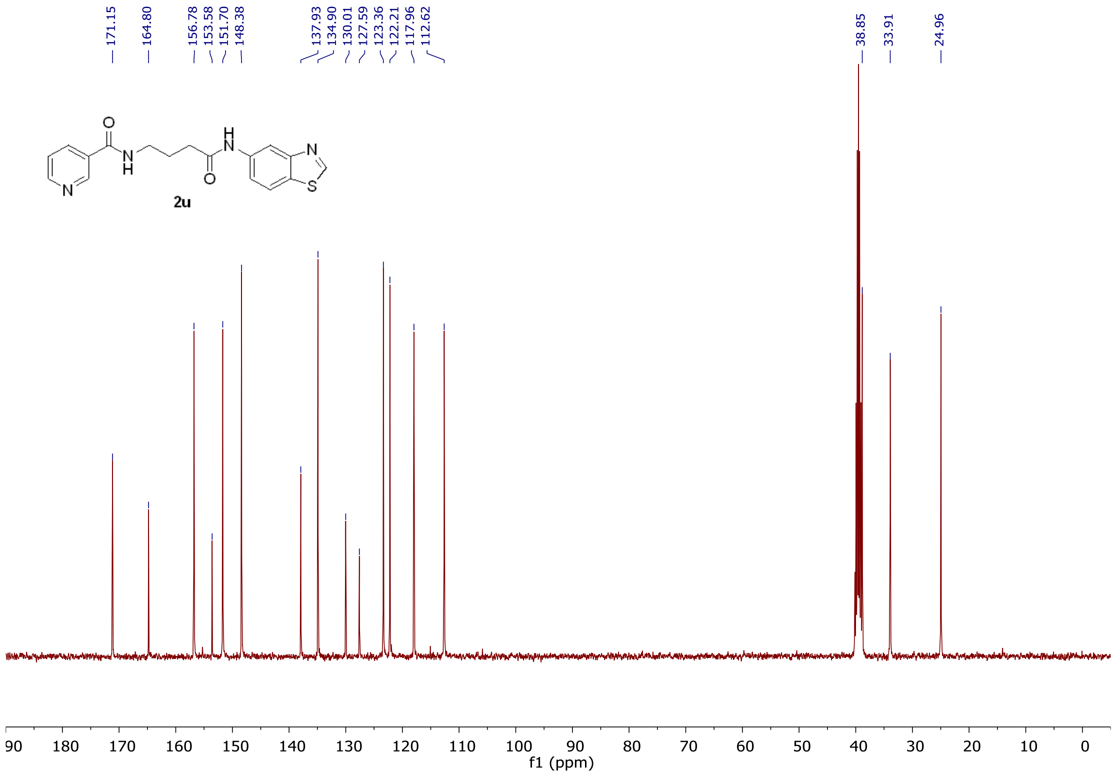


**Supplementary Figure 41.** $^{13}$C NMR spectra for **2u**

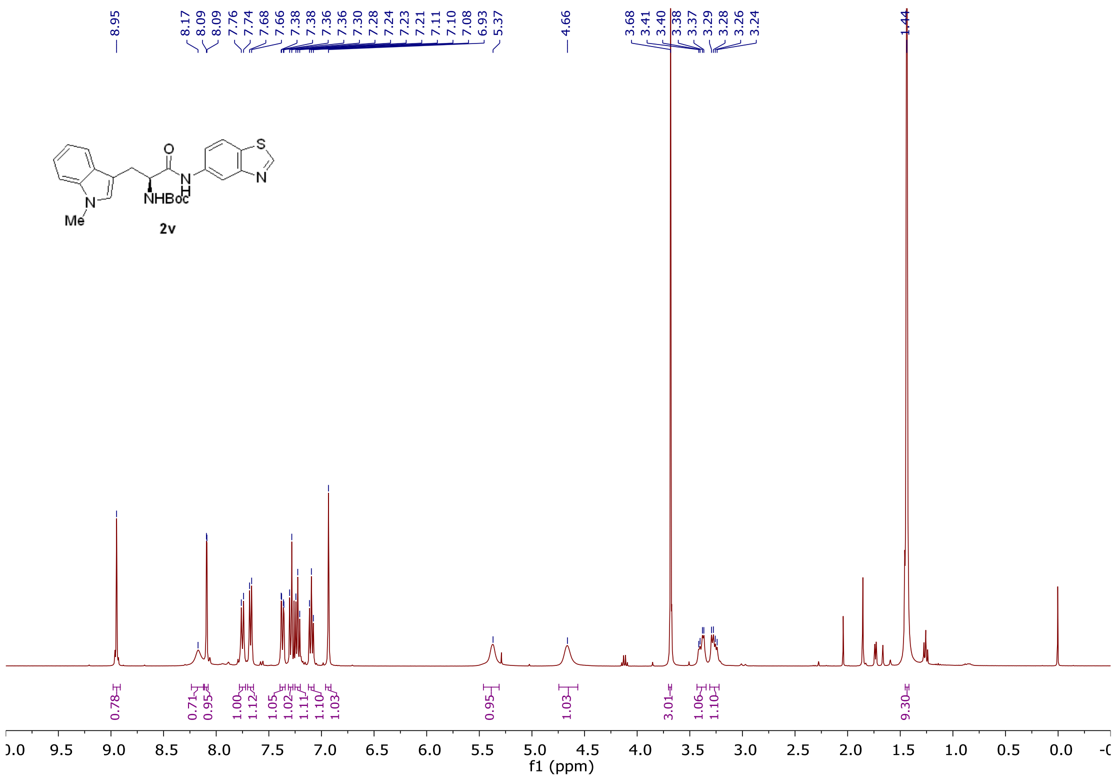

**Supplementary Figure 42.** $^{1}$H NMR spectra for **2v**

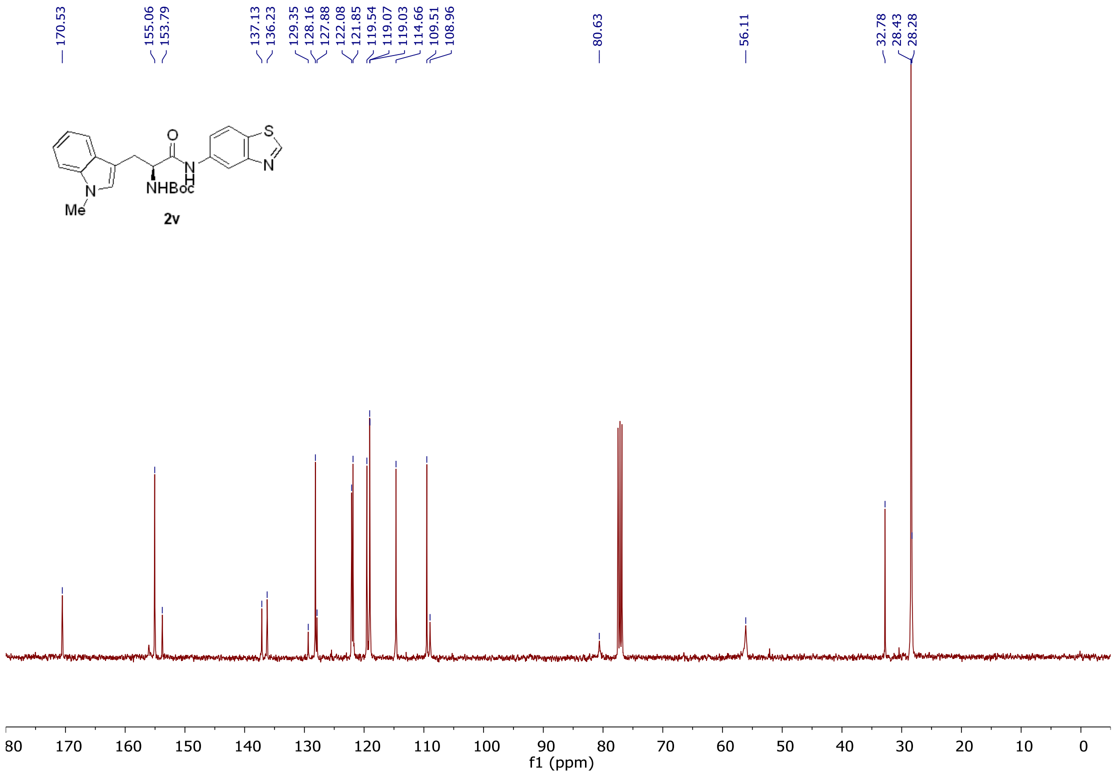

**Supplementary Figure 43.** $^{13}$C NMR spectra for **2v**

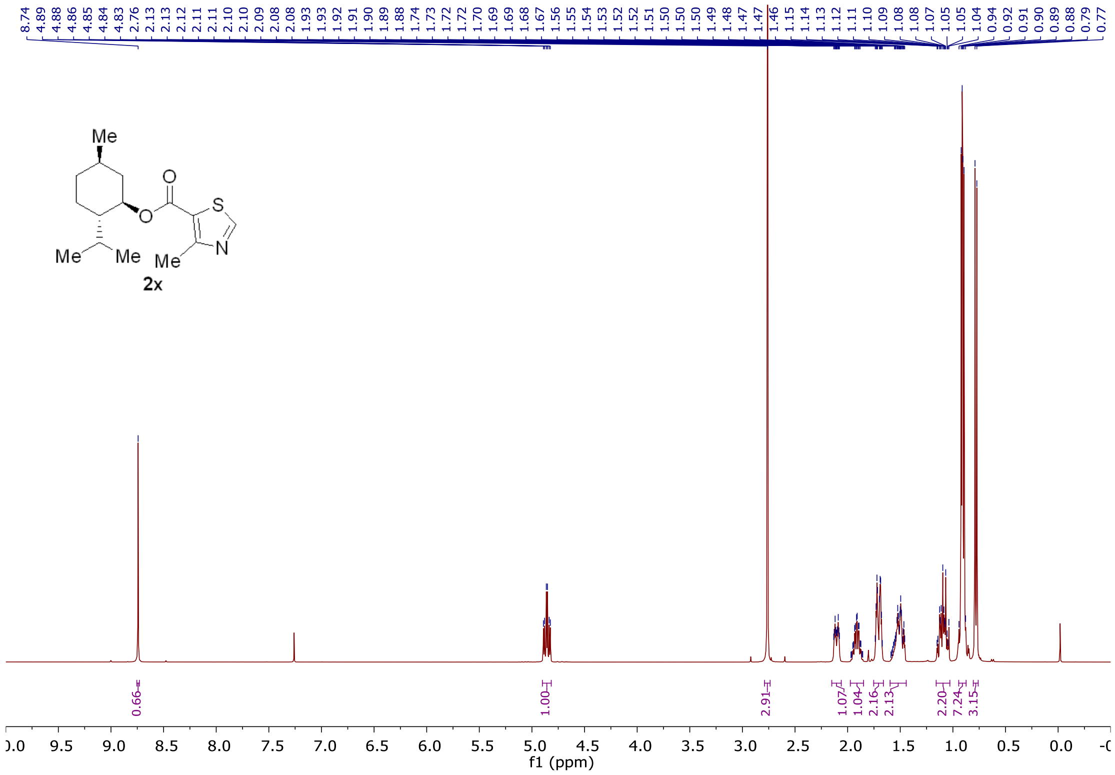


**Supplementary Figure 44.** $^{1}H$ NMR spectra for **2x**

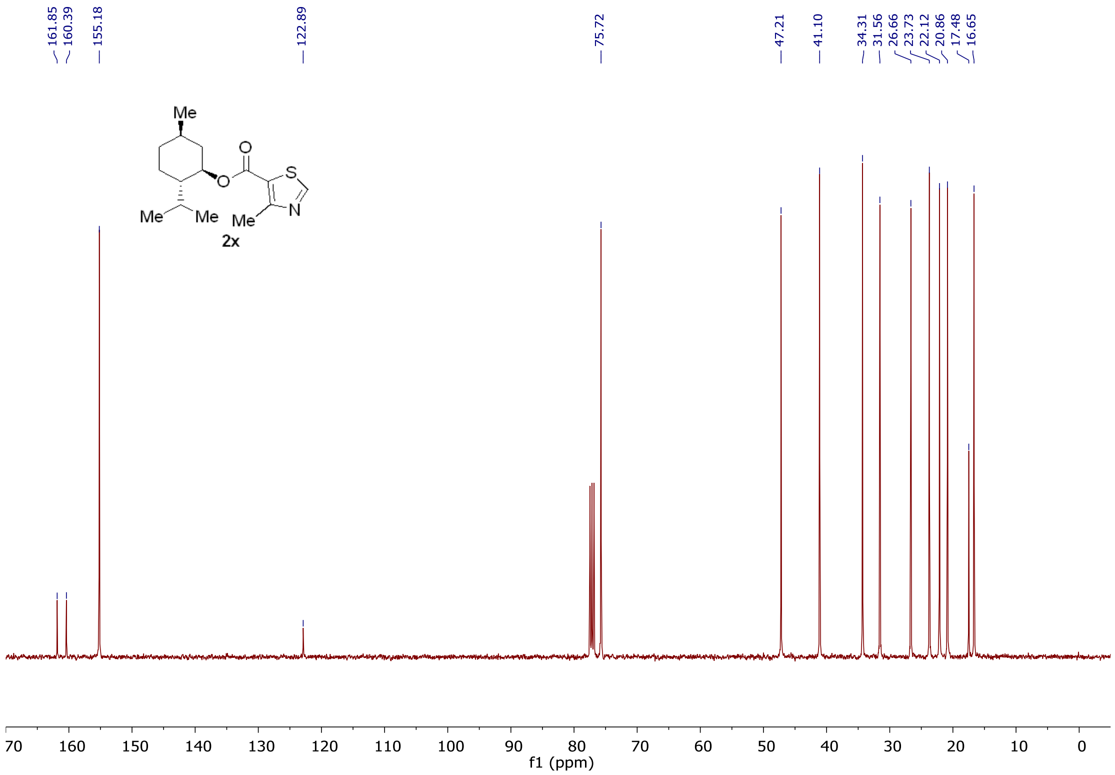


**Supplementary Figure 45.** $^{13}C$ NMR spectra for **2x**

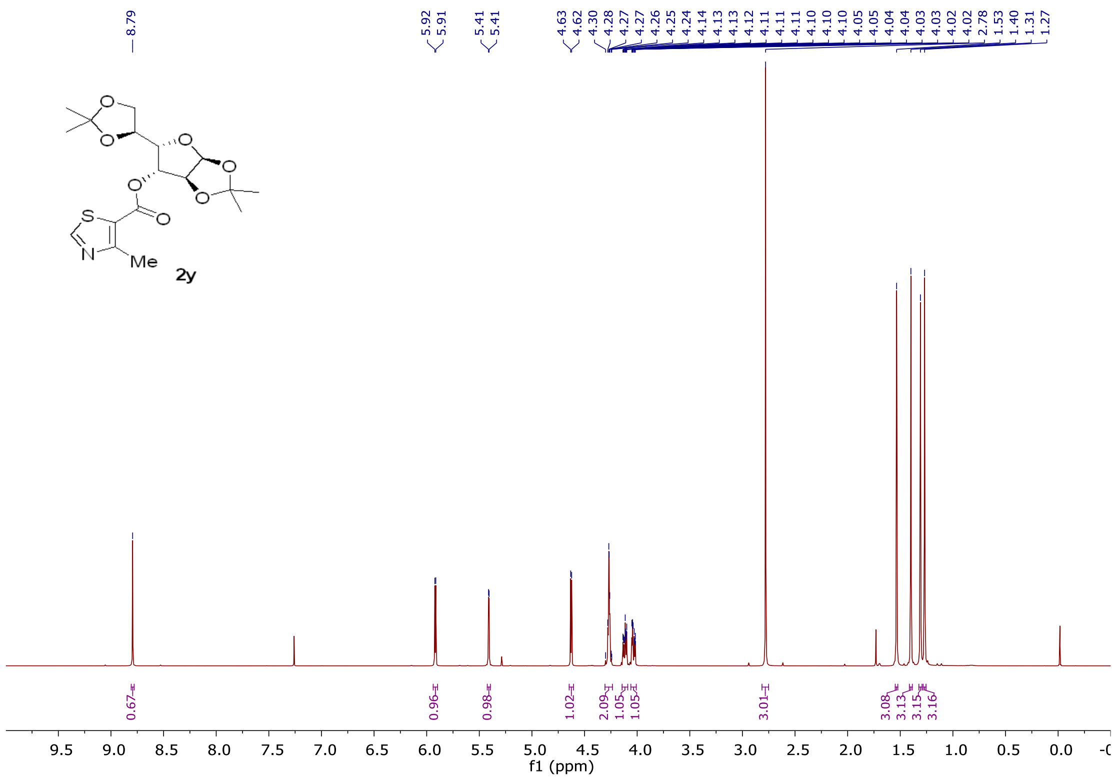

**Supplementary Figure 46.** $^{1}$H NMR spectra for **2y**

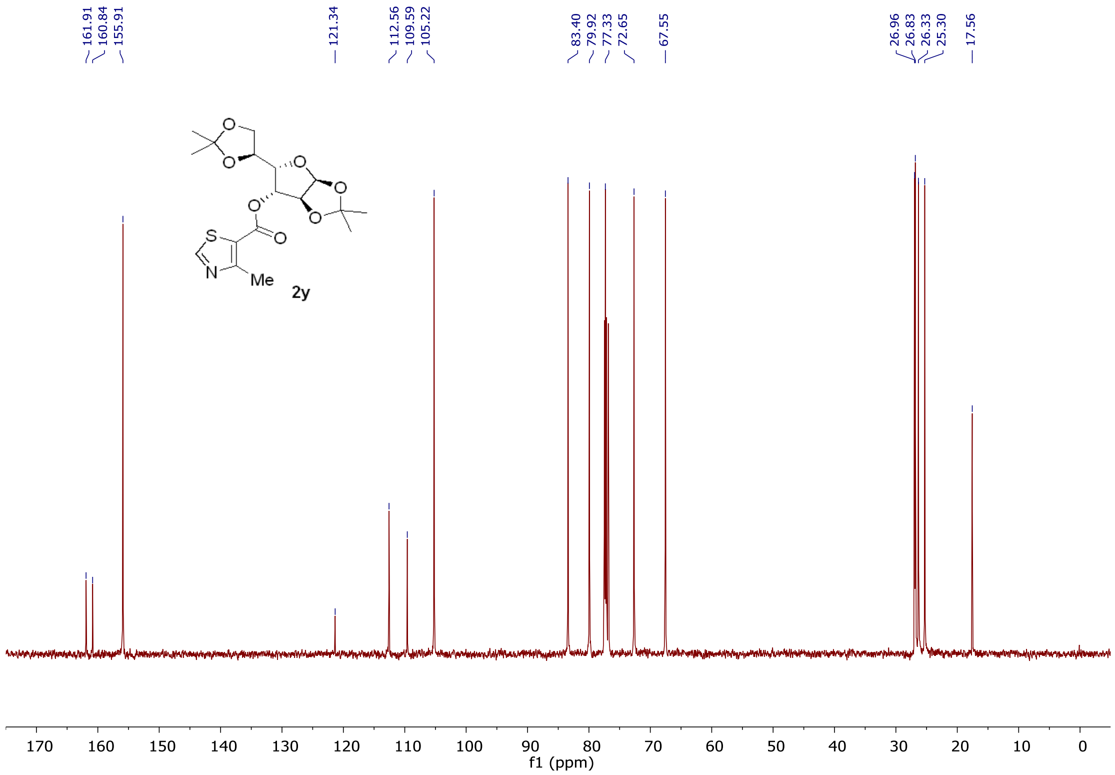

**Supplementary Figure 47.** $^{13}$C NMR spectra for **2y**

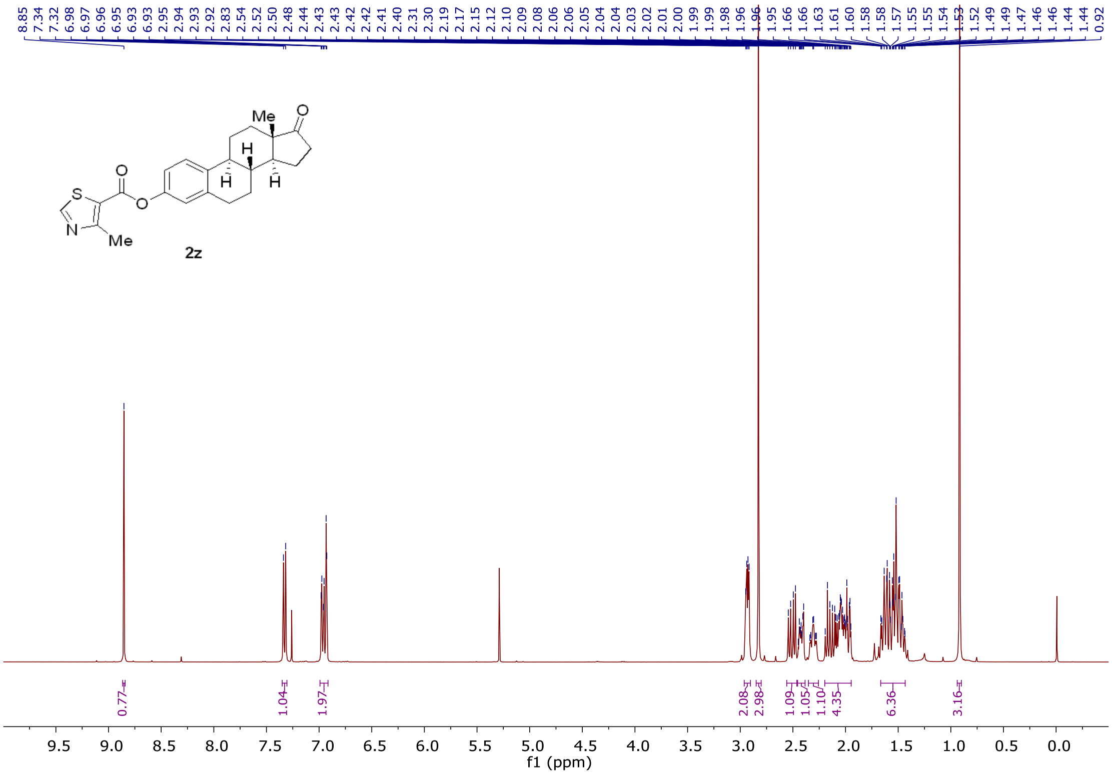


**Supplementary Figure 48.** $^{1}$H NMR spectra for **2z**

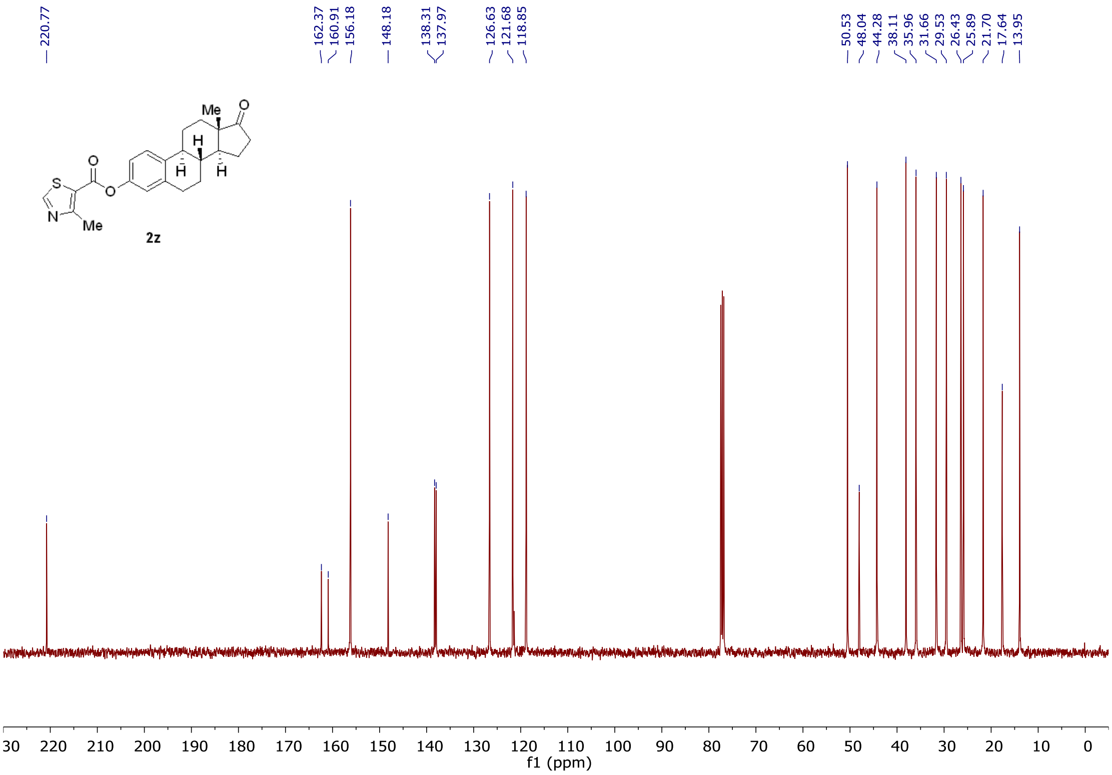


**Supplementary Figure 49.** $^{13}$C NMR spectra for **2z**

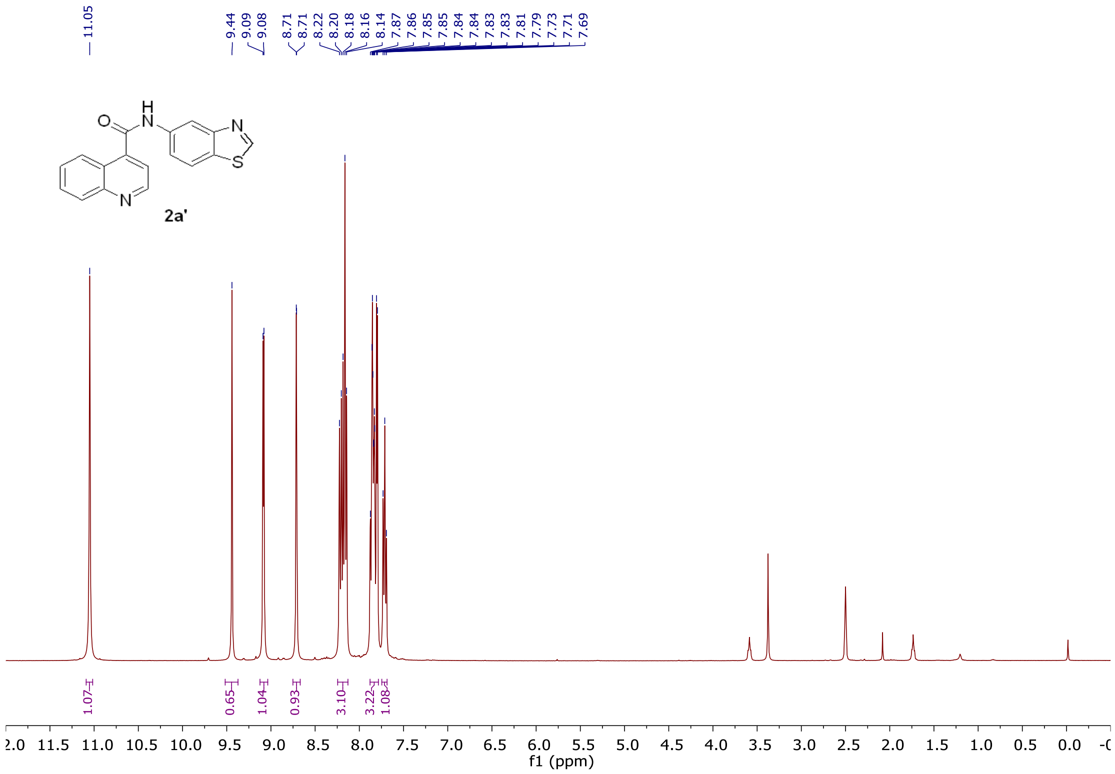

**Supplementary Figure 50.** $^{1}$H NMR spectra for **2a’**

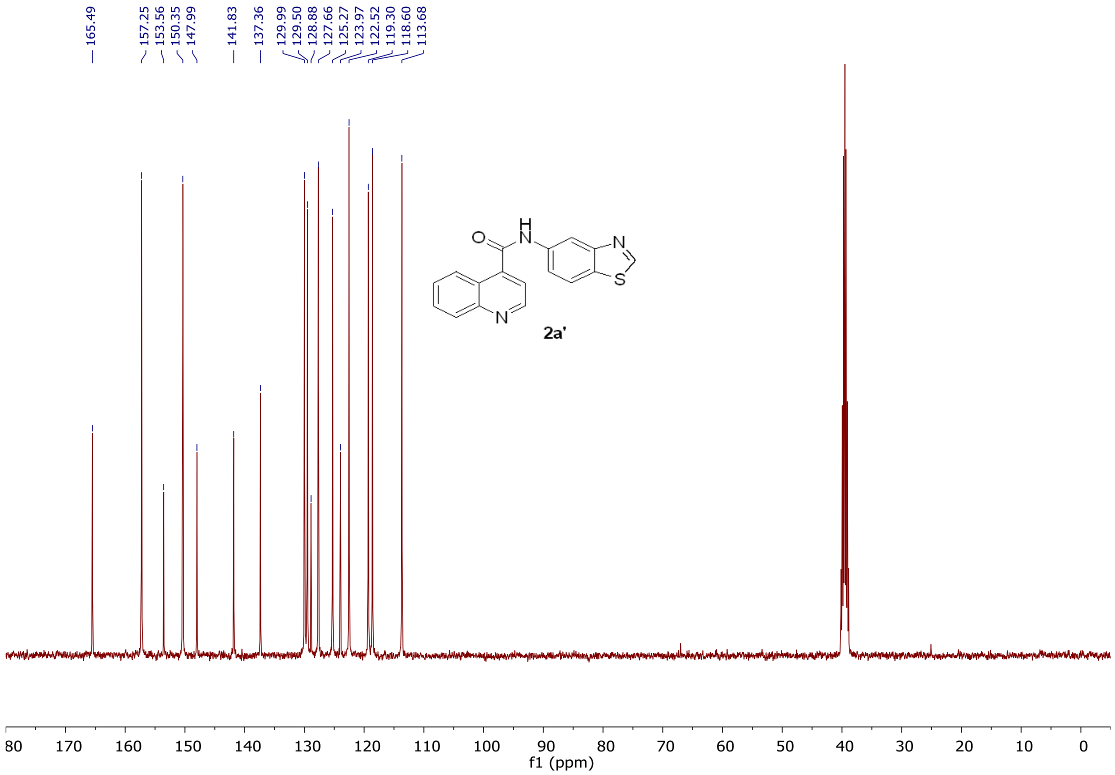

**Supplementary Figure 51.** $^{13}$C NMR spectra for **2a’**

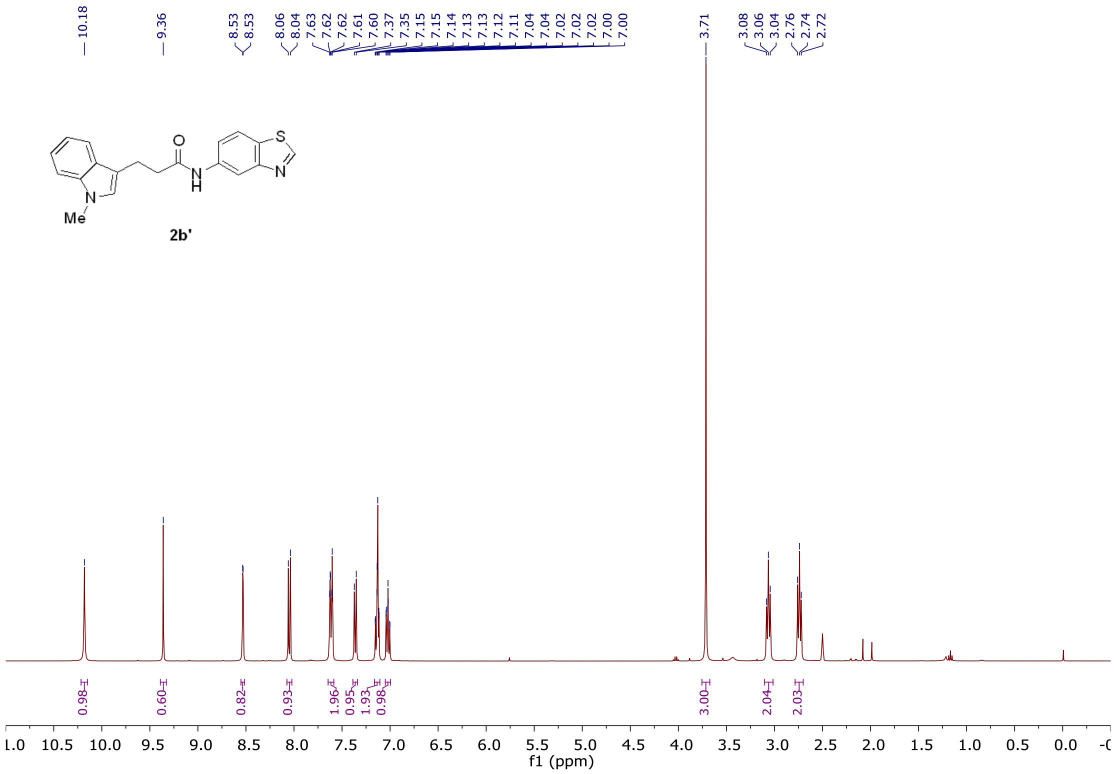


**Supplementary Figure 52.** $^{1}$H NMR spectra for **2b’**

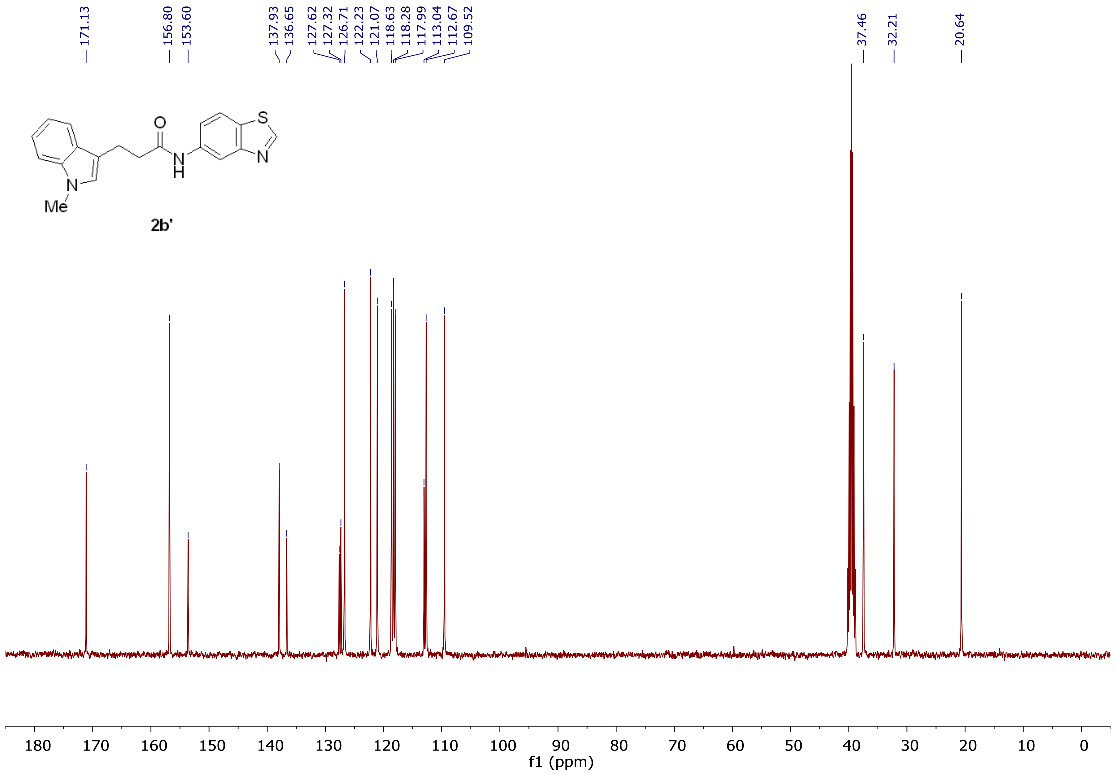


**Supplementary Figure 53.** $^{13}$C NMR spectra for **2b’**

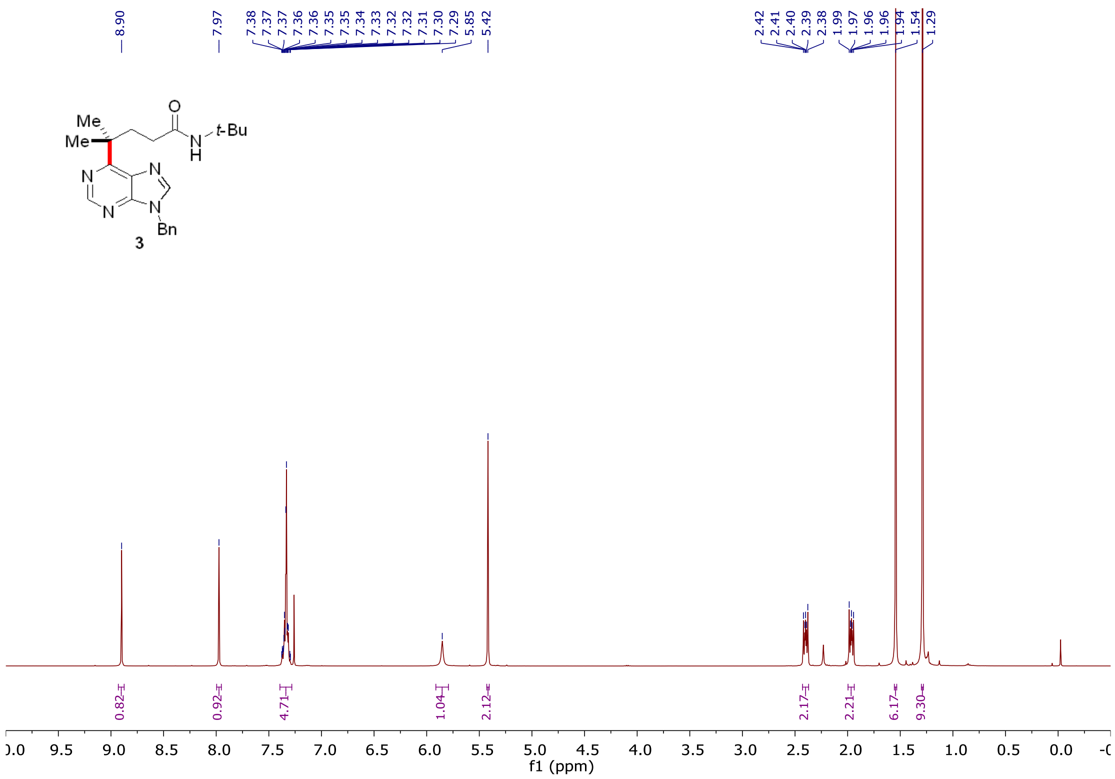

**Supplementary Figure 54.** $^{1}$H NMR spectra for **3**

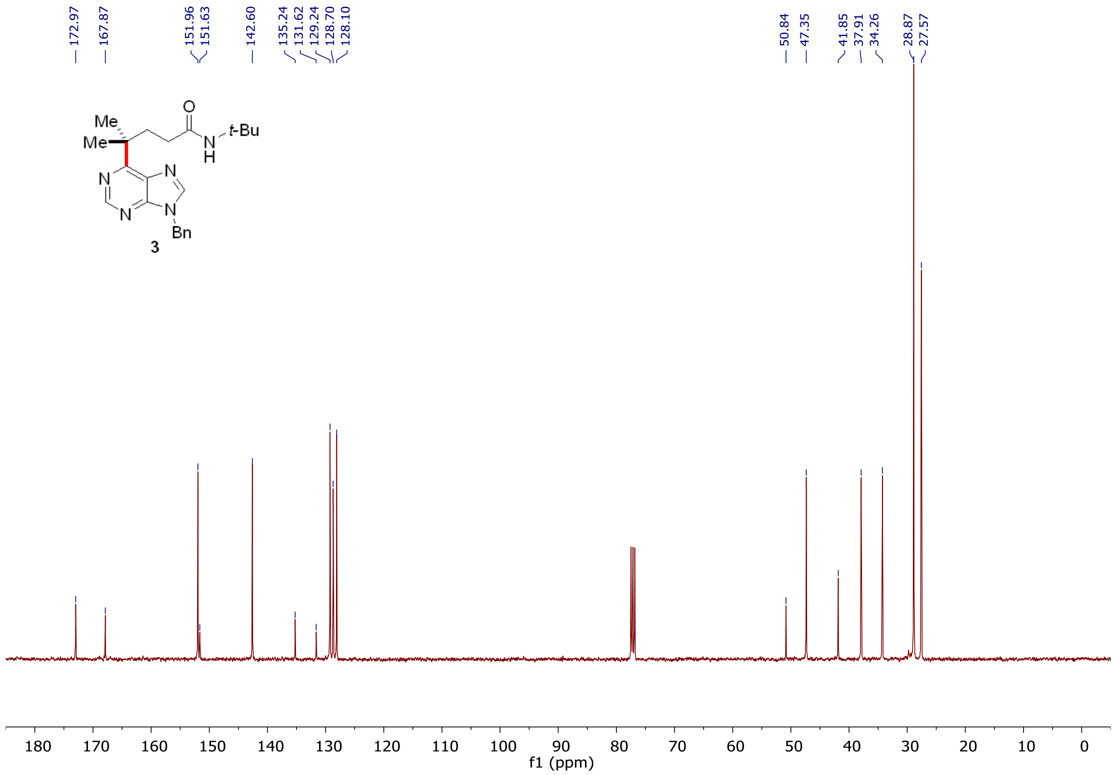

**Supplementary Figure 55.** $^{13}$C NMR spectra for **3**

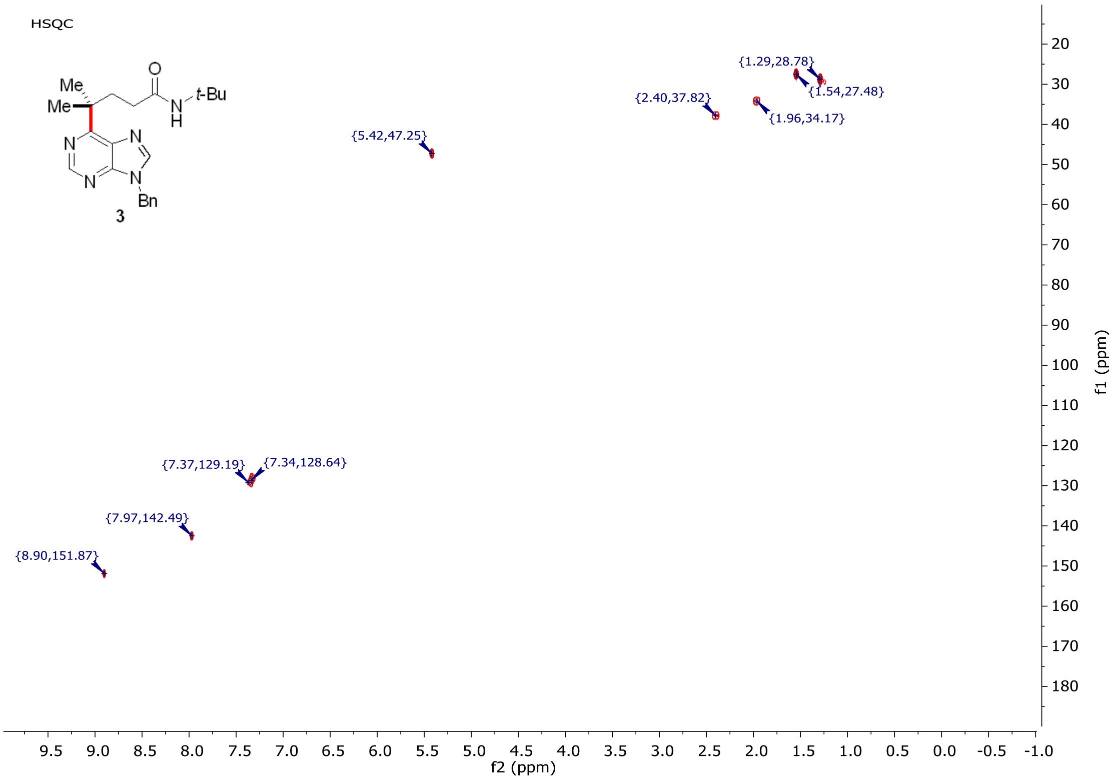


**Supplementary Figure 56.** HSQC NMR spectra for **3**

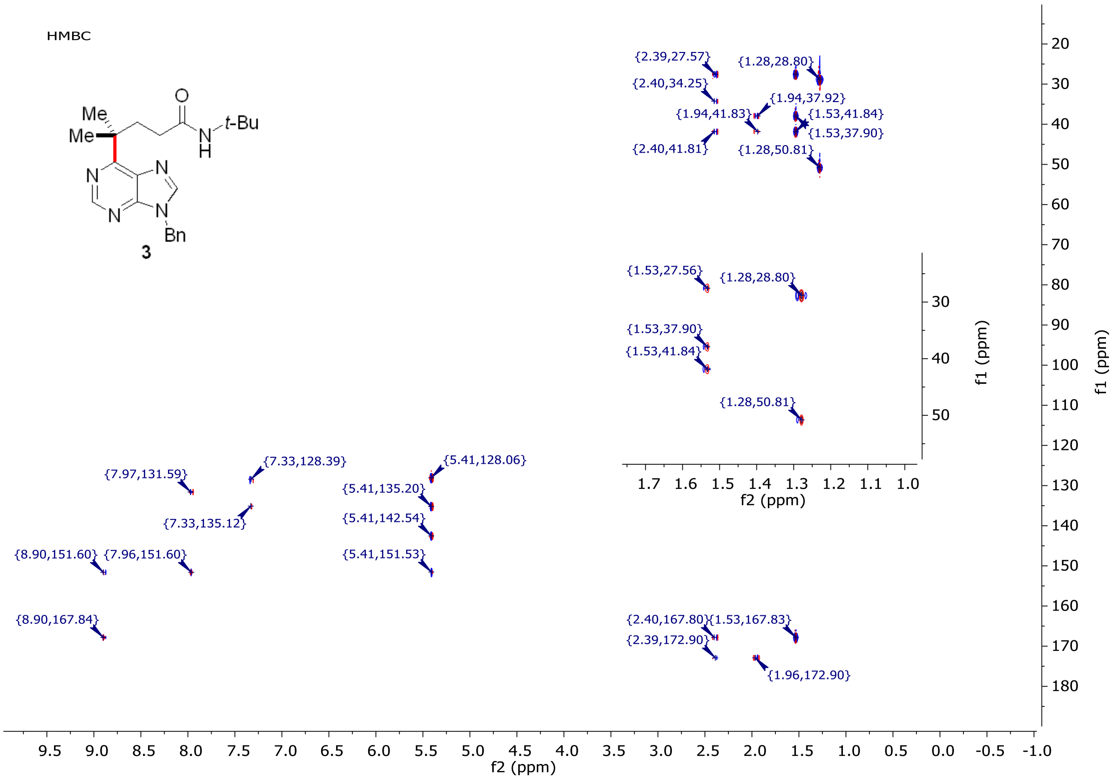


**Supplementary Figure 57.** HMBC NMR spectra for **3**

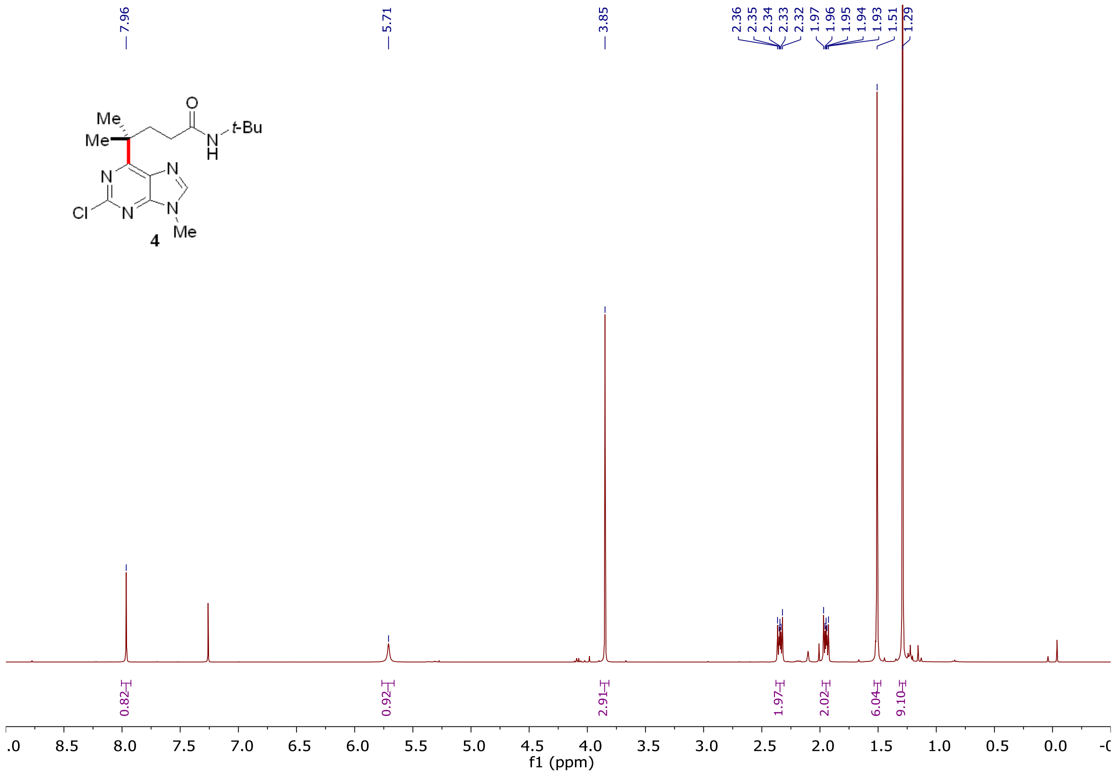

**Supplementary Figure 58.** $^{1}$H NMR spectra for **4**

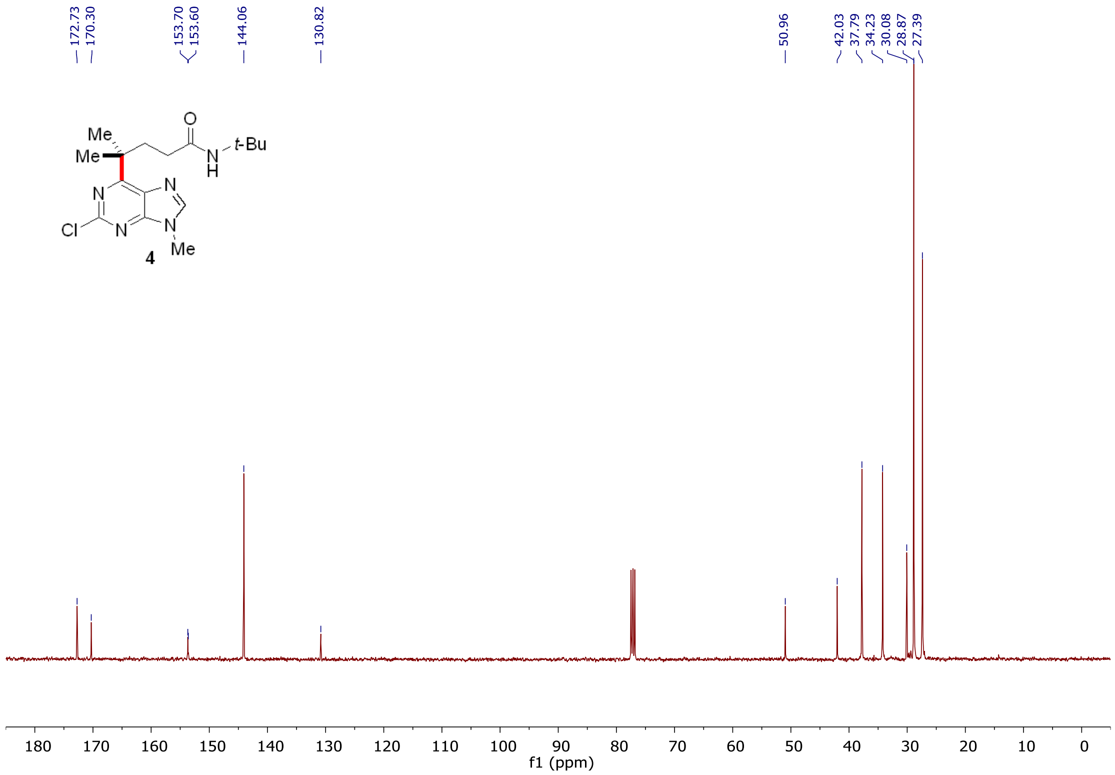

**Supplementary Figure 59.** $^{13}$C NMR spectra for **4**

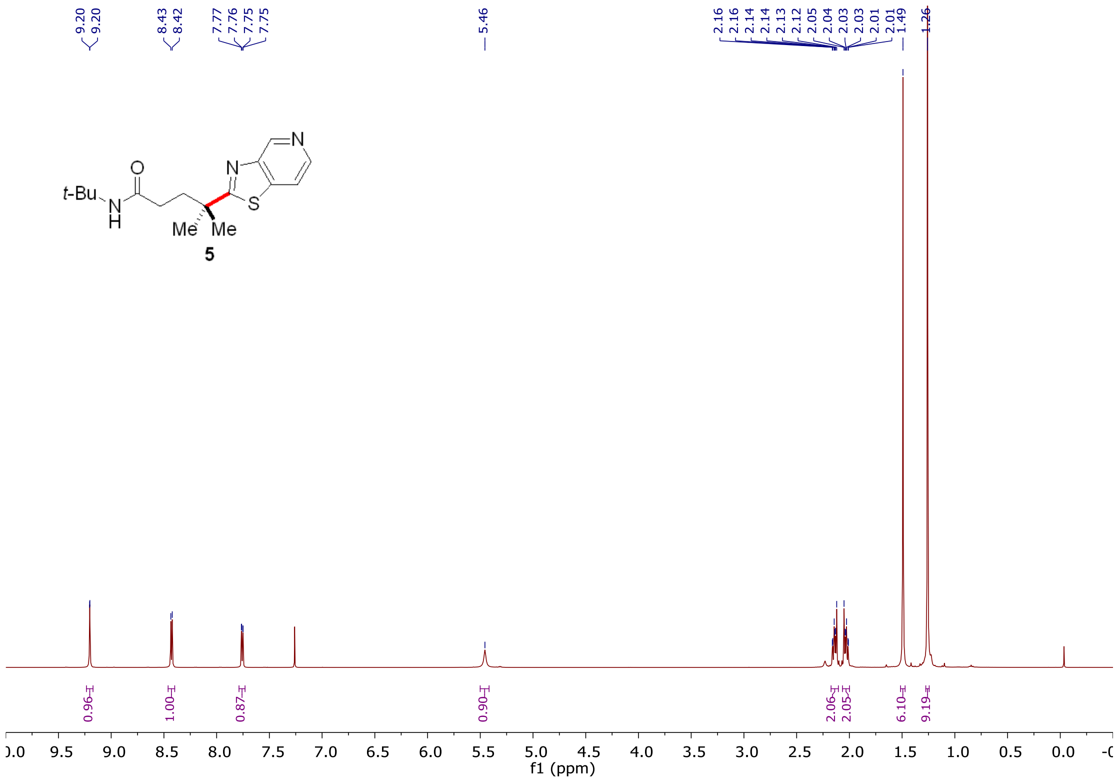


**Supplementary Figure 60.** $^{1}$H NMR spectra for **5**

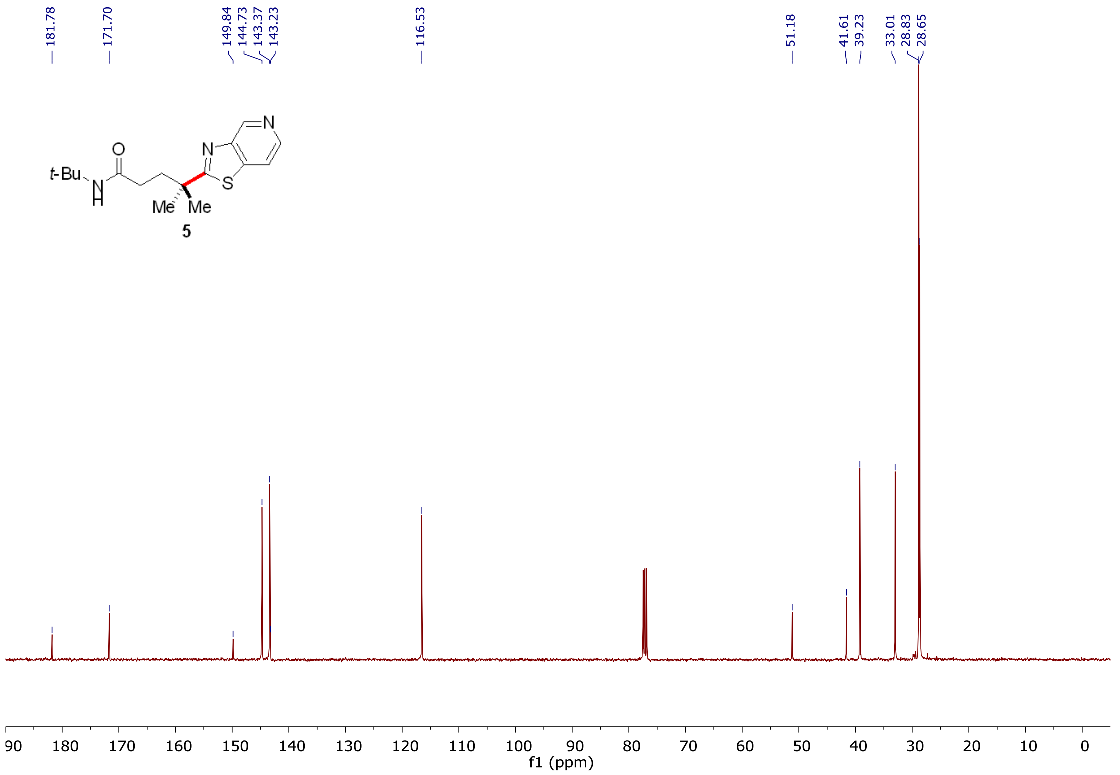


**Supplementary Figure 61.** $^{13}$C NMR spectra for **5**

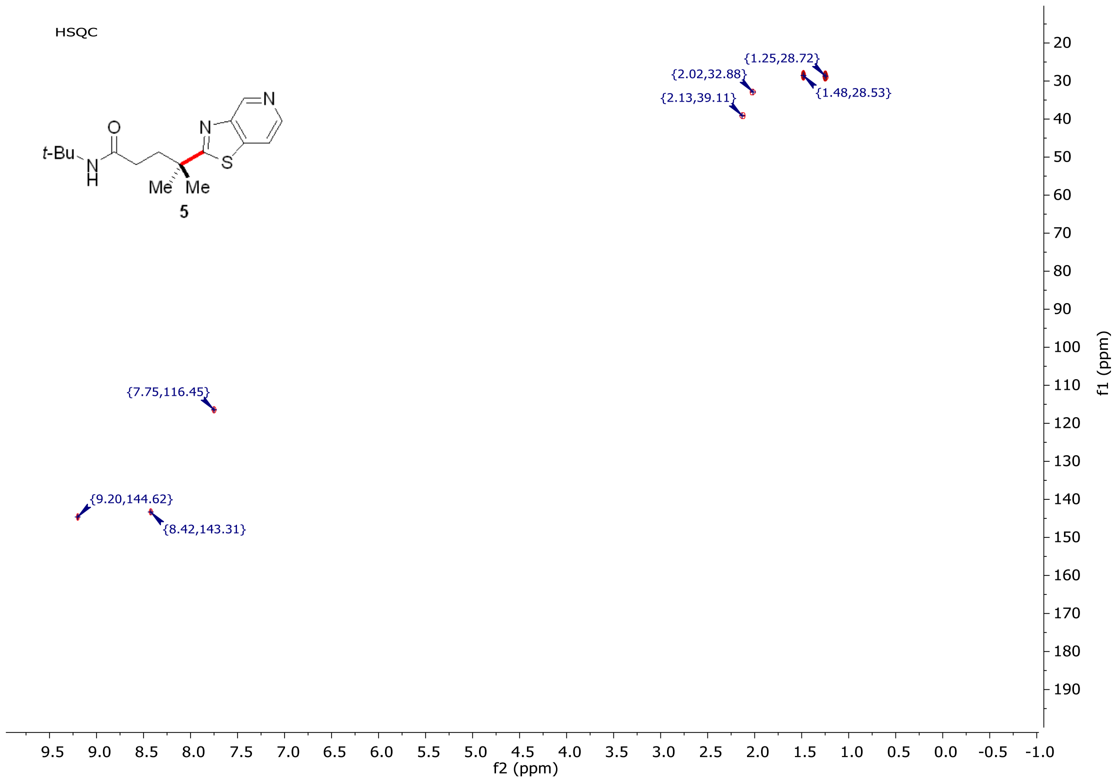

**Supplementary Figure 62.** HSQC NMR spectra for **5**

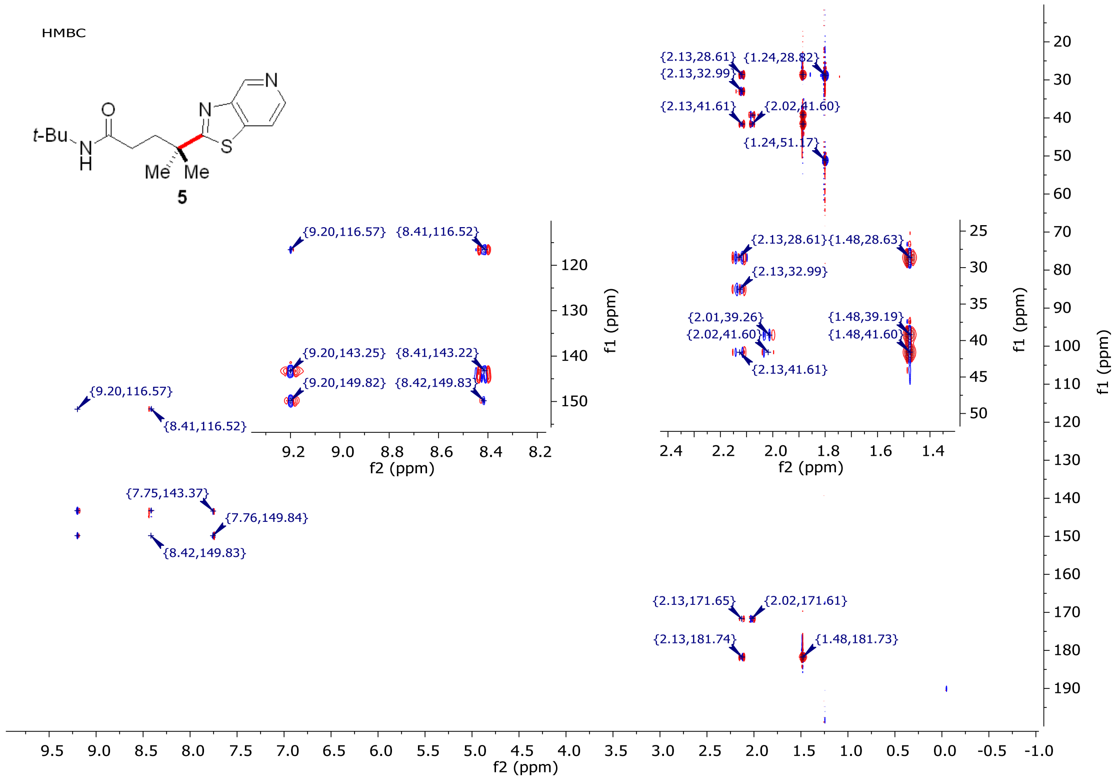

**Supplementary Figure 63.** HMBC NMR spectra for **5**

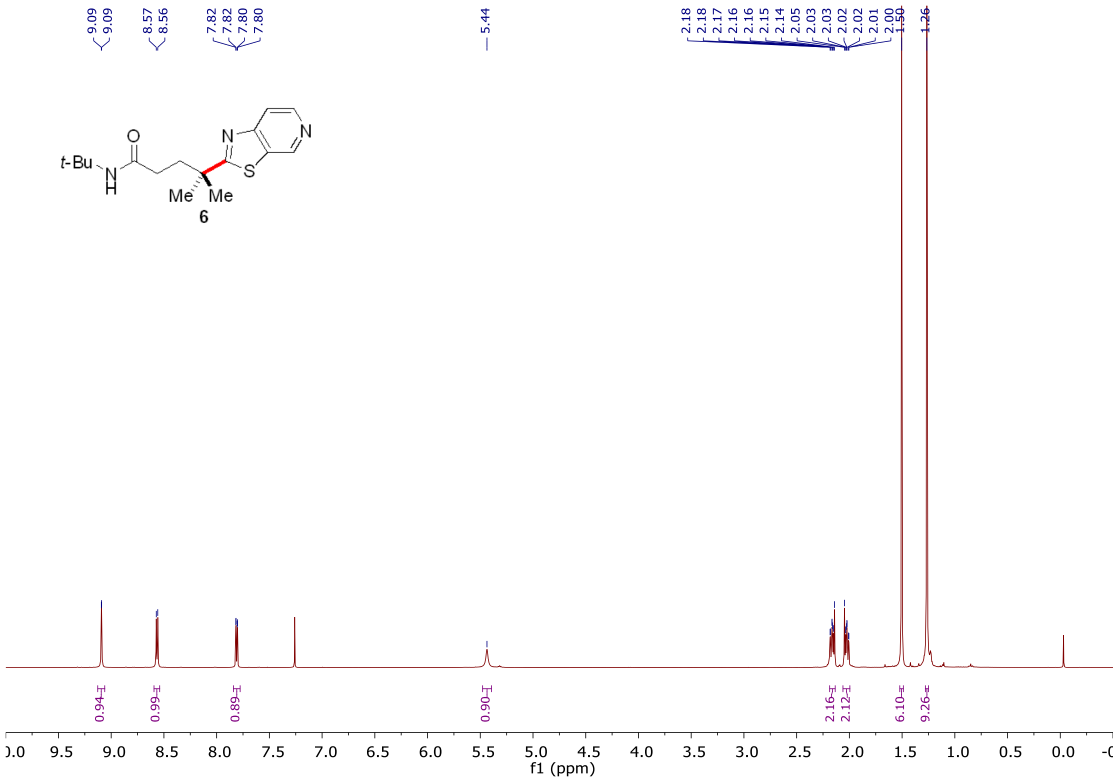


**Supplementary Figure 64.** $^{1}$H NMR spectra for **6**

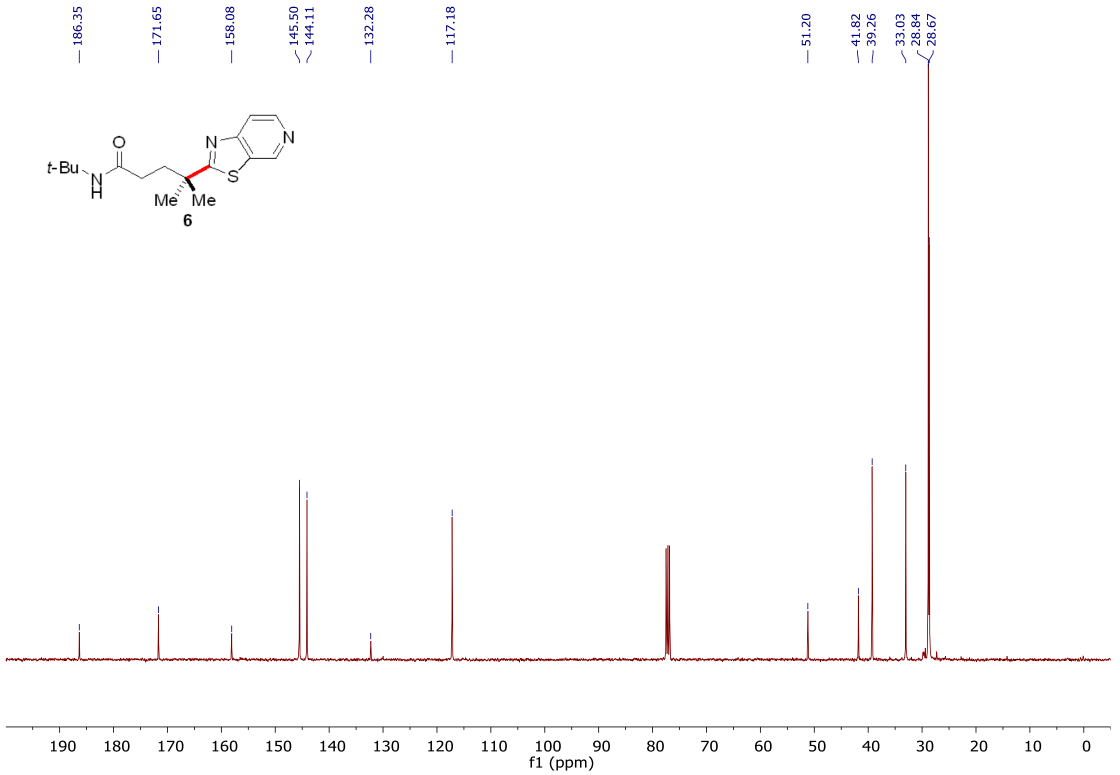


**Supplementary Figure 65.** $^{13}$C NMR spectra for **6**

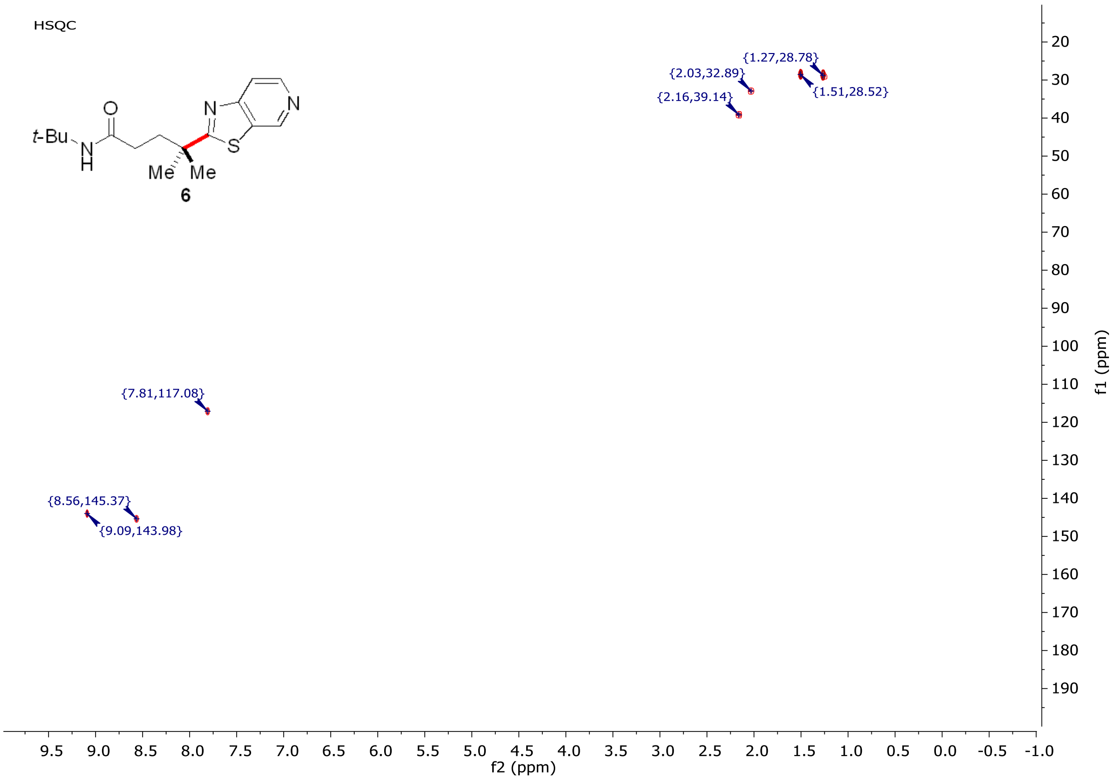


**Supplementary Figure 66.** HSQC NMR spectra for **6**

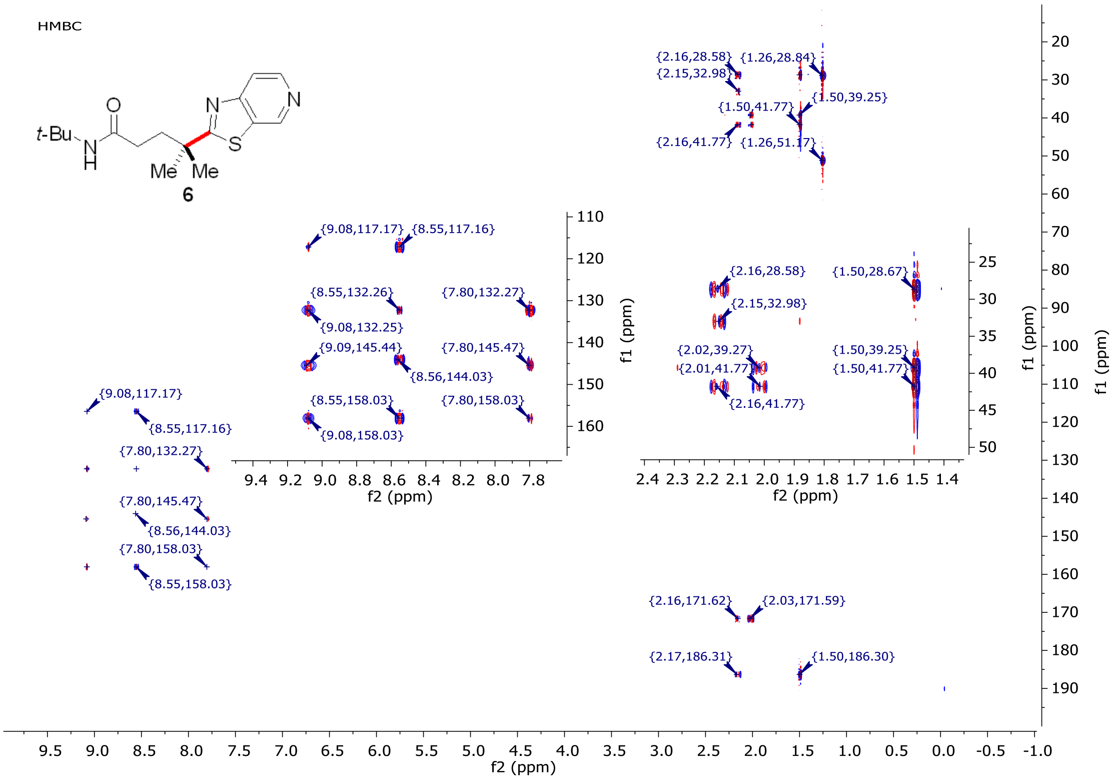


**Supplementary Figure 67.** HMBC NMR spectra for **6**

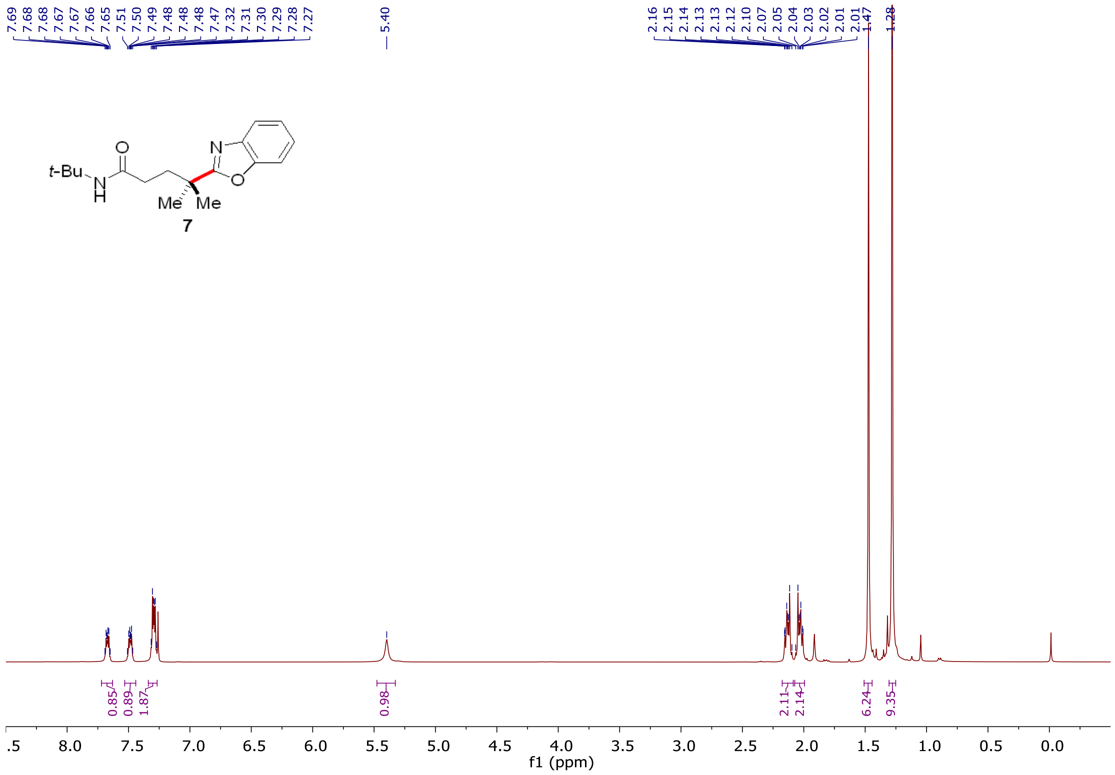


**Supplementary Figure 68.** $^{1}$H NMR spectra for **7**

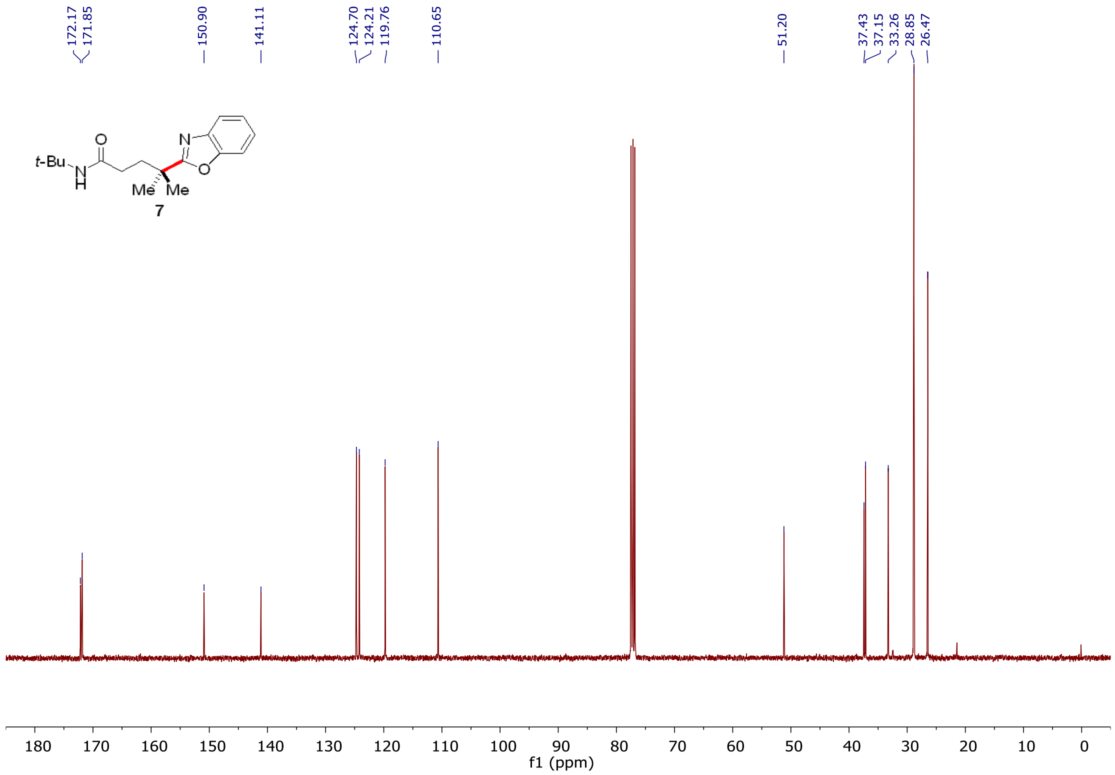


**Supplementary Figure 69.** $^{13}$C NMR spectra for **7**

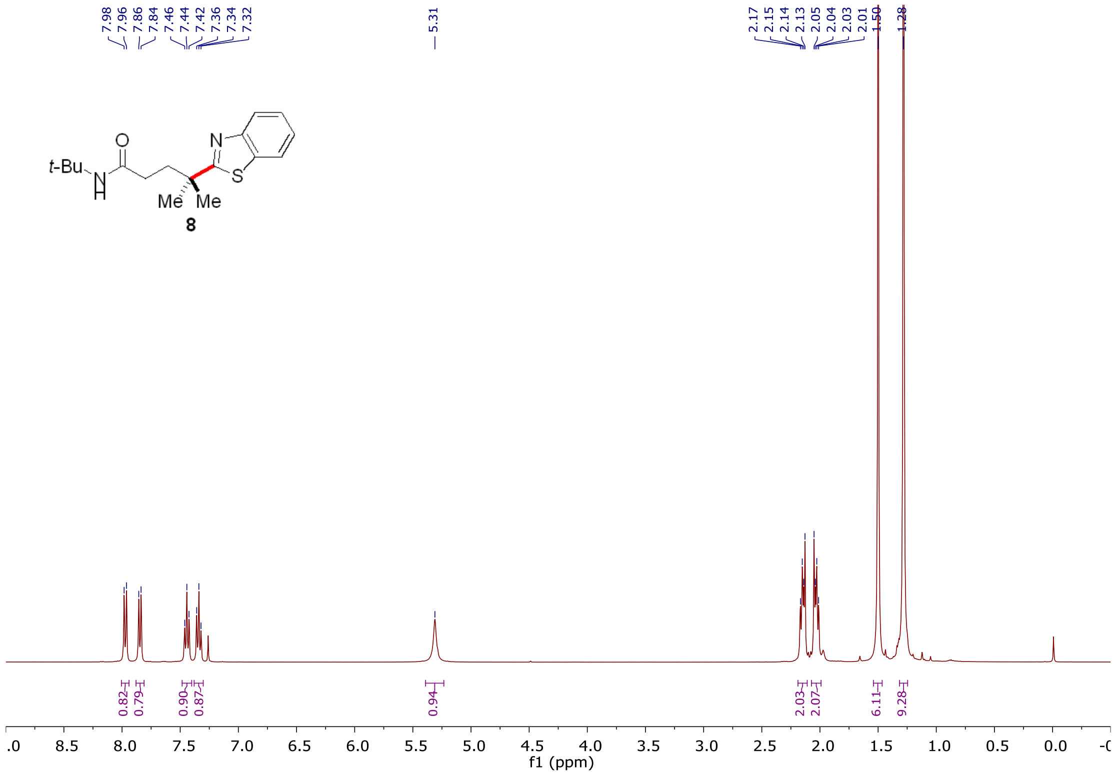


**Supplementary Figure 70.** $^{1}H$ NMR spectra for **8**

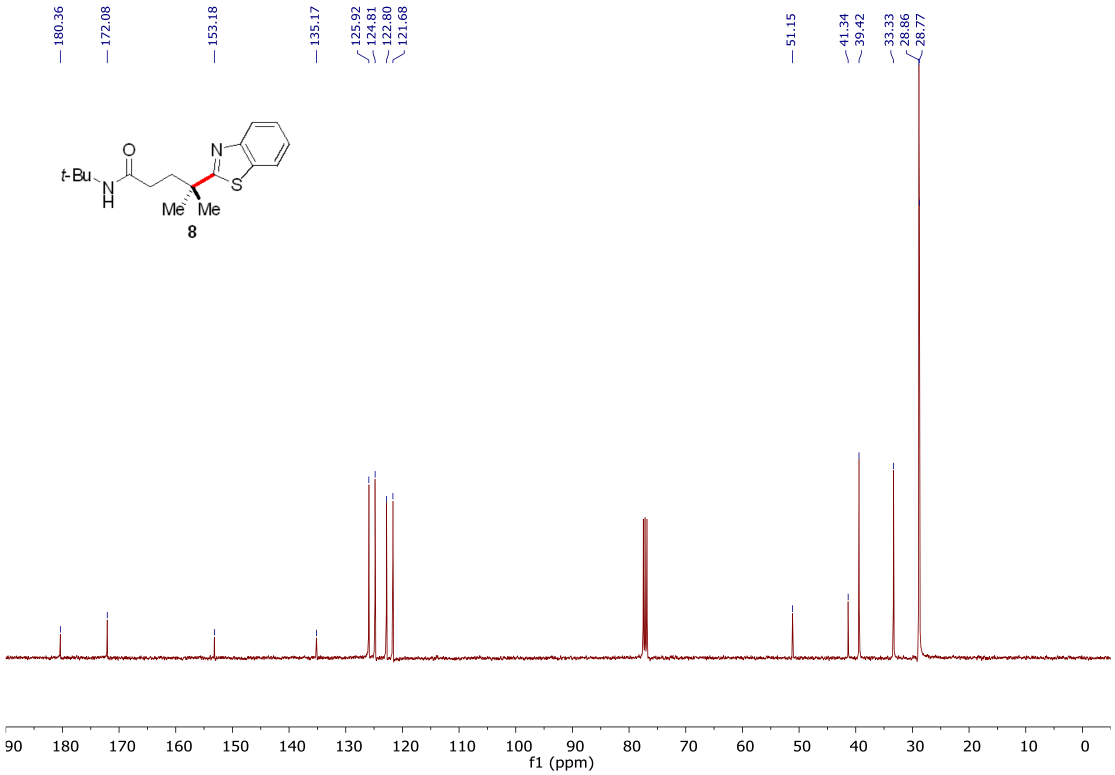


**Supplementary Figure 71.** $^{13}C$ NMR spectra for **8**

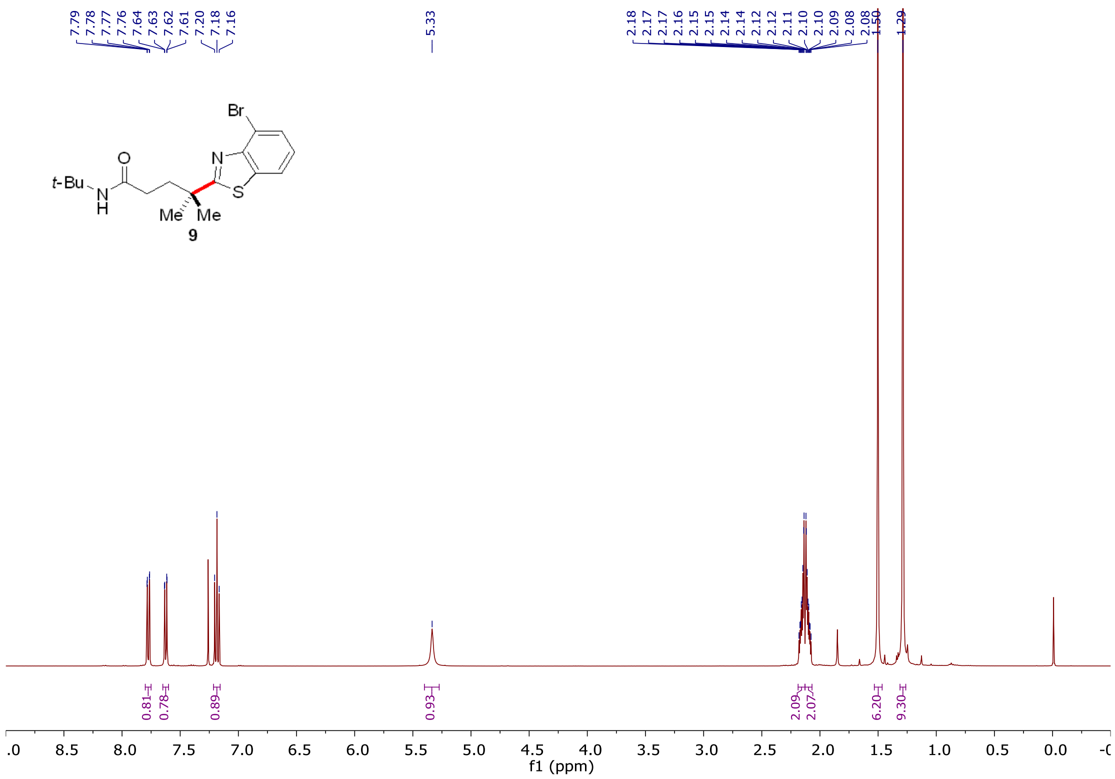

**Supplementary Figure 72.** $^{1}H$ NMR spectra for **9**

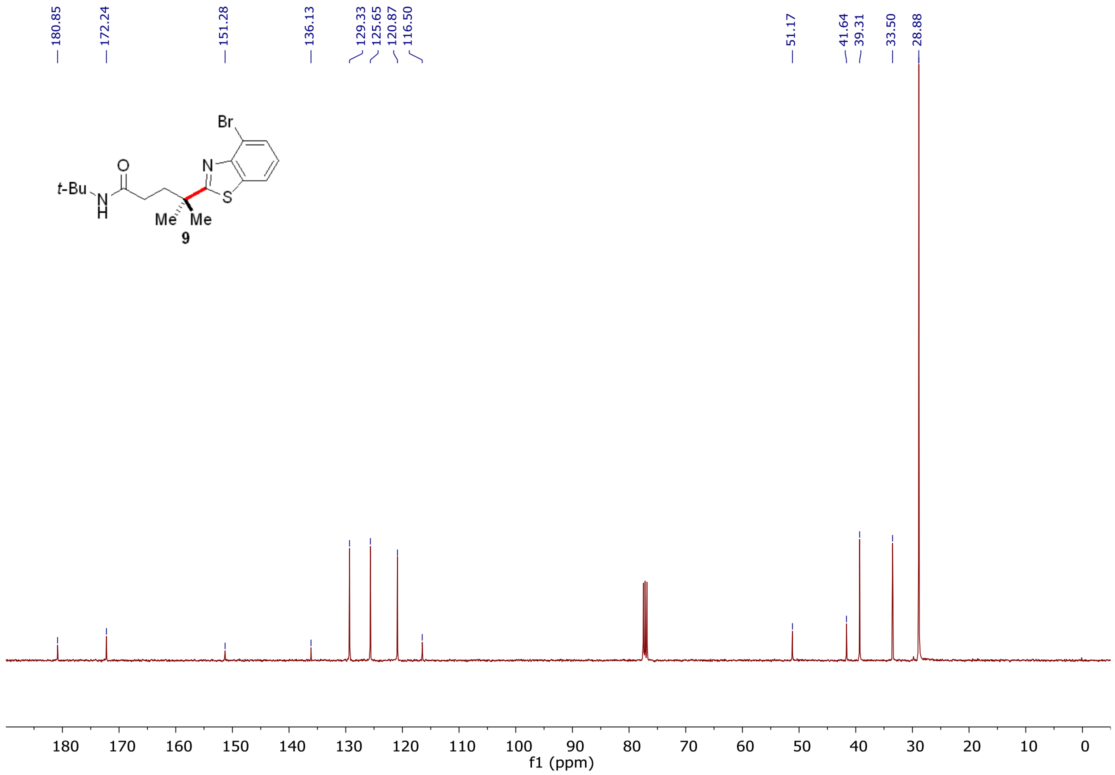

**Supplementary Figure 73.** $^{13}C$ NMR spectra for **9**

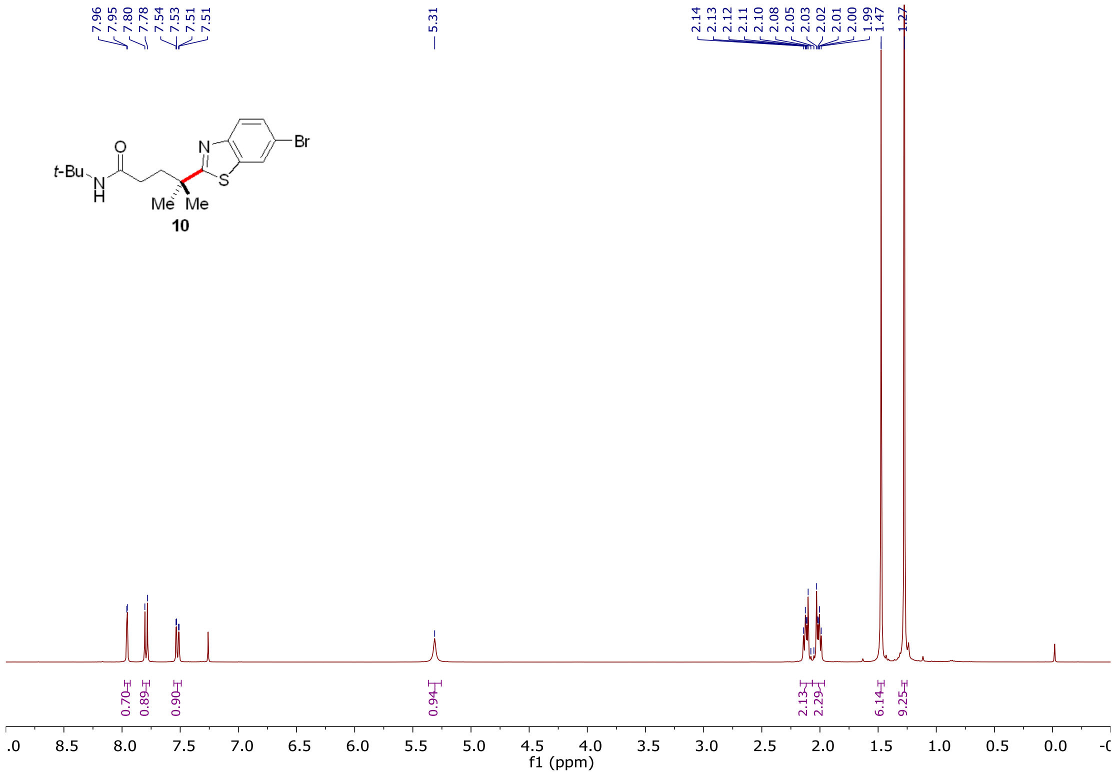


**Supplementary Figure 74.** $^{1}$H NMR spectra for **10**

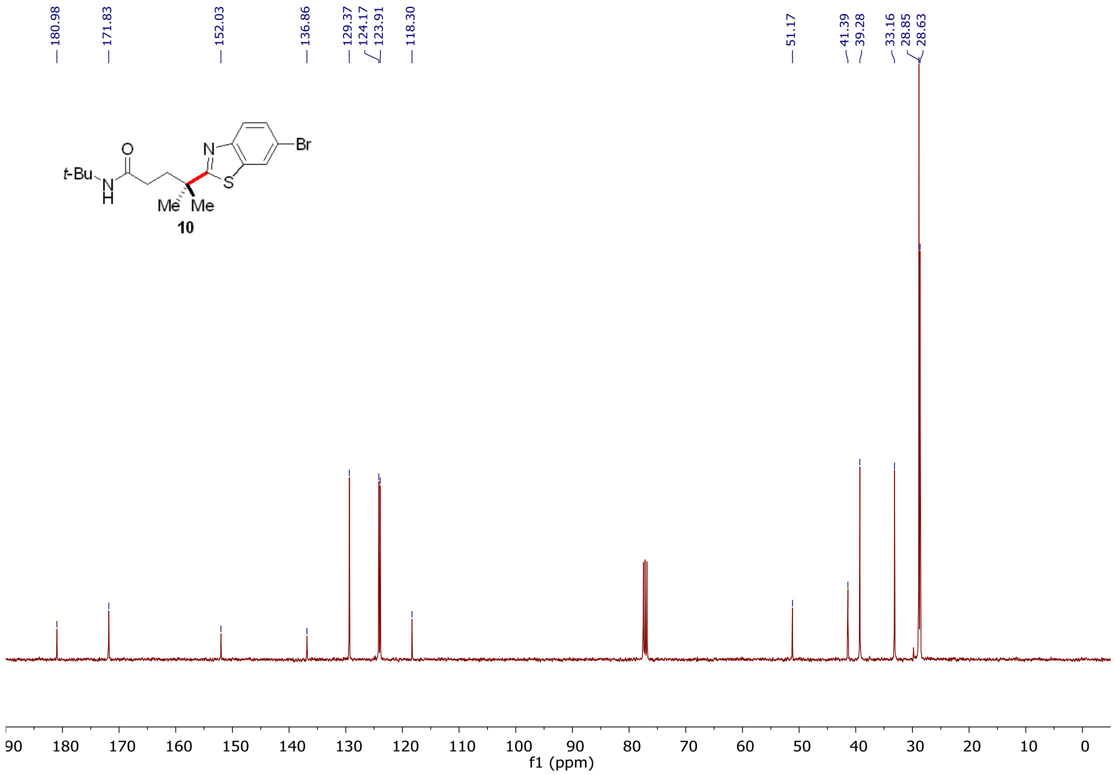


**Supplementary Figure 75.** $^{13}$C NMR spectra for **10**

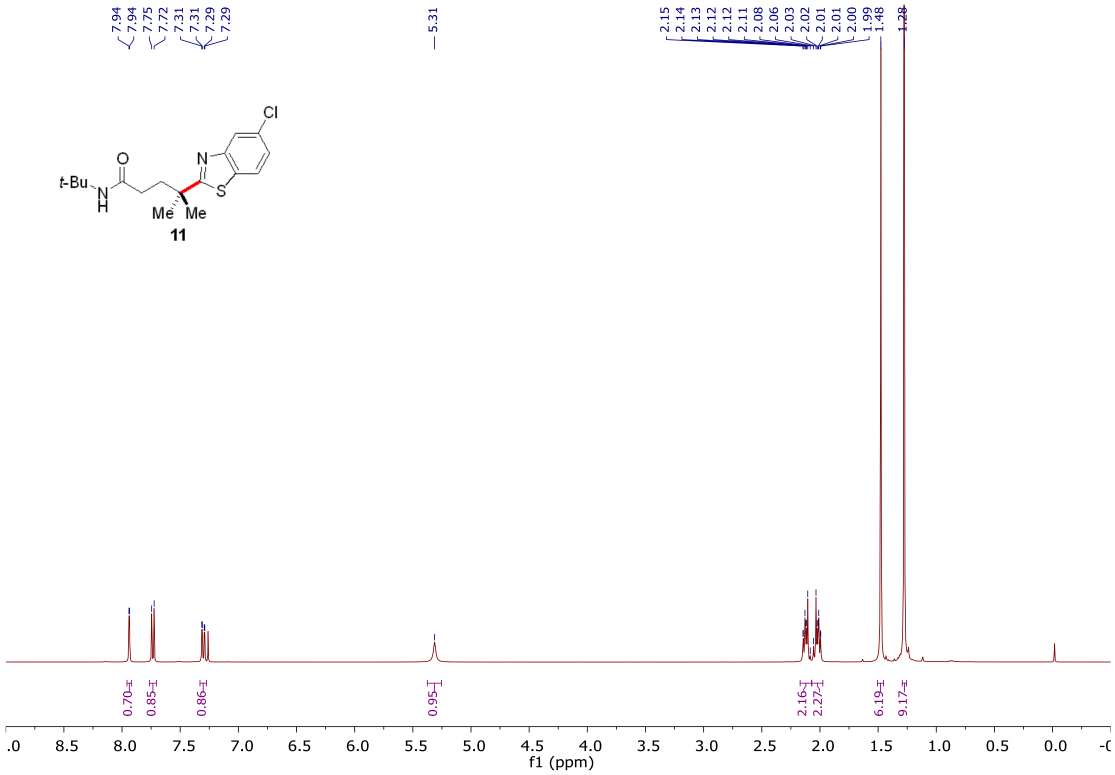


**Supplementary Figure 76.** $^{1}$H NMR spectra for **11**

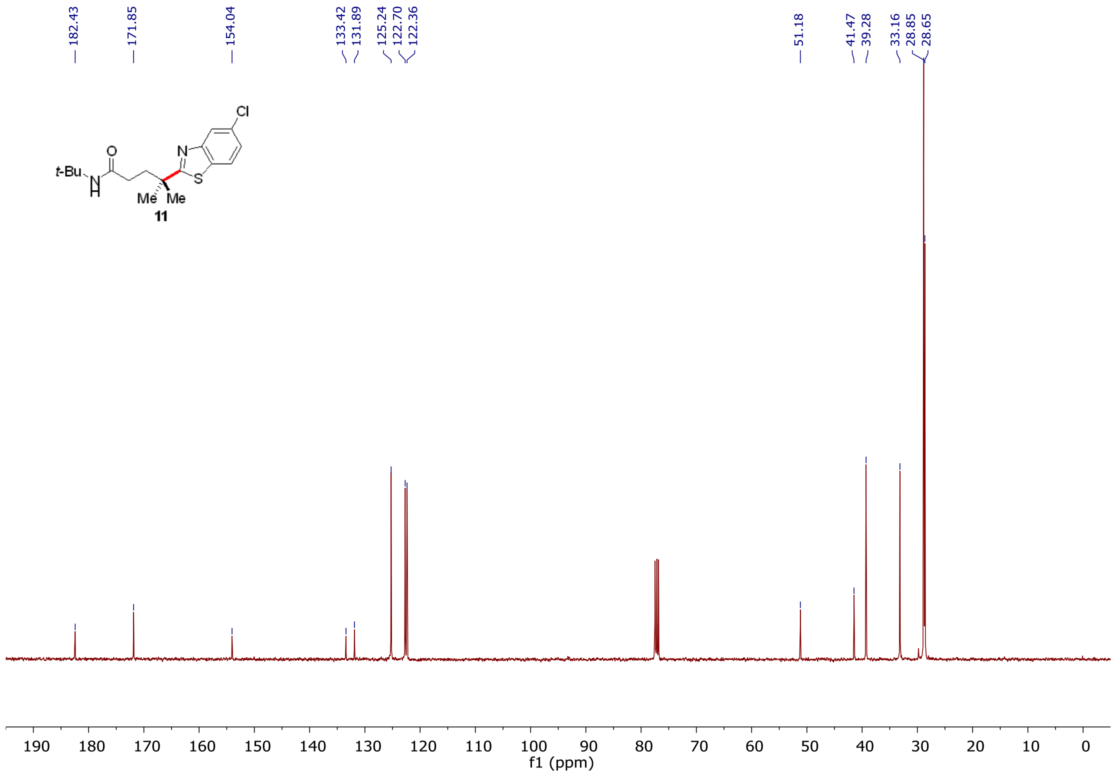


**Supplementary Figure 77.** $^{13}$C NMR spectra for **11**

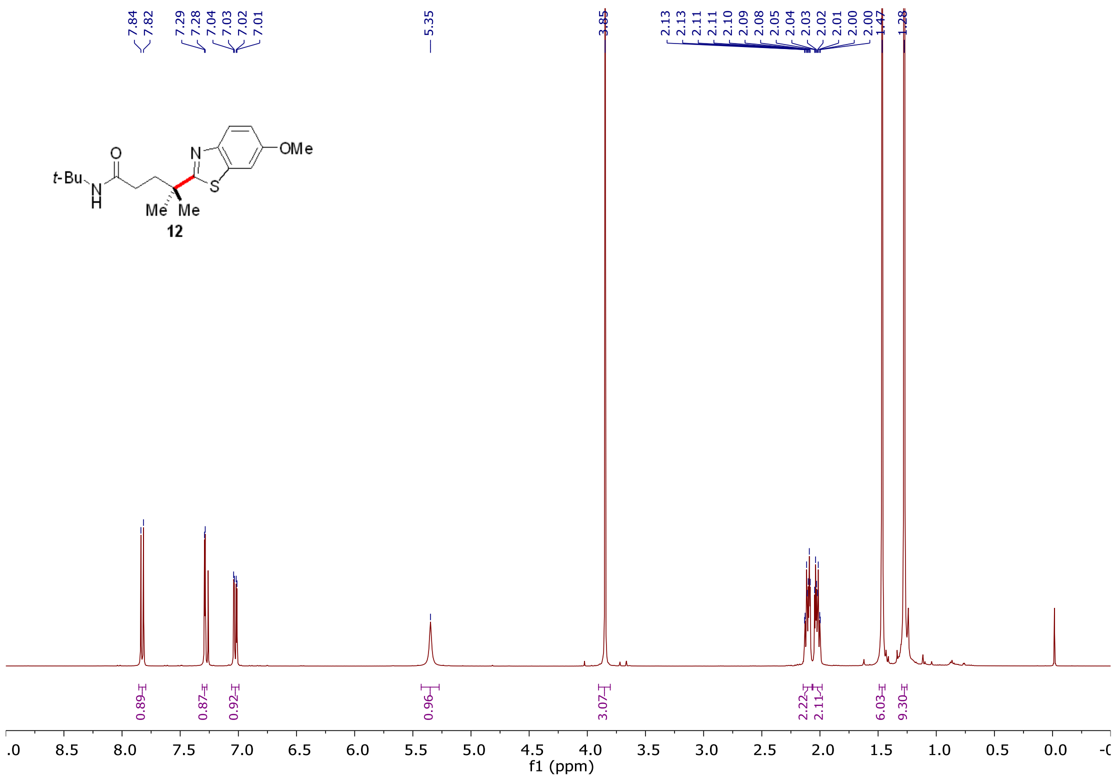


**Supplementary Figure 78.** $^{1}$H NMR spectra for **12**

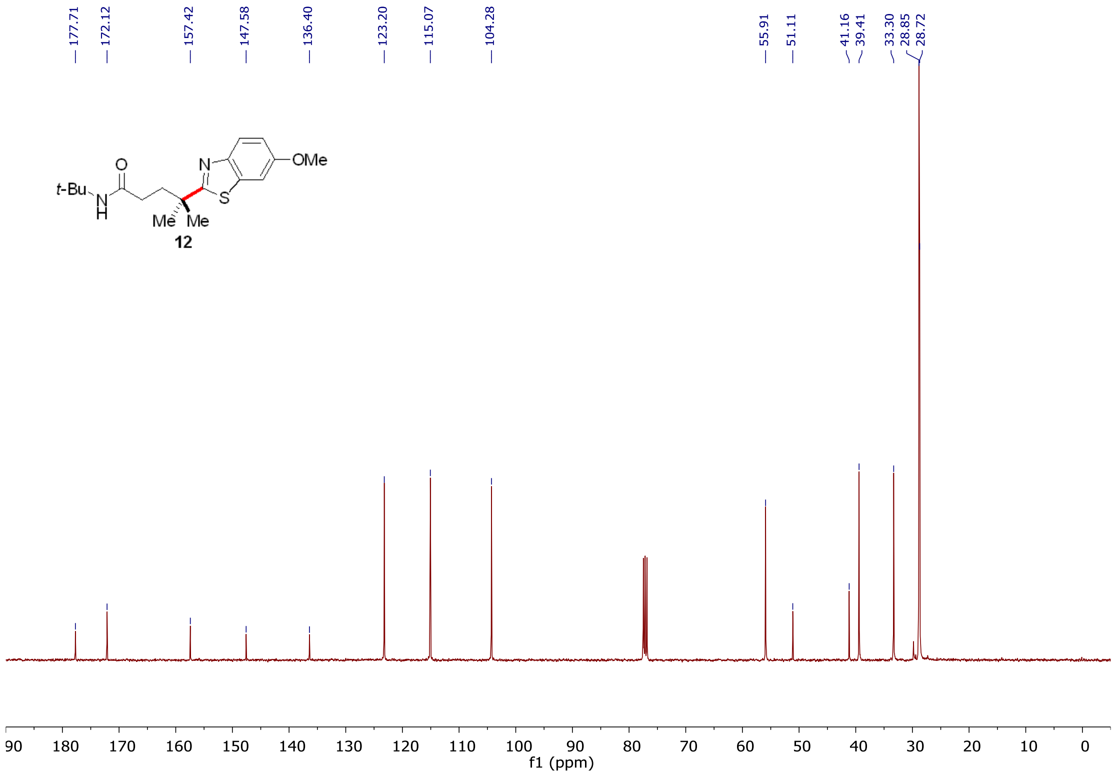


**Supplementary Figure 79.** $^{13}$C NMR spectra for **12**

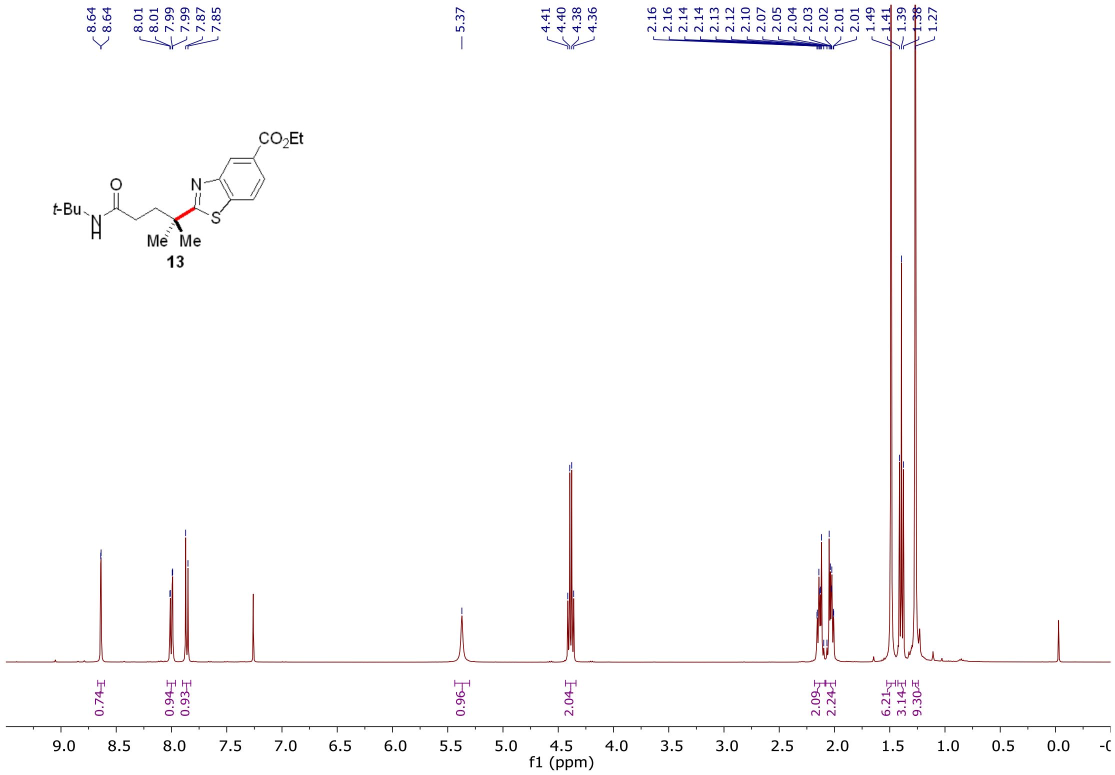


**Supplementary Figure 80.** $^{1}$H NMR spectra for **13**

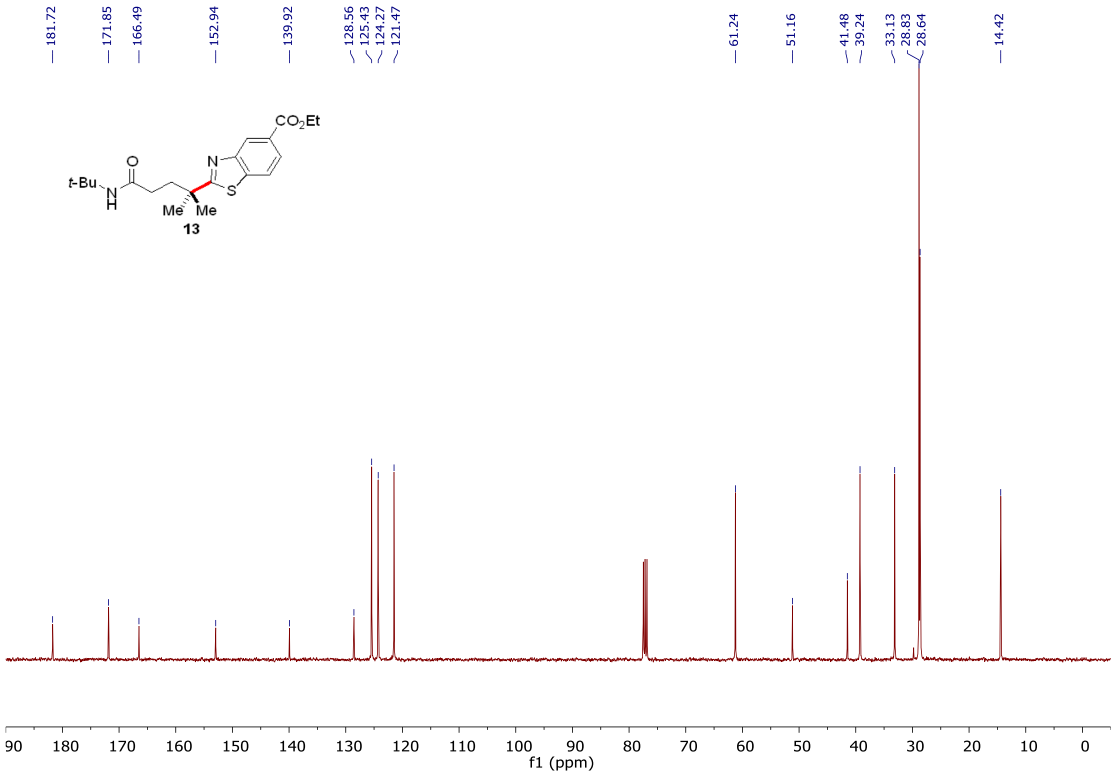


**Supplementary Figure 81.** $^{13}$C NMR spectra for **13**

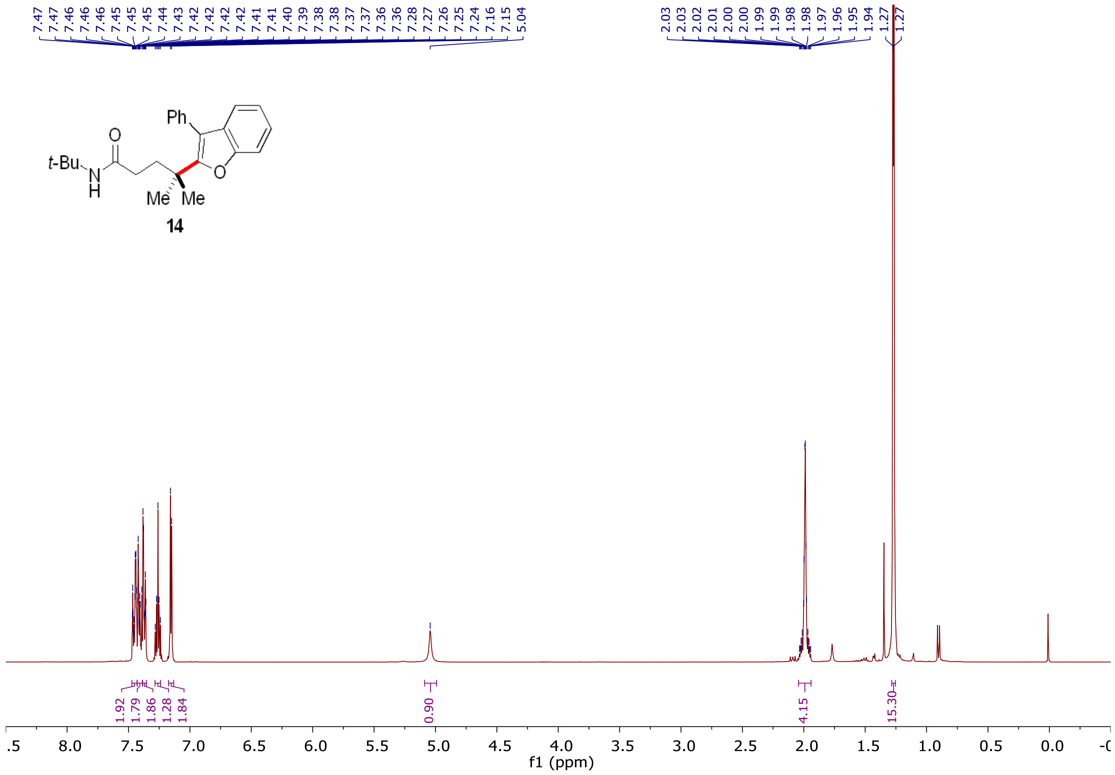

**Supplementary Figure 82.** $^{1}$H NMR spectra for **14**

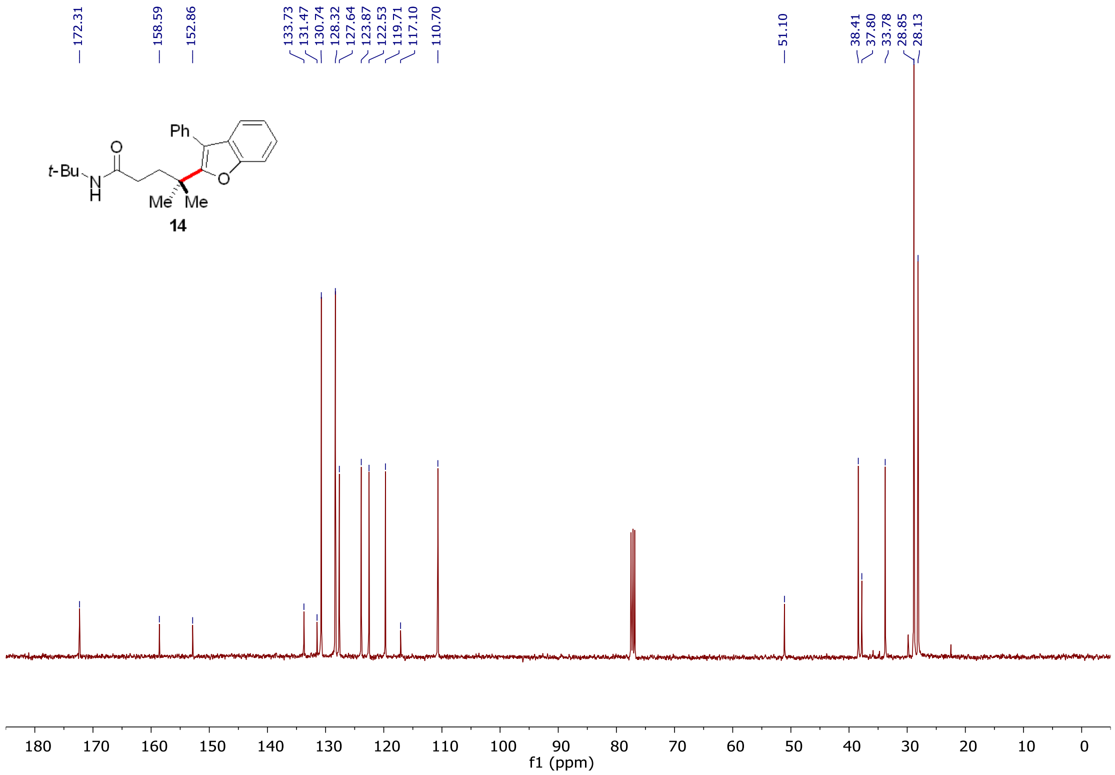

**Supplementary Figure 83.** $^{13}$C NMR spectra for **14**

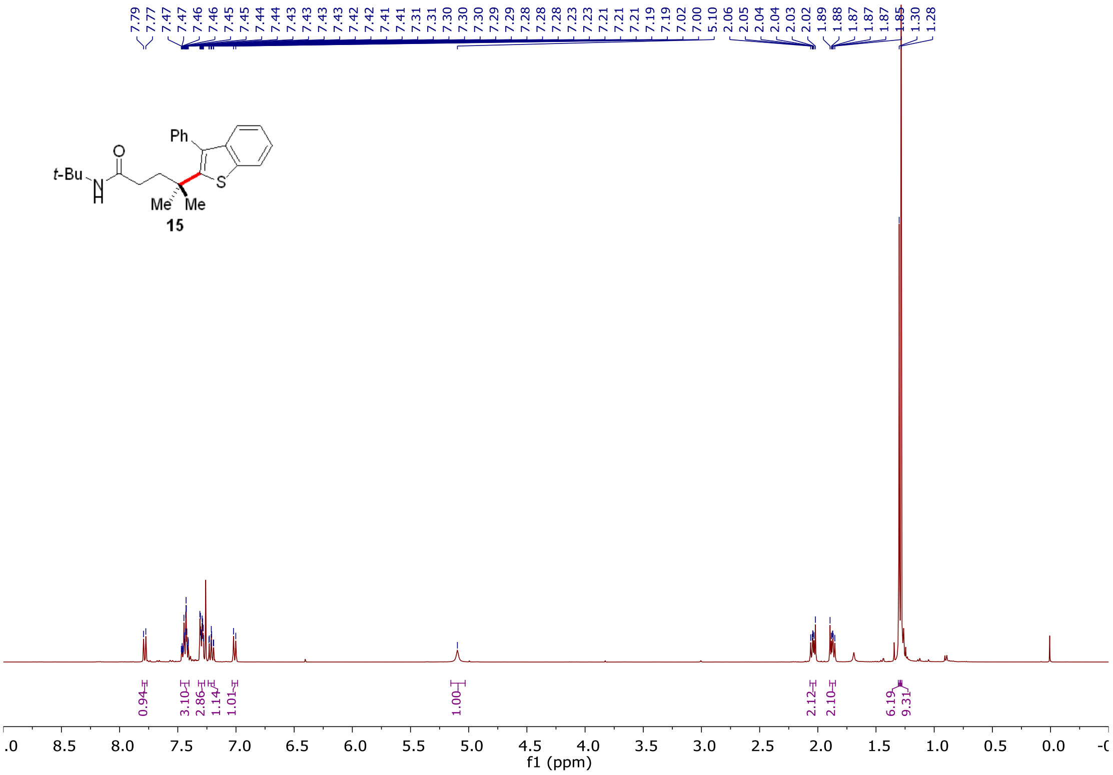

**Supplementary Figure 84.** $^{1}$H NMR spectra for **15**

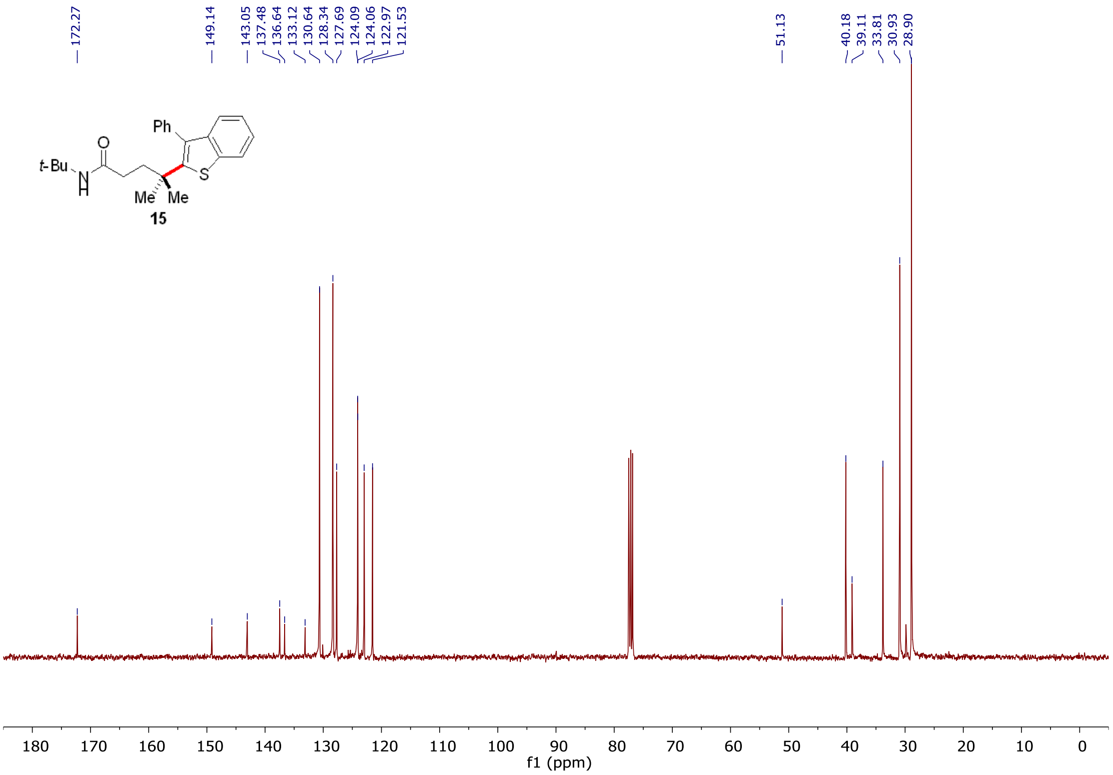

**Supplementary Figure 85.** $^{13}$C NMR spectra for **15**

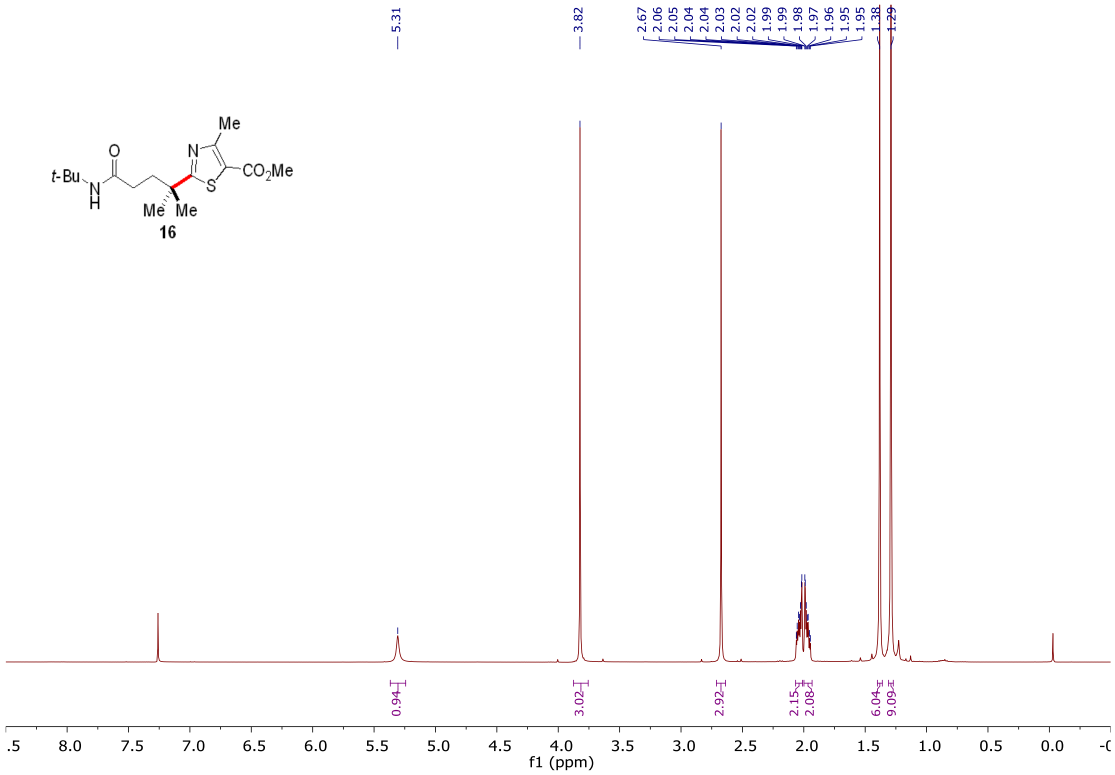


**Supplementary Figure 86.** $^1$H NMR spectra for **16**

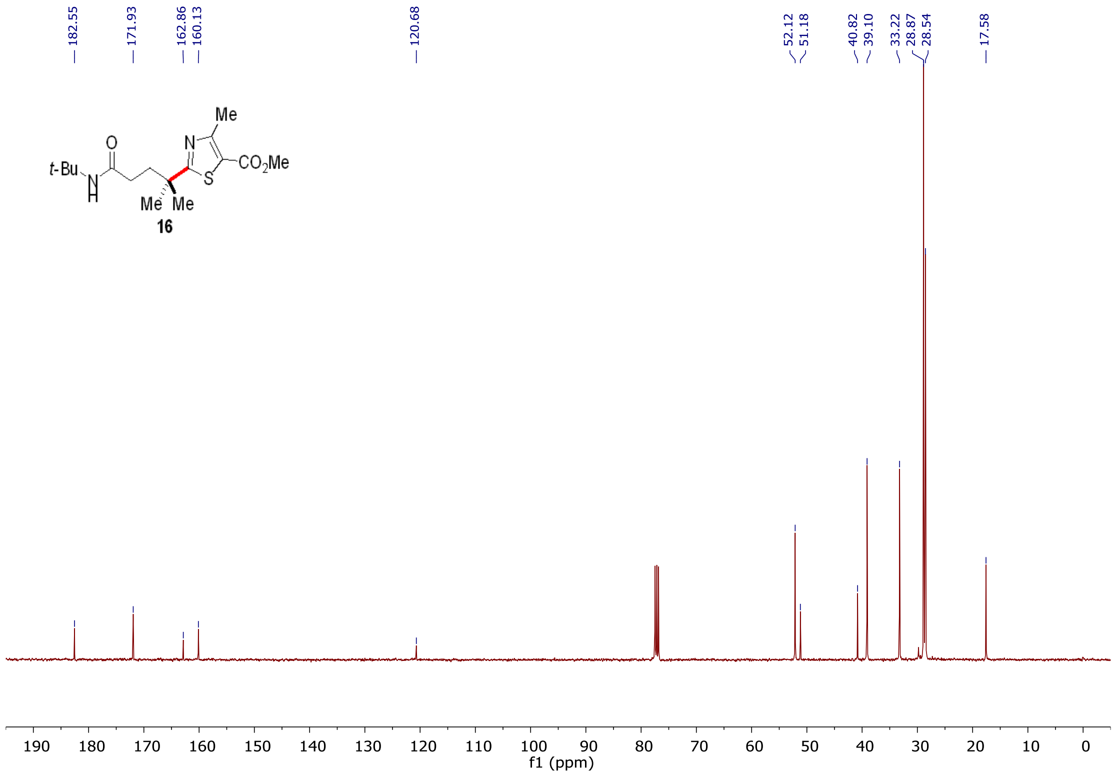


**Supplementary Figure 87.** $^{13}$C NMR spectra for **16**

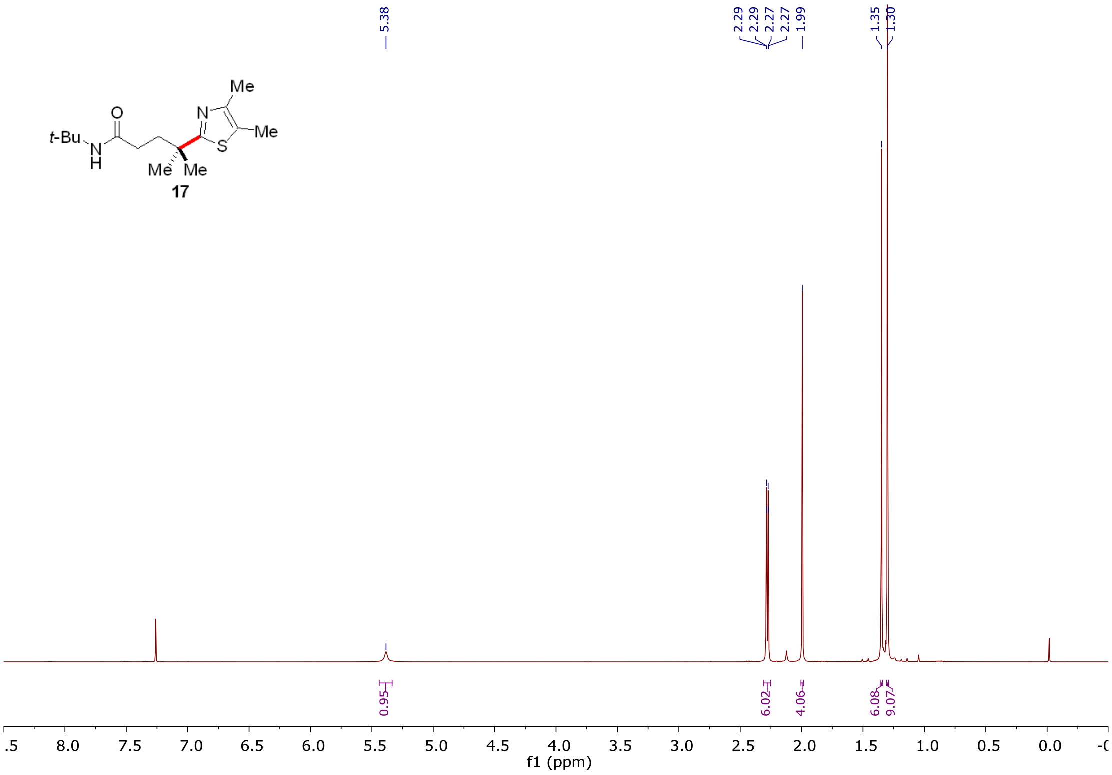

**Supplementary Figure 88.** $^{1}$H NMR spectra for **17**

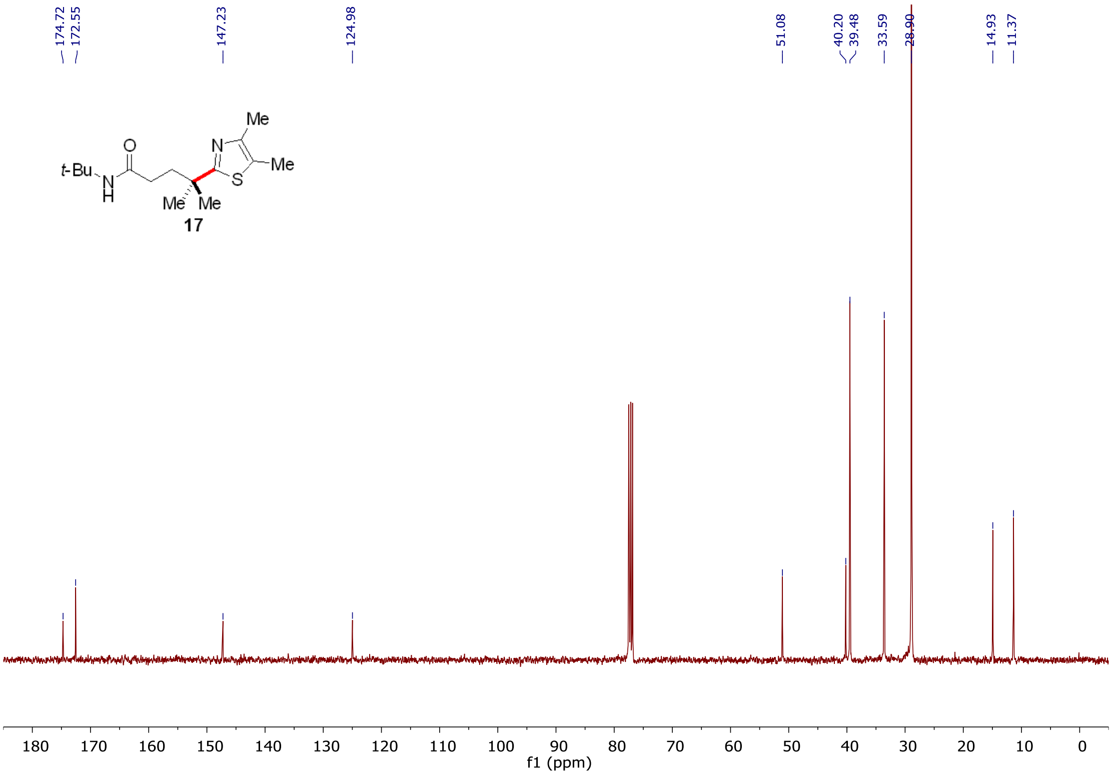

**Supplementary Figure 89.** $^{13}$C NMR spectra for **17**

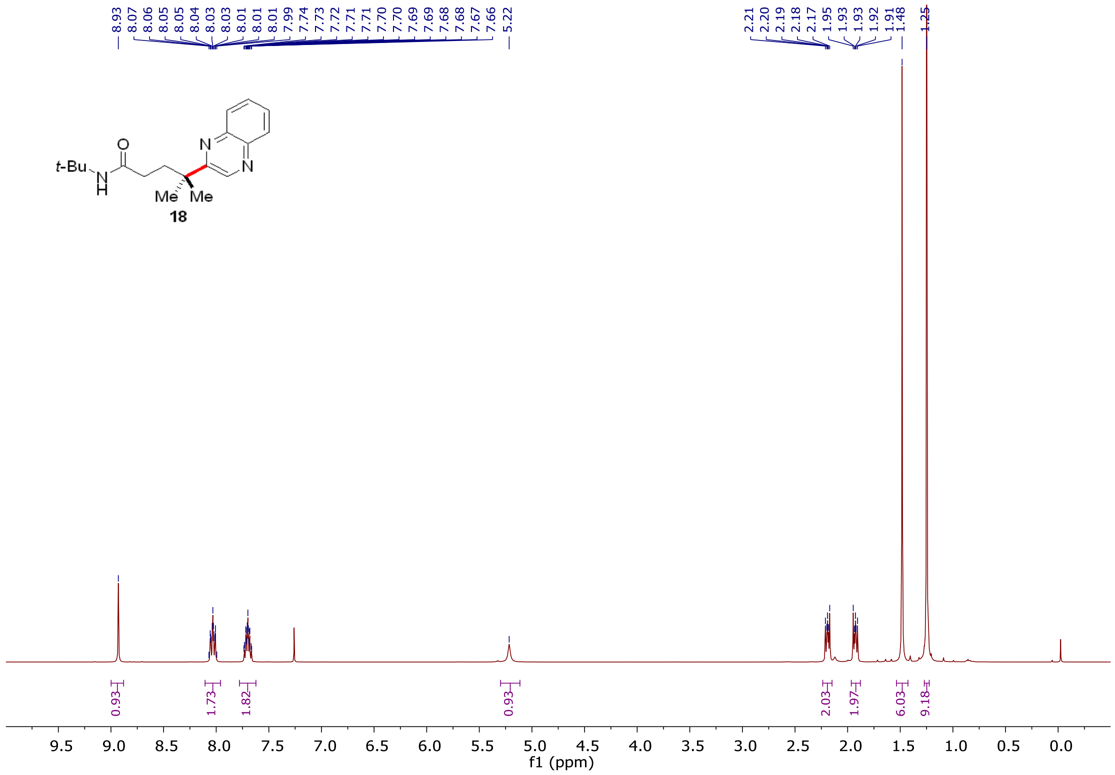


**Supplementary Figure 90.** $^{1}$H NMR spectra for **18**

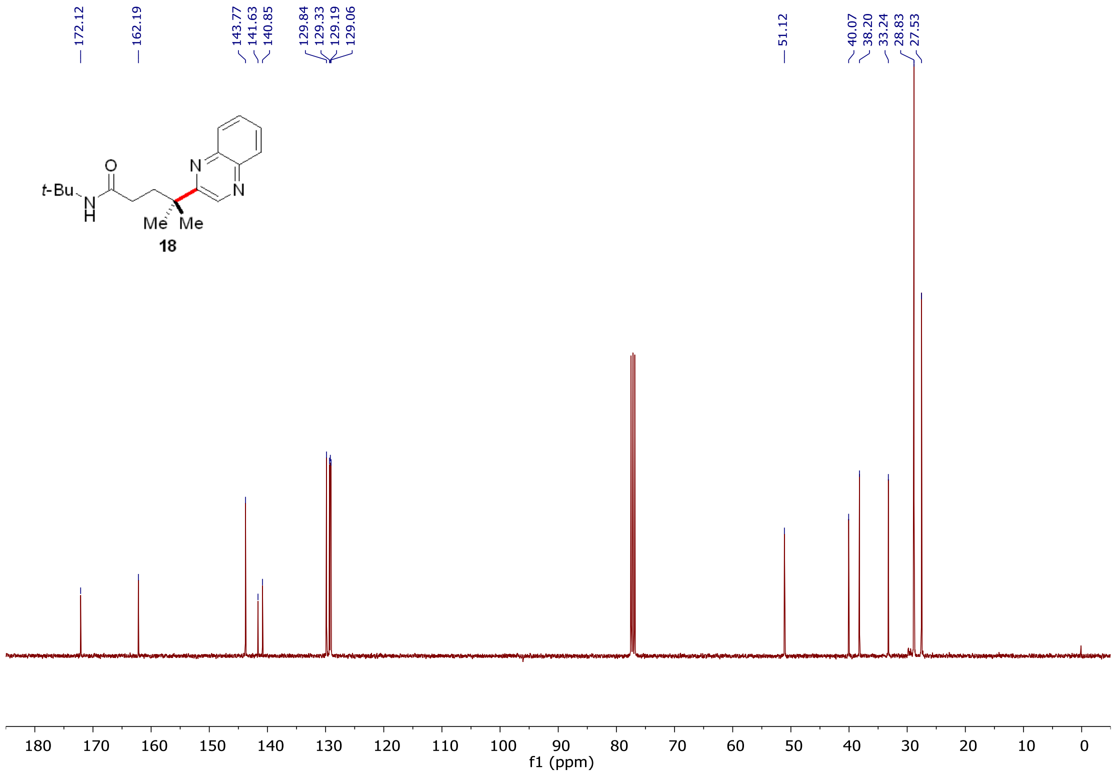


**Supplementary Figure 91.** $^{13}$C NMR spectra for **18**

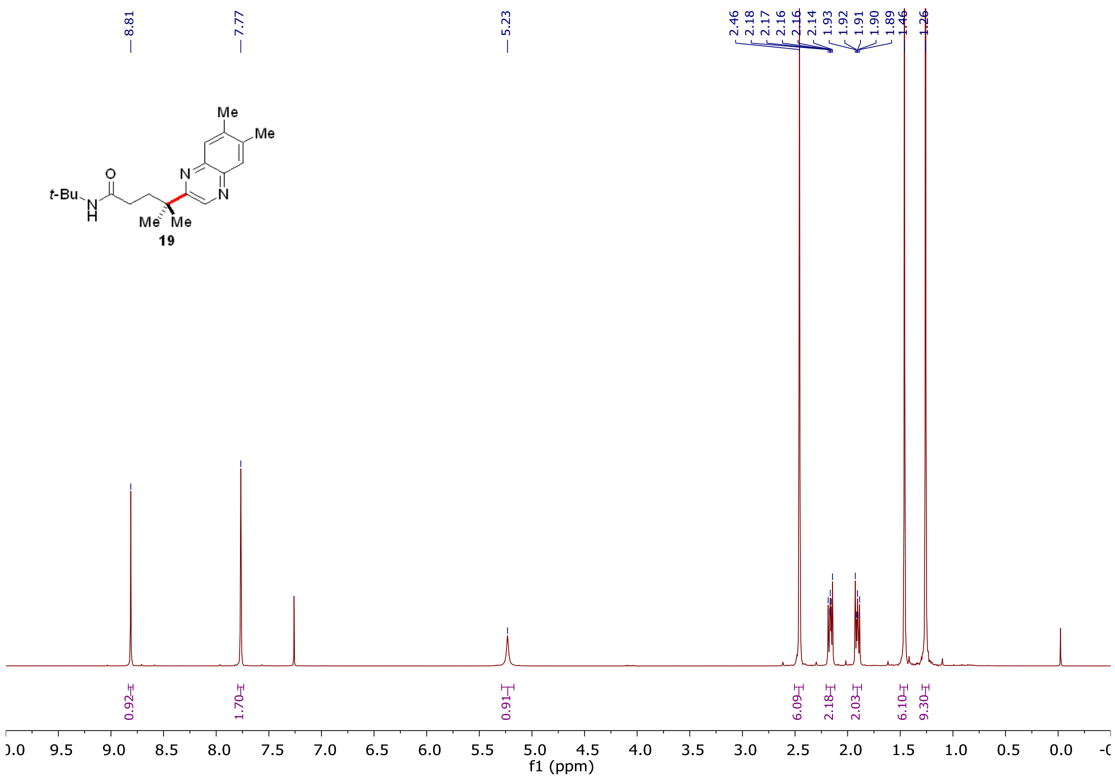


**Supplementary Figure 92.** $^{1}$H NMR spectra for **19**

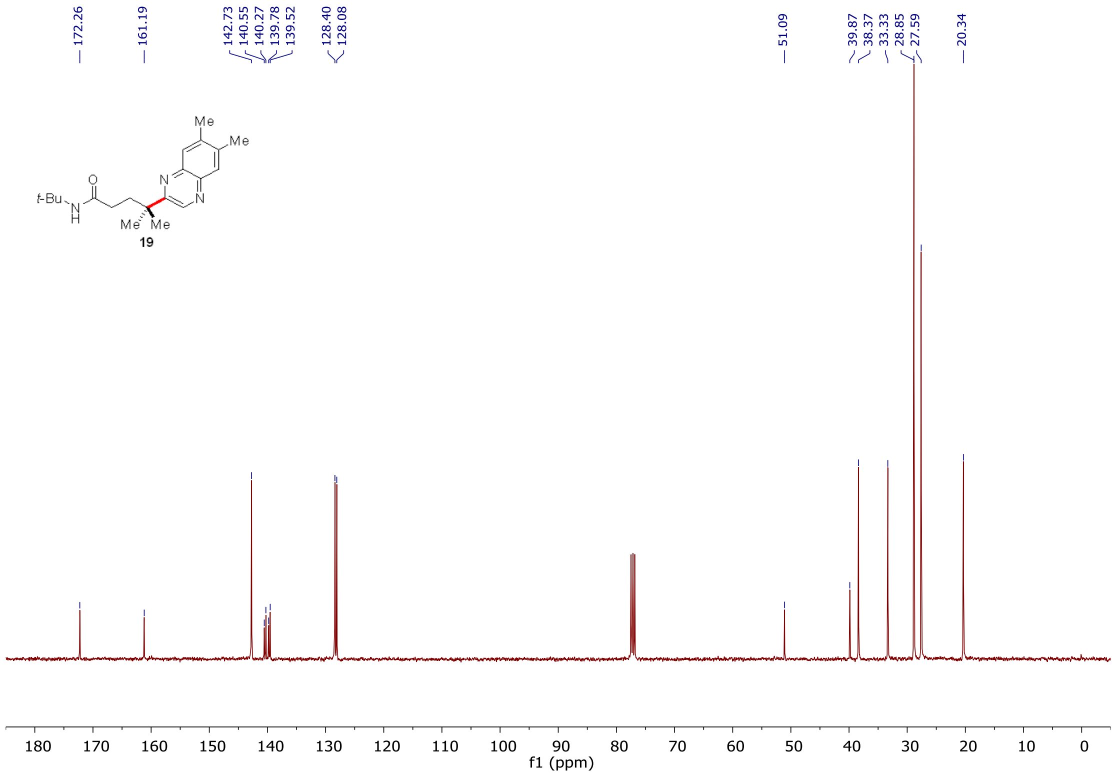


**Supplementary Figure 93.** $^{13}$C NMR spectra for **19**

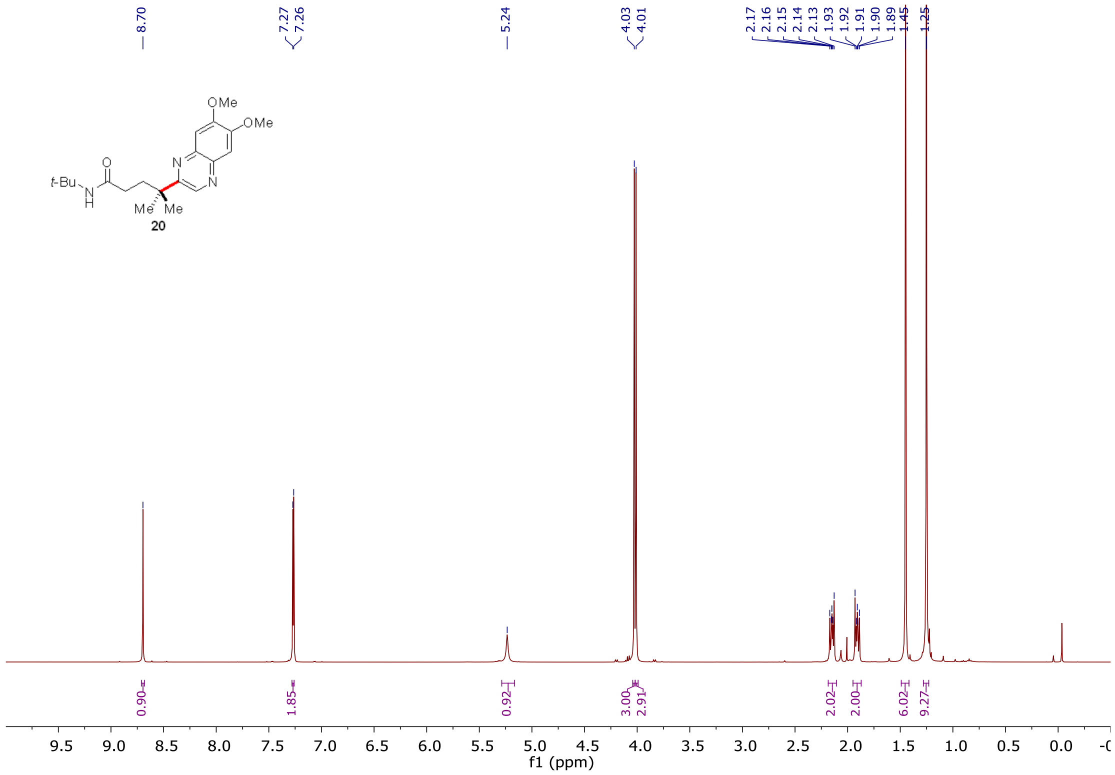


**Supplementary Figure 94.** $^{1}$H NMR spectra for **20**

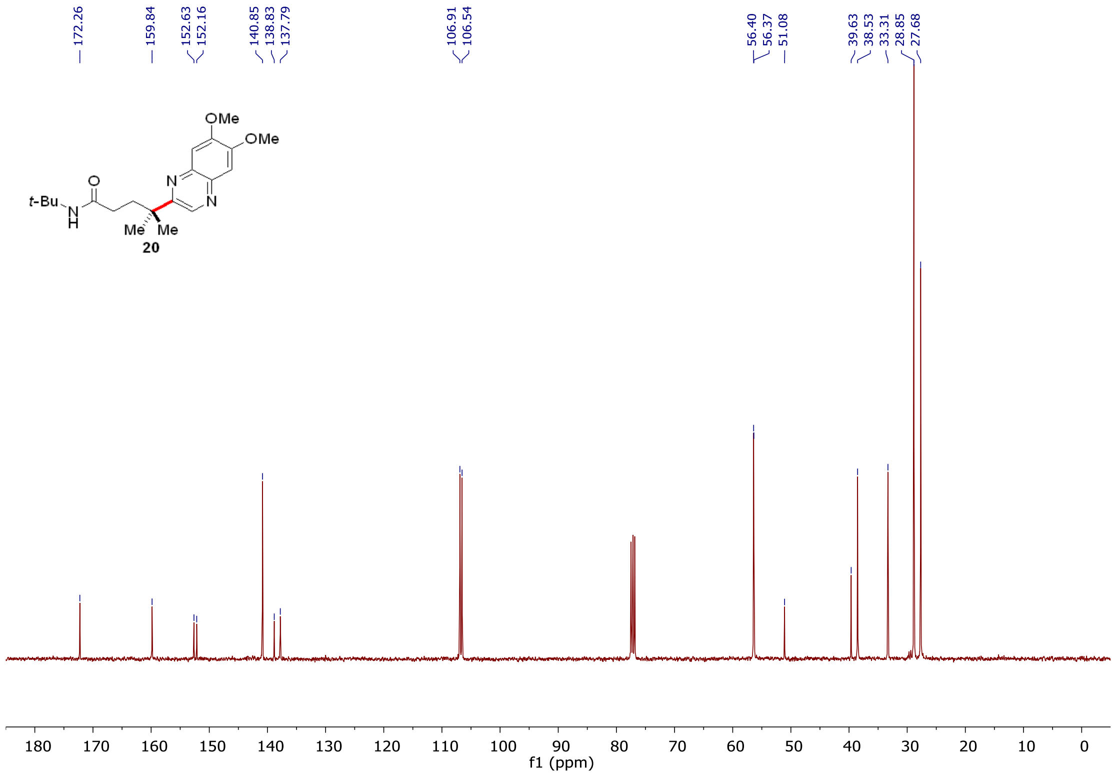


**Supplementary Figure 95.** $^{13}$C NMR spectra for **20**

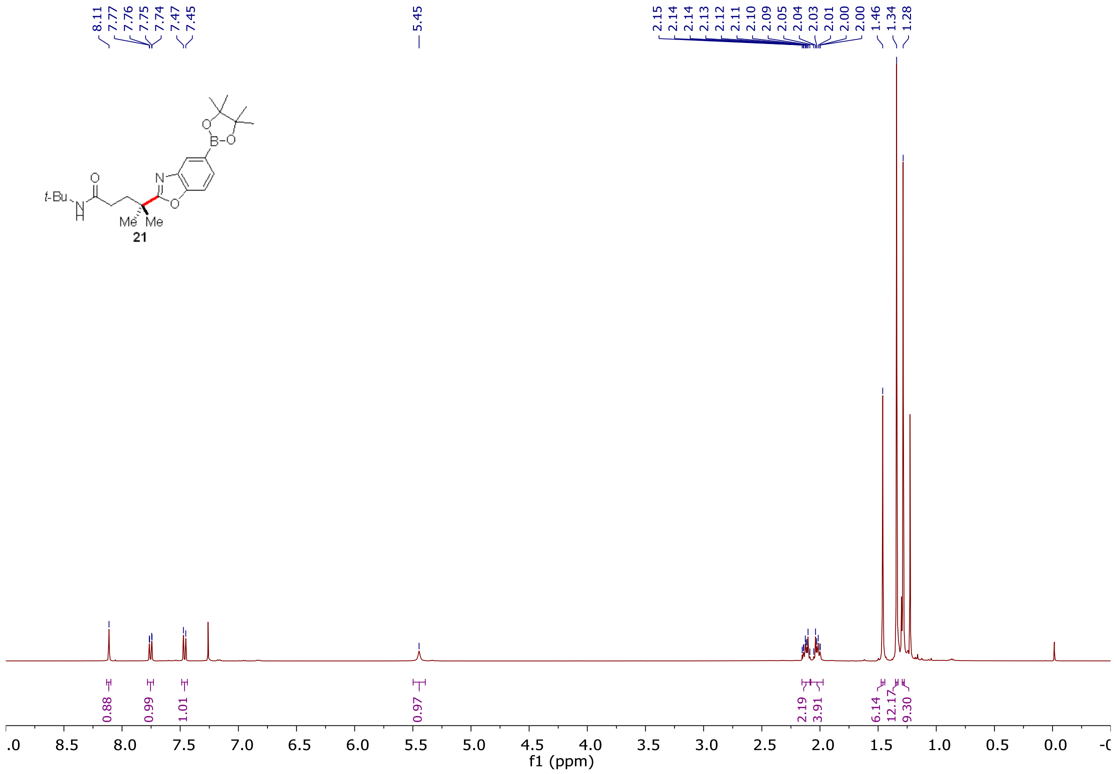


**Supplementary Figure 96.** $^{1}H$ NMR spectra for **21**

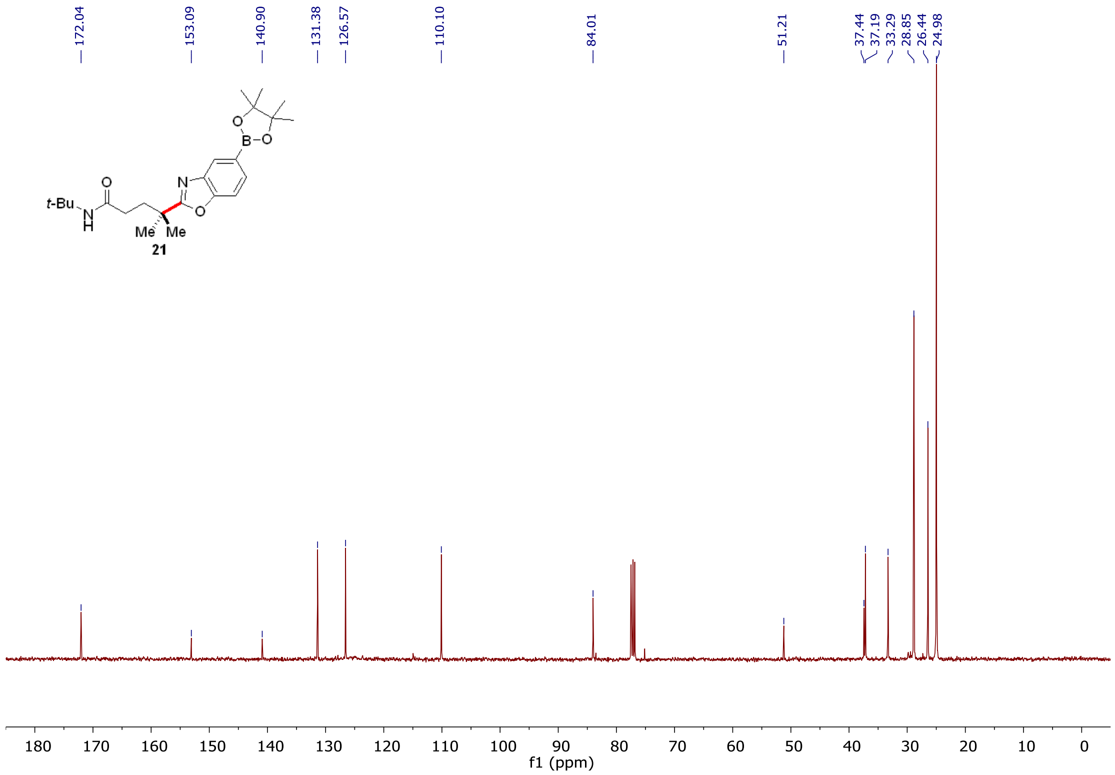


**Supplementary Figure 97.** $^{13}C$ NMR spectra for **21**

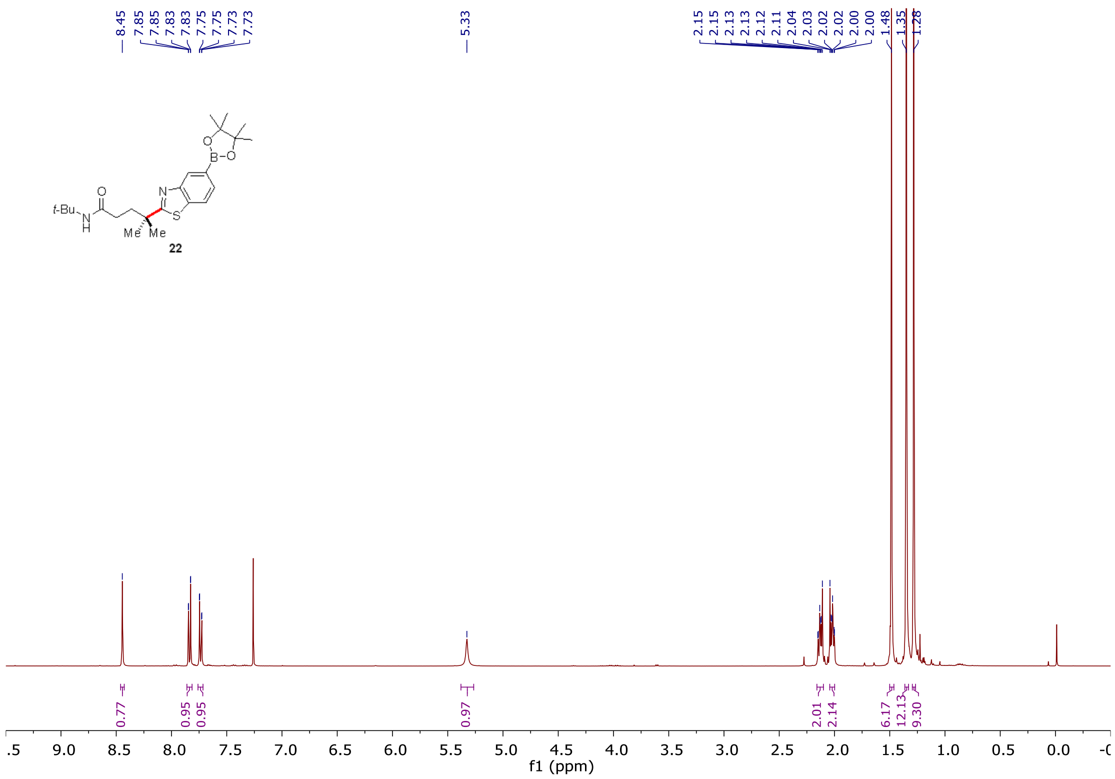


**Supplementary Figure 98.** $^{1}$H NMR spectra for **22**

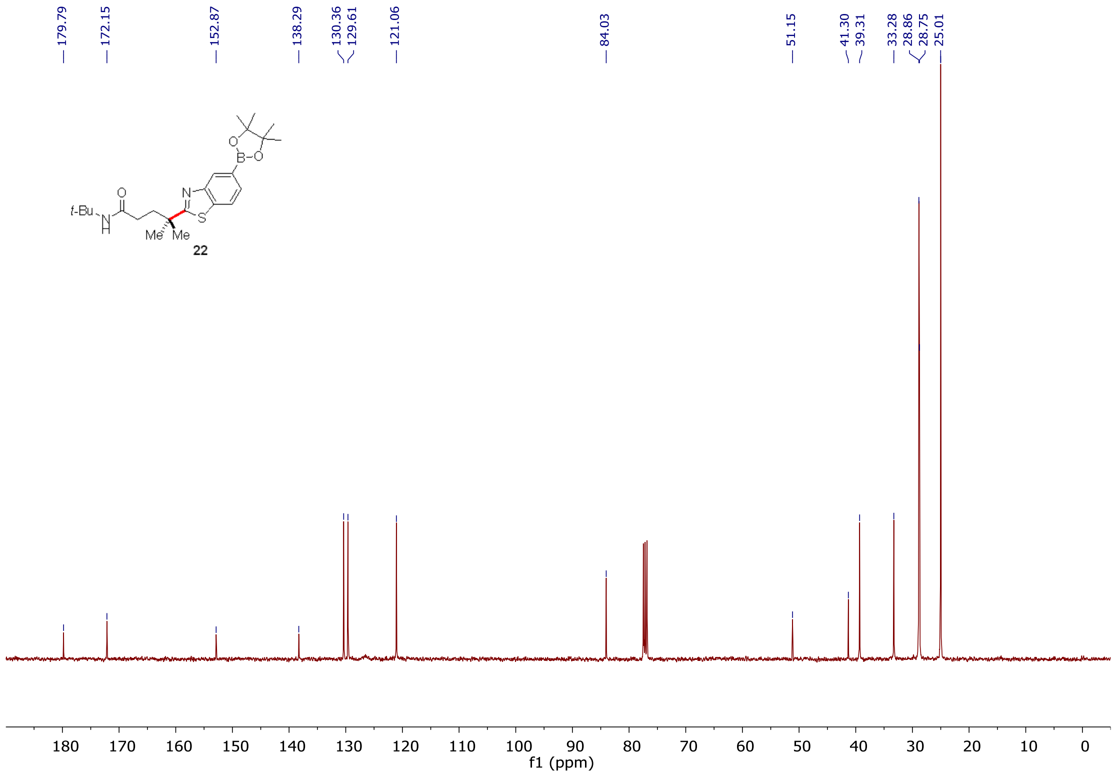


**Supplementary Figure 99.** $^{13}$C NMR spectra for **22**

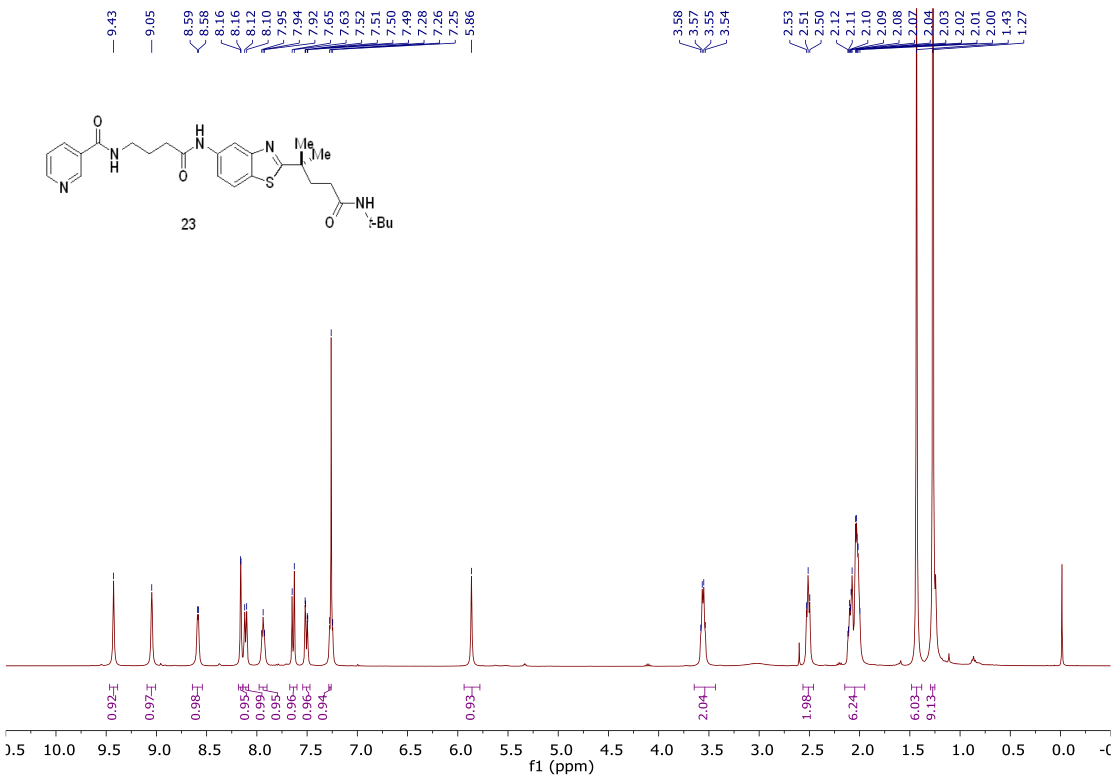

**Supplementary Figure 100.** $^{1}$H NMR spectra for **23**

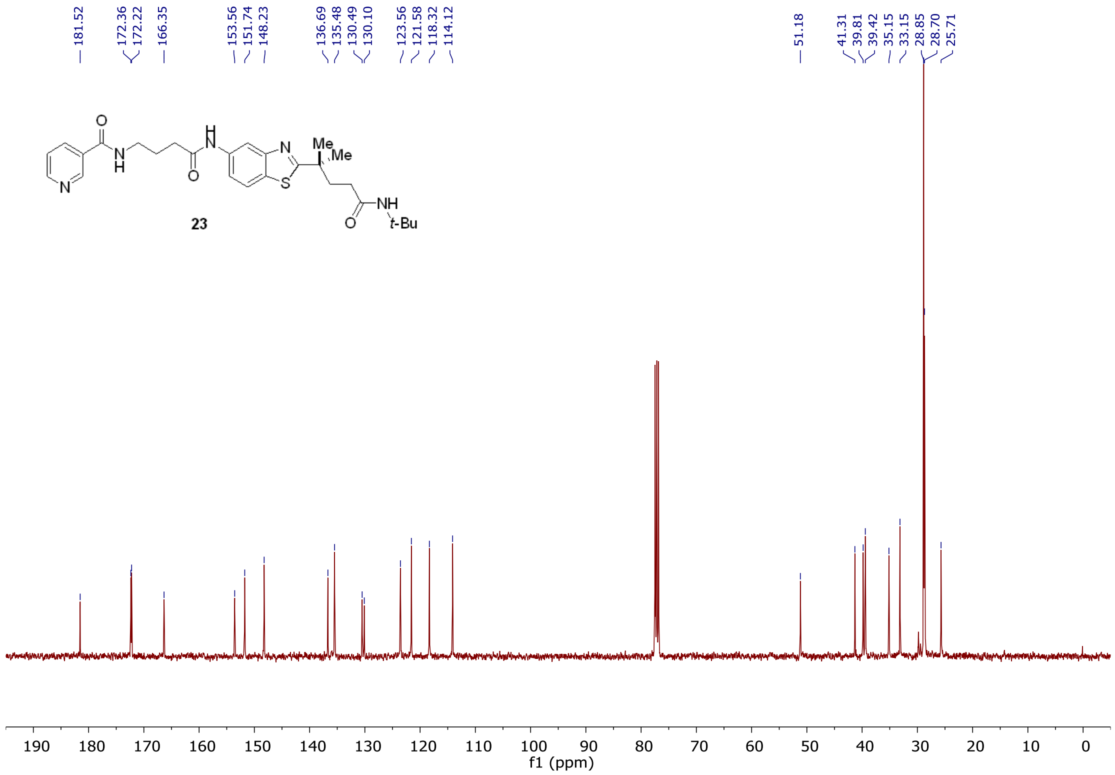

**Supplementary Figure 101.** $^{13}$C NMR spectra for **23**

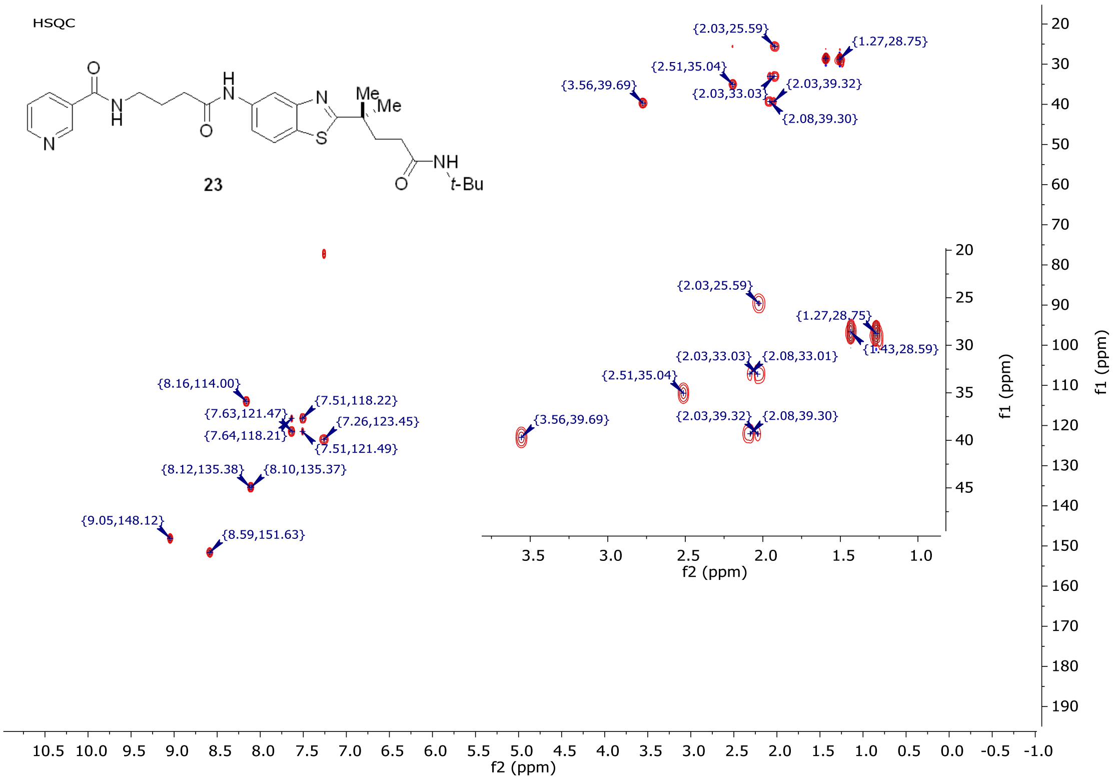


**Supplementary Figure 102.** HSQC NMR spectra for **23**

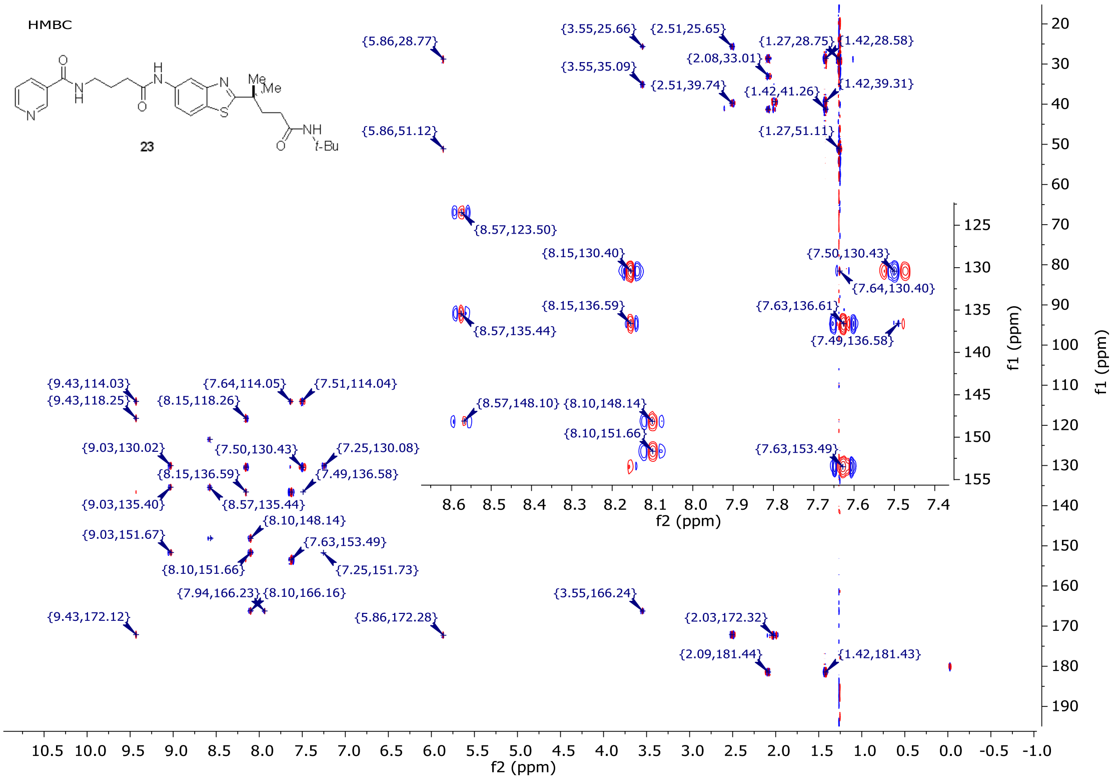


**Supplementary Figure 103.** HMBC NMR spectra for **23**

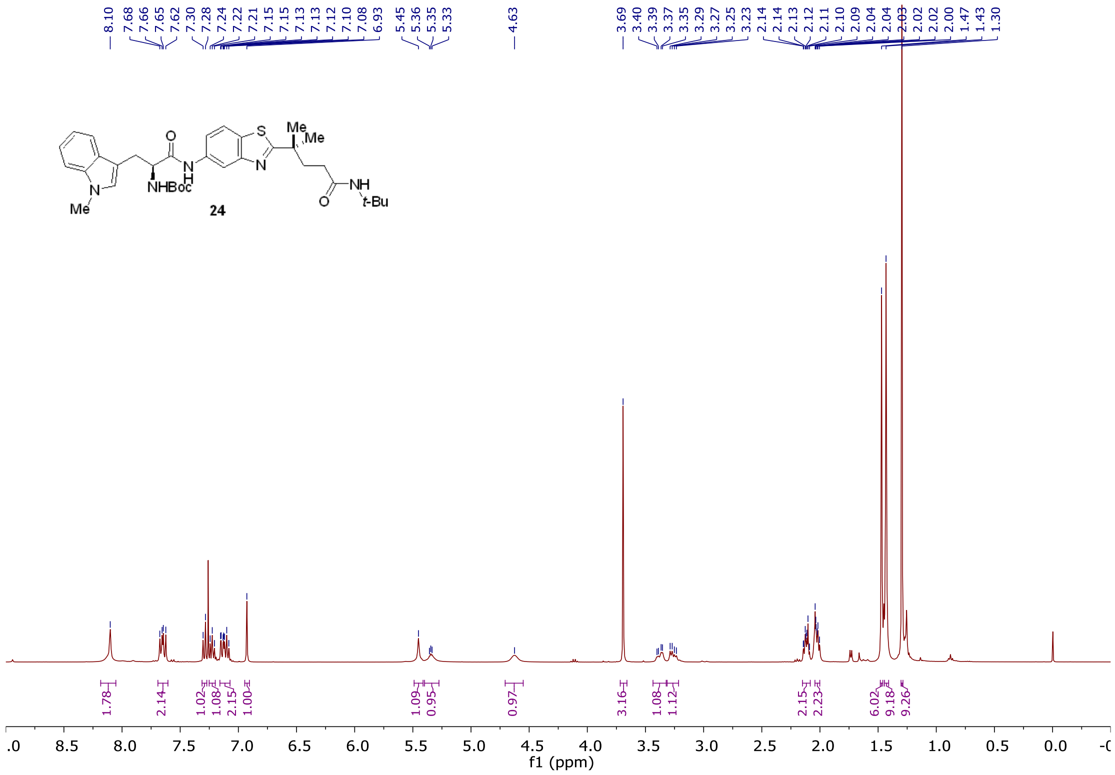


**Supplementary Figure 104.** $^{1}$H NMR spectra for **24**

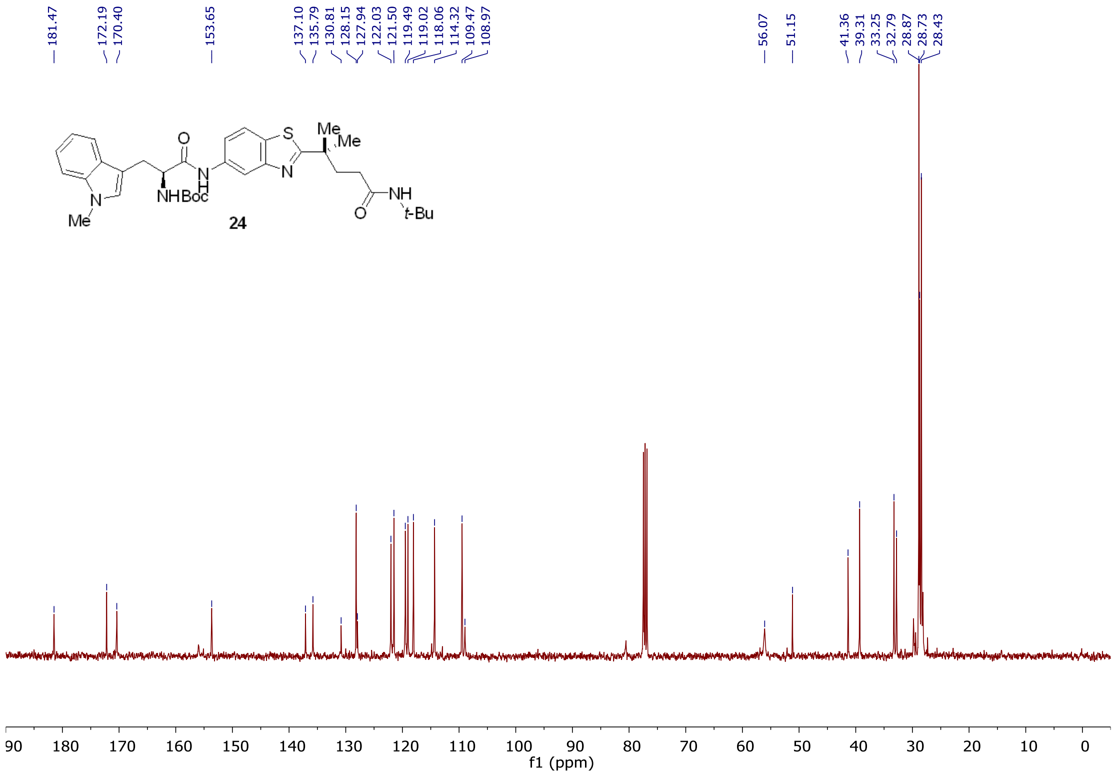


**Supplementary Figure 105.** $^{13}$C NMR spectra for **24**

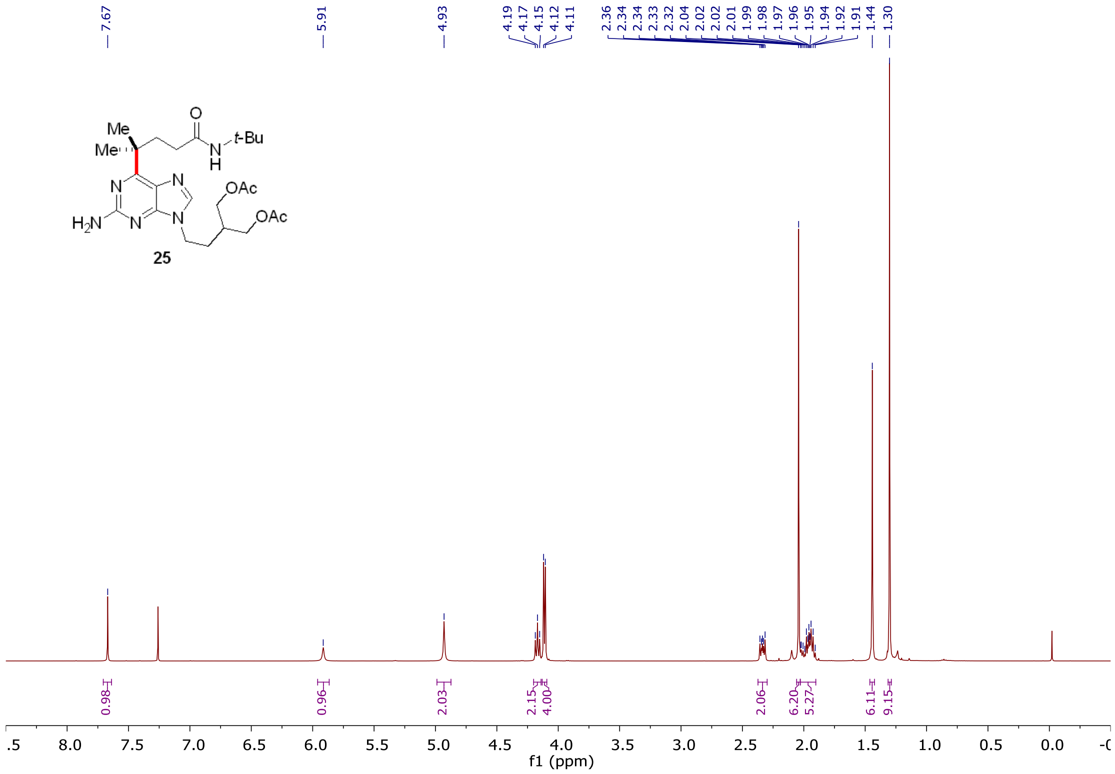


**Supplementary Figure 106.** $^{1}H$ NMR spectra for **25**

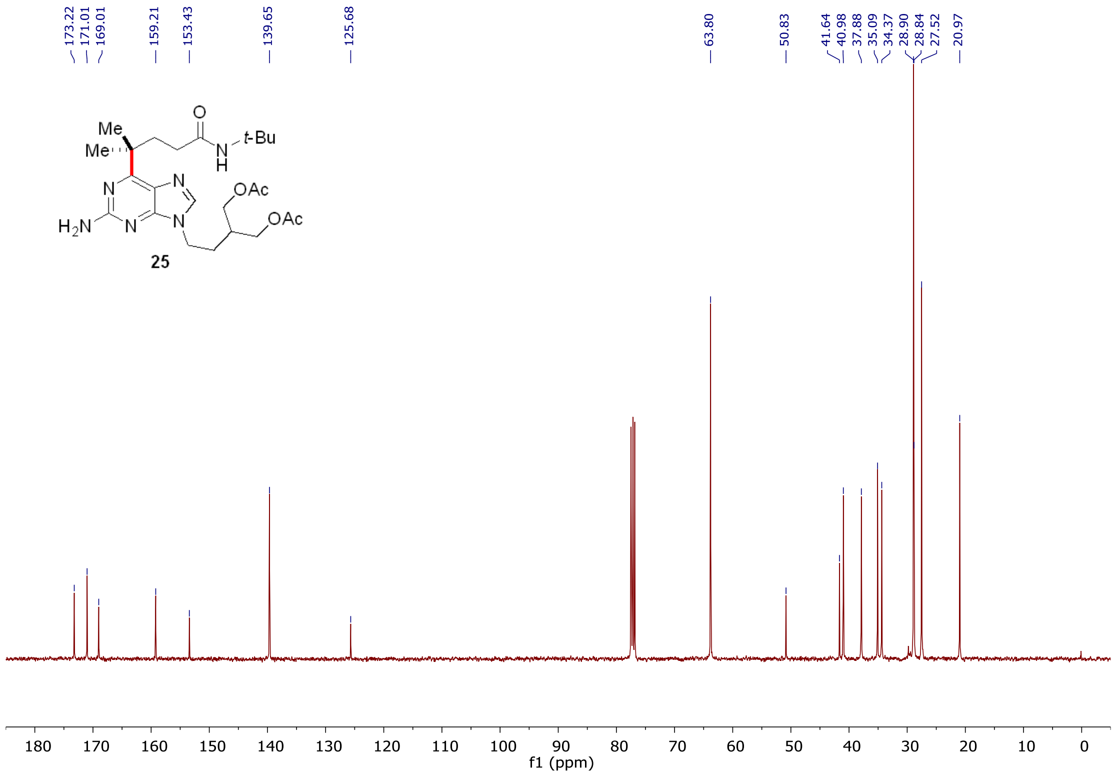


**Supplementary Figure 107.** $^{13}C$ NMR spectra for **25**

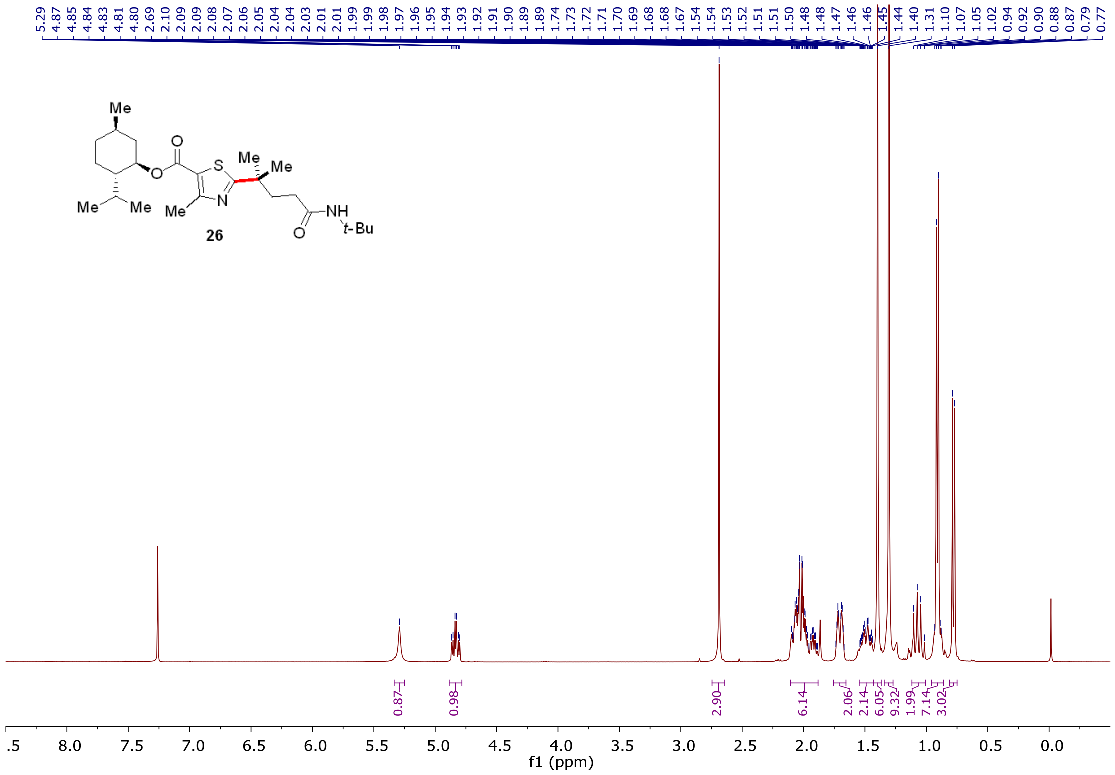


**Supplementary Figure 108.** $^{1}$H NMR spectra for **26**

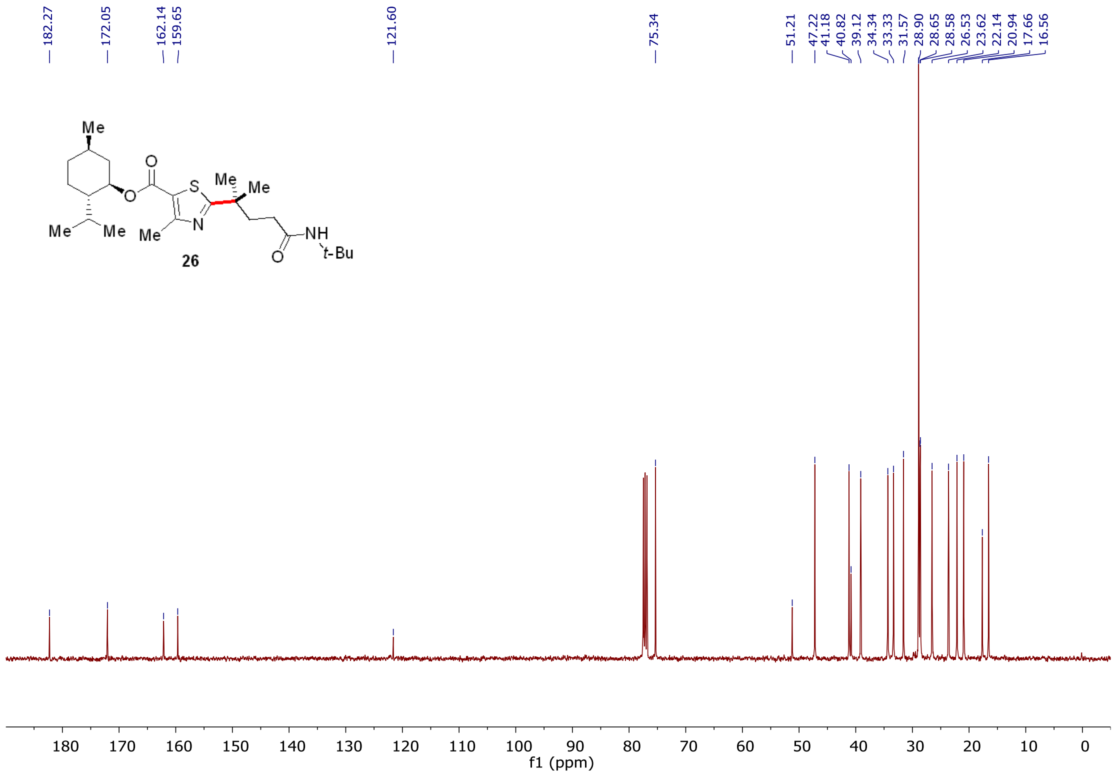


**Supplementary Figure 109.** $^{13}$C NMR spectra for **26**

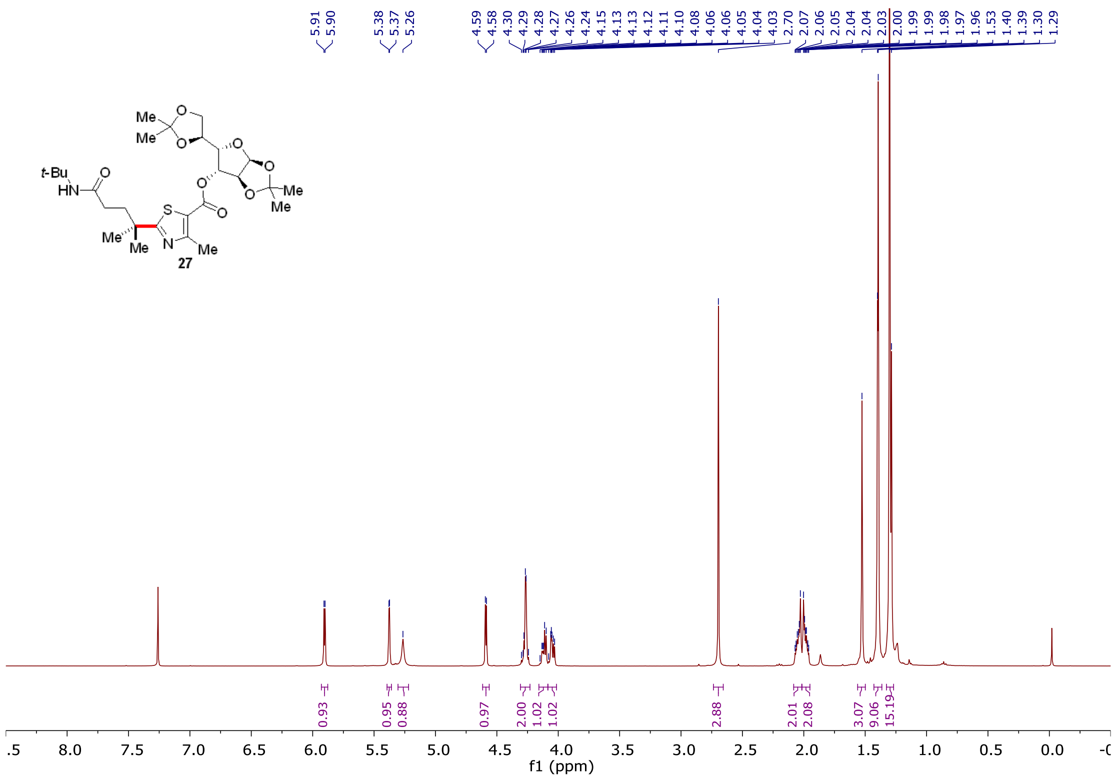

**Supplementary Figure 110.** $^{1}$H NMR spectra for **27**

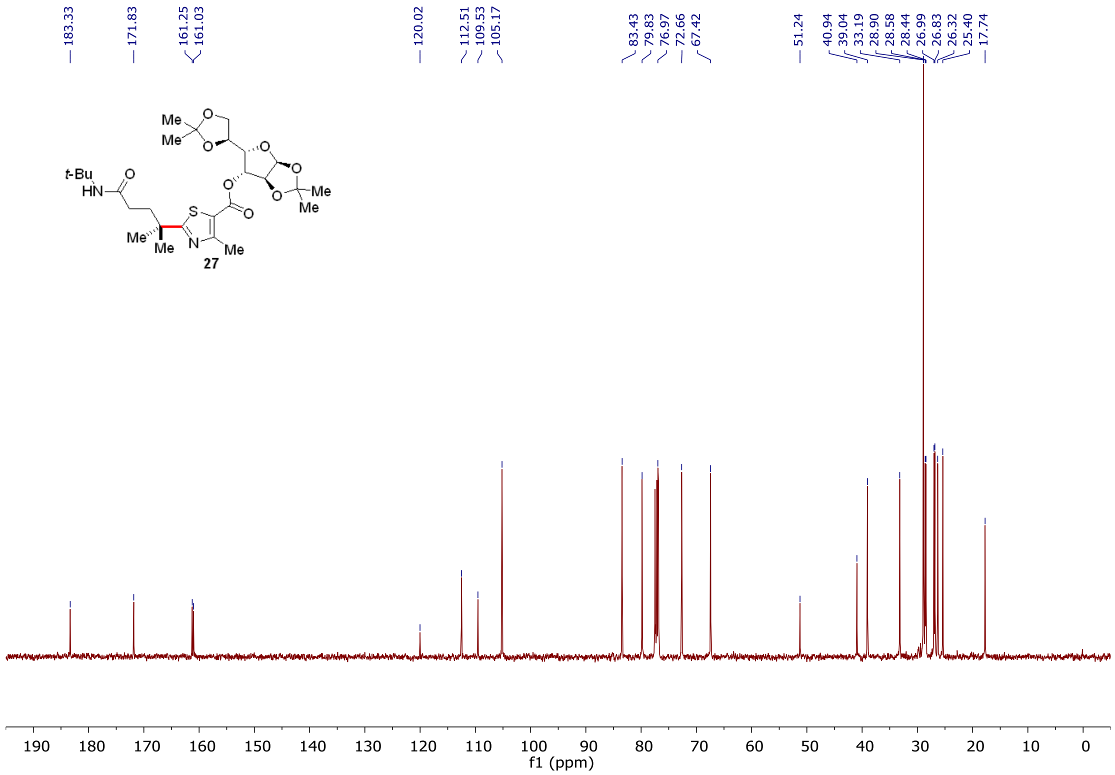

**Supplementary Figure 111.** $^{13}$C NMR spectra for **27**

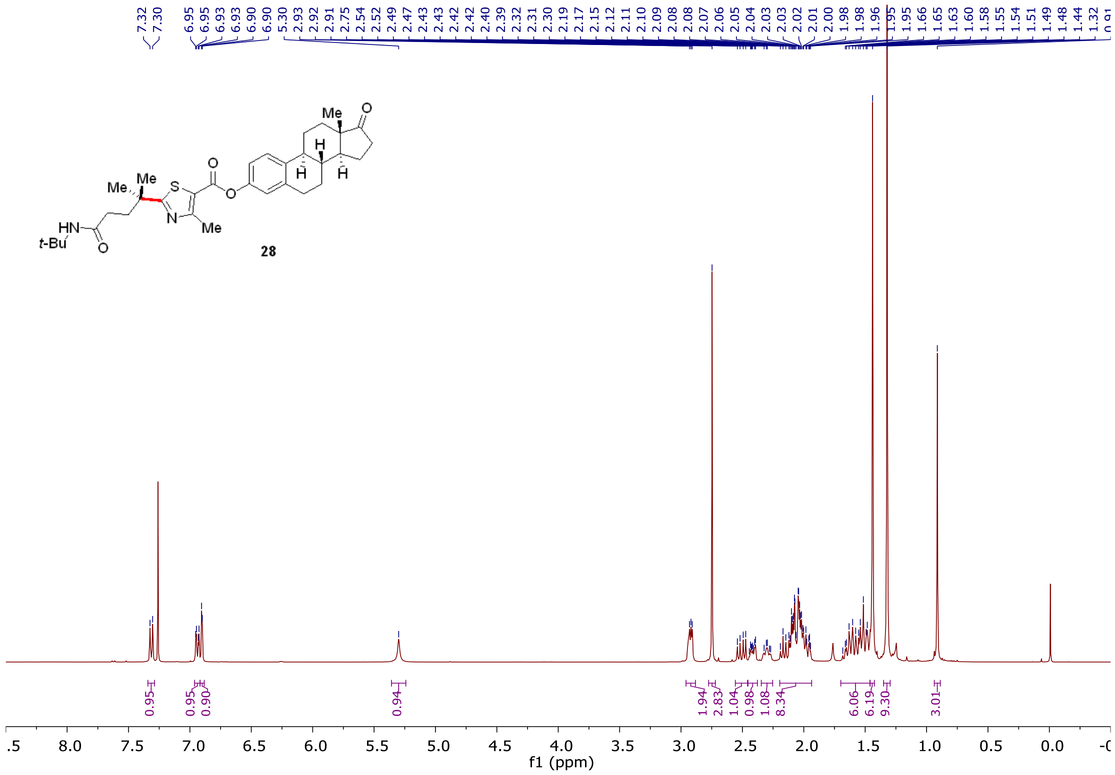

**Supplementary Figure 112.** $^{1}$H NMR spectra for **28**

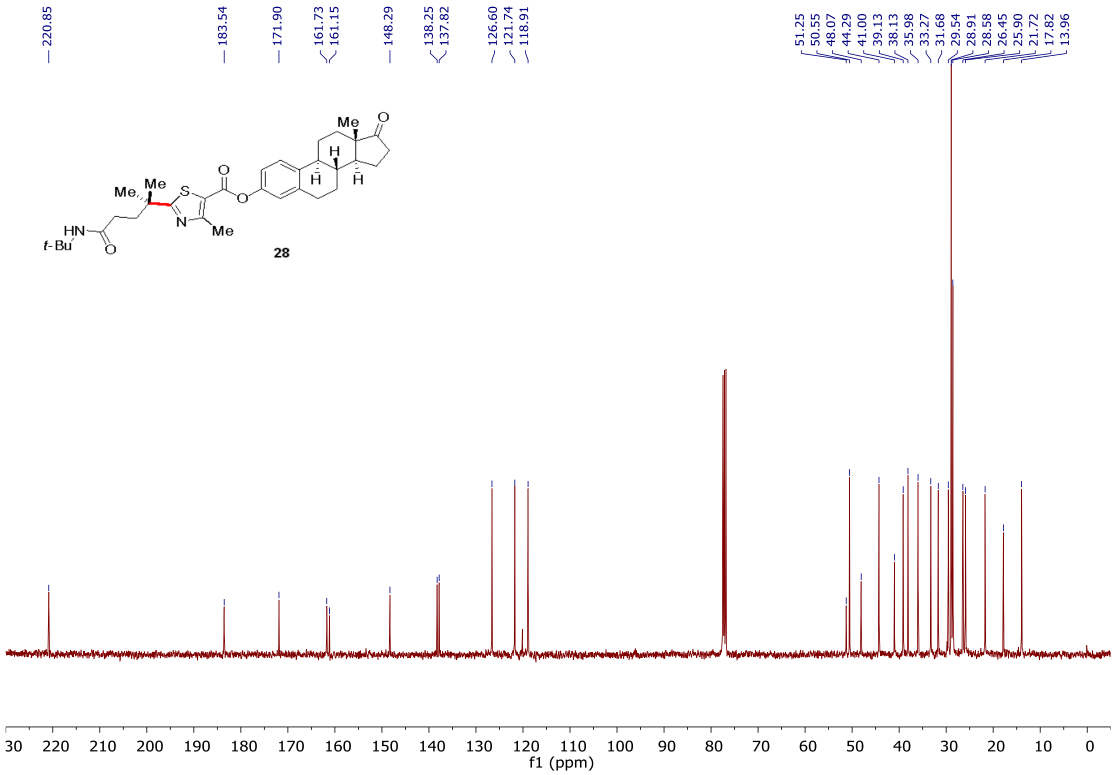

**Supplementary Figure 113.** $^{13}$C NMR spectra for **28**

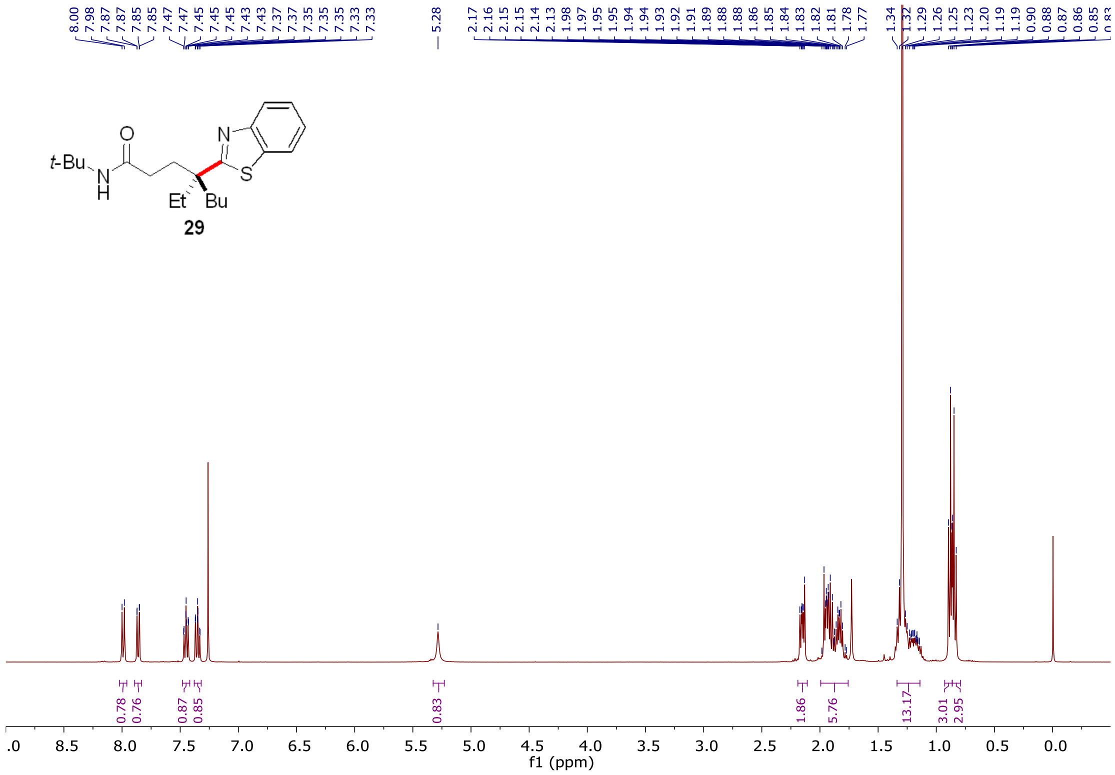


**Supplementary Figure 114.** $^{1}$H NMR spectra for **29**

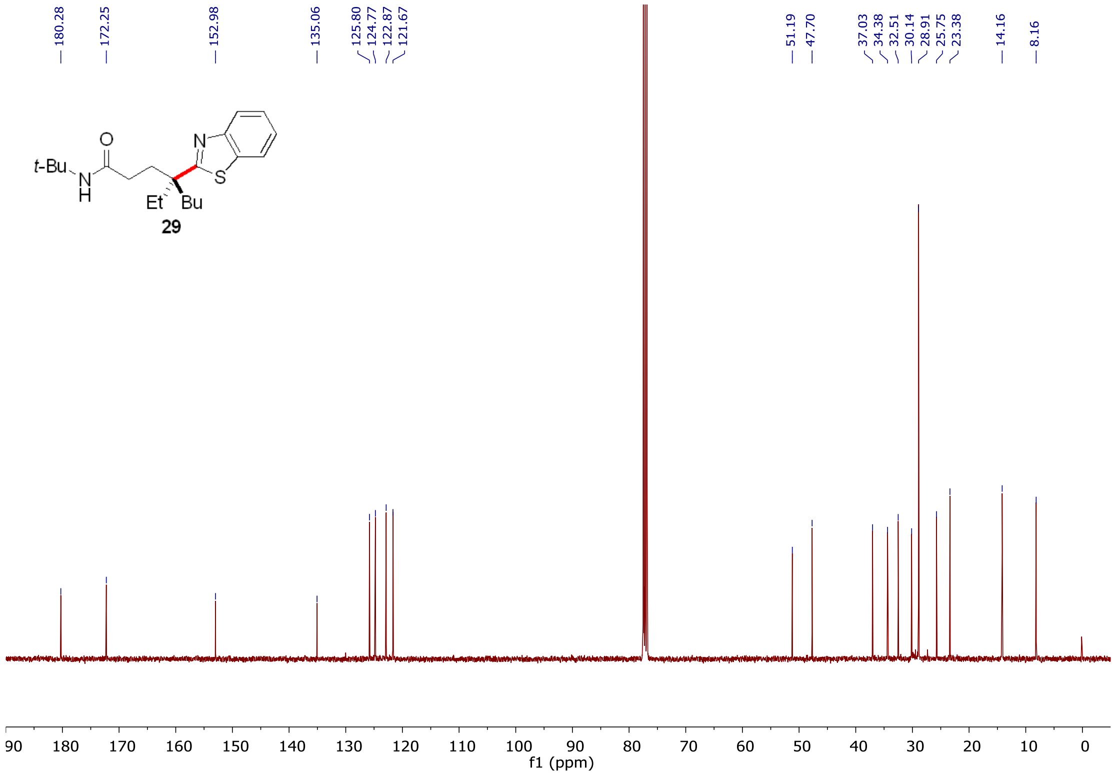


**Supplementary Figure 115.** $^{13}$C NMR spectra for **29**

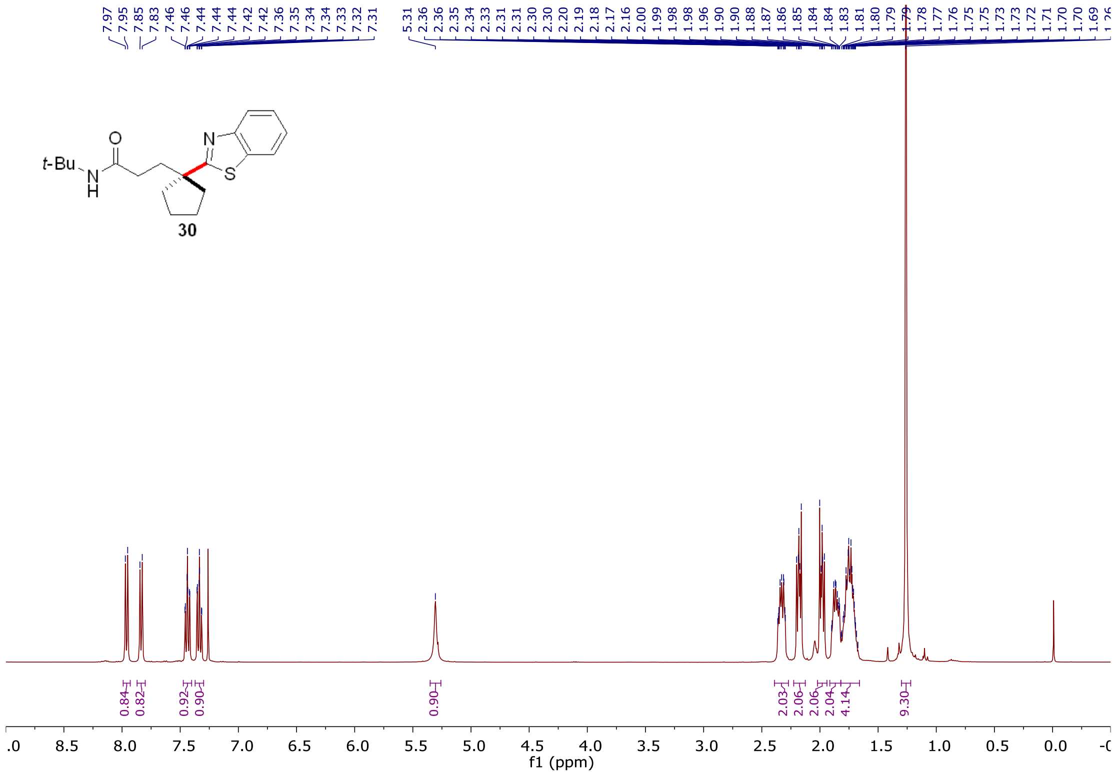


**Supplementary Figure 116.** $^{1}$H NMR spectra for **30**

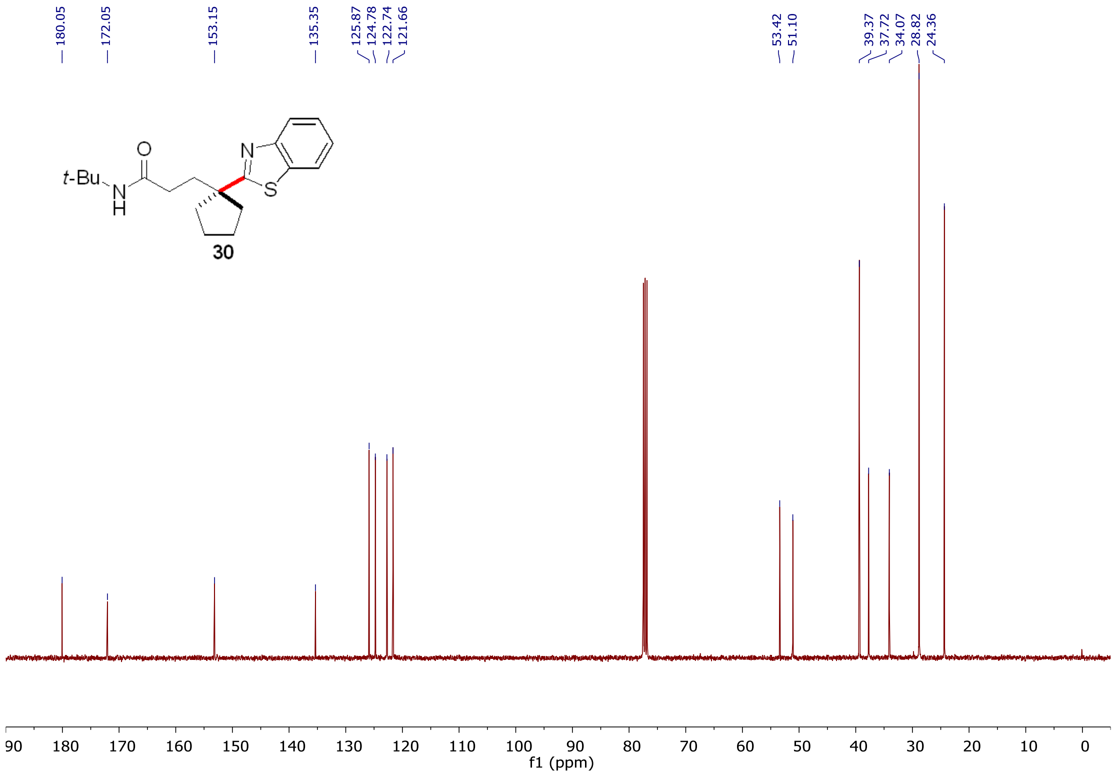


**Supplementary Figure 117.** $^{13}$C NMR spectra for **30**

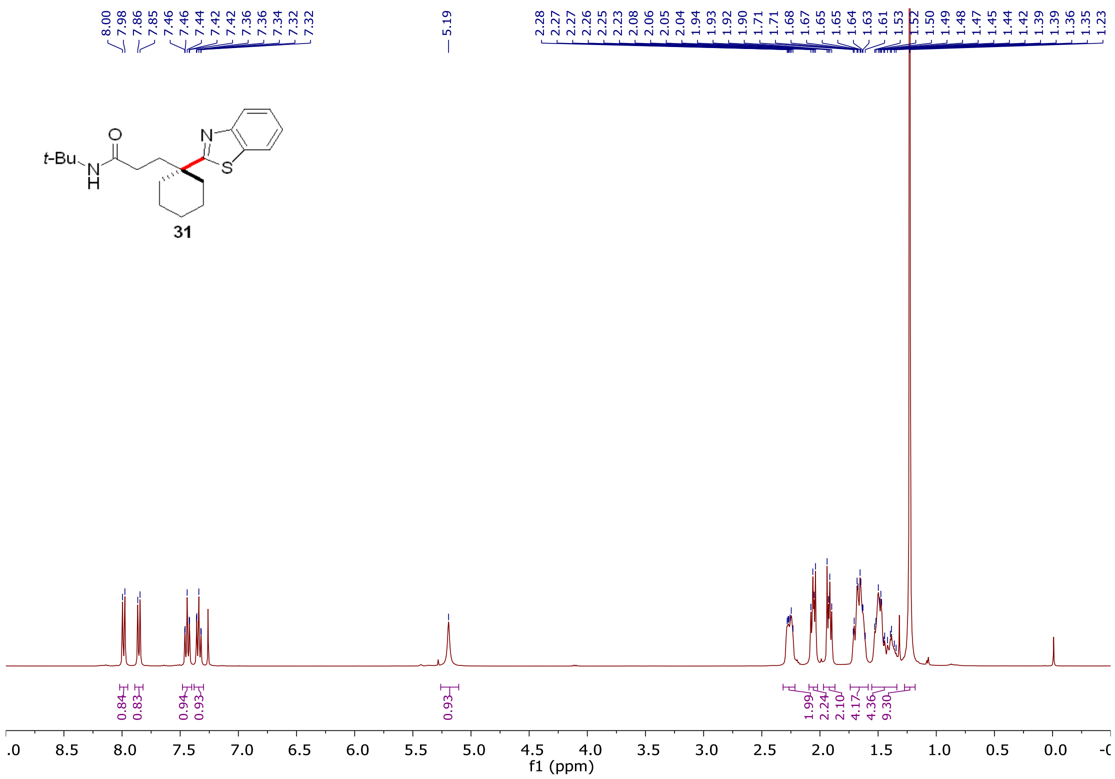

**Supplementary Figure 118.** $^{1}$H NMR spectra for **31**

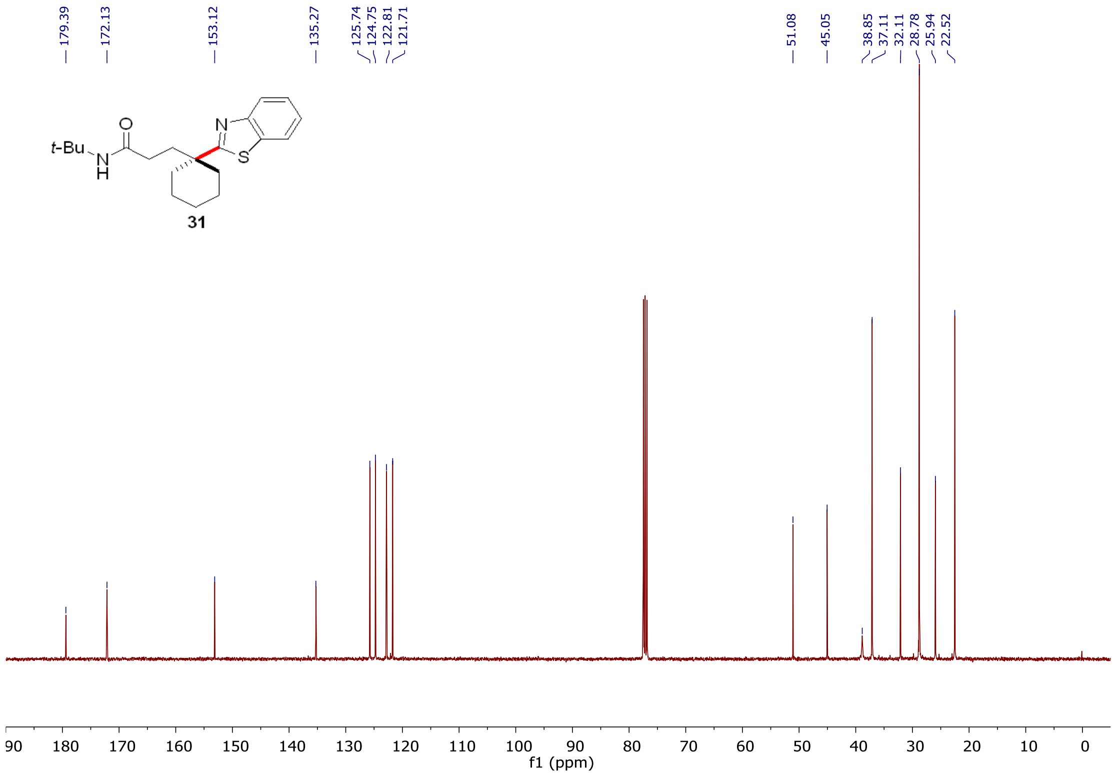

**Supplementary Figure 119.** $^{13}$C NMR spectra for **31**

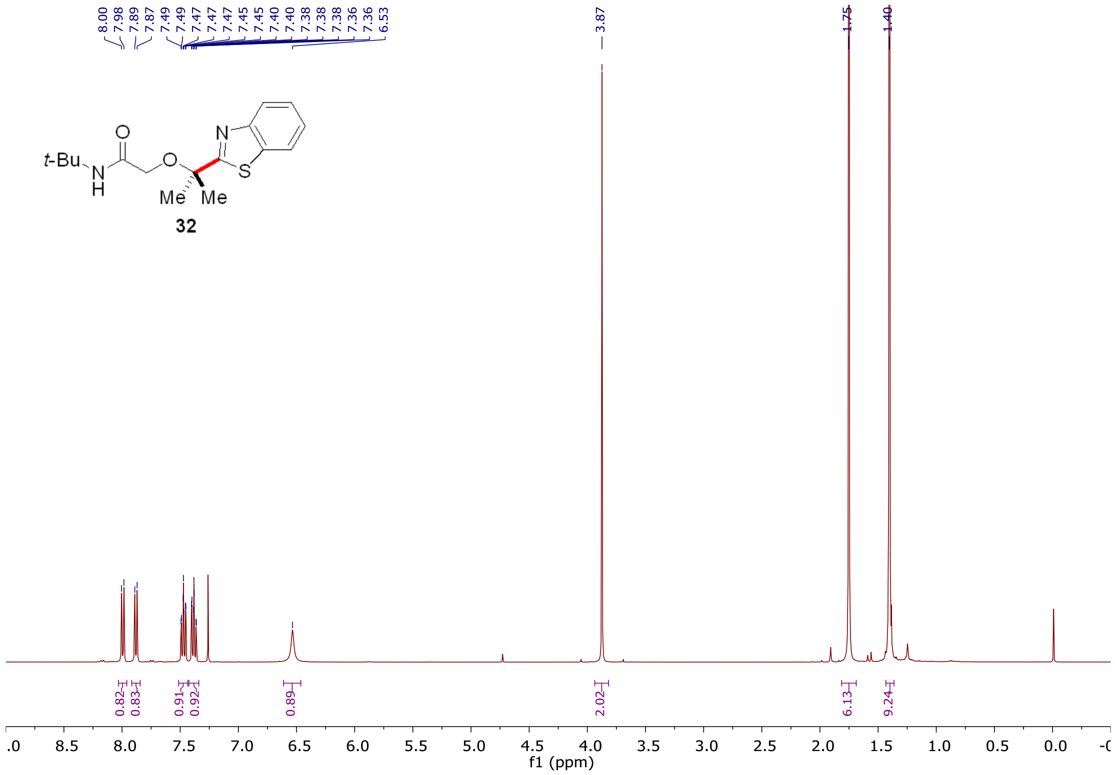


**Supplementary Figure 120.** $^{1}$H NMR spectra for **32**

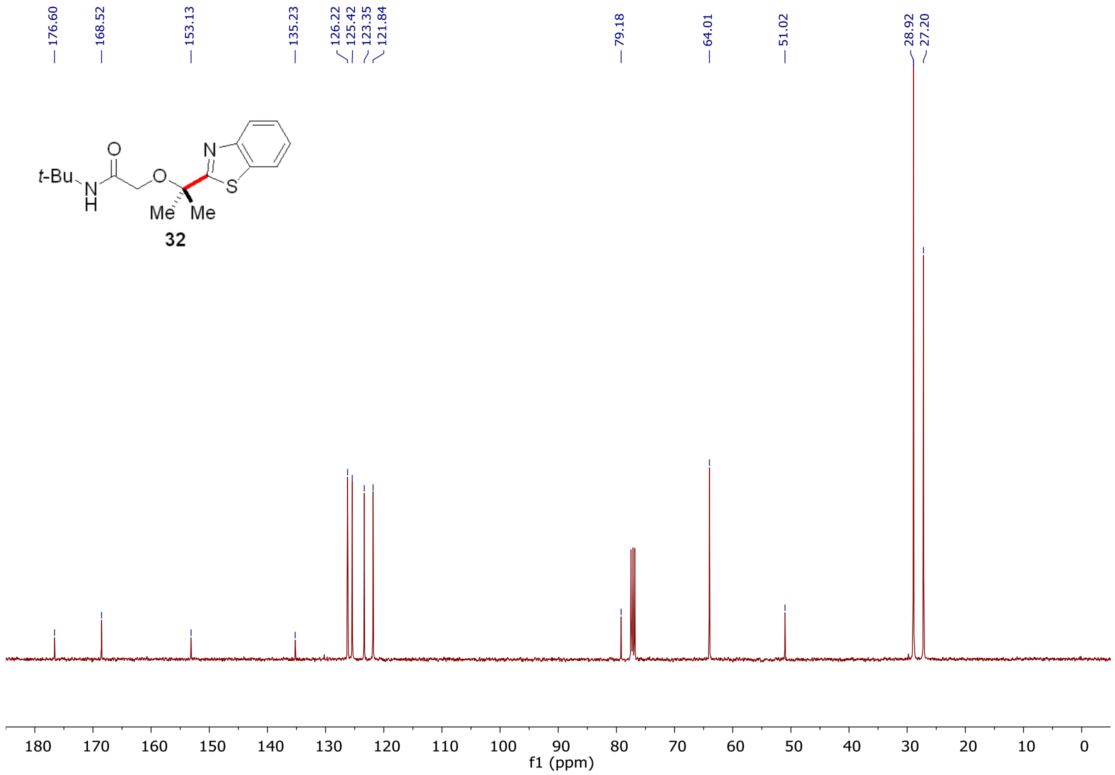


**Supplementary Figure 121.** $^{13}$C NMR spectra for **32**

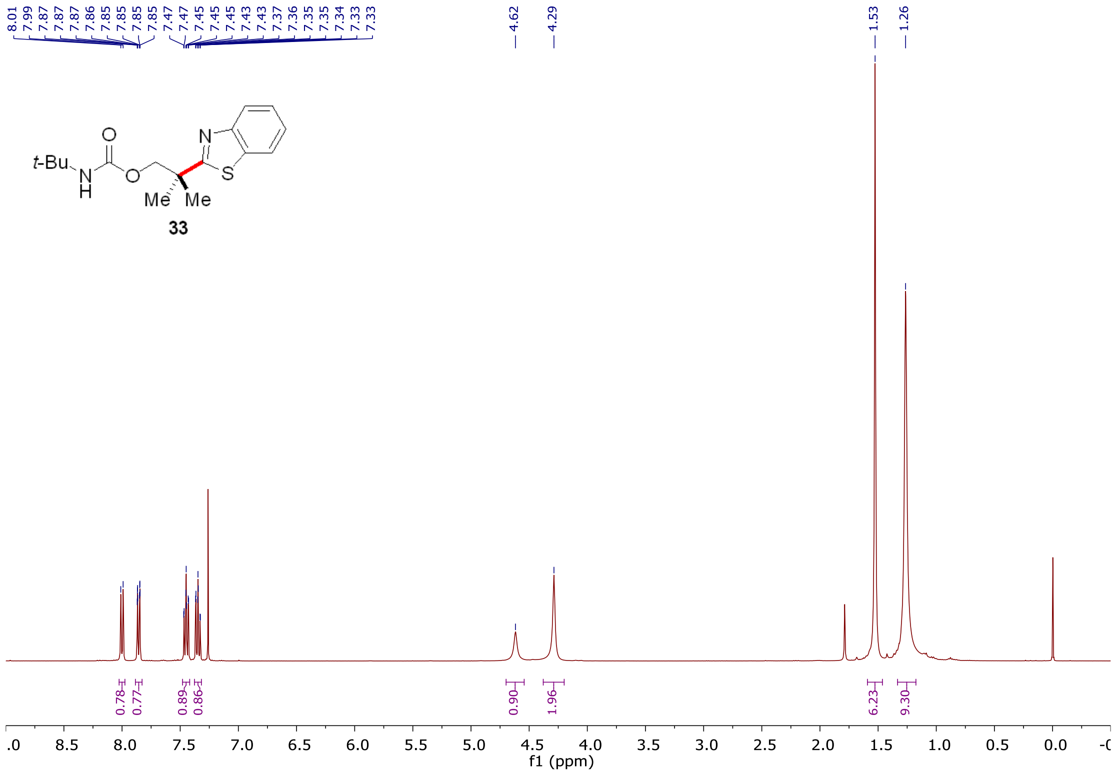

**Supplementary Figure 122.** $^{1}$H NMR spectra for **33**

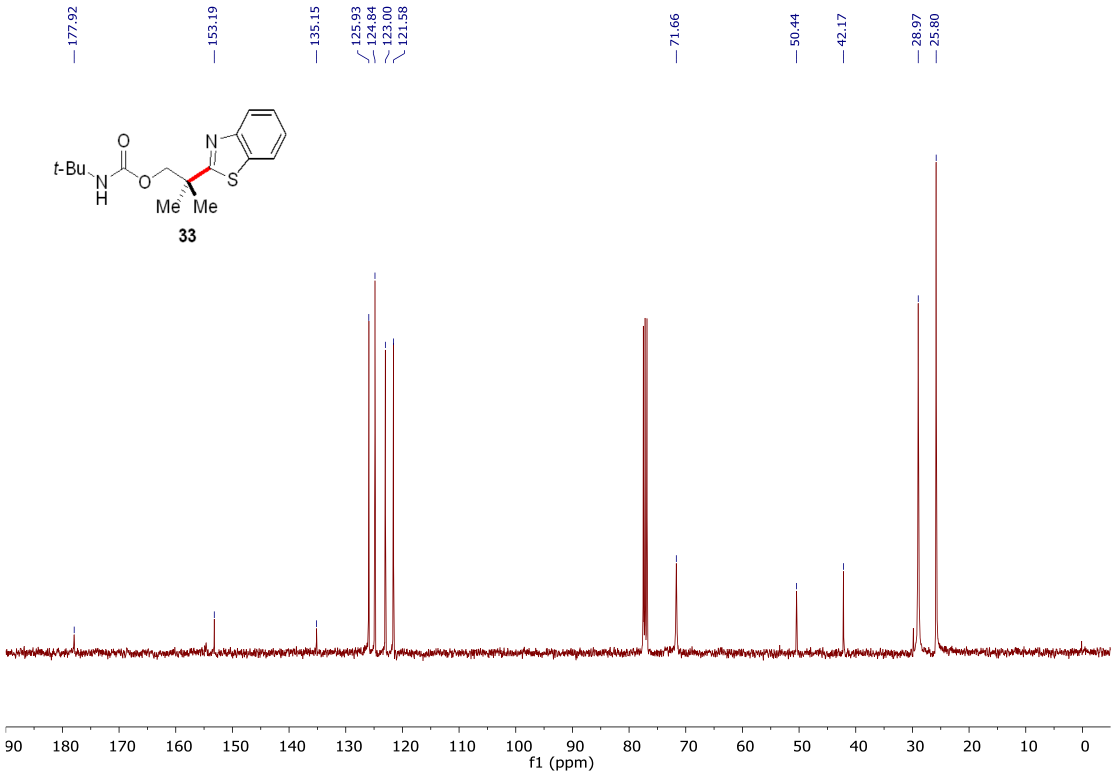

**Supplementary Figure 123.** $^{13}$C NMR spectra for **33**

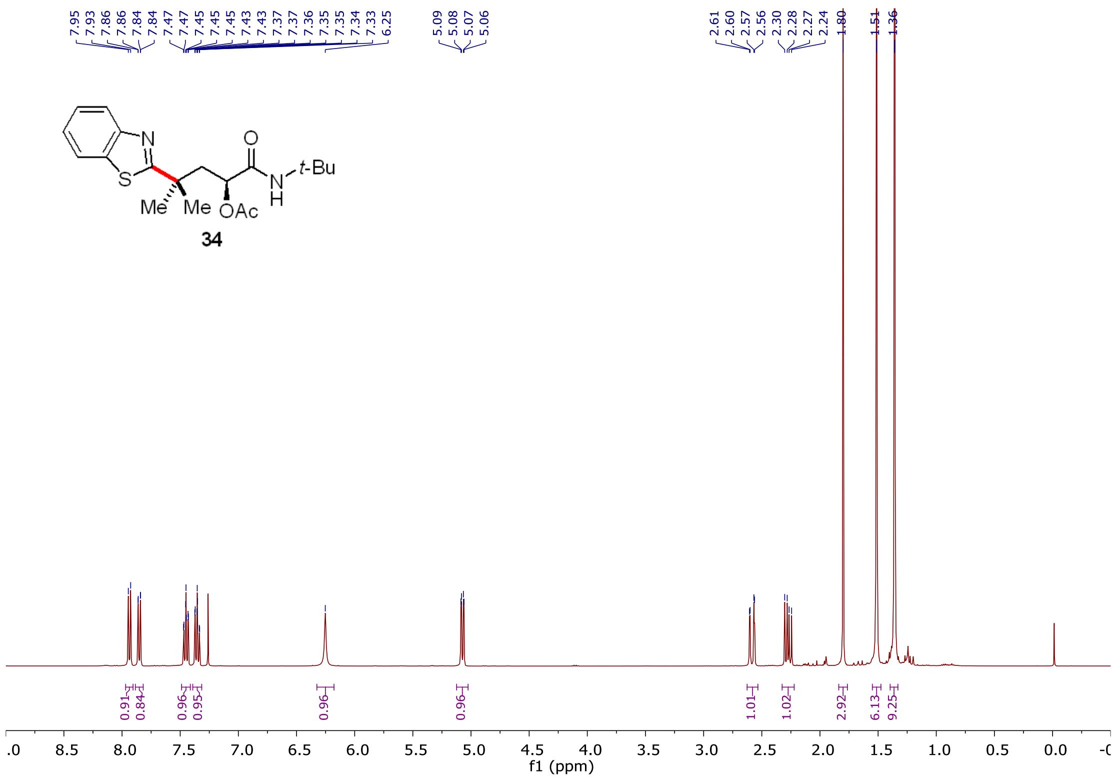

**Supplementary Figure 124.** $^{1}$H NMR spectra for **34**

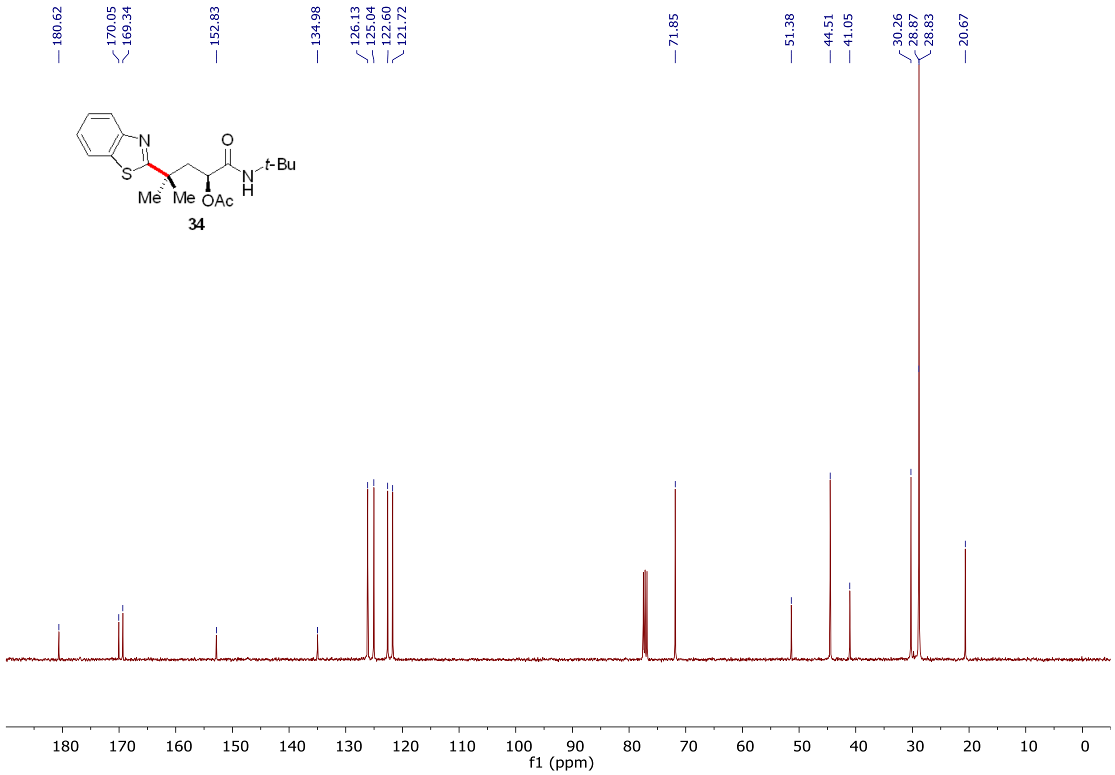

**Supplementary Figure 125.** $^{13}$C NMR spectra for **34**

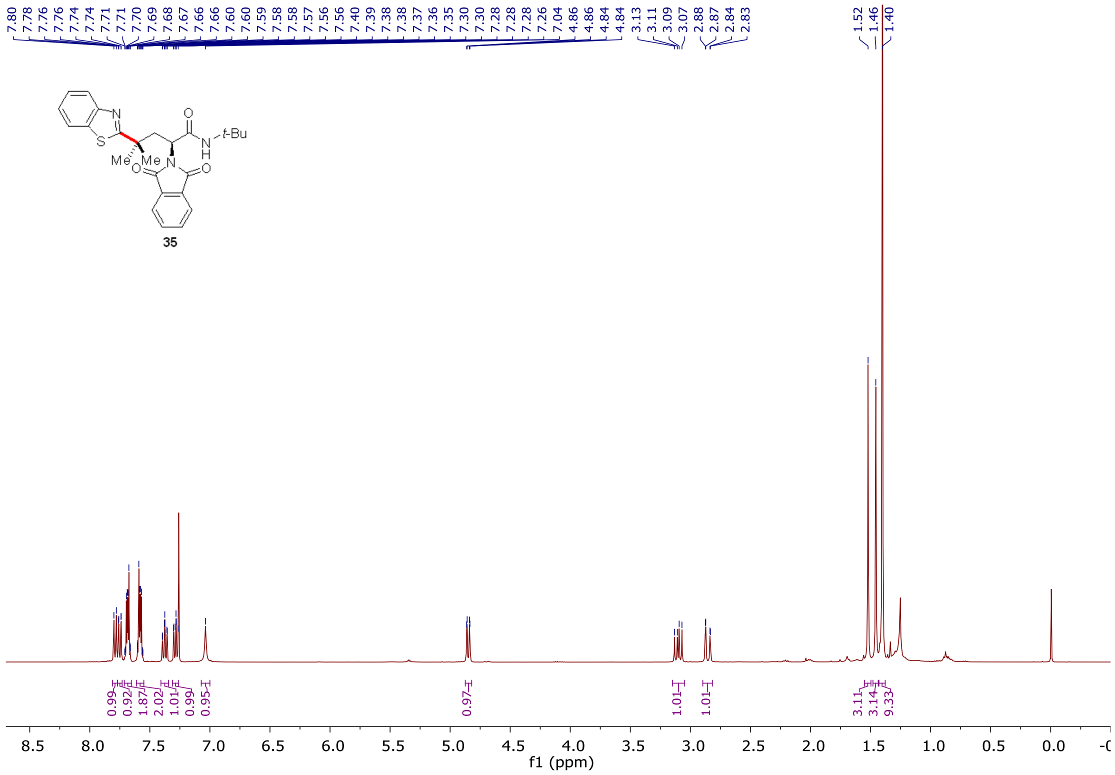

**Supplementary Figure 126.** $^{1}$H NMR spectra for **35**

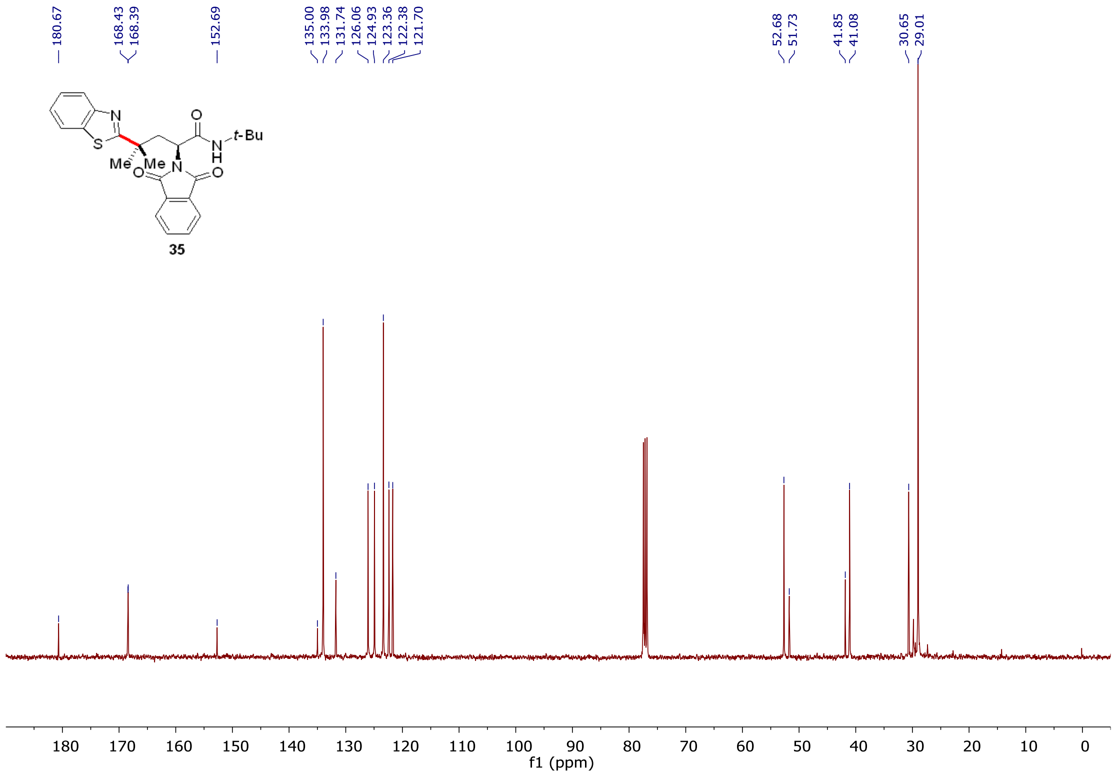

**Supplementary Figure 127.** $^{13}$C NMR spectra for **35**

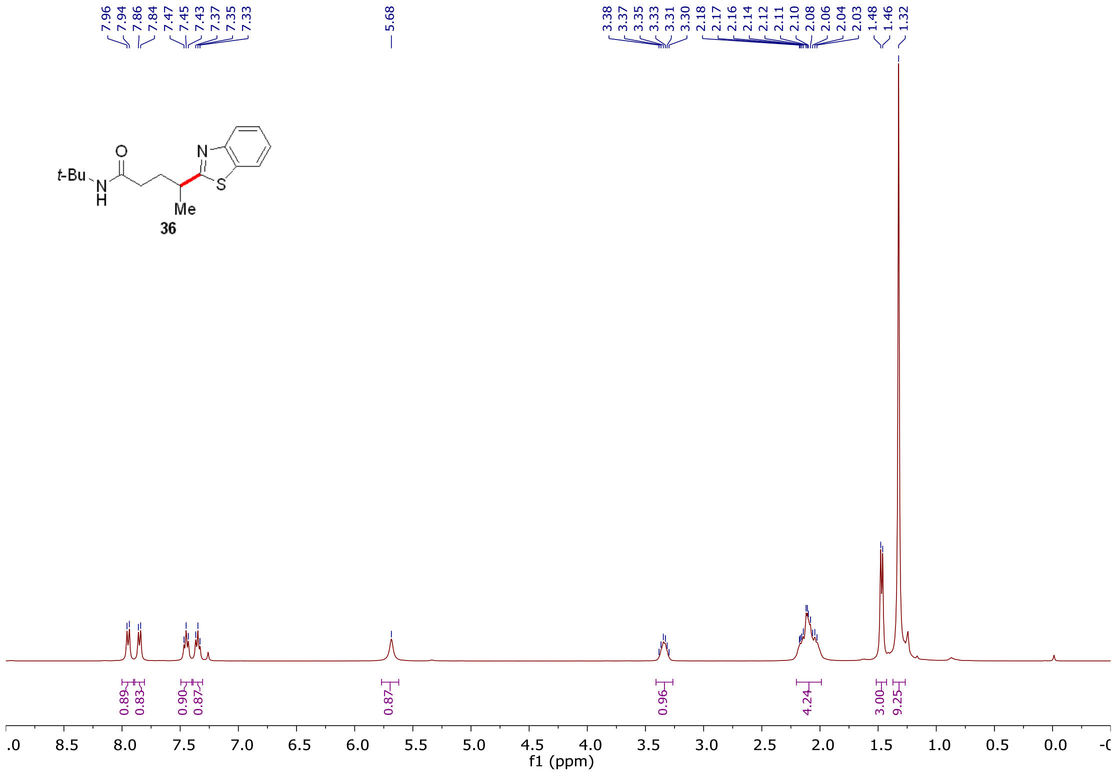


**Supplementary Figure 128.** $^{1}$H NMR spectra for **36**

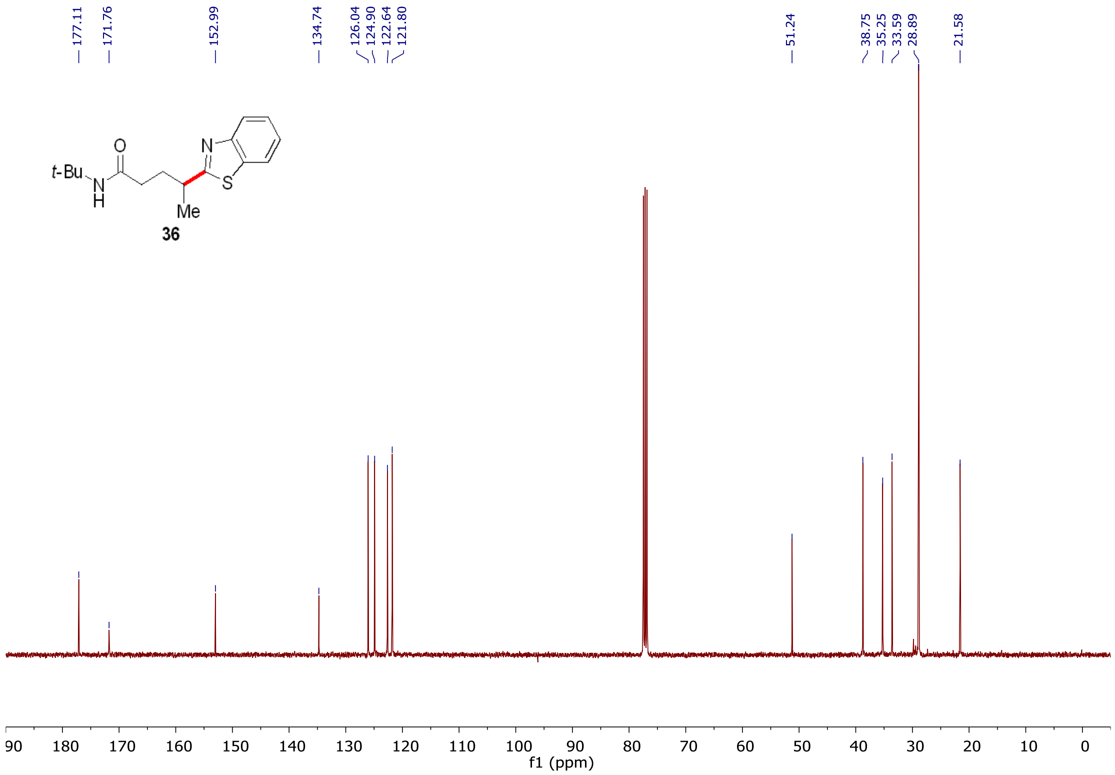


**Supplementary Figure 129.** $^{13}$C NMR spectra for **36**

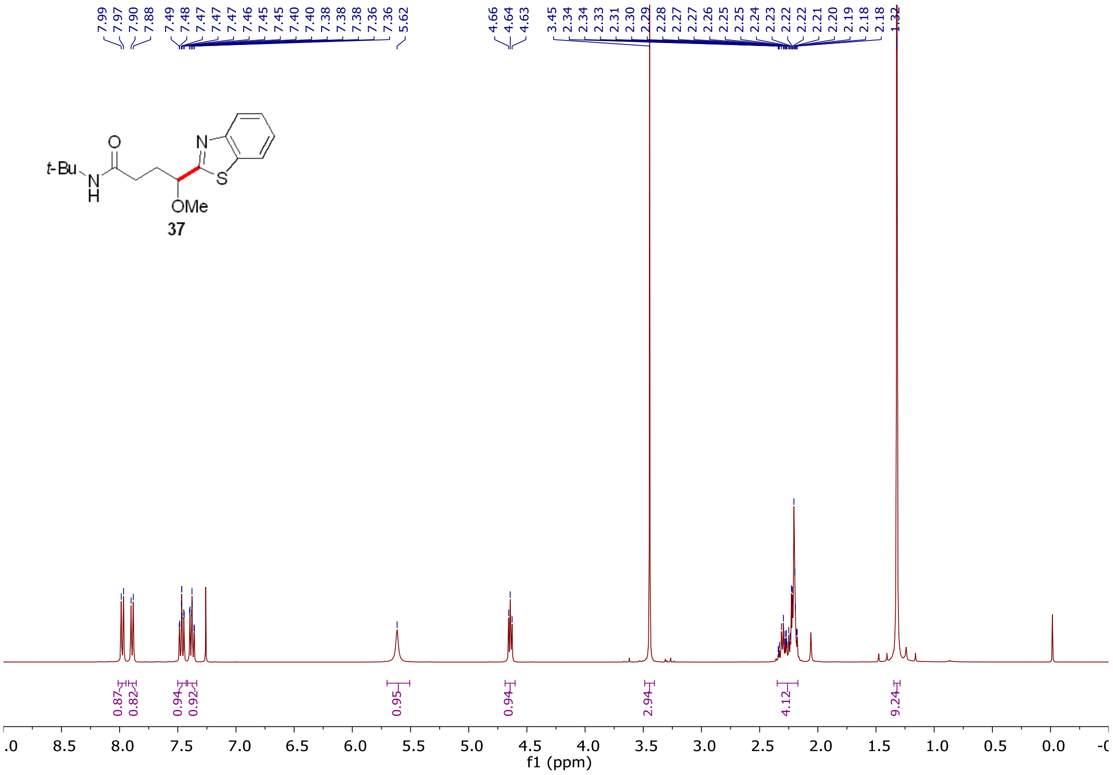

**Supplementary Figure 130.** $^{1}$H NMR spectra for **37**

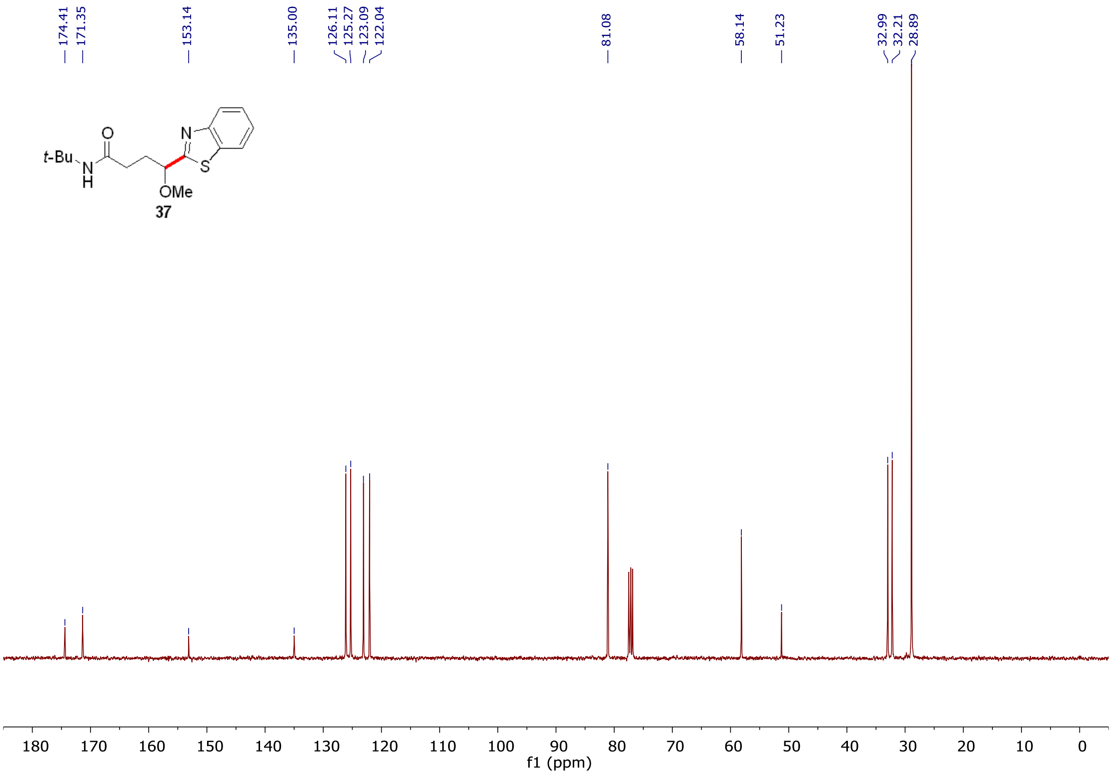

**Supplementary Figure 131.** $^{13}$C NMR spectra for **37**

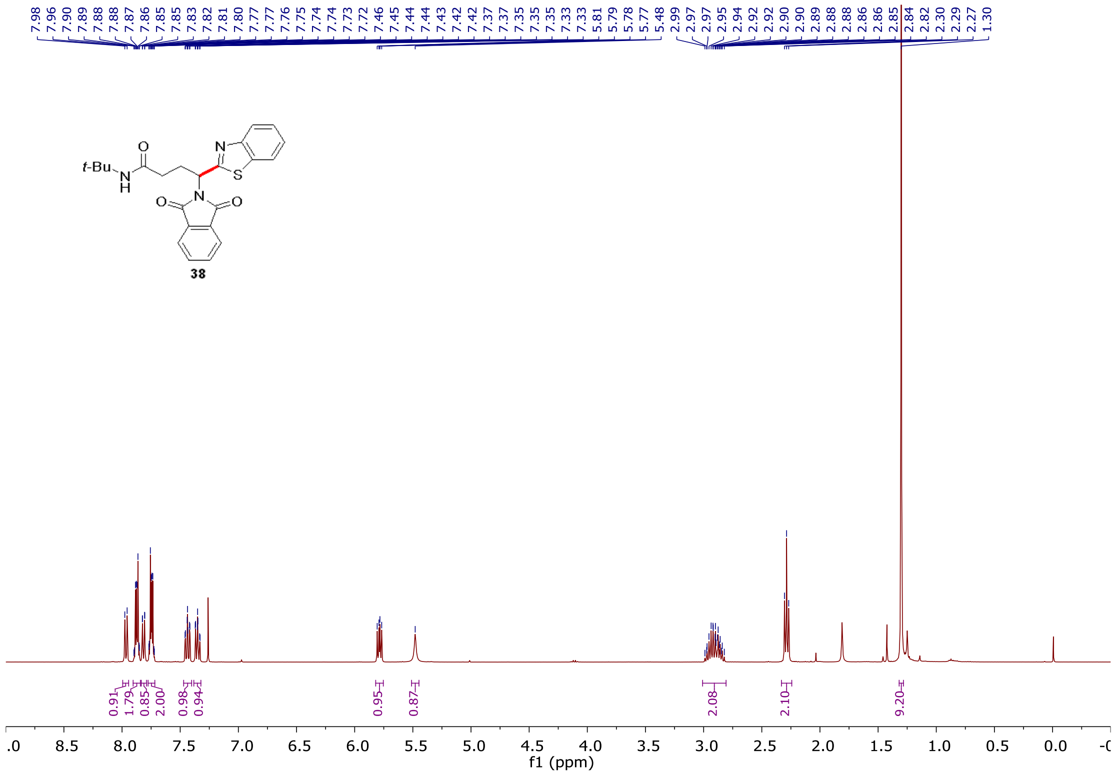


**Supplementary Figure 132.** $^{1}$H NMR spectra for **38**

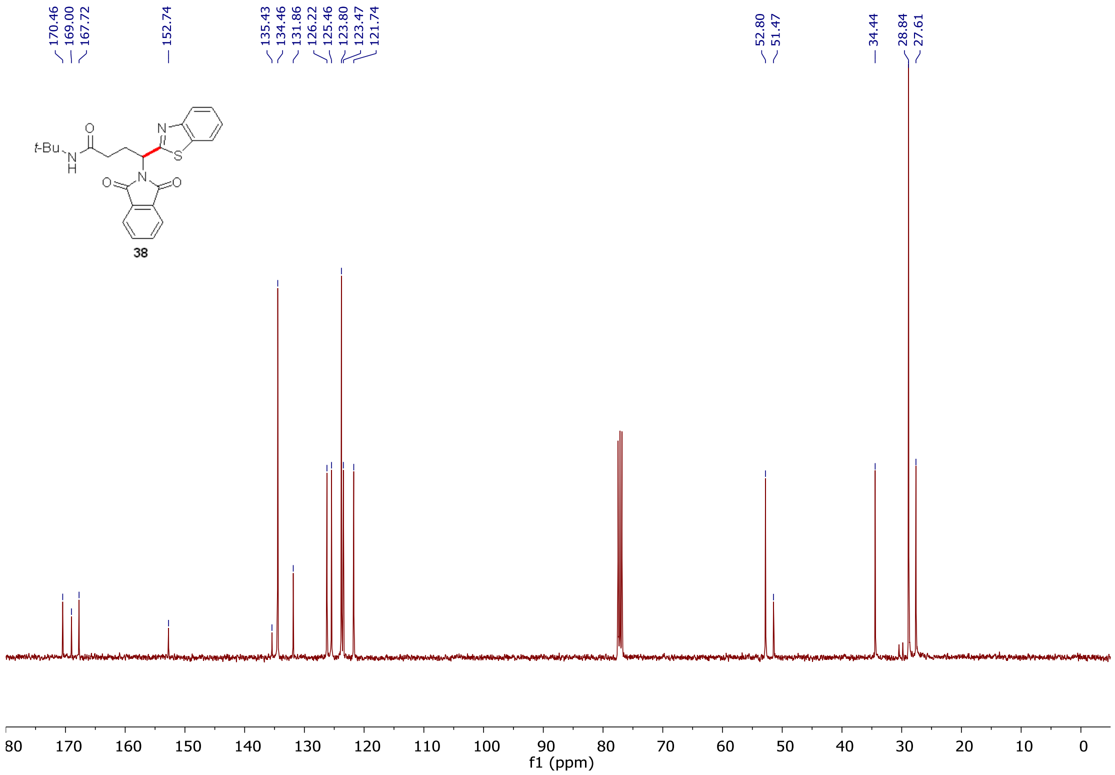


**Supplementary Figure 133.** $^{13}$C NMR spectra for **38**

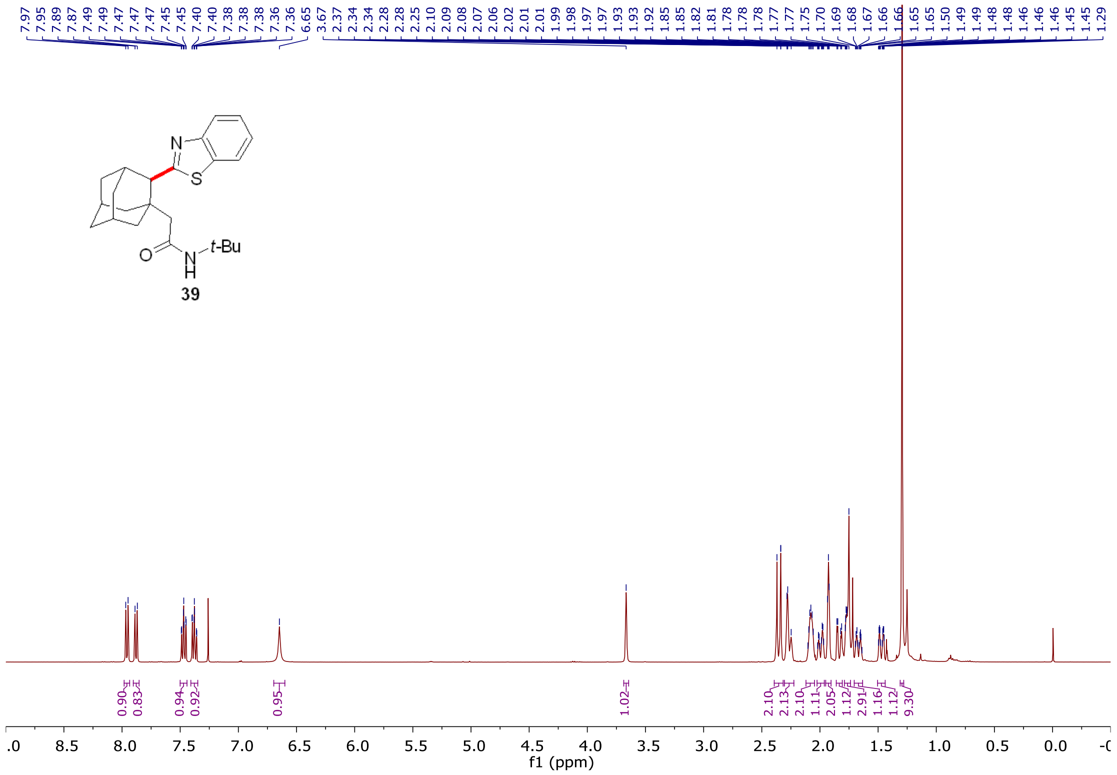


**Supplementary Figure 134.** $^{1}$H NMR spectra for **39**

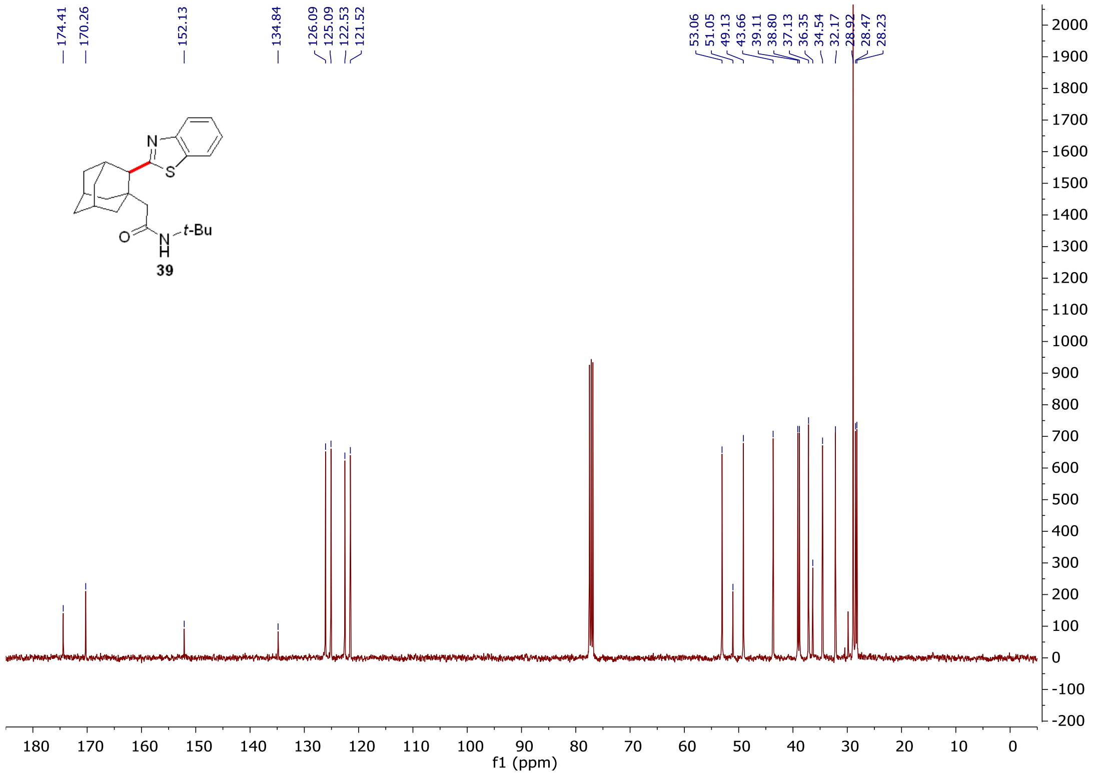


**Supplementary Figure 135.** $^{13}$C NMR spectra for **39**

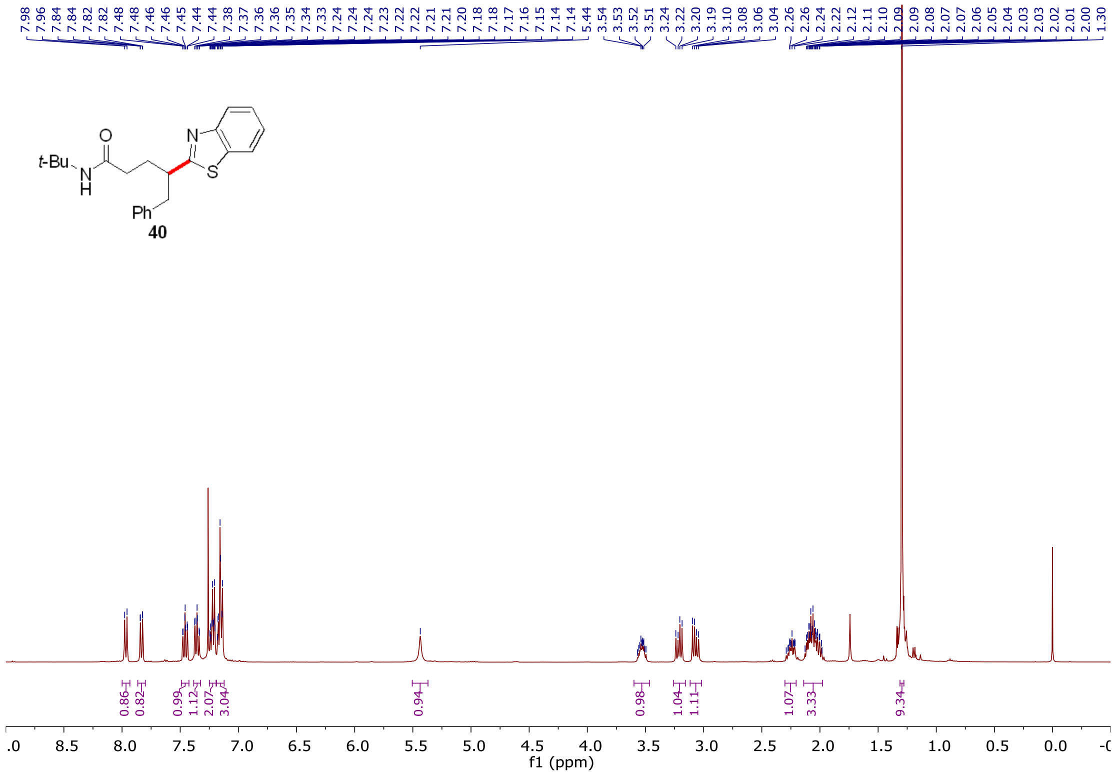


**Supplementary Figure 136.** $^{1}$H NMR spectra for **40**

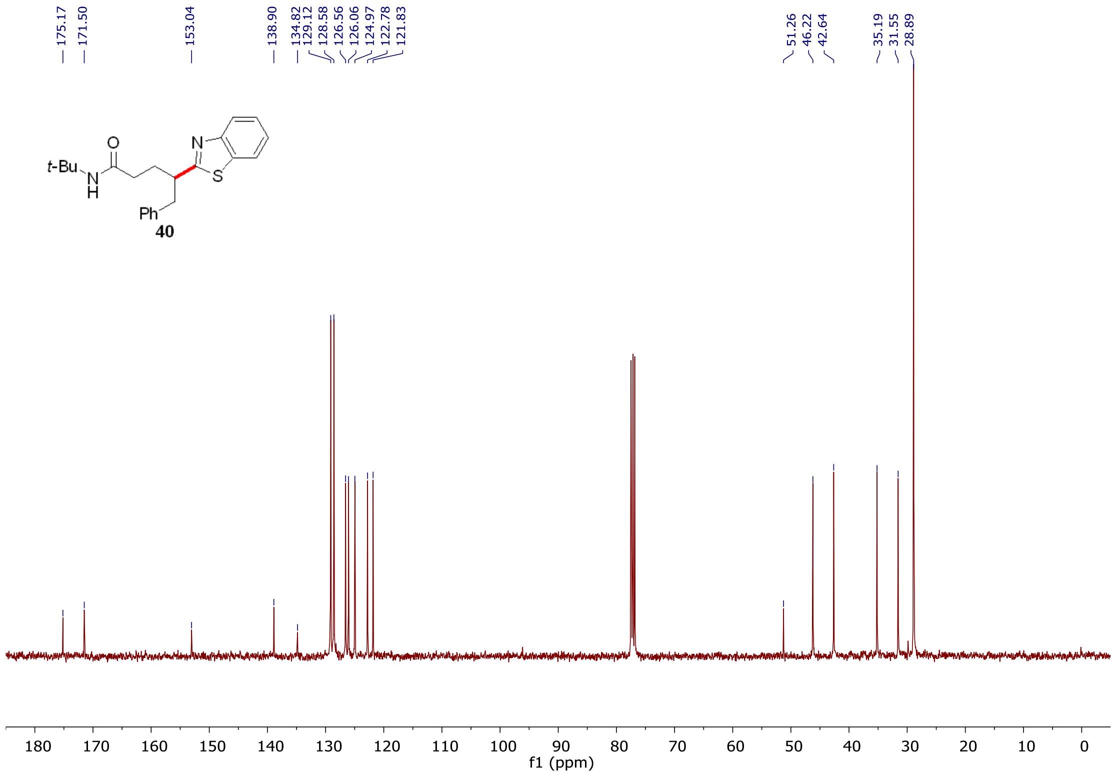


**Supplementary Figure 137.** $^{13}$C NMR spectra for **40**

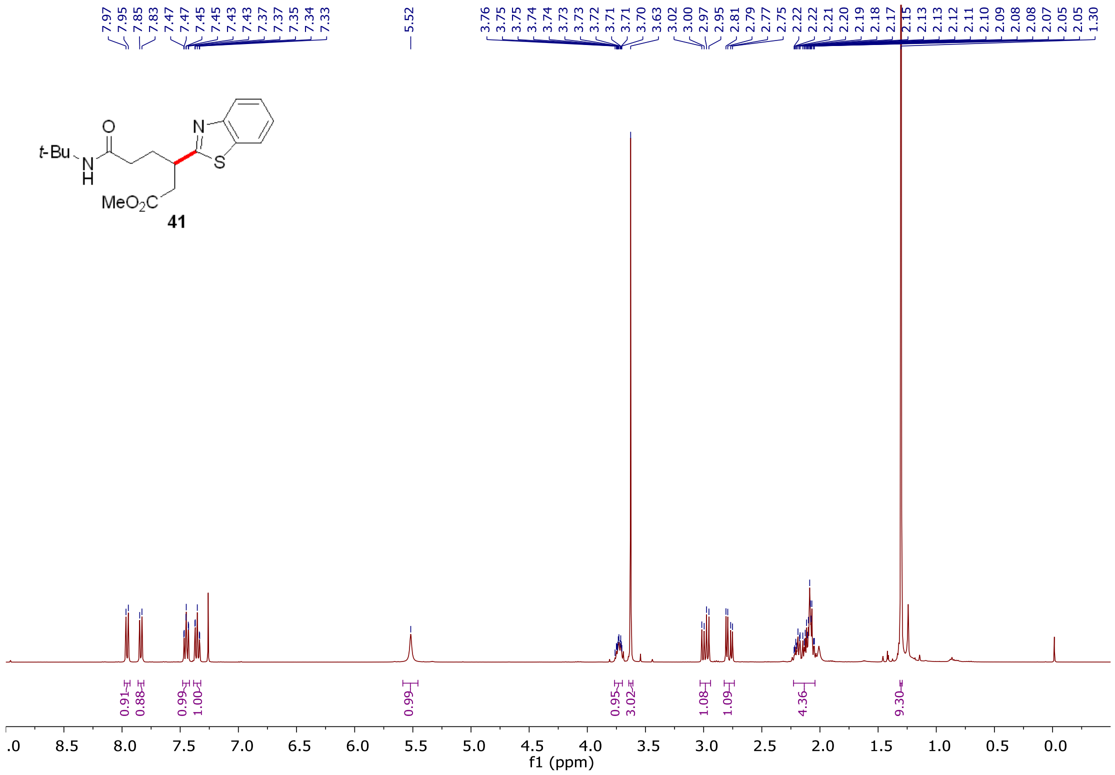


**Supplementary Figure 138.** $^{1}$H NMR spectra for **41**

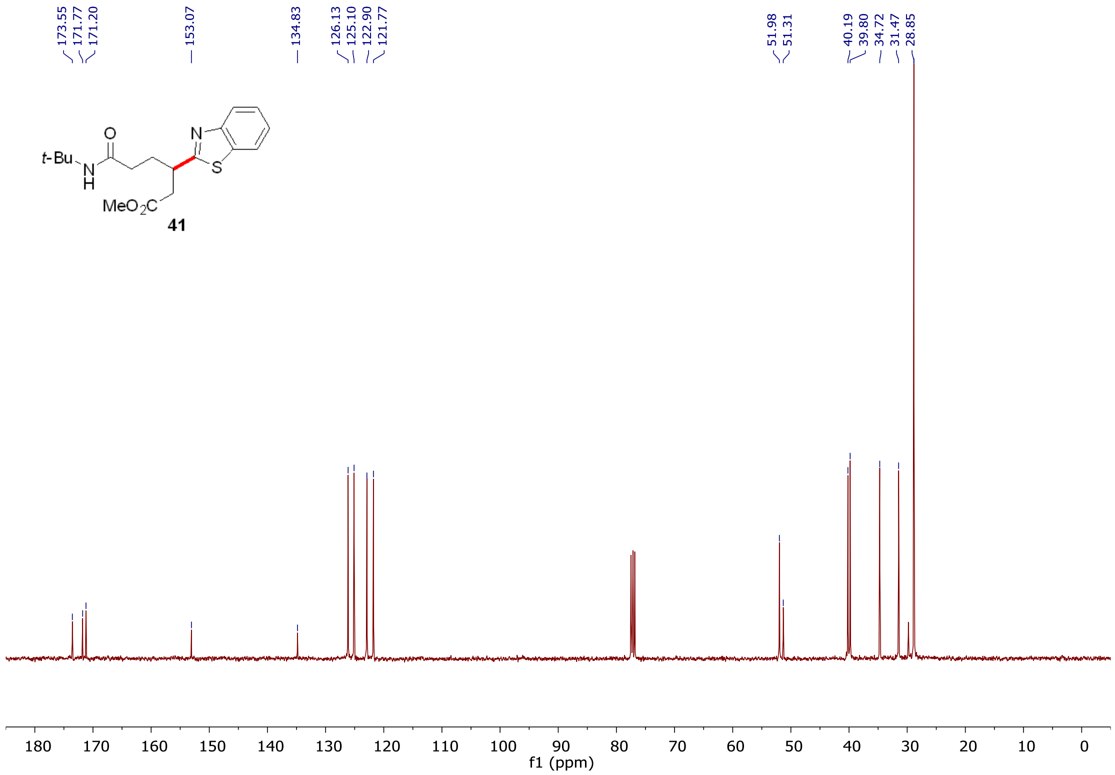


**Supplementary Figure 139.** $^{13}$C NMR spectra for **41**

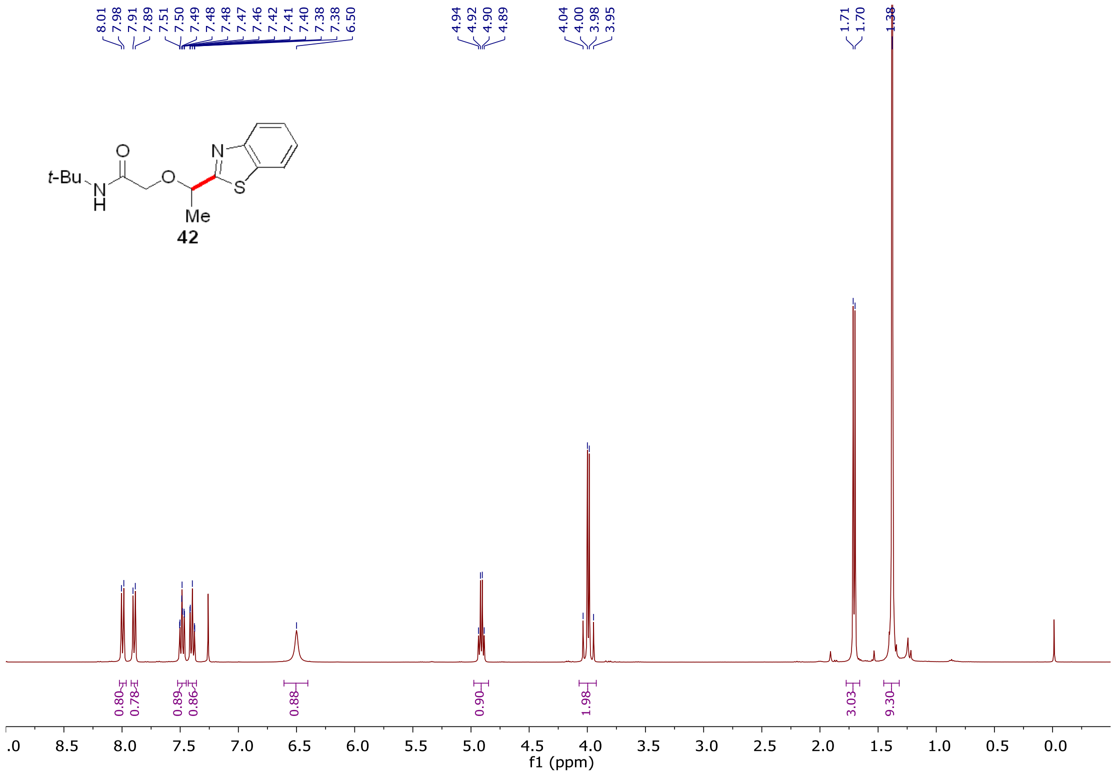

**Supplementary Figure 140.** $^{1}$H NMR spectra for **42**

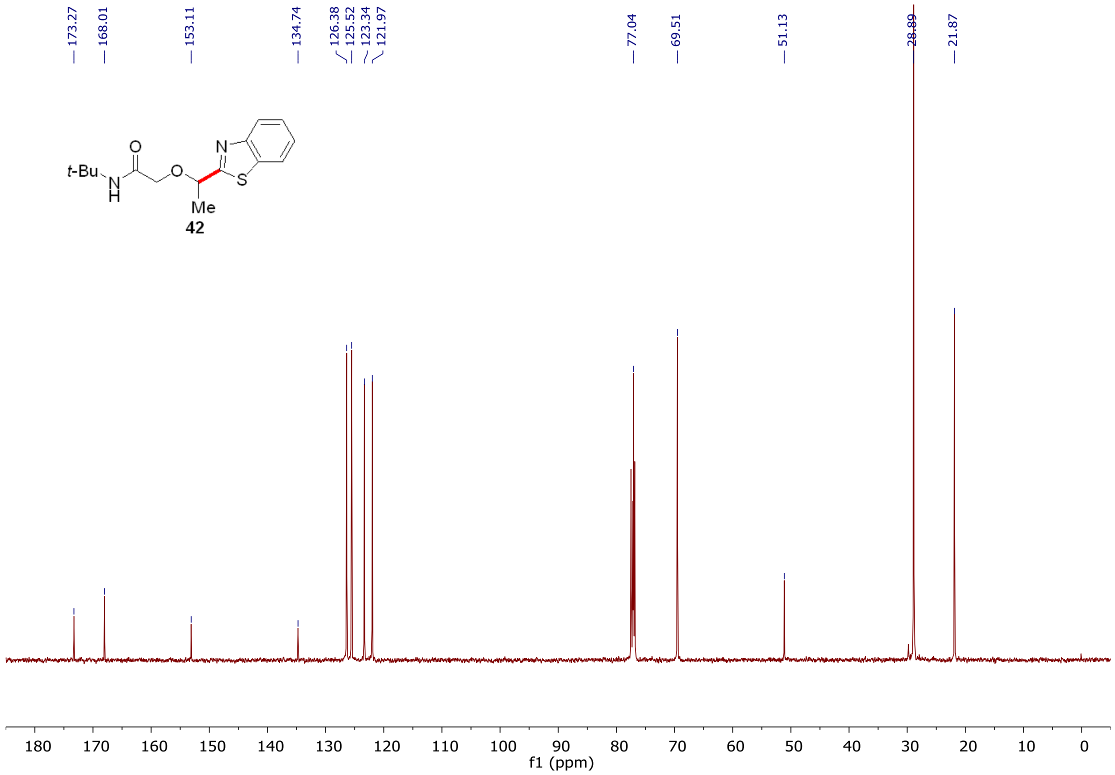

**Supplementary Figure 141.** $^{13}$C NMR spectra for **42**

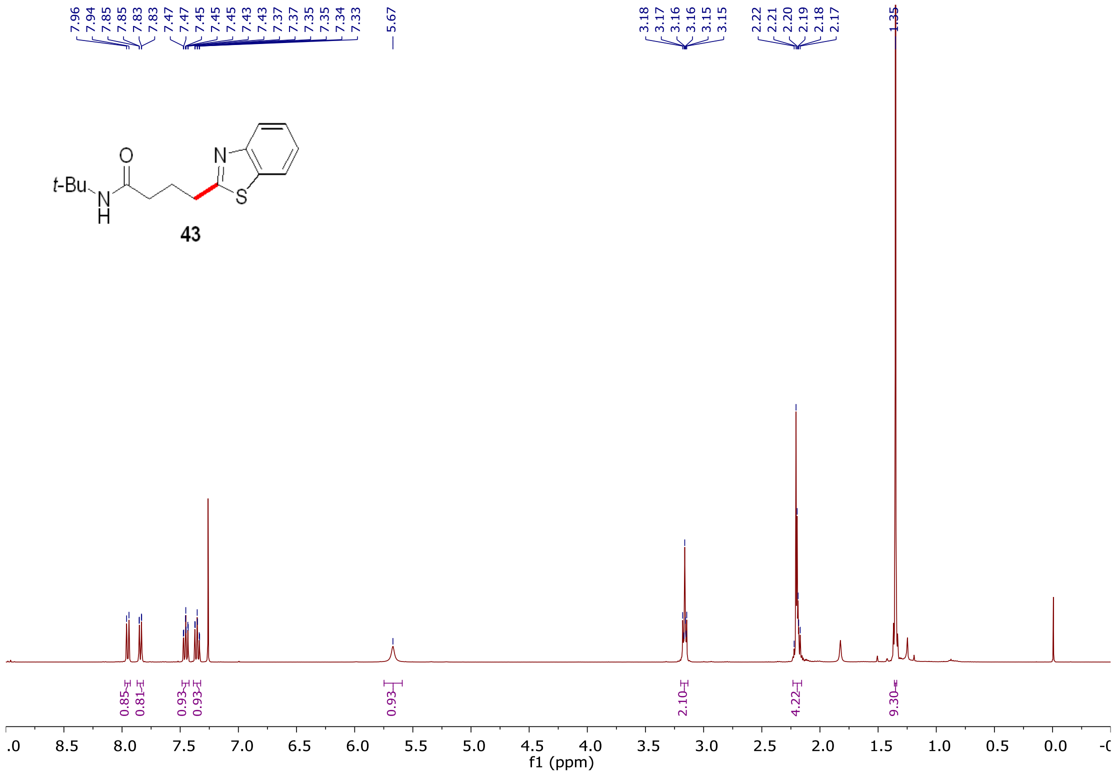


**Supplementary Figure 142.** $^{1}$H NMR spectra for **43**

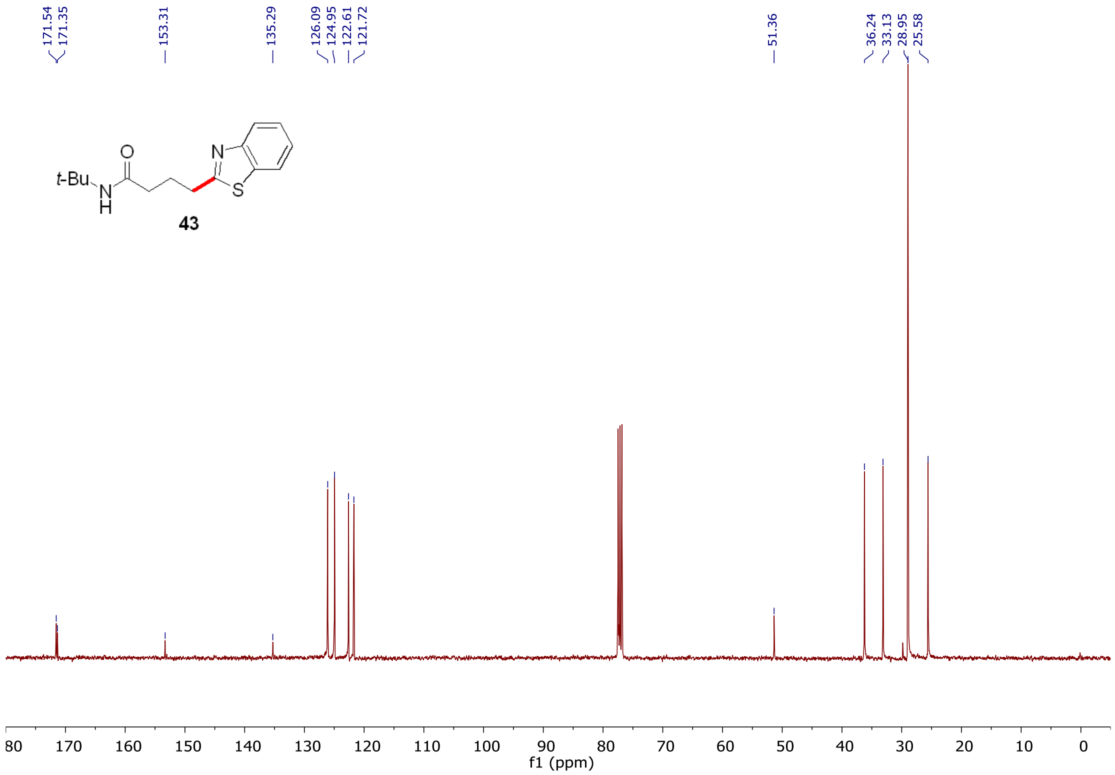


**Supplementary Figure 143.** $^{13}$C NMR spectra for **43**

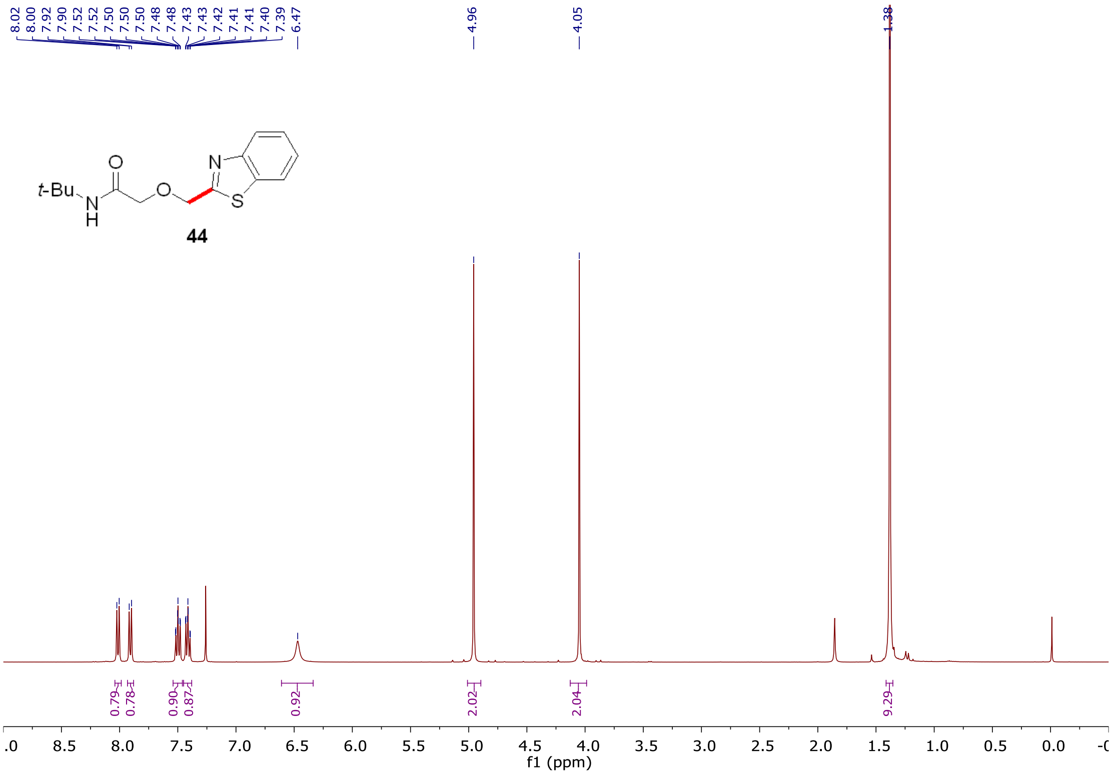


**Supplementary Figure 144.** $^{1}$H NMR spectra for **44**

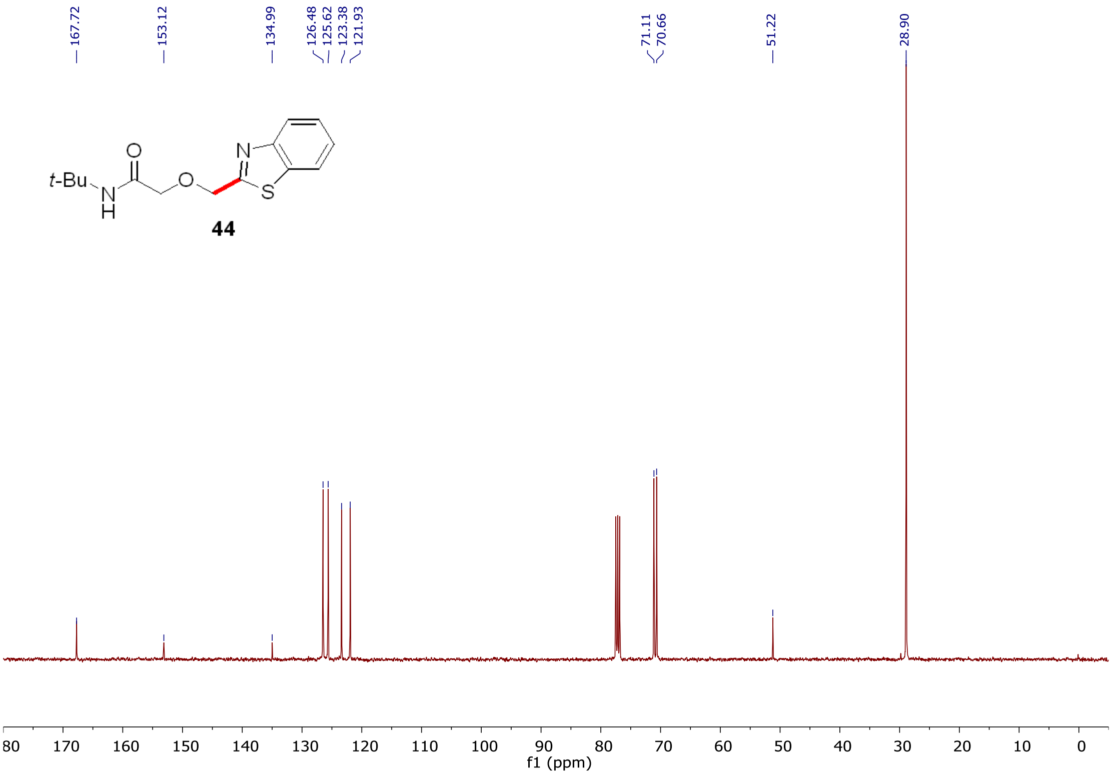


**Supplementary Figure 145.** $^{13}$C NMR spectra for **44**

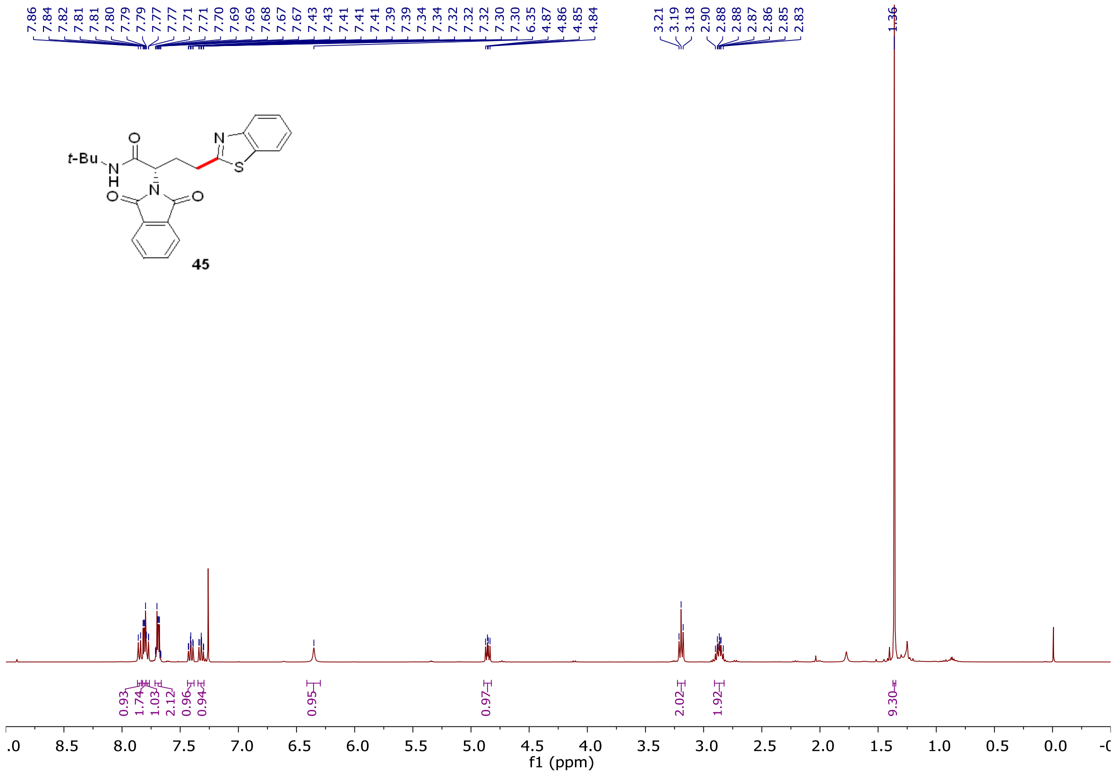

**Supplementary Figure 146.** $^{1}$H NMR spectra for **45**

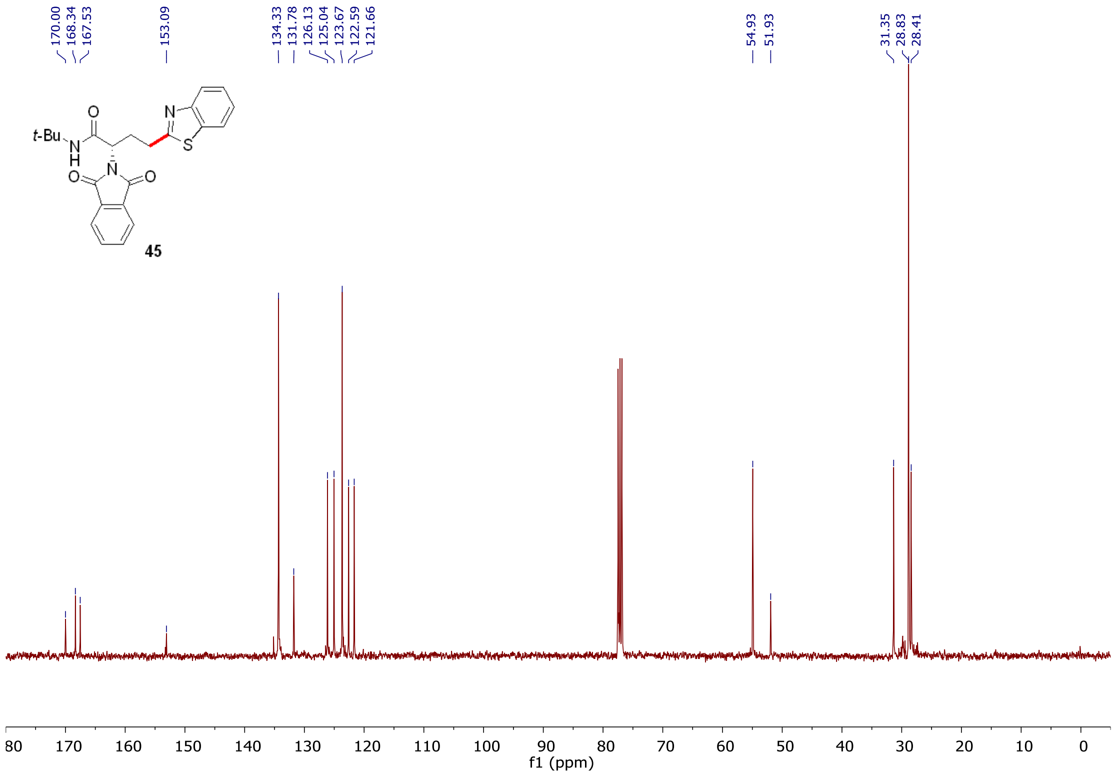

**Supplementary Figure 147.** $^{13}$C NMR spectra for **45**

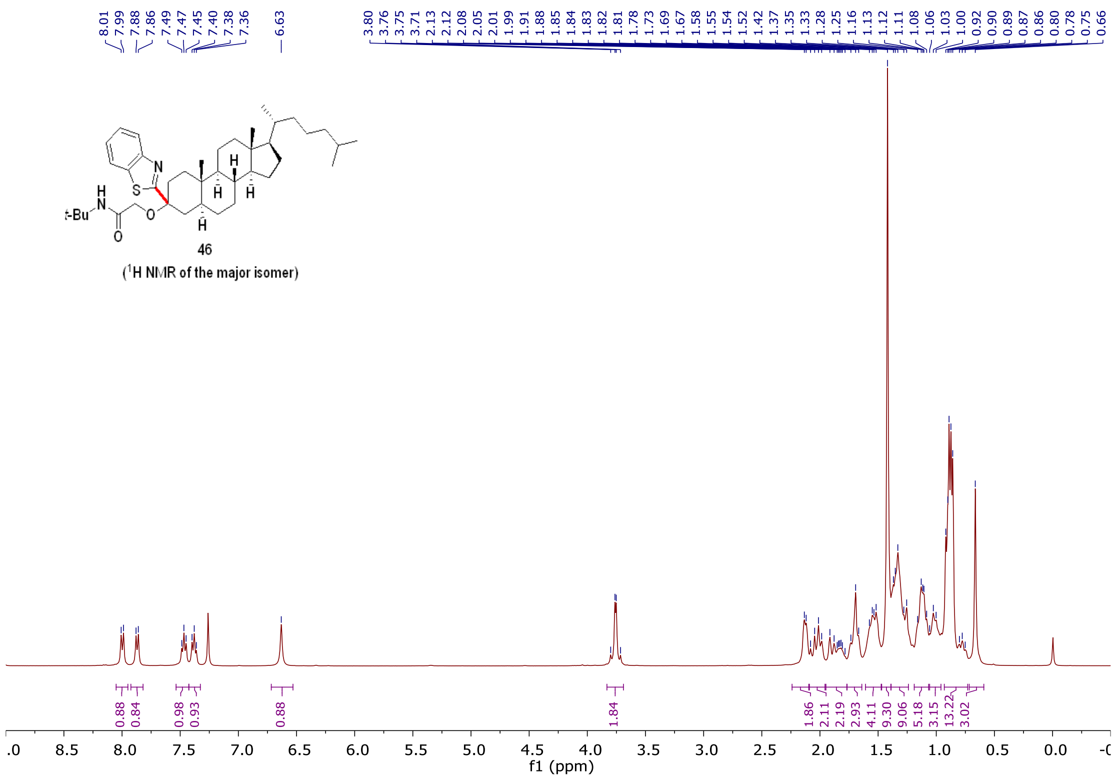


**Supplementary Figure 148.** $^{1}$H NMR spectra for **46**

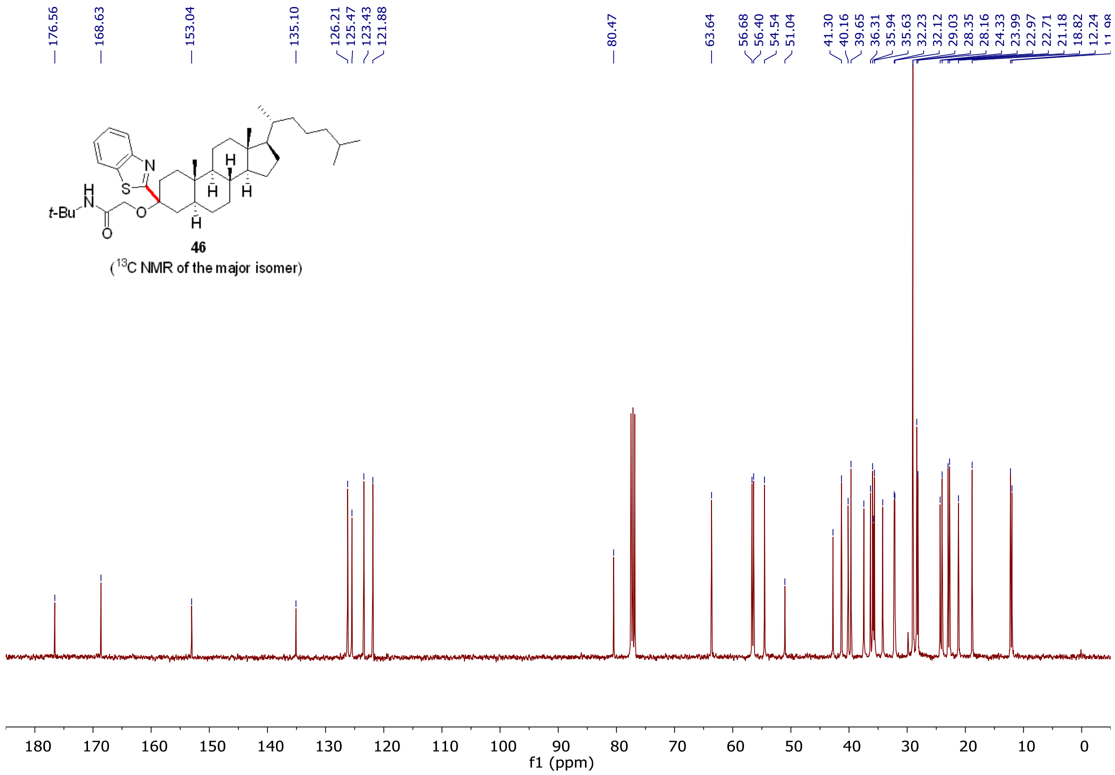


**Supplementary Figure 149.** $^{13}$C NMR spectra for **46**

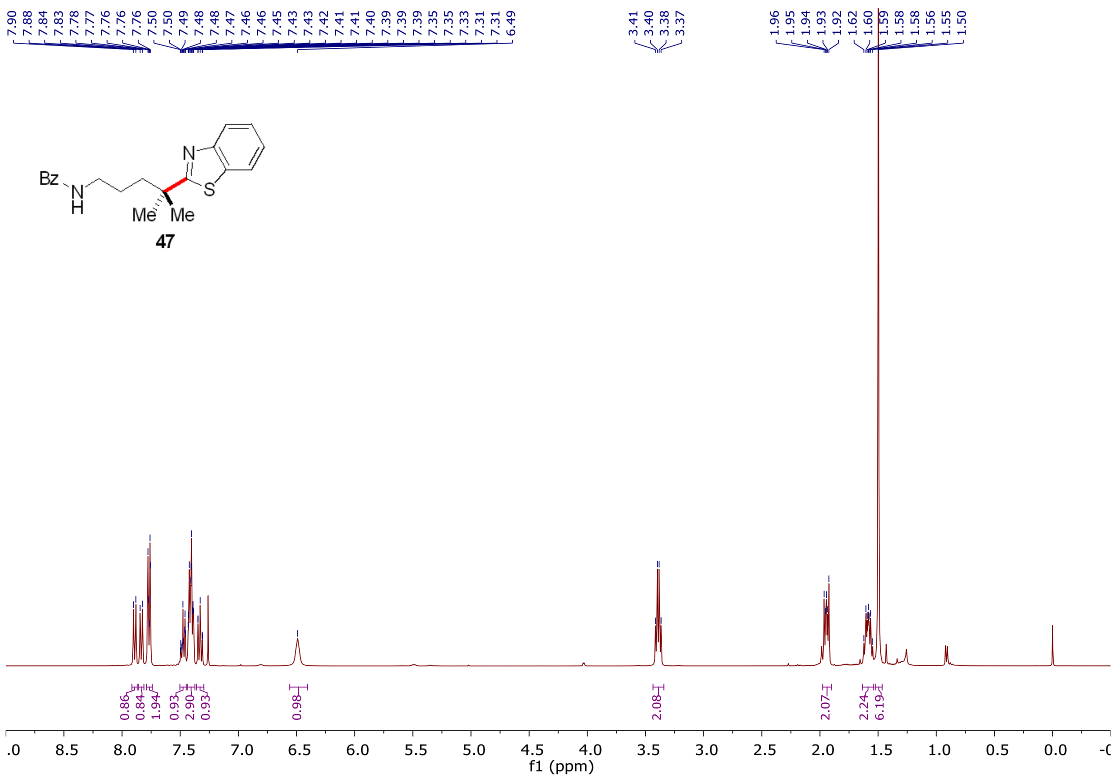

**Supplementary Figure 150.** $^{1}$H NMR spectra for **47**

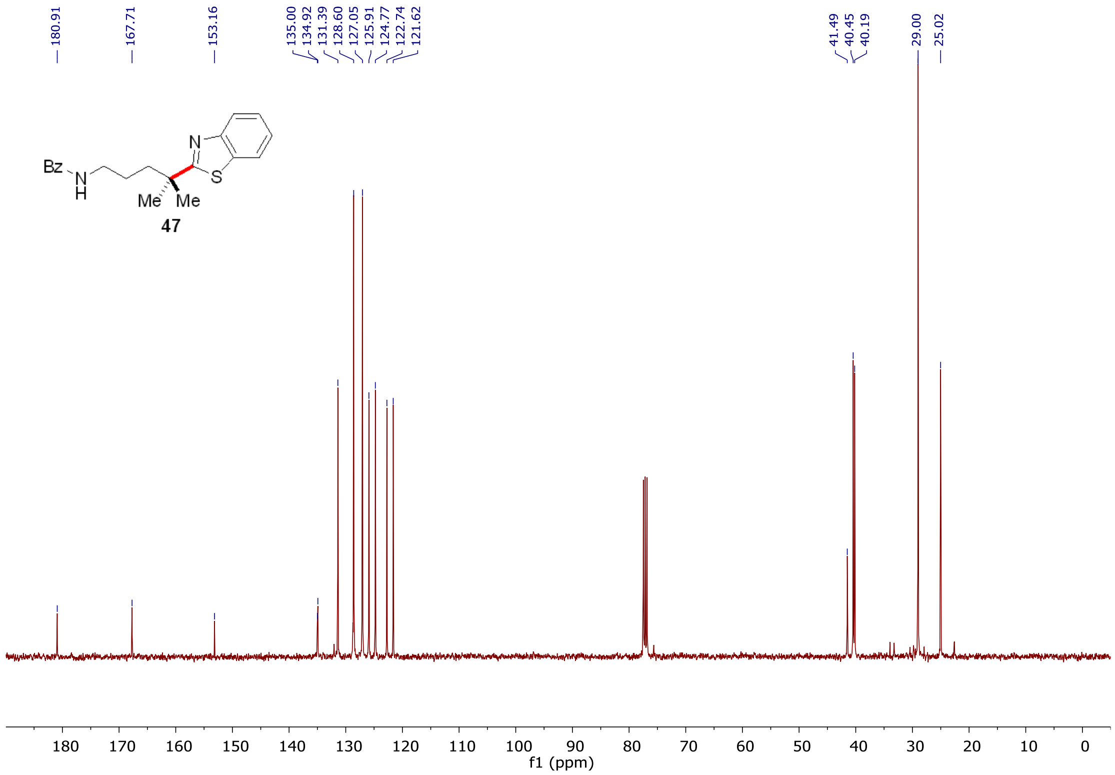

**Supplementary Figure 151.** $^{13}$C NMR spectra for **47**

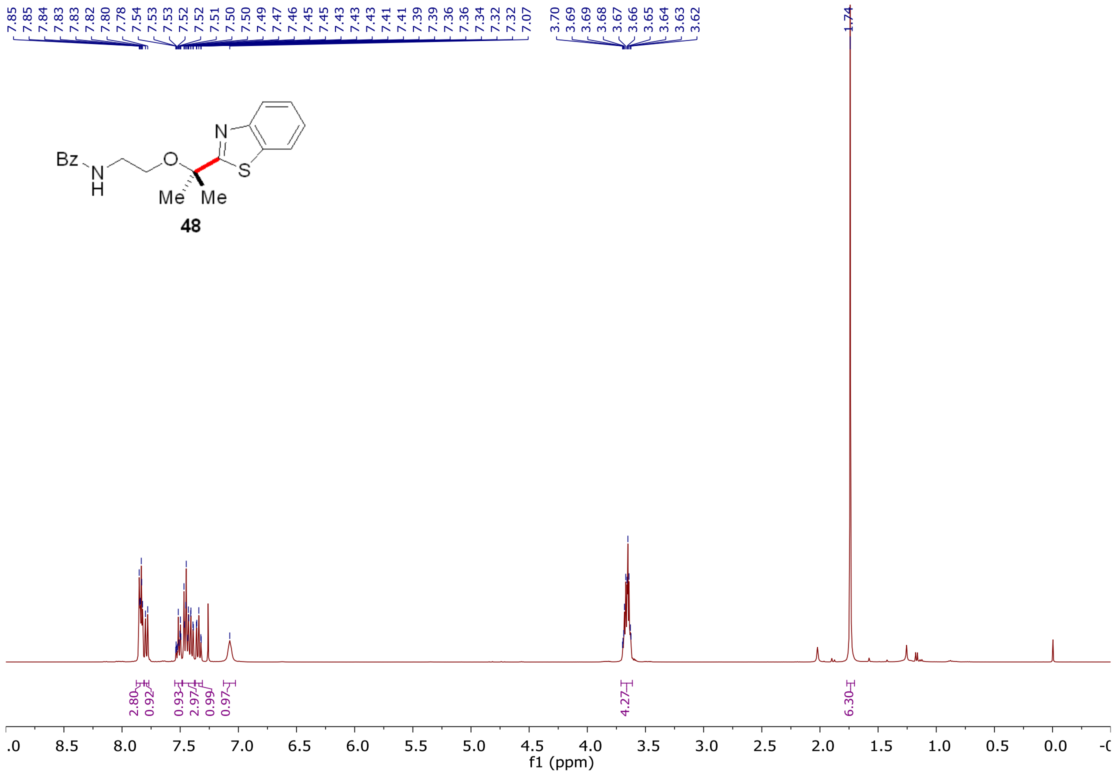

**Supplementary Figure 152.** $^{1}$H NMR spectra for **48**

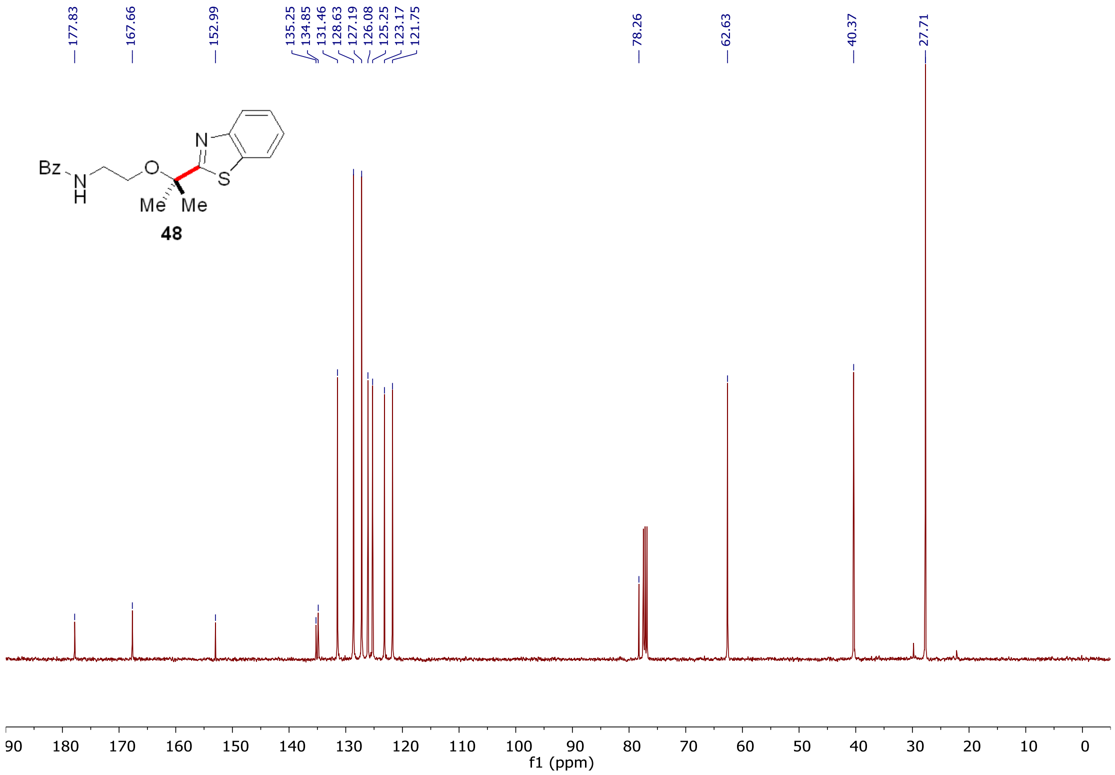

**Supplementary Figure 153.** $^{13}$C NMR spectra for **48**

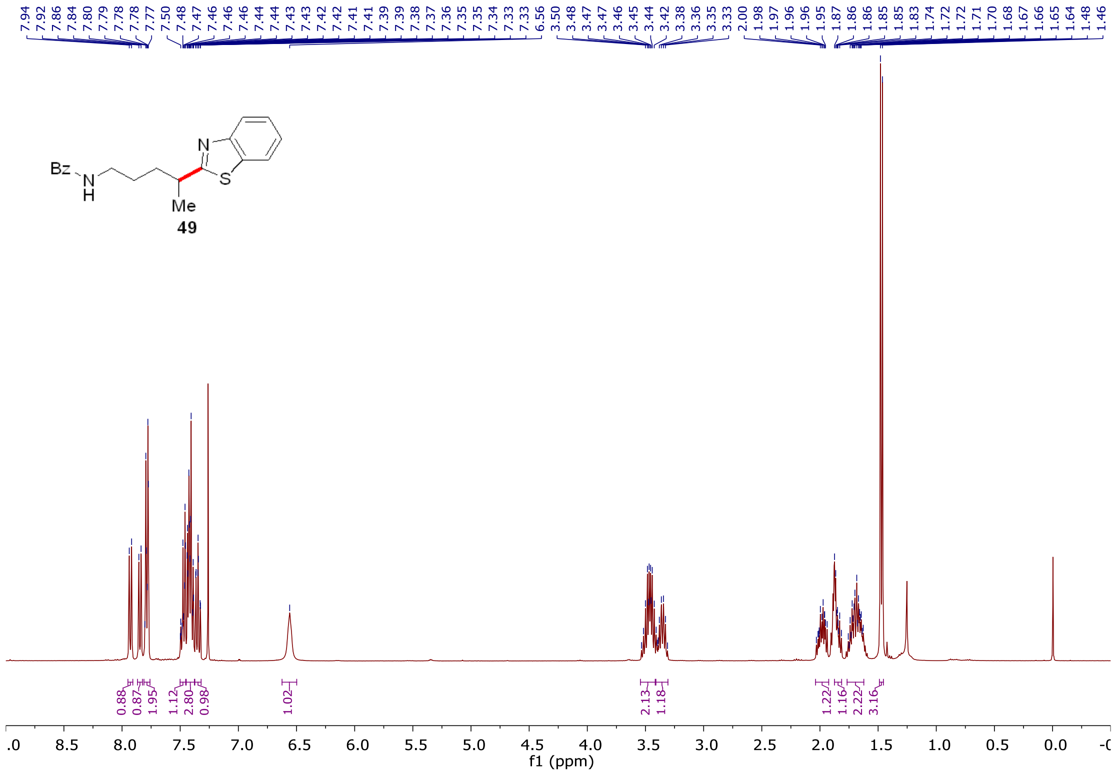


**Supplementary Figure 154.** $^{1}$H NMR spectra for **49**

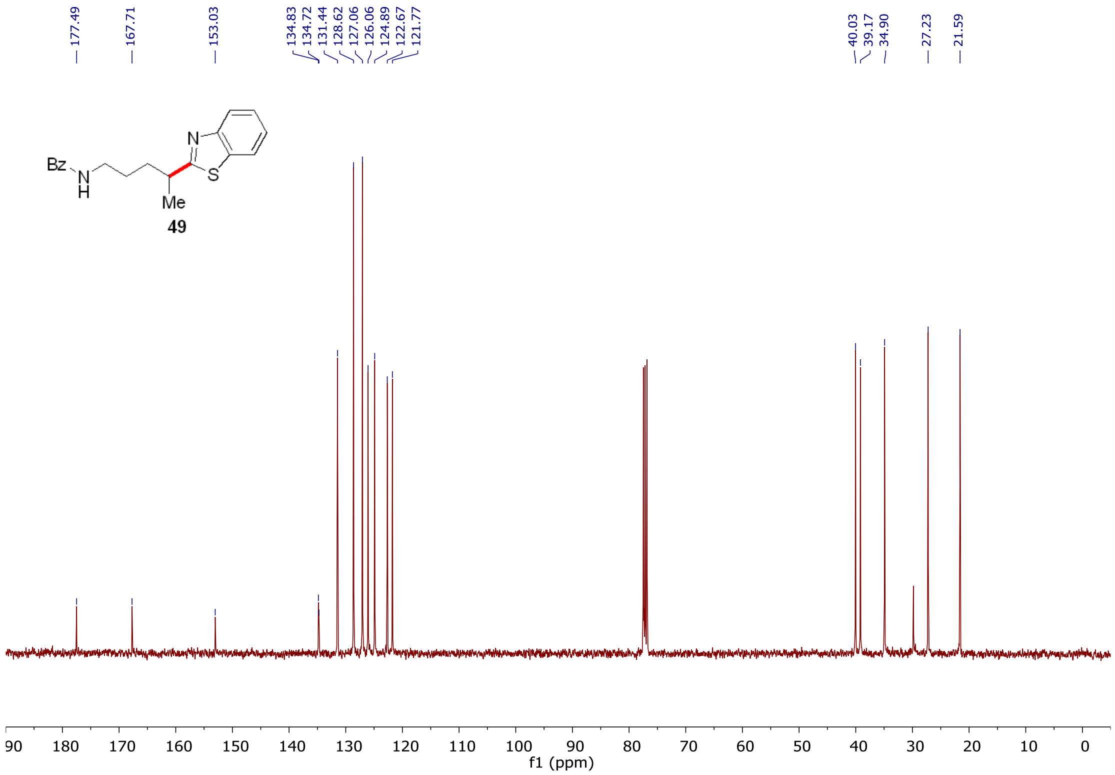


**Supplementary Figure 155.** $^{13}$C NMR spectra for **49**

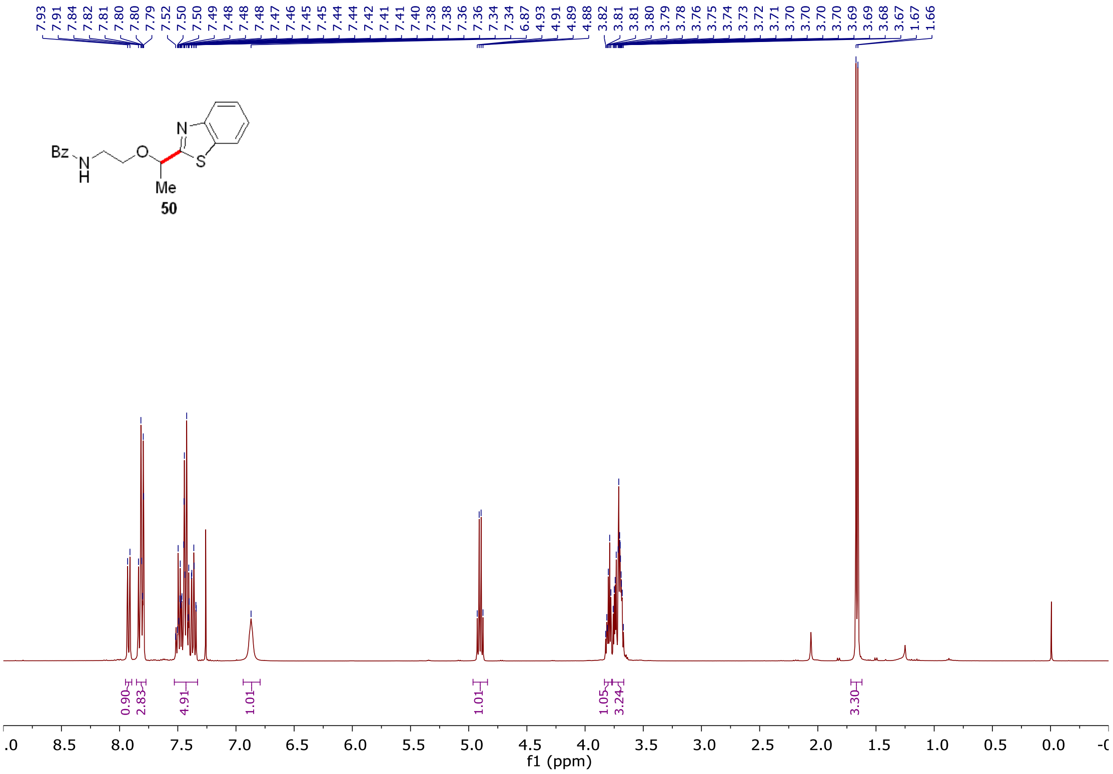


**Supplementary Figure 156.** $^{1}$H NMR spectra for **50**

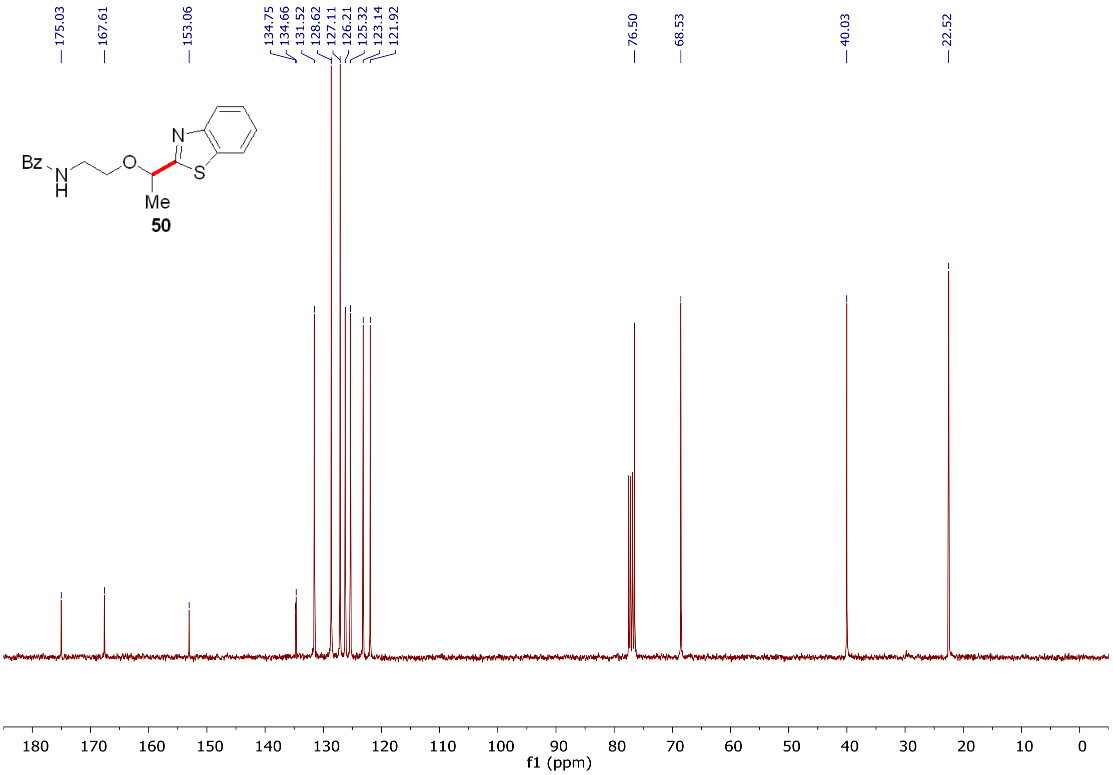


**Supplementary Figure 157.** $^{13}$C NMR spectra for **50**

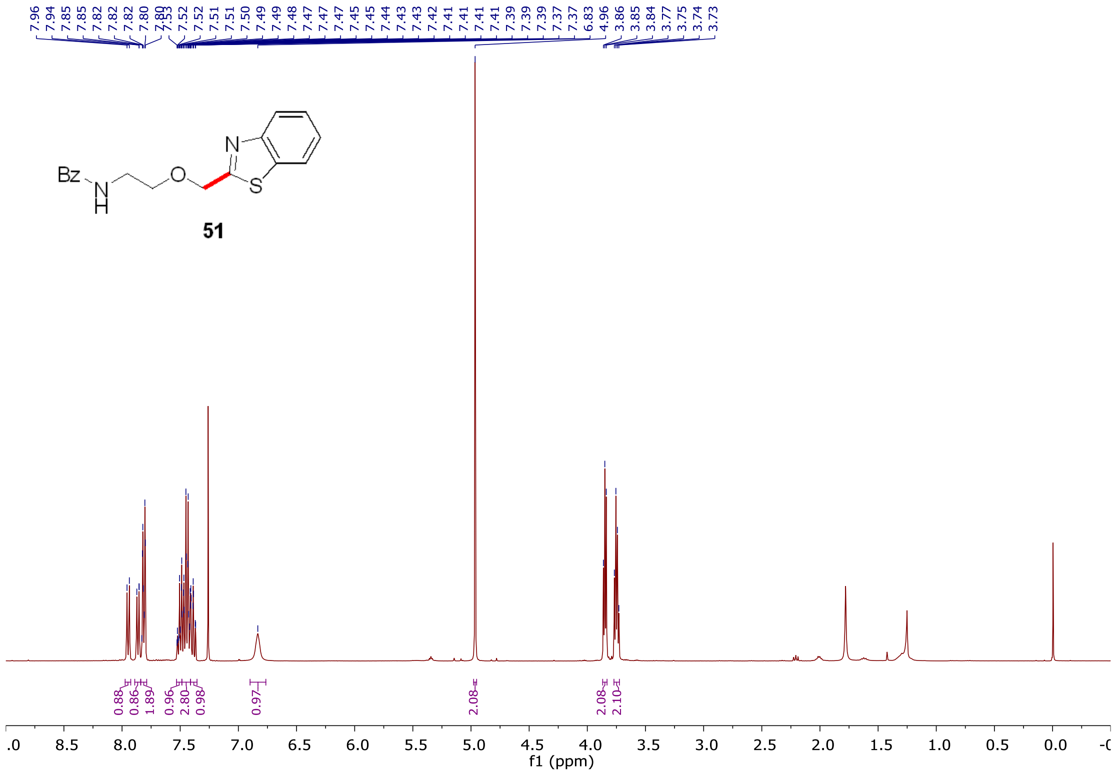

**Supplementary Figure 158.** $^{1}$H NMR spectra for **51**

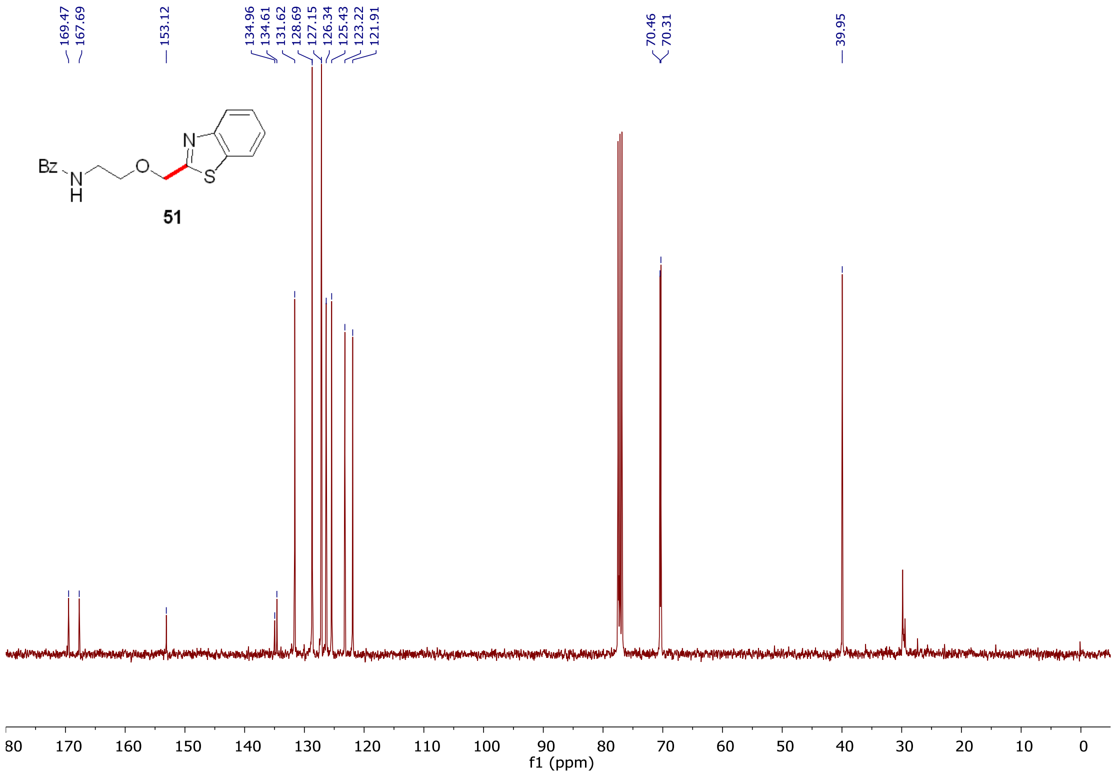

**Supplementary Figure 159.** $^{13}$C NMR spectra for **51**

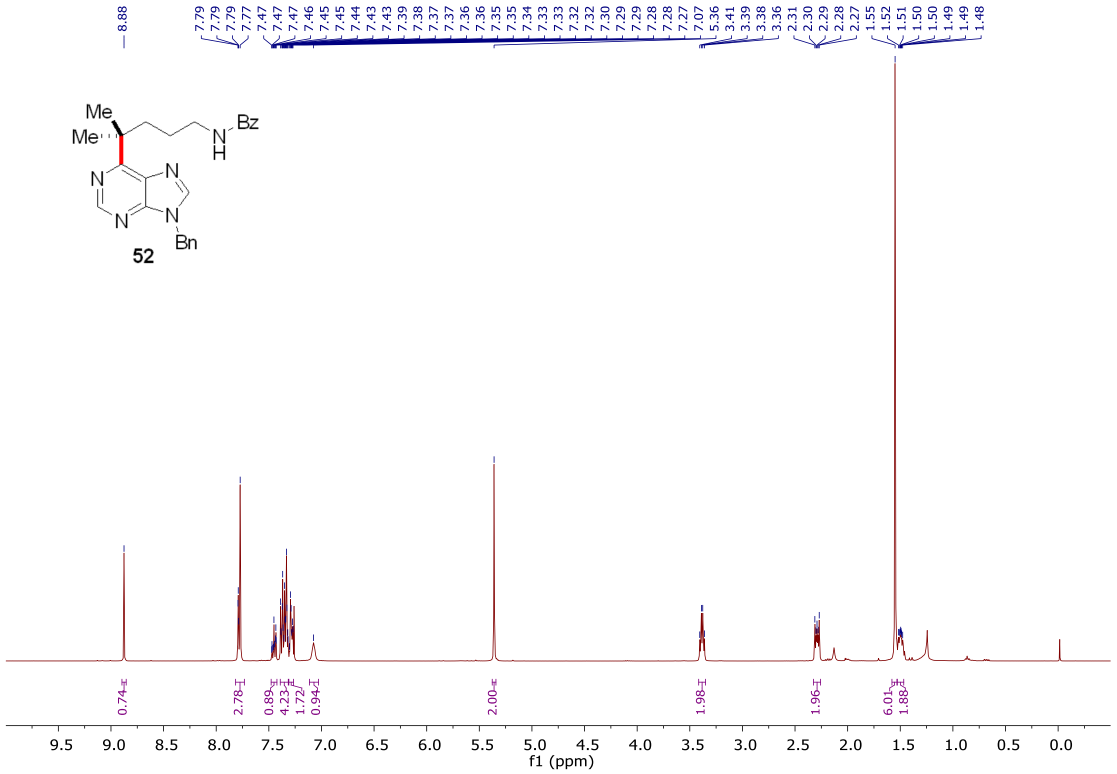


**Supplementary Figure 160.** $^{1}$H NMR spectra for **52**

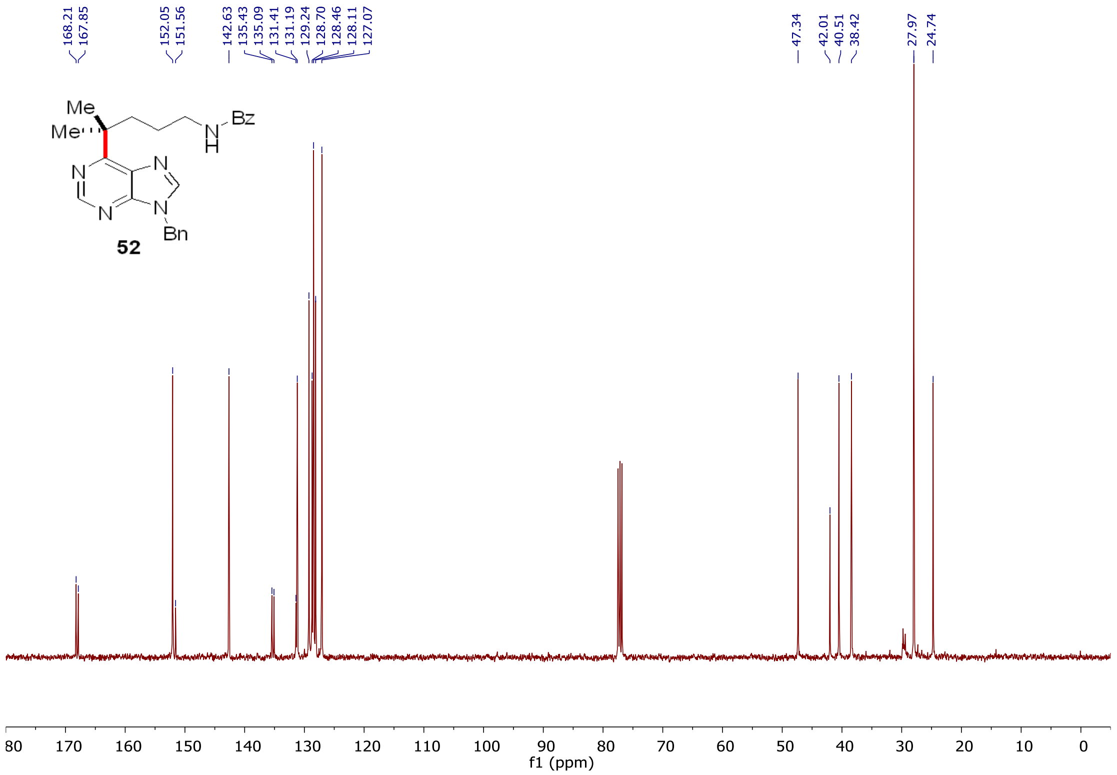


**Supplementary Figure 161.** $^{13}$C NMR spectra for **52**

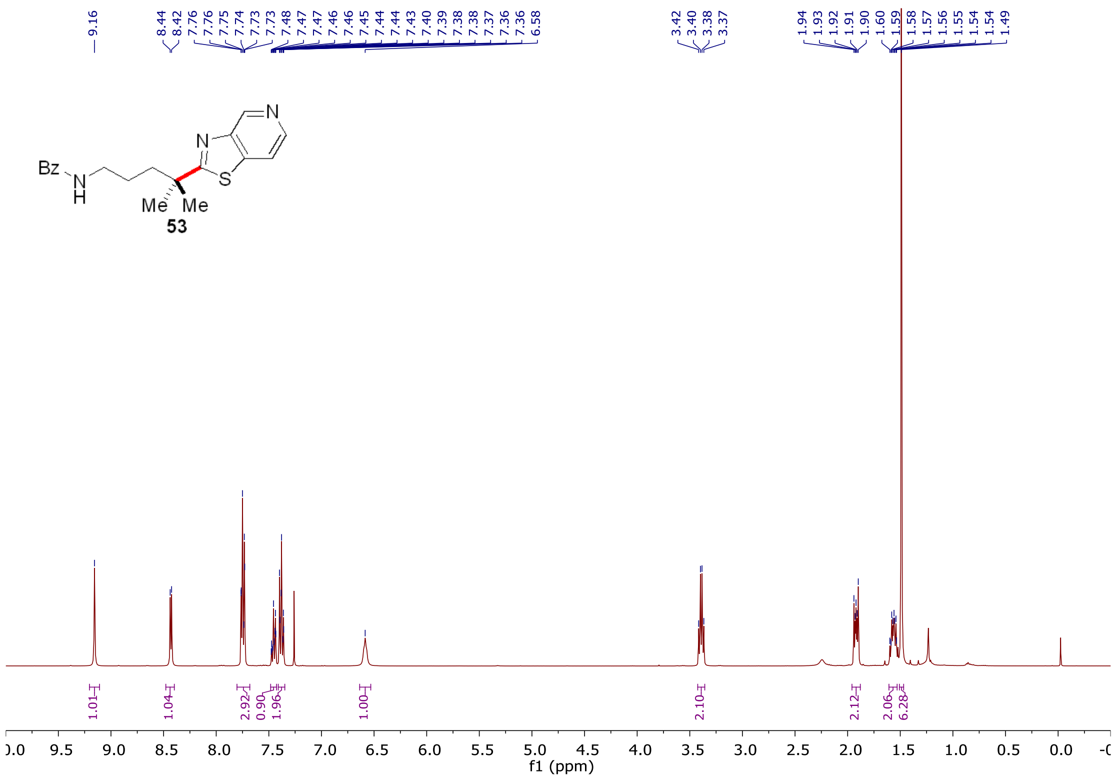


**Supplementary Figure 162.** $^{1}$H NMR spectra for **53**

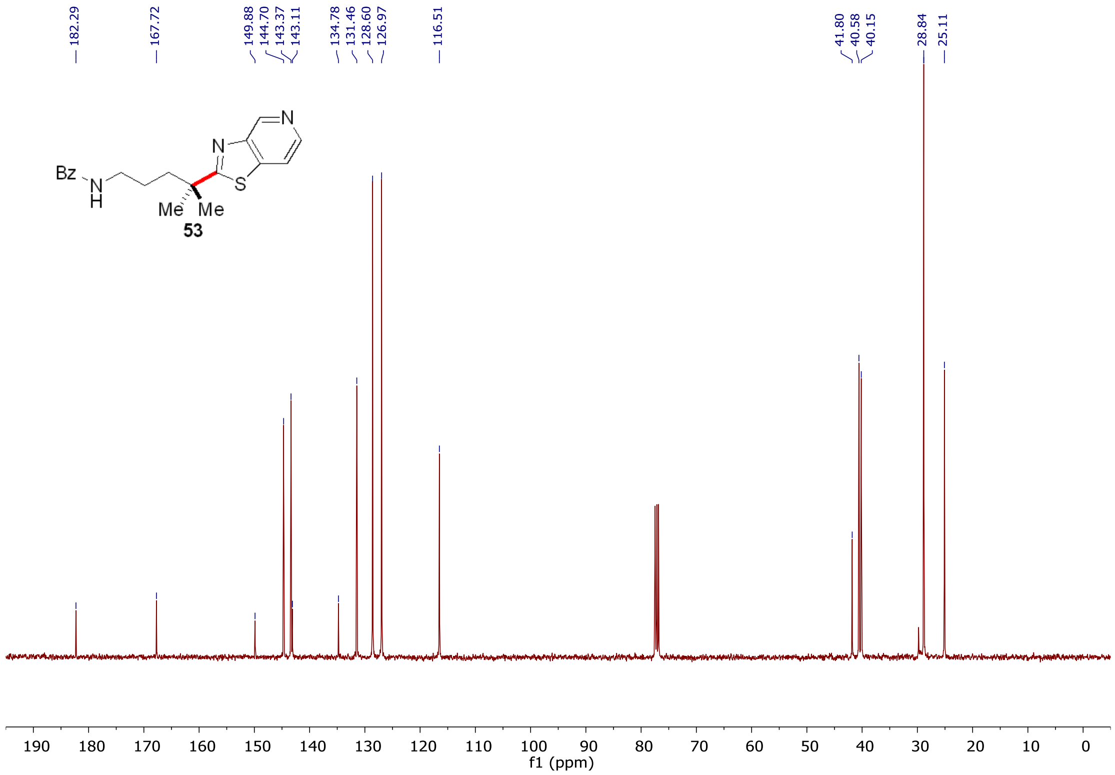


**Supplementary Figure 163.** $^{13}$C NMR spectra for **53**

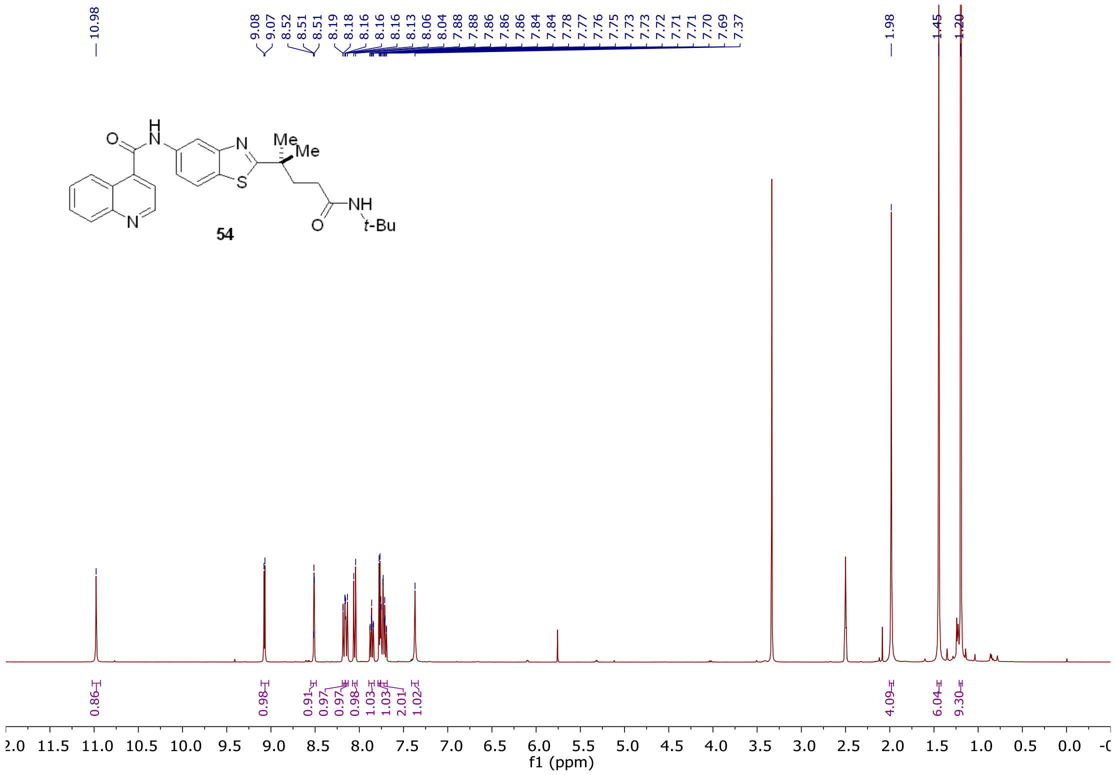


**Supplementary Figure 164.** $^{1}$H NMR spectra for **54**

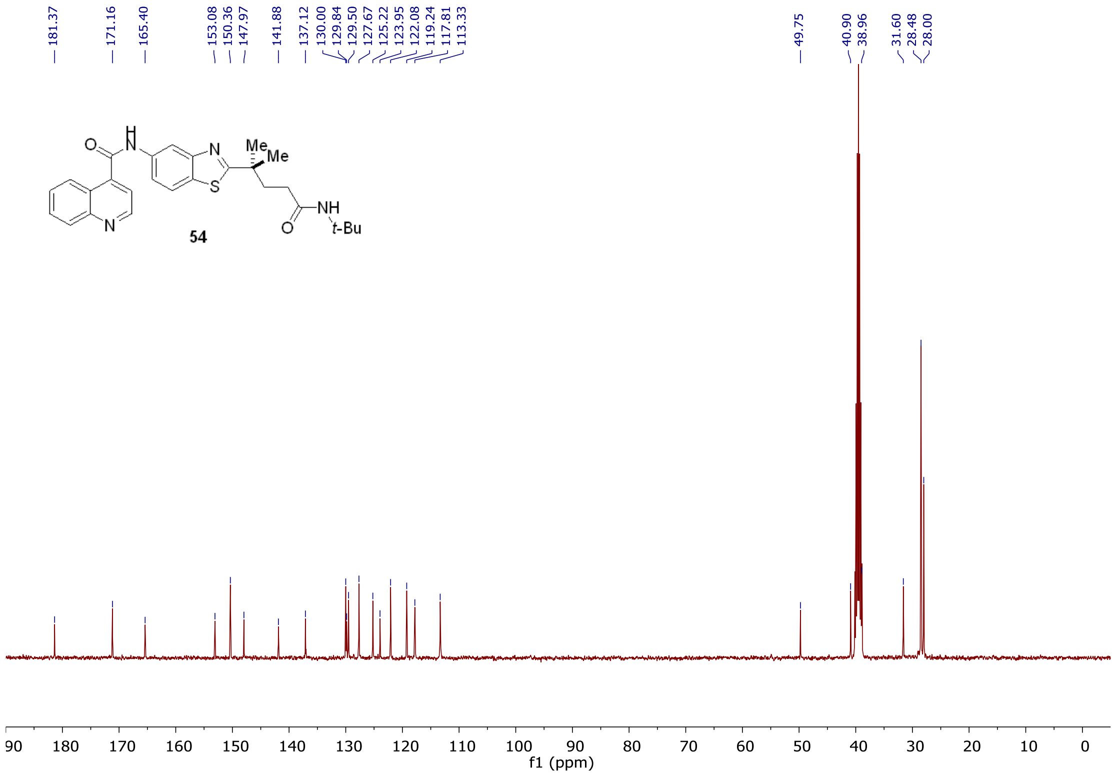


**Supplementary Figure 165.** $^{13}$C NMR spectra for **54**

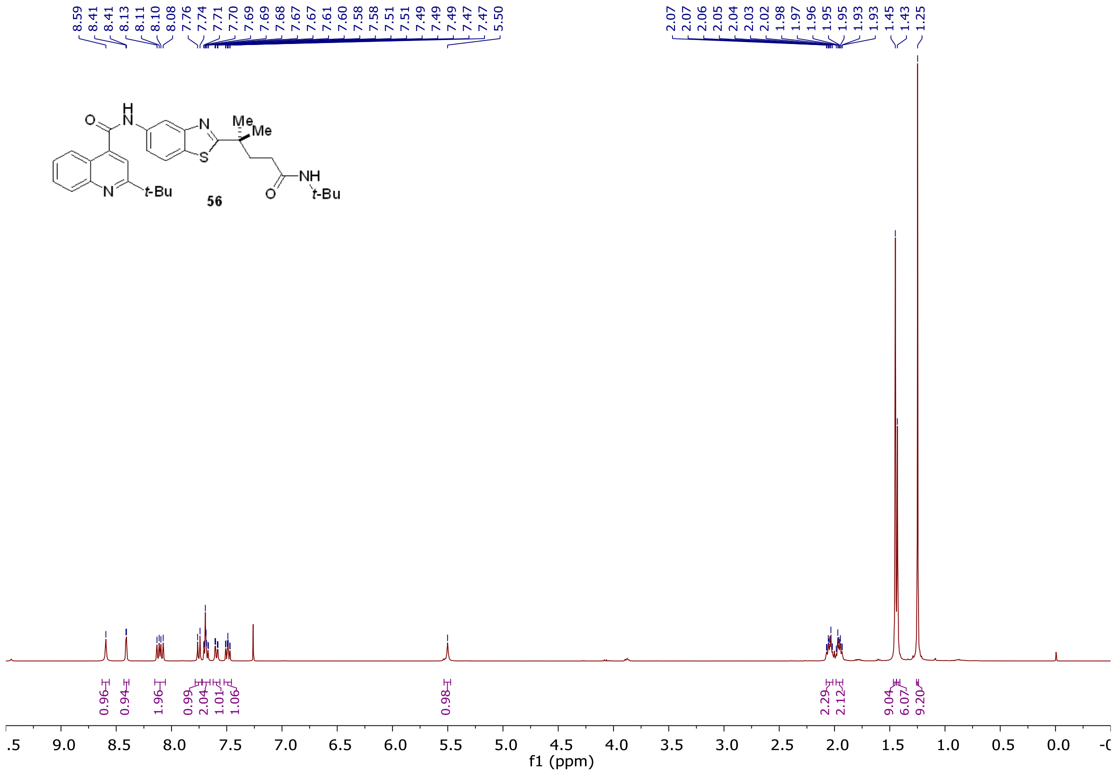


**Supplementary Figure 166.** $^{1}$H NMR spectra for **56**

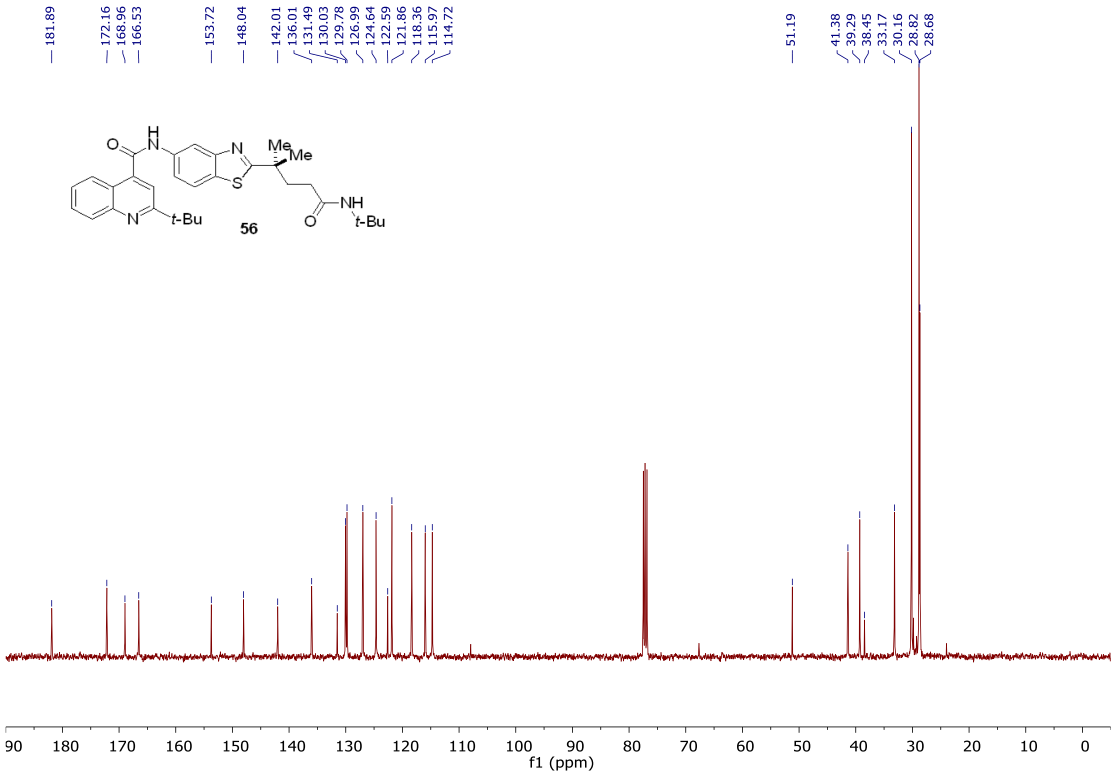


**Supplementary Figure 167.** $^{13}$C NMR spectra for **56**

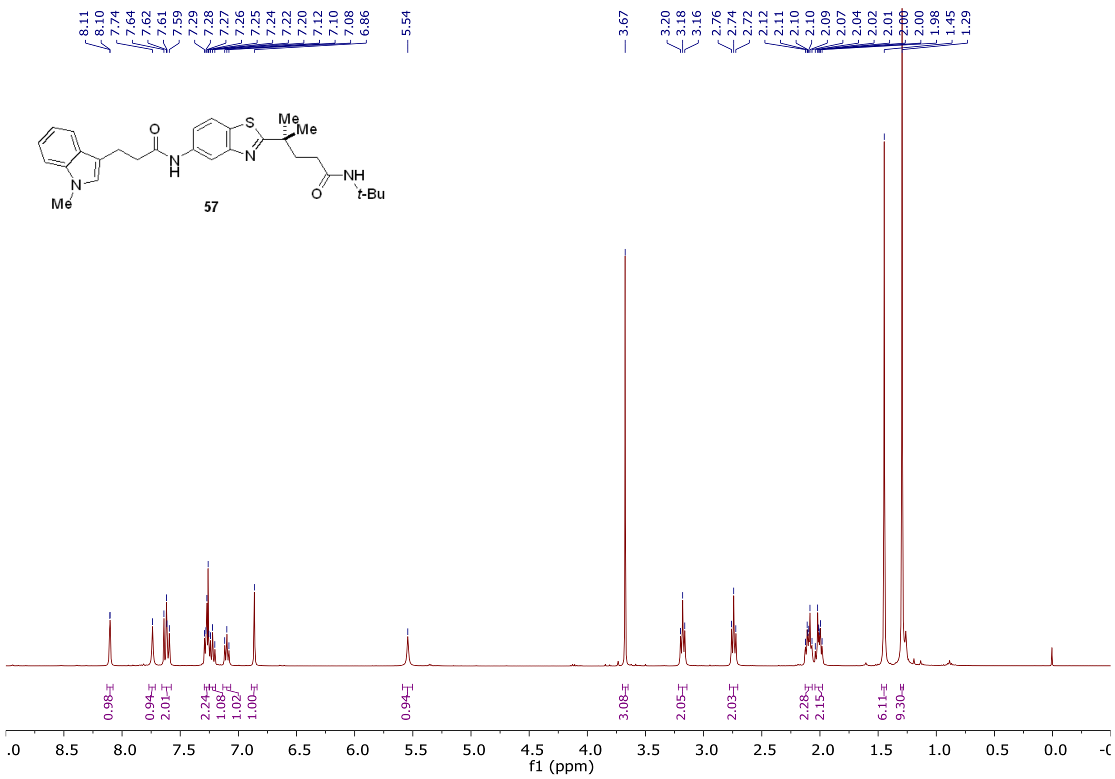

**Supplementary Figure 168.** $^{1}$H NMR spectra for **57**

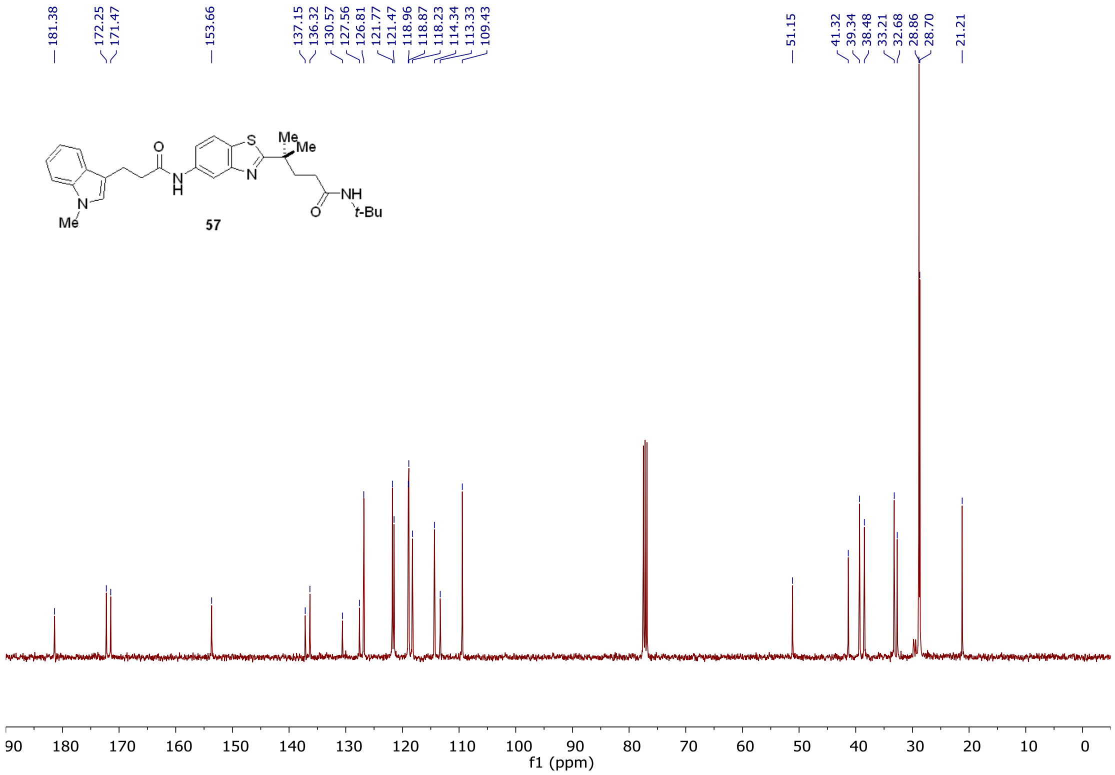

**Supplementary Figure 169.** $^{13}$C NMR spectra for **57**

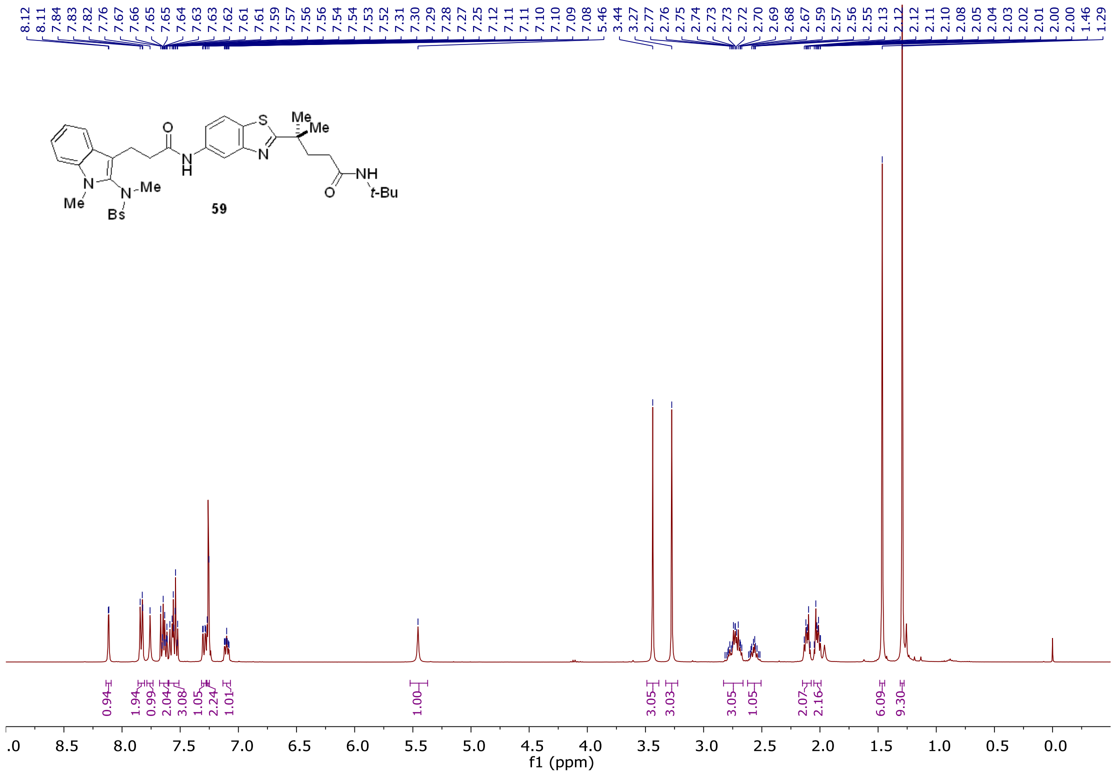

**Supplementary Figure 170.** $^{1}$H NMR spectra for **59**

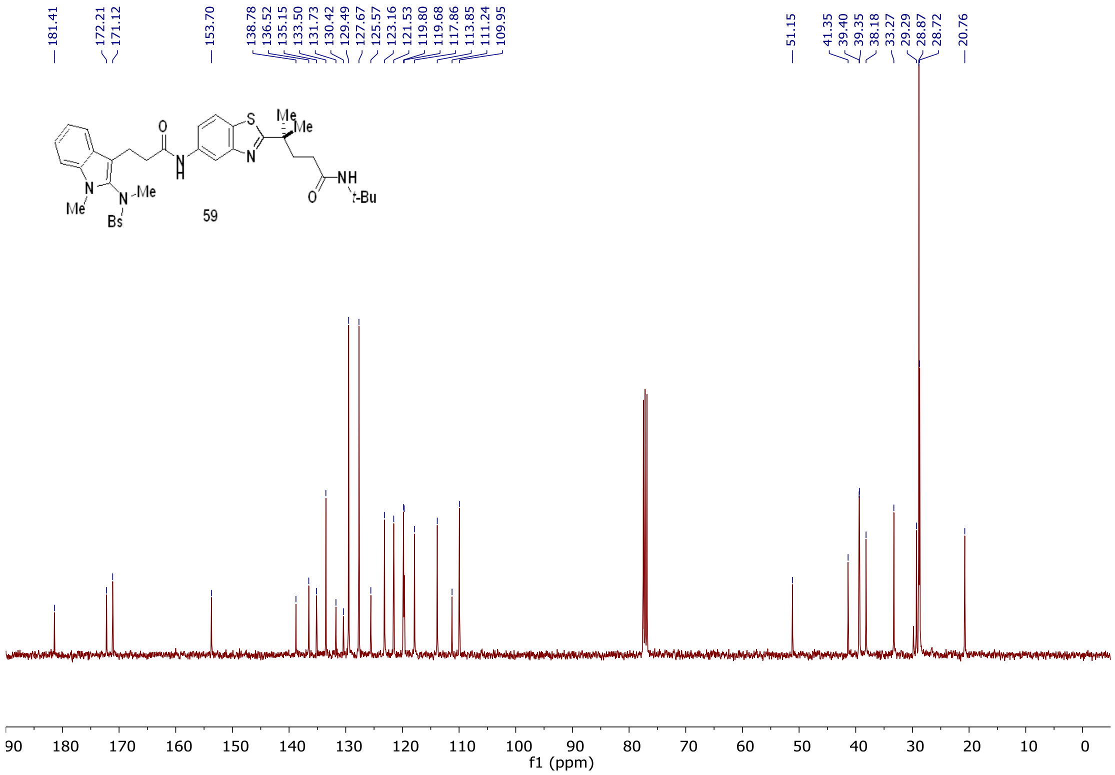

**Supplementary Figure 171.** $^{13}$C NMR spectra for **59**

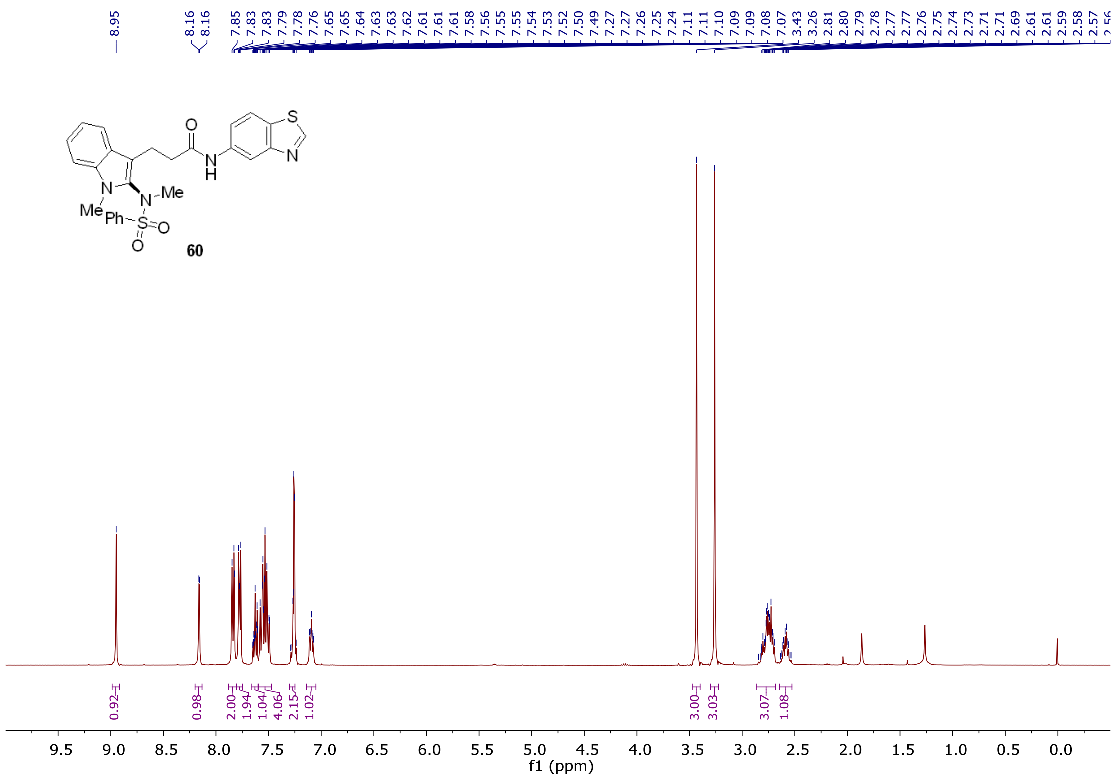

**Supplementary Figure 172.** $^{1}$H NMR spectra for **60**

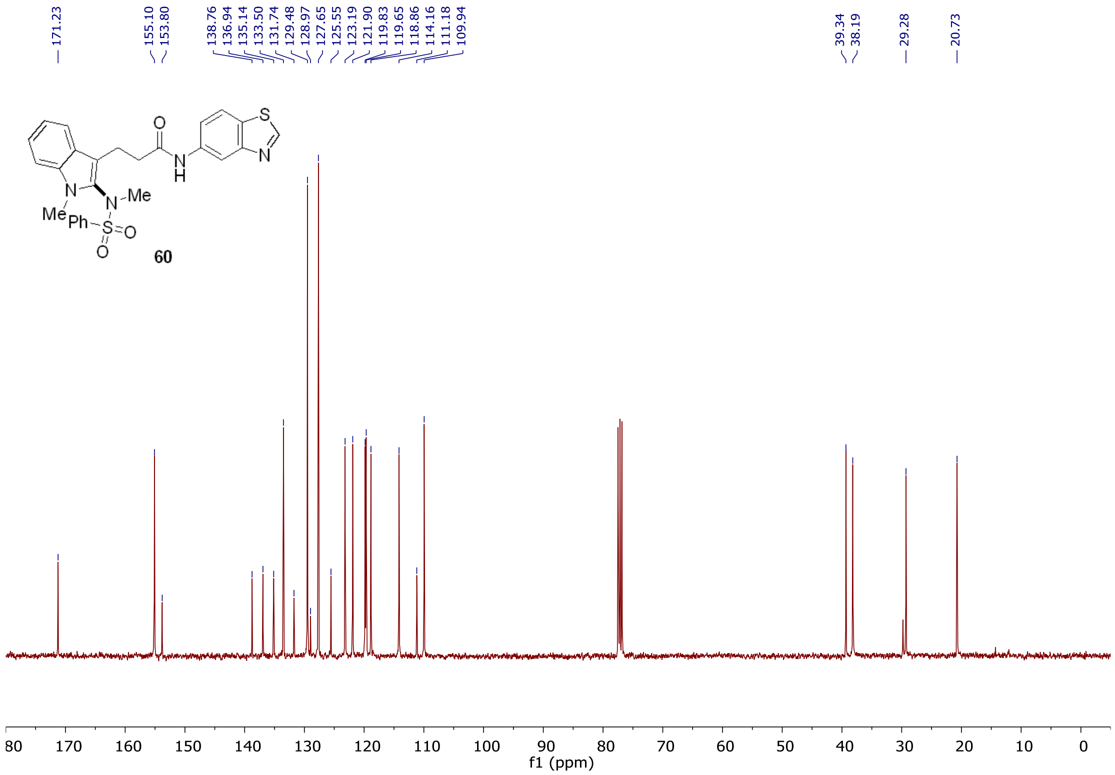

**Supplementary Figure 173.** $^{13}$C NMR spectra for **60**

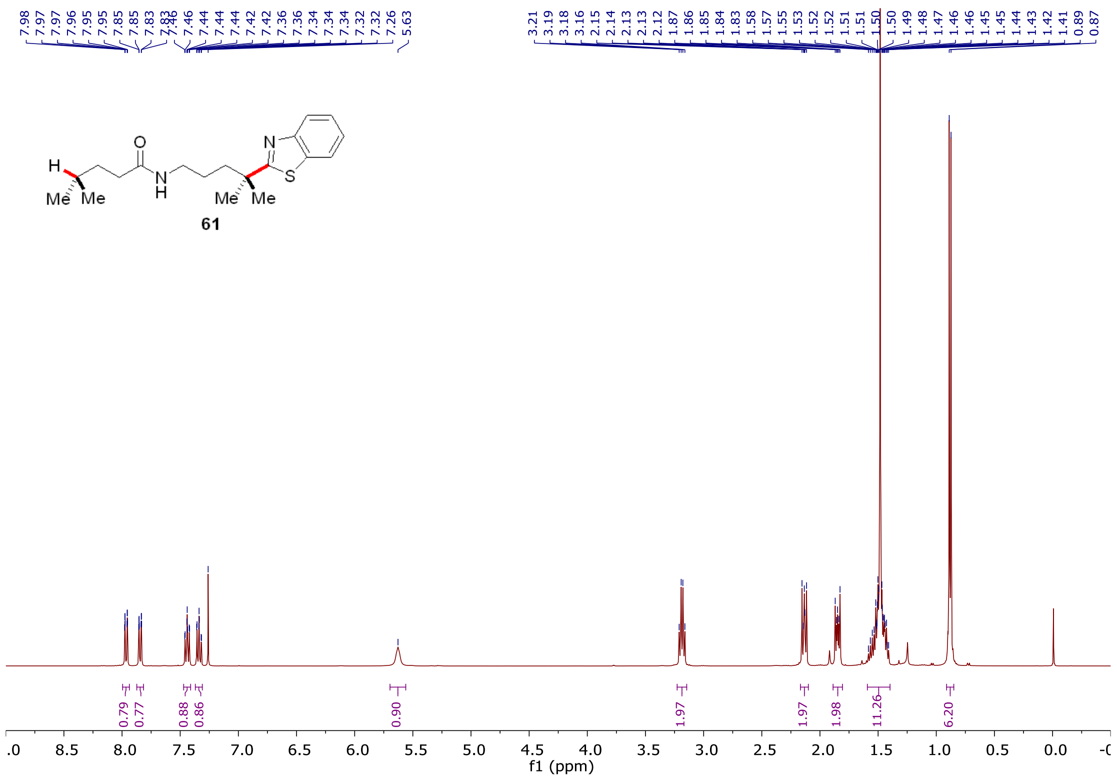

**Supplementary Figure 174.** $^{1}$H NMR spectra for **61**

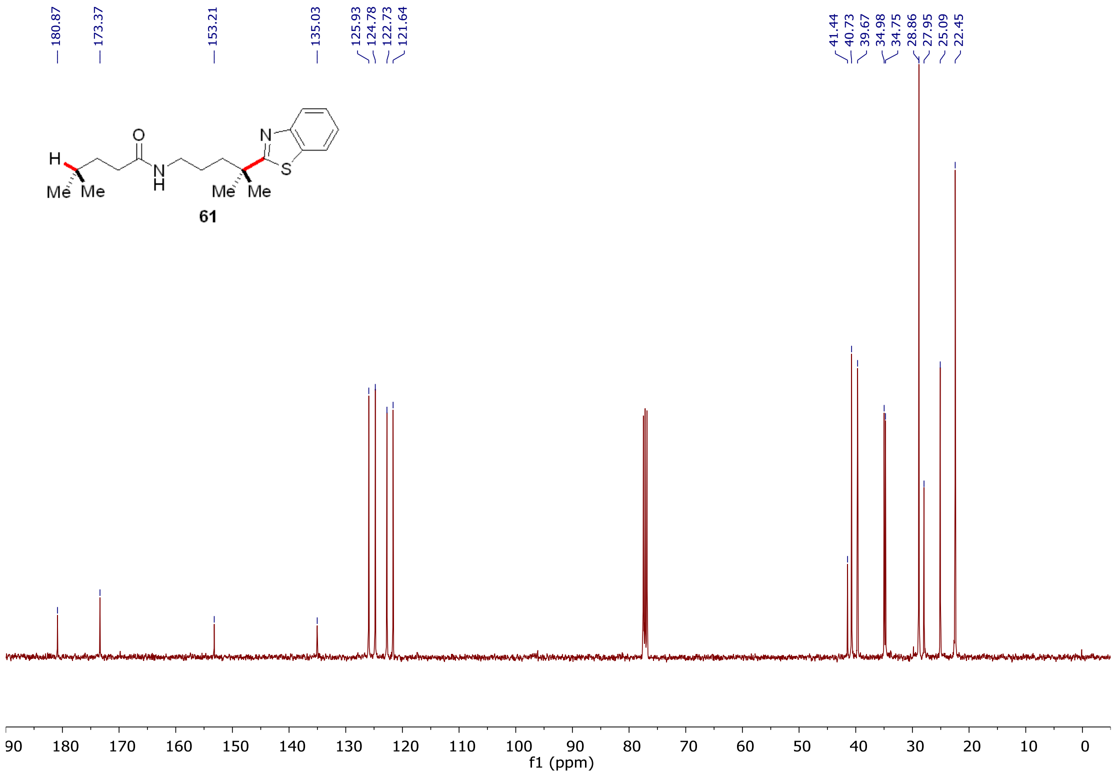

**Supplementary Figure 175.** $^{13}$C NMR spectra for **61**

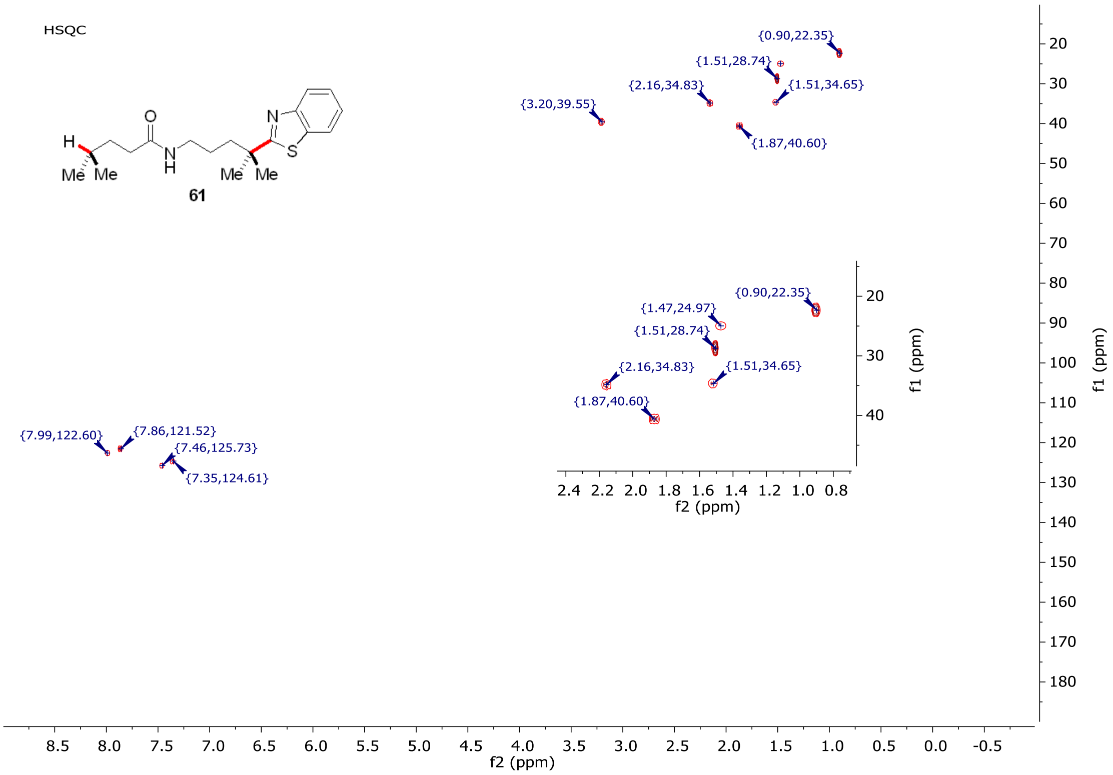


**Supplementary Figure 176.** HSQC NMR spectra for **61**

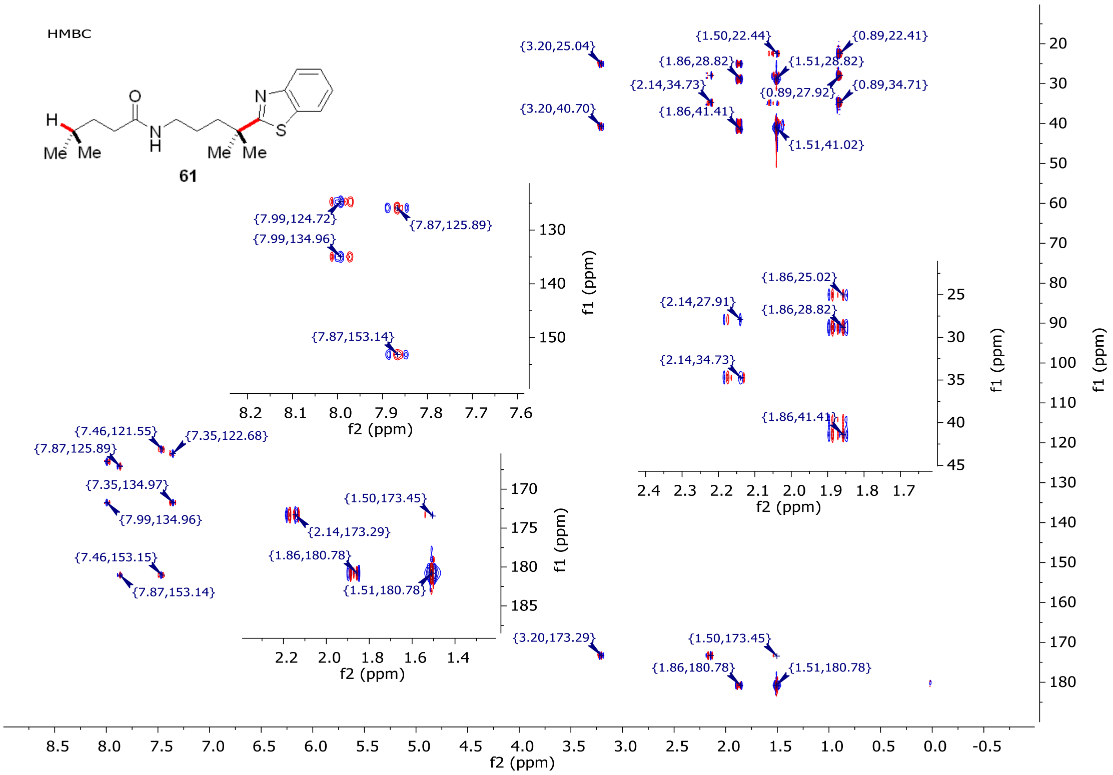


**Supplementary Figure 177.** HMBC NMR spectra for **61**

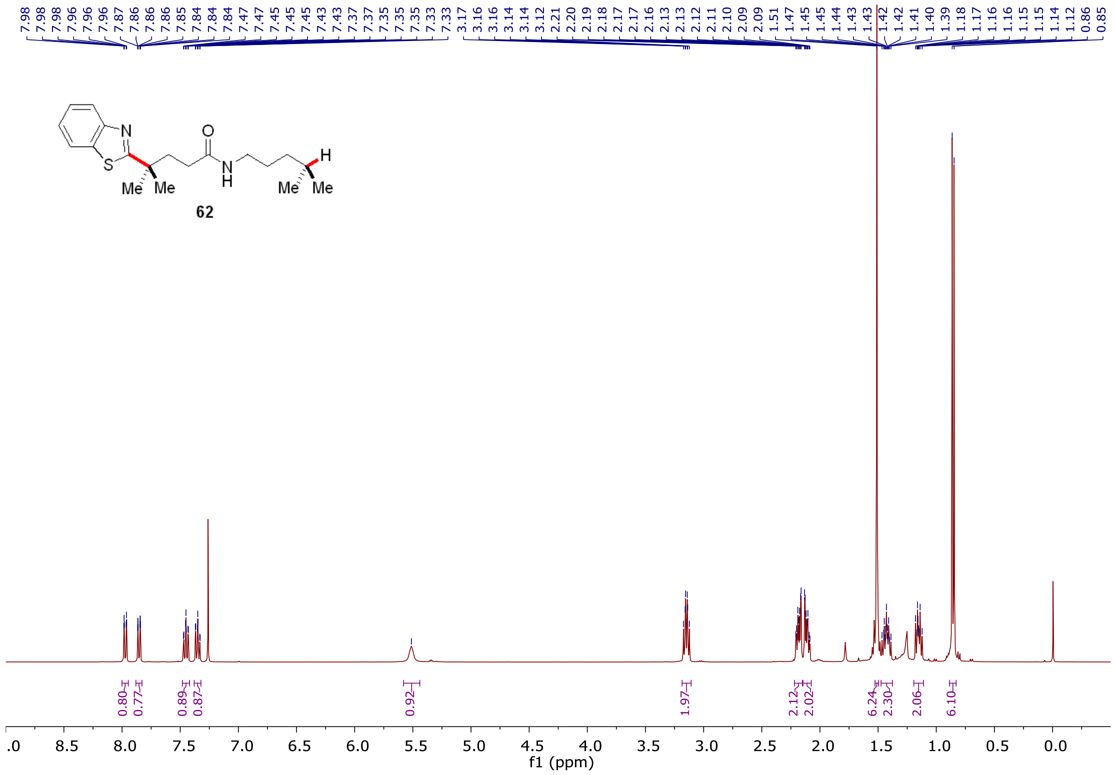

**Supplementary Figure 178.** $^{1}$H NMR spectra for **62**

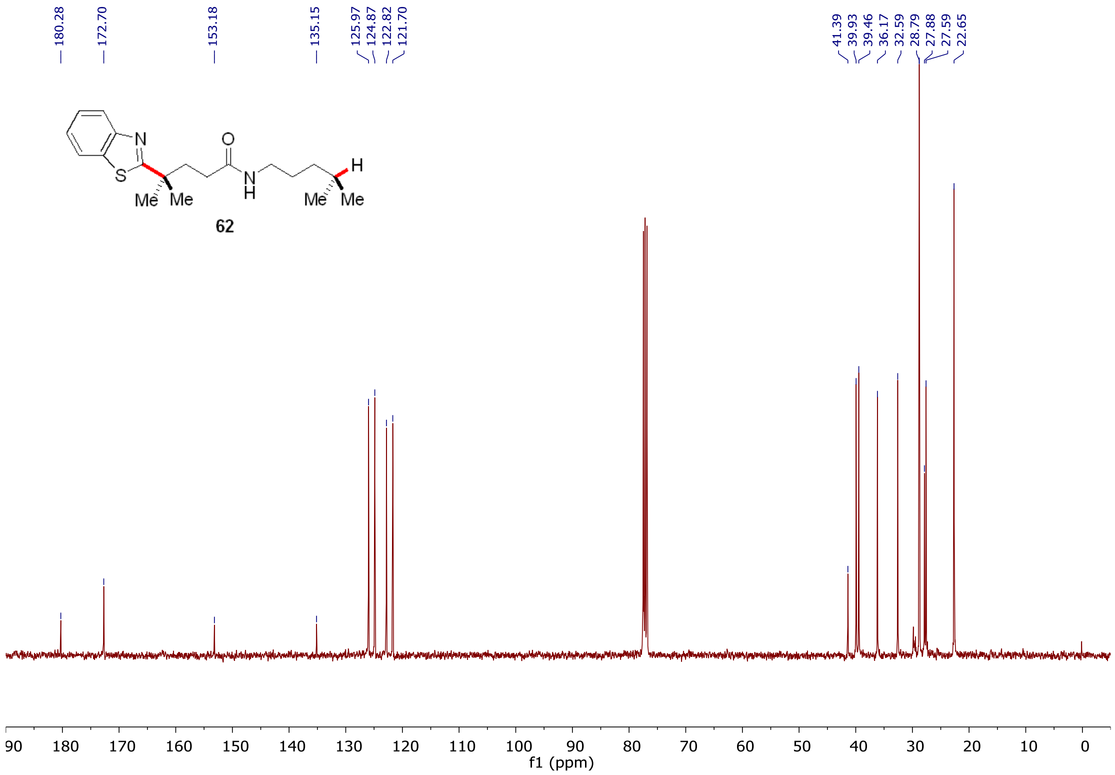

**Supplementary Figure 179.** $^{13}$C NMR spectra for **62**

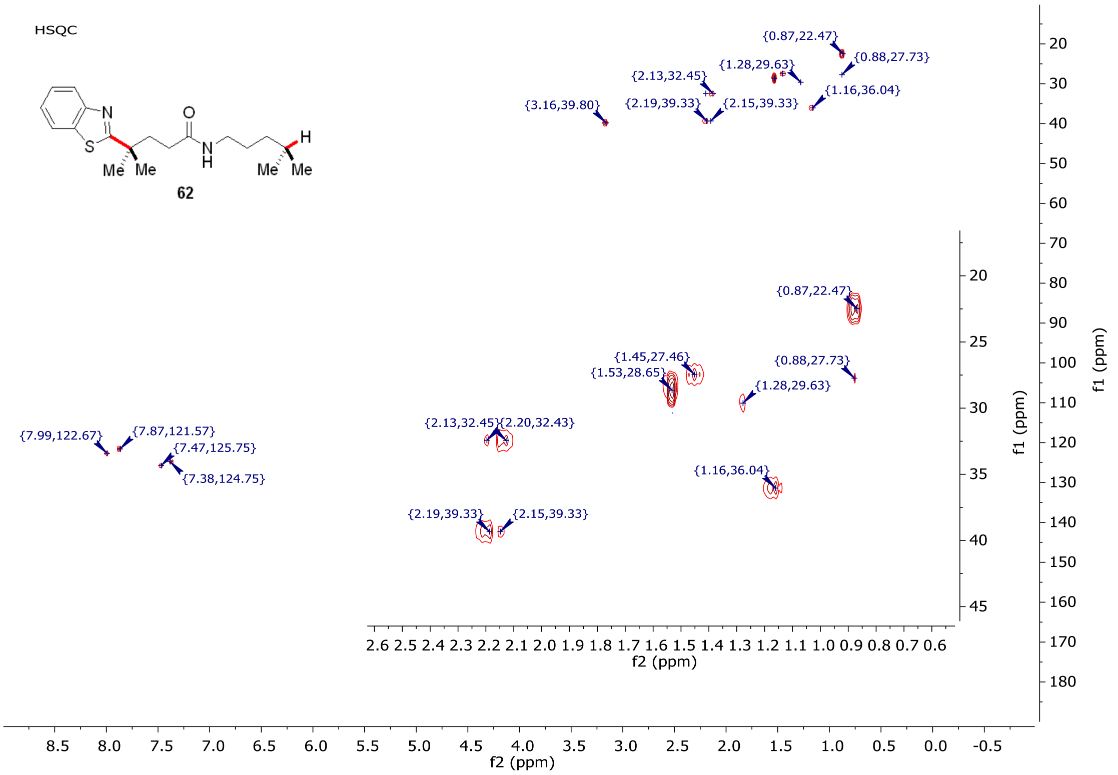


**Supplementary Figure 180.** HSQC NMR spectra for **62**

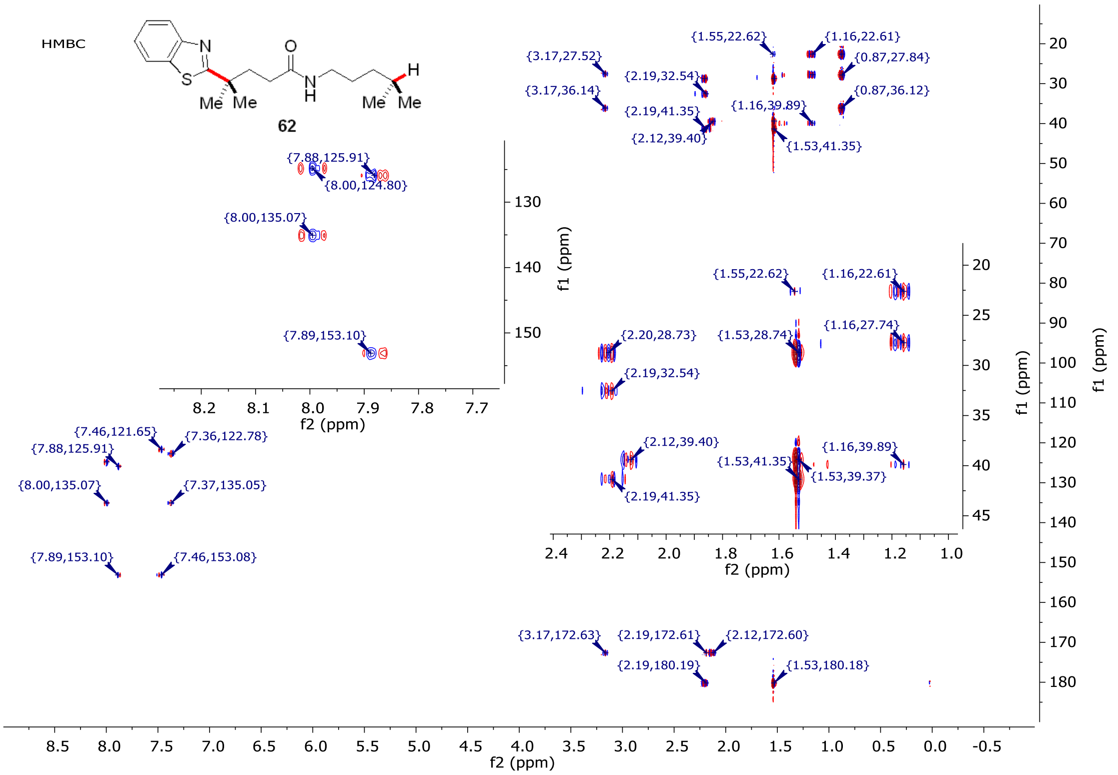


**Supplementary Figure 181.** HMBC NMR spectra for **62**

## Supplementary References


1 Chen, H., Guo, L. & Yu, S. Primary, Secondary, and Tertiary γ-C(sp3)–H Vinylation of Amides via Organic Photoredox-Catalyzed Hydrogen Atom Transfer. *Org. Lett.* **20**, 6255-6259 (2018).

2 Luo, Z., Jiang, Z., Jiang, W. & Lin, D. C–H Amination of Purine Derivatives via Radical Oxidative Coupling. *J. Org. Chem.* **83**, 3710-3718 (2018).

3 Zou, Y., Yue, G., Xu, J. & Zhou, J. General Suzuki Coupling of Heteroaryl Bromides by Using Tri-tert-butylphosphine as a Supporting Ligand. *Eur. J. Org. Chem.* **2014**, 5901-5905 (2014).

4 He, Z., Shrives, H. J., Fernández-Salas, J. A., Abengózar, A., Neufeld, J., Yang, K., Pulis, A. P. & Procter, D. J. Synthesis of C2 Substituted Benzothiophenes via an Interrupted Pummerer/[3,3]-Sigmatropic/1,2-Migration Cascade of Benzothiophene S-Oxides. *Angew. Chem., Int. Ed.* **57**, 5759-5764 (2018).

5 Baudy, R. B., Greenblatt, L. P., Jirkovsky, I. L., Conklin, M., Russo, R. J., Bramlett, D. R., Emrey, T. A., Simmonds, J. T. & Kowal, D. M. Potent quinoxaline-spaced phosphono .alpha.-amino acids of the AP-6 type as competitive NMDA antagonists: synthesis and biological evaluation. *J. Med. Chem.* **36**, 331-342 (1993).

6 Zheng, C., Wang, Y., Xu, Y., Chen, Z., Chen, G. & Liang, S. H. Ru-Photoredox-Catalyzed Decarboxylative Oxygenation of Aliphatic Carboxylic Acids through N-(acyloxy)phthalimide. *Org. Lett.* **20**, 4824-4827 (2018).

7 Qin, Q. & Yu, S. Visible-Light-Promoted Redox Neutral C–H Amidation of Heteroarenes with Hydroxylamine Derivatives. *Org. Lett.* **16**, 3504-3507 (2014).

8 Banerjee, A. & Yamamoto, H. Direct N–O bond formation via oxidation of amines with benzoyl peroxide. *Chem. Sci.* **10**, 2124-2129 (2019).

9 Yu, W.-Y., Sit, W. N., Zhou, Z. & Chan, A. S. C. Palladium-Catalyzed Decarboxylative Arylation of C−H Bonds by Aryl Acylperoxides. *Org. Lett.* **11**, 3174-3177 (2009).

10 Frisch, M. J., Trucks, G. W., Schlegel, H. B., Scuseria, G. E., Robb, M. A.,

Cheeseman, J. R., Scalmani, G., Barone, V., Mennucci,B., Petersson, G. A., et al. Gaussian 09, revision D.01; Gaussian, Inc.: Wallingford, CT, 2013.

11 Marenich, A. V., Cramer, C. J. & Truhlar, D. G. Universal Solvation Model Based on Solute Electron Density and on a Continuum Model of the Solvent Defined by the Bulk Dielectric Constant and Atomic Surface Tensions. *J. Phys. Chem. B* **113**, 6378-6396 (2009).

12 Zhao, Y. & Truhlar, D. G. The M06 suite of density functionals for main group thermochemistry, thermochemical kinetics, noncovalent interactions, excited states, and transition elements: two new functionals and systematic testing of four M06-class functionals and 12 other functionals. *Theor. Chem. Acc.* **120**, 215-241 (2008).

13 Weigend, F. & Ahlrichs, R. Balanced basis sets of split valence, triple zeta valence and quadruple zeta valence quality for H to Rn: Design and assessment of accuracy. *PCCP* **7**, 3297-3305 (2005).

14 Fukui, K. Formulation of the reaction coordinate. *J. Phys. Chem.* **74**, 4161-4163 (1970).

15 Fukui, K. The path of chemical reactions - the IRC approach. *Acc. Chem. Res.* **14**, 363-368 (1981).

16 Grimme, S., Ehrlich, S. & Goerigk, L. Effect of the damping function in dispersion corrected density functional theory. *J. Comput. Chem.* **32**, 1456-1465 (2011).

17 Grimme, S., Antony, J., Ehrlich, S. & Krieg, H. A consistent and accurate ab initio parametrization of density functional dispersion correction (DFT-D) for the 94 elements H-Pu. *J. Chem. Phys.* **132**, 154104 (2010).

18 Li, Z., Zhang, S.-L., Fu, Y., Guo, Q.-X. & Liu, L. Mechanism of Ni-Catalyzed Selective C−O Bond Activation in Cross-Coupling of Aryl Esters. *J. Am. Chem. Soc.* **131**, 8815-8823 (2009).

19 Li, H. & Hall, M. B. Role of the Chemically Non-Innocent Ligand in the Catalytic Formation of Hydrogen and Carbon Dioxide from Methanol and

Water with the Metal as the Spectator. *J. Am. Chem. Soc.* **137**, 12330-12342 (2015).

20 Jiang, Y.-Y., Yan, L., Yu, H.-Z., Zhang, Q. & Fu, Y. Mechanism of Vanadium-Catalyzed Selective C–O and C–C Cleavage of Lignin Model Compound. *ACS Catal.* **6**, 4399-4410 (2016).

21 Lu T. & Chen F. Comparison of Computational Methods for Atomic Charges. *Acta Phys. -Chim. Sin.* **28**, 1-18 (2012).

22 Martínez-Araya, J. I. Why is the dual descriptor a more accurate local reactivity descriptor than Fukui functions? *J. Math. Chem.* **53**, 451-465 (2015).

23 Lu, T. & Chen, F. Multiwfn: A multifunctional wavefunction analyzer. *J. Comput. Chem.* **33**, 580-592 (2012).

24 Marcus, R. A. On the Theory of Oxidation-Reduction Reactions Involving Electron Transfer. I. *J. Chem. Phys.* **24**, 966-978 (1956).

25 Marcus, R. A. Electrostatic Free Energy and Other Properties of States Having Nonequilibrium Polarization. I. *J. Chem. Phys.* **24**, 979-989 (1956).

26 Marcus, R. A. On the Theory of Oxidation-Reduction Reactions Involving Electron Transfer. III. Applications to Data on the Rates of Organic Redox Reactions. *J. Chem. Phys.* **26**, 872-877 (1957).

27 Hush, N. S. Adiabatic Rate Processes at Electrodes. I. Energy-Charge Relationships. *J. Chem. Phys.* **28**, 962-972 (1958).

28 Marcus, R. A. ON THE THEORY OF ELECTROCHEMICAL AND CHEMICAL ELECTRON TRANSFER PROCESSES. *Can. J. Chem.* **37**, 155-163 (1959).

29 Hush, N. S. Adiabatic theory of outer sphere electron-transfer reactions in solution. *Trans. Faraday Soc.* **57**, 557-580 (1961).

30 Marcus, R. A. The second R. A. Robinson Memorial Lecture. Electron, proton and related transfers. *Faraday Discuss. Chem. Soc.* **74**, 7-15 (1982).

31 Marcus, R. A. & Sutin, N. Electron transfers in chemistry and biology. *Biochim. Biophys. Acta, Rev. Bioenerg.* **811**, 265-322 (1985).

32 Houmam, A. Electron Transfer Initiated Reactions: Bond Formation and Bond Dissociation. *Chem. Rev.* **108**, 2180-2237 (2008).

33 Lin, C. Y., Coote, M. L., Gennaro, A. & Matyjaszewski, K. Ab Initio Evaluation of the Thermodynamic and Electrochemical Properties of Alkyl Halides and Radicals and Their Mechanistic Implications for Atom Transfer Radical Polymerization. *J. Am. Chem. Soc.* **130**, 12762-12774 (2008).

34 Jones, G. O., Liu, P., Houk, K. N. & Buchwald, S. L. Computational Explorations of Mechanisms and Ligand-Directed Selectivities of Copper-Catalyzed Ullmann-Type Reactions. *J. Am. Chem. Soc.* **132**, 6205-6213 (2010).

35 Zhang, Q., Zhang, Z.-Q., Fu, Y. & Yu, H.-Z. Mechanism of the Visible Light-Mediated Gold-Catalyzed Oxyarylation Reaction of Alkenes. *ACS Catal.* **6**, 798-808 (2016).
