# Supplementary material for: Site-selective remote C(sp3)–H heteroarylation of amides via organic photoredox catalysis
Source: Nat Commun. 2019 Oct 18;10:4743. doi: 10.1038/s41467-019-12722-4 (PMC6800443; doi:10.1038/s41467-019-12722-4)
Supplement: Supplementary file 3 — Description of Additional Supplementary Files [file 41467_2019_12722_MOESM3_ESM.pdf]

Description of Additional Supplementary Files

File Name: Supplementary Data 1
Description: Coordinates and energies of DFT-computed stationary points
