# Supplementary material for: Site-selective remote C(sp3)–H heteroarylation of amides via organic photoredox catalysis
Source: Nat Commun. 2019 Oct 18;10:4743. doi: 10.1038/s41467-019-12722-4 (PMC6800443; doi:10.1038/s41467-019-12722-4)
Supplement: Supplementary file 4 — Supplementary Data 1 [file 41467_2019_12722_MOESM4_ESM.pdf]

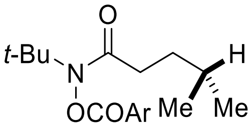

Ar = p-$CF_3C_6H_4$

1a

Zero-point correction= 0.402393 (Hartree/Particle)

Thermal correction to Energy= 0.427925

Thermal correction to Enthalpy= 0.428869

Thermal correction to Gibbs Free Energy= 0.344610

Sum of electronic and zero-point Energies= -1278.469746

Sum of electronic and thermal Energies= -1278.444214

Sum of electronic and thermal Enthalpies= -1278.443270

Sum of electronic and thermal Free Energies= -1278.527529

N	-2.25748900	-0.85386200	0.04054700
C	-2.97828000	-0.12817600	-0.87126100
C	-2.27879700	1.05247300	-1.52040100
O	-4.14586300	-0.39599500	-1.09616600
C	-2.03829000	2.20800800	-0.53842200
C	-3.26228800	2.60771200	0.29260400
C	-2.90234500	3.75700400	1.23049200
C	-4.45813600	2.96923300	-0.58594000
C	-2.76447200	-2.00567700	0.84151800
C	-1.63422600	-2.52912000	1.72675100
C	-3.23213600	-3.11842300	-0.10017200
C	-3.91122900	-1.51291200	1.72868000
H	-2.94481900	1.36768300	-2.33341400
H	-1.32728100	0.74575500	-1.97946000
H	-1.69981200	3.08232900	-1.11985900
H	-1.21328600	1.94580400	0.14154200
H	-3.54571800	1.73916800	0.91605600

H	-3.75029500	4.02373400	1.87902800
H	-2.62394900	4.65457400	0.65433600
H	-2.05036600	3.49591500	1.87680200
H	-5.30221200	3.32063200	0.02694600
H	-4.80515800	2.10391200	-1.16846700
H	-4.19325400	3.77751600	-1.28805200
H	-2.03968800	-3.34480900	2.34068500
H	-1.24597700	-1.75146800	2.39822000
H	-0.80270400	-2.94070400	1.13576900
H	-3.57132600	-3.97996900	0.49331900
H	-2.40354900	-3.43773200	-0.74727900
H	-4.06239400	-2.77413100	-0.72885500
H	-4.30829300	-2.35504700	2.31390600
H	-4.72319400	-1.09456600	1.12132600
H	-3.55137500	-0.74407800	2.42866400
O	-0.93603600	-0.50291300	0.20250800
C	-0.06693500	-1.12267100	-0.64681800
O	-0.41066200	-1.90188900	-1.48578400
C	1.33222800	-0.68120100	-0.38772400
C	2.33058700	-1.19452700	-1.22143600
C	1.65126300	0.21684700	0.63702400
C	3.65293600	-0.80810700	-1.03708700
H	2.06003100	-1.89154500	-2.01513400
C	2.97498500	0.60398200	0.82225900
H	0.87213100	0.61393900	1.28727700
C	3.96507900	0.09057600	-0.01588800
H	4.43713900	-1.19827200	-1.68701700
H	3.23376000	1.30803600	1.61401300
C	5.40075500	0.48245600	0.21034200
F	6.11119500	0.44428400	-0.91856000

F	6.00491800	-0.33875900	1.07694700
F	5.50836400	1.71579400	0.70875000

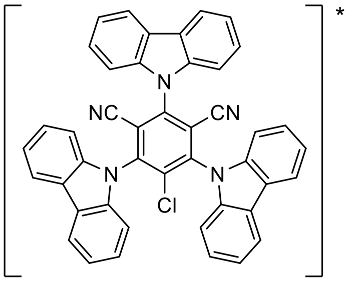

3CzClIPN*

Zero-point correction= 0.559127 (Hartree/Particle)

Thermal correction to Energy= 0.596781

Thermal correction to Enthalpy= 0.597725

Thermal correction to Gibbs Free Energy= 0.485566

Sum of electronic and zero-point Energies= -2422.157014

Sum of electronic and thermal Energies= -2422.119360

Sum of electronic and thermal Enthalpies= -2422.118416

Sum of electronic and thermal Free Energies= -2422.230575

C	-1.18676400	0.68144700	-0.27789300
C	-1.19616000	-0.77057800	-0.23326400
C	-0.00020600	-1.45228700	0.00046700
C	1.19610300	-0.77051900	0.23371300
C	1.18690900	0.68151900	0.27764300
C	0.00007800	1.37375700	-0.00026900
C	2.36575200	1.37393700	0.65498600
N	3.35099000	1.91471100	0.94640300
C	-2.36548700	1.37388600	-0.65558100
N	-3.35055500	1.91475200	-0.94734100
Cl	-0.00026500	-3.18334000	0.00116900
C	-4.62238600	-0.91161600	2.58063100
C	-5.72717800	-1.62883900	2.10122000
C	-5.70050000	-2.25262400	0.84785200

C	-4.54851100	-2.14416300	0.07556200
C	-3.44333100	-1.41561600	0.57619000
C	-3.45575500	-0.79141900	1.82609500
C	-4.15657100	-2.63593400	-1.24243800
C	-2.83964000	-2.16885600	-1.47020300
N	-2.41797300	-1.44182100	-0.36283800
C	-4.79220900	-3.39226300	-2.22228700
C	-4.10168800	-3.66596300	-3.40838900
C	-2.79950600	-3.18929000	-3.61879600
C	-2.14308200	-2.42881500	-2.65286400
C	-2.28629200	4.25215800	2.54804100
C	-2.02988800	5.60257800	2.25676900
C	-1.13075100	5.95805400	1.25462400
C	-0.49061300	4.94450900	0.53551300
C	-0.76796700	3.59159500	0.83546500
C	-1.66062400	3.22526300	1.84702500
C	0.49178000	4.94422100	-0.53694200
C	0.76863300	3.59114900	-0.83662200
N	0.00012600	2.77874000	-0.00055900
C	1.13234600	5.95739600	-1.25619700
C	2.03142200	5.60139800	-2.25820700
C	2.28732700	4.25081900	-2.54921000
C	1.66121400	3.22429500	-1.84804100
C	4.62062900	-0.91550600	-2.58195600
C	5.72540500	-1.63248700	-2.10231800
C	5.69929500	-2.25503000	-0.84819200
C	4.54783000	-2.14550900	-0.07546500
C	3.44259800	-1.41716200	-0.57634600
C	3.45440600	-0.79421800	-1.82688800
C	4.15641500	-2.63562400	1.24338900

C	2.83976600	-2.16774500	1.47128100
N	2.41777900	-1.44189200	0.36328900
C	4.79208900	-3.39101600	2.22377100
C	4.10187200	-3.66297000	3.41058300
C	2.80005500	-3.18550300	3.62110500
C	2.14353600	-2.42587400	2.65453200
H	-4.67334800	-0.43865300	3.56226700
H	-6.62460900	-1.70258300	2.71688100
H	-6.56526700	-2.81065800	0.48524700
H	-2.58763800	-0.24079000	2.19336200
H	-5.80816900	-3.76068400	-2.07137600
H	-4.58626100	-4.25827700	-4.18574900
H	-2.28971500	-3.41574000	-4.55627100
H	-1.13052600	-2.05241900	-2.80779600
H	-2.99047700	3.99869300	3.34210900
H	-2.53954300	6.38044200	2.82703500
H	-0.92478800	7.00693300	1.03386900
H	-1.85994100	2.17801400	2.07961700
H	0.92676100	7.00638900	-1.03564800
H	2.54144200	6.37895100	-2.82856800
H	2.99145400	3.99693900	-3.34319700
H	1.86011700	2.17692500	-2.08044000
H	4.67120700	-0.44361000	-3.56411600
H	6.62250900	-1.70713400	-2.71834800
H	6.56416900	-2.81286700	-0.48555500
H	2.58616800	-0.24378000	-2.19412900
H	5.80779800	-3.76013900	2.07295200
H	4.58658000	-4.25457600	4.18839400
H	2.29064700	-3.41061000	4.55910600
H	1.13123900	-2.04874000	2.80930000

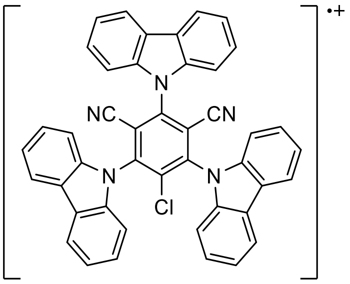

3CzClIPN $^{\bullet+}$

Zero-point correction= 0.563026 (Hartree/Particle)

Thermal correction to Energy= 0.600066

Thermal correction to Enthalpy= 0.601010

Thermal correction to Gibbs Free Energy= 0.491316

Sum of electronic and zero-point Energies= -2422.034365

Sum of electronic and thermal Energies= -2421.997326

Sum of electronic and thermal Enthalpies= -2421.996381

Sum of electronic and thermal Free Energies= -2422.106076

C	-1.14458000	0.76279900	-0.15295000
C	-1.27198500	-0.63707300	-0.15435700
C	-0.12109100	-1.42790500	0.01622500
C	1.13202200	-0.82492000	0.19470900
C	1.23871800	0.57666100	0.22154500
C	0.10314500	1.37636600	0.03424600
C	2.51095700	1.18653100	0.49090500
N	3.53381700	1.67615000	0.70162500
C	-2.30300000	1.57003400	-0.42775200
N	-3.23192900	2.21763900	-0.64594000
Cl	-0.25325500	-3.13725600	-0.01448700
C	-4.91194400	-0.49367700	2.42373900
C	-6.02026500	-1.16708300	1.88282600
C	-5.93187600	-1.81481900	0.65454900
C	-4.71725400	-1.78014200	-0.03914300
C	-3.61726900	-1.09602700	0.52107800

C	-3.69157700	-0.44811600	1.75510000
C	-4.27240900	-2.33008500	-1.31004500
C	-2.92035900	-1.95776400	-1.46968300
N	-2.52666600	-1.21673300	-0.34922300
C	-4.90485800	-3.07361400	-2.31264600
C	-4.17819600	-3.42155900	-3.44676600
C	-2.83718300	-3.02689100	-3.59291900
C	-2.18645600	-2.28546400	-2.61100200
C	-1.63618900	4.43547000	2.76992500
C	-1.29762100	5.75276300	2.42898000
C	-0.47638300	6.03175000	1.32909500
C	-0.00924800	4.96945300	0.56469900
C	-0.37392600	3.64813600	0.91473300
C	-1.17415100	3.35437600	2.02358600
C	0.85752400	4.87219100	-0.60586200
C	0.97716800	3.49706900	-0.91338300
N	0.22255800	2.77092200	0.00977900
C	1.50592600	5.80894300	-1.40118200
C	2.26393800	5.35326500	-2.48838900
C	2.36418200	3.98746600	-2.78332500
C	1.71851200	3.03000100	-2.00321000
C	4.72272600	-1.18091800	-2.41873600
C	5.71493700	-2.02357400	-1.90137300
C	5.54413700	-2.68267200	-0.67592800
C	4.36363300	-2.47910200	0.02809500
C	3.37349500	-1.62453900	-0.51027400
C	3.52835500	-0.96554800	-1.73316400
C	3.84519300	-2.97567100	1.29983300
C	2.56486800	-2.39827100	1.47121300
N	2.28921700	-1.59747200	0.36597700

C	4.35257400	-3.81345200	2.28616300
C	3.57252400	-4.05313600	3.42445300
C	2.31188700	-3.45885300	3.58370700
C	1.78463800	-2.61319800	2.61187500
H	-5.00547200	0.00492900	3.38980500
H	-6.96023500	-1.18231200	2.43608300
H	-6.79121300	-2.34165700	0.23599000
H	-2.83135700	0.07061200	2.18326400
H	-5.95018800	-3.36837700	-2.20471200
H	-4.65526600	-4.00139300	-4.23803600
H	-2.29177900	-3.30316300	-4.49677700
H	-1.14804700	-1.97226900	-2.73478300
H	-2.26690800	4.24947200	3.63981100
H	-1.67411900	6.57579200	3.03769400
H	-0.20685500	7.05900800	1.07986100
H	-1.42177900	2.32834700	2.30130900
H	1.42358000	6.87524900	-1.18598900
H	2.78043300	6.07646200	-3.12067900
H	2.95461900	3.66319800	-3.64085400
H	1.78675900	1.96708900	-2.24242300
H	4.88323900	-0.68242600	-3.37530000
H	6.63795800	-2.17097200	-2.46338800
H	6.32088200	-3.34080400	-0.28390400
H	2.75270000	-0.31204800	-2.13676400
H	5.33806100	-4.26884800	2.17906600
H	3.95639700	-4.70831400	4.20746100
H	1.73443300	-3.65612600	4.48752700
H	0.81106200	-2.13684600	2.74135600

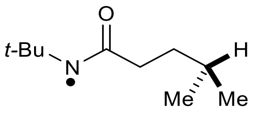

A

Zero-point correction= 0.286159 (Hartree/Particle)

Thermal correction to Energy= 0.301185

Thermal correction to Enthalpy= 0.302129

Thermal correction to Gibbs Free Energy= 0.243433

Sum of electronic and zero-point Energies= -522.118354

Sum of electronic and thermal Energies= -522.103327

Sum of electronic and thermal Enthalpies= -522.102383

Sum of electronic and thermal Free Energies= -522.161079

N	-0.91759100	0.26073100	-0.75339400
C	-0.40681000	1.41678600	-0.19393100
C	1.01346800	1.76436800	-0.59077600
O	-1.10158500	2.17162400	0.46290500
C	1.91686000	0.59536800	-0.97437800
C	2.03109400	-0.51807000	0.07362500
C	3.05669600	-1.55488000	-0.37494100
C	2.36387600	0.01822400	1.46334400
C	-1.81724600	-0.62169000	-0.02220500
C	-1.68593600	-2.01197600	-0.64885600
C	-3.23652300	-0.07593100	-0.28318200
C	-1.54317700	-0.68781300	1.48301400
H	1.43067100	2.35636100	0.23610700
H	0.92132200	2.45566800	-1.44561500
H	2.92395000	0.99776100	-1.17637600
H	1.56120600	0.15468600	-1.91934400
H	1.05153500	-1.02489400	0.13214000
H	3.09806200	-2.40521800	0.32212200
H	4.06385000	-1.10918100	-0.42272600

H	2.81582900	-1.94712600	-1.37487700
H	2.48675500	-0.80755300	2.18073500
H	1.57518300	0.67899400	1.85520600
H	3.30566000	0.59214800	1.44633000
H	-2.41589600	-2.69726600	-0.19449400
H	-0.67800400	-2.42120600	-0.48255200
H	-1.86904300	-1.96644600	-1.73192900
H	-3.96571800	-0.75460400	0.18378100
H	-3.44044900	-0.02755000	-1.36225100
H	-3.34639000	0.92702300	0.14851800
H	-2.25037500	-1.38705300	1.95191900
H	-1.66250000	0.29563900	1.95541600
H	-0.52364200	-1.05460500	1.67812800

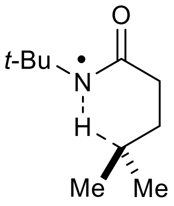

TS1

Zero-point correction= 0.281955 (Hartree/Particle)

Thermal correction to Energy= 0.296285

Thermal correction to Enthalpy= 0.297230

Thermal correction to Gibbs Free Energy= 0.241219

Sum of electronic and zero-point Energies= -522.109235

Sum of electronic and thermal Energies= -522.094904

Sum of electronic and thermal Enthalpies= -522.093960

Sum of electronic and thermal Free Energies= -522.149971

N	-0.54559700	0.18101200	-0.49801300
C	-0.32743200	1.48220900	-0.14410400
C	1.12883500	1.93394500	-0.12011900
O	-1.23167200	2.26904100	0.09896600

C	2.13479100	0.90485600	-0.62592100
C	1.89404500	-0.46601600	-0.00460400
C	2.50733700	-1.60933300	-0.78478400
C	2.14858400	-0.54141100	1.48772200
C	-1.67417400	-0.61967700	-0.01912600
C	-1.34710700	-2.08081400	-0.33458400
C	-2.92014800	-0.19140600	-0.81078900
C	-1.90009100	-0.44498200	1.48448600
H	1.33234100	2.20864400	0.92756600
H	1.18109400	2.87164300	-0.69192300
H	3.16306200	1.24547300	-0.41606000
H	2.04238100	0.81321300	-1.71973300
H	0.70999600	-0.52190100	-0.17900100
H	2.25738000	-2.58345700	-0.33910600
H	3.60767100	-1.51590800	-0.78833000
H	2.16862900	-1.60632400	-1.83158500
H	1.83367700	-1.51350400	1.89563800
H	1.62239800	0.24948600	2.04198300
H	3.22836600	-0.42998800	1.68952300
H	-2.20013800	-2.72472100	-0.07673200
H	-0.47375800	-2.41763500	0.24579100
H	-1.12264400	-2.20408400	-1.40463600
H	-3.77108400	-0.82819100	-0.52536400
H	-2.74402900	-0.30480600	-1.89057500
H	-3.16385300	0.85711300	-0.59927700
H	-2.73283300	-1.08013000	1.82087100
H	-2.13742100	0.60010300	1.72462100
H	-0.99651700	-0.73866400	2.04162400

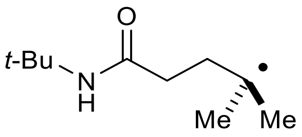

B

Zero-point correction= 0.285846 (Hartree/Particle)

Thermal correction to Energy= 0.301292

Thermal correction to Enthalpy= 0.302236

Thermal correction to Gibbs Free Energy= 0.243120

Sum of electronic and zero-point Energies= -522.143085

Sum of electronic and thermal Energies= -522.127638

Sum of electronic and thermal Enthalpies= -522.126694

Sum of electronic and thermal Free Energies= -522.185811

N	-0.61425600	0.02957800	-0.23180000
C	-0.36340900	1.34567300	-0.05114200
C	1.08564200	1.79063200	-0.21727900
O	-1.22416400	2.16580000	0.23969900
C	2.09345800	0.79997200	-0.79650300
C	2.39531600	-0.42067600	0.02896300
C	3.27838200	-1.45142200	-0.59372500
C	2.32907400	-0.34502200	1.51991700
C	-1.91775700	-0.62583700	-0.04668200
C	-1.70042400	-2.10695100	-0.35191300
C	-2.94649400	-0.04169000	-1.01831900
C	-2.38965900	-0.45828200	1.40069800
H	1.40857500	2.14123400	0.77615300
H	1.04246400	2.69070800	-0.84734700
H	3.03981700	1.35493200	-0.96292200
H	1.76989100	0.48676500	-1.80515600
H	0.17886200	-0.57799200	-0.41840600
H	3.18089900	-2.42773700	-0.09438600

H	4.34712100	-1.16286400	-0.52283100
H	3.05750300	-1.58335100	-1.66431900
H	2.46295900	-1.33853300	1.97309500
H	1.36922600	0.06227300	1.87645000
H	3.12334500	0.30909600	1.93381800
H	-2.63983100	-2.66231200	-0.22173500
H	-0.94755700	-2.53831100	0.32649000
H	-1.35578600	-2.24592600	-1.38816700
H	-3.90532100	-0.57111100	-0.91486000
H	-2.59737700	-0.15379200	-2.05609400
H	-3.10523200	1.02471900	-0.81434000
H	-3.35455300	-0.96628500	1.54694800
H	-2.50899000	0.60563800	1.64352200
H	-1.65768800	-0.89897200	2.09502000

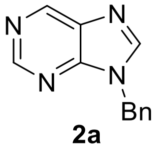

2a

Zero-point correction= 0.207481 (Hartree/Particle)

Thermal correction to Energy= 0.218922

Thermal correction to Enthalpy= 0.219866

Thermal correction to Gibbs Free Energy= 0.167737

Sum of electronic and zero-point Energies= -681.353328

Sum of electronic and thermal Energies= -681.341887

Sum of electronic and thermal Enthalpies= -681.340943

Sum of electronic and thermal Free Energies= -681.393072

N	3.45633500	-1.46479900	-0.54831900
C	2.47950500	-1.93349400	0.24107900
N	1.44334400	-1.25162100	0.71822000
C	1.43481500	0.01901600	0.33999500
C	2.40026000	0.63958200	-0.48301300

C	3.43621500	-0.18655000	-0.91907900
N	0.53235100	0.99802900	0.64653600
C	0.97600500	2.12953000	0.01241800
N	2.08043700	1.96560400	-0.66645500
C	-1.81700900	0.24798300	0.61973100
C	-2.67354000	1.08751200	-0.10052900
C	-3.69952900	0.54965100	-0.87722800
C	-3.87704200	-0.83342900	-0.93925500
C	-3.02643000	-1.67608000	-0.22237000
C	-2.00001000	-1.13760000	0.55398500
C	-0.68527400	0.83316600	1.43391800
H	2.54319000	-2.98896500	0.52014400
H	4.24127300	0.18181300	-1.56245500
H	0.41735600	3.06165400	0.10643200
H	-2.53837200	2.17070400	-0.04620300
H	-4.36568800	1.21259900	-1.43226500
H	-4.68176100	-1.25483300	-1.54444100
H	-3.16446900	-2.75796400	-0.26524900
H	-1.32929300	-1.79399400	1.11283200
H	-0.95655600	1.82266700	1.82168800
H	-0.44073800	0.18010700	2.28174100

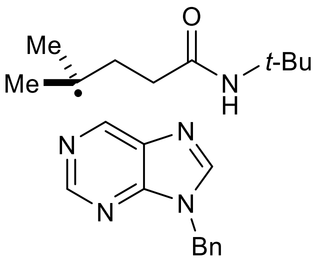

Comlpex I

Zero-point correction= 0.494756 (Hartree/Particle)

Thermal correction to Energy= 0.523403

Thermal correction to Enthalpy= 0.524347

Thermal correction to Gibbs Free Energy= 0.431360

Sum of electronic and zero-point Energies= -1203.507004

Sum of electronic and thermal Energies= -1203.478357

Sum of electronic and thermal Enthalpies= -1203.477413

Sum of electronic and thermal Free Energies= -1203.570400

N	3.59814300	-1.24044600	0.49698400
C	4.24086300	-0.14224000	0.03116700
C	4.21993300	1.04516000	0.98105100
O	4.77660600	-0.08453000	-1.06748600
C	2.80358700	1.63788300	1.15526300
C	2.20508000	2.14098600	-0.12112200
C	3.49612400	-2.53723100	-0.19063600
C	2.71885100	-3.46321200	0.74276300
C	4.89401800	-3.11151200	-0.44074200
C	2.73774100	-2.38119600	-1.51143400
H	4.89790800	1.80105200	0.56297100
H	4.61001900	0.73209600	1.96239800
H	2.87616600	2.46218700	1.88388100
H	2.14439300	0.87291800	1.60073800
H	3.22136100	-1.18875400	1.43829600
H	2.60518000	-4.45498400	0.28328100
H	1.71517600	-3.05905300	0.94522000
H	3.24617500	-3.58430800	1.70154600
H	4.81715700	-4.09766300	-0.92229800
H	5.43276700	-3.23108600	0.51156600
H	5.47374700	-2.44399500	-1.09143800
H	2.65815100	-3.35562200	-2.01628200
H	3.26225500	-1.68140600	-2.17538600
H	1.71938200	-2.00481400	-1.32967200
C	2.60485800	3.47978700	-0.64679300

H	2.93439000	4.15385100	0.15858800
H	3.43707600	3.40819200	-1.37578000
H	1.76975500	3.96451400	-1.18134800
C	1.56940400	1.17890000	-1.07076300
H	1.15535900	0.30386200	-0.54548300
H	0.75951100	1.65893500	-1.64781700
H	2.29889200	0.79599500	-1.81184800
N	-0.96907700	3.62480000	0.13979000
C	-1.71876600	3.09377900	-0.83766100
N	-2.06801700	1.81856300	-0.97421200
C	-1.60767200	1.04480800	0.00060700
C	-0.83229400	1.47617400	1.09682900
C	-0.51242400	2.83435500	1.10948200
N	-1.72563600	-0.30939200	0.14715800
C	-1.04875700	-0.62410800	1.29659100
N	-0.50020800	0.40338700	1.89205400
C	-3.94172100	-1.22298600	-0.43771400
C	-4.79710900	-0.32300400	-1.08294700
C	-6.15919900	-0.30733300	-0.78204300
C	-6.67652800	-1.19156800	0.16563900
C	-5.82857000	-2.09343700	0.81050500
C	-4.46677600	-2.10868800	0.50934900
C	-2.45881500	-1.21419200	-0.73241500
H	-2.07232600	3.78361300	-1.60911500
H	0.10676300	3.27810300	1.89565800
H	-0.99786600	-1.65990700	1.63425900
H	-4.38711800	0.37196000	-1.81927400
H	-6.81955100	0.39715900	-1.29097100
H	-7.74260500	-1.18038400	0.39956600
H	-6.22999400	-2.79049300	1.54830600

H	-3.80329100	-2.81857400	1.00967600
H	-2.02831600	-2.21429200	-0.59775800
H	-2.27076500	-0.88799800	-1.76386800

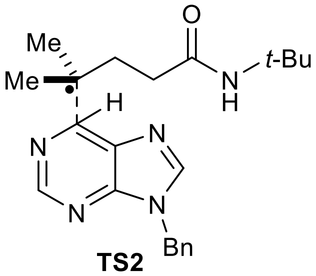

TS2

Zero-point correction= 0.495644 (Hartree/Particle)

Thermal correction to Energy= 0.522774

Thermal correction to Enthalpy= 0.523719

Thermal correction to Gibbs Free Energy= 0.435525

Sum of electronic and zero-point Energies= -1203.497102

Sum of electronic and thermal Energies= -1203.469972

Sum of electronic and thermal Enthalpies= -1203.469027

Sum of electronic and thermal Free Energies= -1203.557221

N	3.63115200	-1.13012600	0.48092600
C	4.27167200	0.03607300	0.23278800
C	3.98179000	1.14576800	1.23471200
O	5.02769000	0.20704900	-0.71418700
C	2.49333100	1.51411700	1.38342300
C	1.88737700	2.09267700	0.13067100
C	3.75539800	-2.36624800	-0.30774600
C	2.83555300	-3.39274200	0.35020700
C	5.20247900	-2.86615900	-0.27468800
C	3.30578000	-2.12659200	-1.75181100
H	4.56950900	2.01753200	0.91982600
H	4.35861300	0.82379800	2.21857800
H	2.41591000	2.25332000	2.19644300
H	1.91809300	0.62988400	1.70023400

H	3.06330100	-1.17817100	1.32102900
H	2.87917500	-4.34446600	-0.19750200
H	1.79293900	-3.03874500	0.34679700
H	3.13770400	-3.57908300	1.39231900
H	5.29022200	-3.81049100	-0.83236600
H	5.52114000	-3.04438000	0.76354900
H	5.87469300	-2.12532500	-0.72725600
H	3.39316300	-3.05993100	-2.32777000
H	3.92950000	-1.35909600	-2.22805600
H	2.25498500	-1.80139100	-1.78112200
C	2.32550000	3.47809100	-0.23396000
H	2.29256200	4.15719200	0.63193800
H	3.36916500	3.45167100	-0.59836700
H	1.70374200	3.89594400	-1.03730700
C	1.66381300	1.16026000	-1.02131400
H	1.31644100	0.17207300	-0.68652800
H	0.93720400	1.57942000	-1.73639200
H	2.60828900	1.01433600	-1.57678200
N	-0.65398400	3.29496500	-0.11721300
C	-1.47955900	2.83440500	-1.04750500
N	-1.96738500	1.59273500	-1.17193600
C	-1.54699000	0.78479900	-0.20027700
C	-0.68481800	1.12912900	0.85515700
C	-0.14340100	2.44263900	0.81646600
N	-1.85291300	-0.53732300	-0.00590000
C	-1.18625700	-0.91909000	1.12427000
N	-0.47401000	0.04230700	1.66449700
C	-4.20474300	-1.16475200	-0.41211900
C	-4.96531500	-0.11703400	-0.94408800
C	-6.28825600	0.06691600	-0.54247200

C	-6.86224100	-0.79548100	0.39324300
C	-6.10954300	-1.84298000	0.92570000
C	-4.78603800	-2.02605000	0.52419800
C	-2.75868500	-1.34221400	-0.81708300
H	-1.81833200	3.55705900	-1.79731000
H	0.31560000	2.89319500	1.70160500
H	-1.26874300	-1.94071900	1.49582000
H	-4.50876500	0.55963800	-1.67002300
H	-6.87448800	0.88567700	-0.96365800
H	-7.89838300	-0.65260000	0.70534700
H	-6.55554300	-2.52261600	1.65409300
H	-4.19768400	-2.84916600	0.93734600
H	-2.45136800	-2.38960400	-0.70731900
H	-2.61127800	-1.04246000	-1.86293100

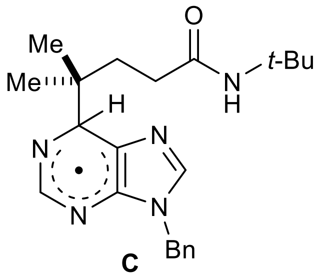

C

Zero-point correction= 0.498530 (Hartree/Particle)

Thermal correction to Energy= 0.525106

Thermal correction to Enthalpy= 0.526050

Thermal correction to Gibbs Free Energy= 0.440928

Sum of electronic and zero-point Energies= -1203.519734

Sum of electronic and thermal Energies= -1203.493157

Sum of electronic and thermal Enthalpies= -1203.492213

Sum of electronic and thermal Free Energies= -1203.577335

N	3.32637900	-1.13615200	0.51072200
C	4.16628000	-0.08156800	0.37393500
C	3.89252100	1.11297800	1.28111700

O	5.13343400	-0.08320600	-0.37585800
C	2.43709300	1.52426700	1.48892100
C	1.77051500	2.25238900	0.29951700
C	3.42518000	-2.39818500	-0.24006800
C	2.35439400	-3.32636000	0.33032400
C	4.80150200	-3.03808700	-0.03670500
C	3.16141400	-2.14691300	-1.72787200
H	4.49360300	1.94045200	0.87986300
H	4.32943600	0.85255200	2.25929400
H	2.38854100	2.20284600	2.35562500
H	1.83823500	0.64949900	1.77749500
H	2.47062000	-0.99730100	1.04144500
H	2.34849600	-4.27939500	-0.21714000
H	1.35715200	-2.87118300	0.24037100
H	2.54606900	-3.53499300	1.39392600
H	4.82811100	-4.02151100	-0.52911100
H	4.99749400	-3.18384600	1.03662300
H	5.59400300	-2.40721900	-0.45576900
H	3.27122100	-3.08077000	-2.29949200
H	3.87323500	-1.40841100	-2.12188100
H	2.13778600	-1.76984200	-1.87930400
C	2.22530700	3.71352600	0.29413900
H	1.85008600	4.24576300	1.18211800
H	3.32487700	3.76243300	0.30444700
H	1.86569400	4.24320600	-0.59750300
C	2.10018300	1.61486200	-1.05285900
H	1.87072600	0.53971900	-1.06855000
H	1.52087300	2.10447900	-1.85193200
H	3.16731700	1.73600600	-1.29256600
N	-0.47265400	3.19745700	-0.23958200

C	-1.49576500	2.92404300	-0.96881000
N	-2.11080800	1.70178000	-1.17140100
C	-1.50610000	0.73342800	-0.51454100
C	-0.37093900	0.83564300	0.32763700
C	0.22321400	2.18870600	0.53974700
N	-1.82837400	-0.60641900	-0.52763700
C	-0.92007900	-1.22328800	0.26024500
N	-0.02763000	-0.38731100	0.78529100
C	-4.25187000	-1.03505300	-0.44365900
C	-5.10459300	0.04351500	-0.69991000
C	-6.27298900	0.20928800	0.04479400
C	-6.59600400	-0.70107800	1.05160800
C	-5.74898900	-1.78021300	1.31114000
C	-4.58259500	-1.94649700	0.56532800
C	-2.96625100	-1.19967900	-1.22205200
H	-1.96022800	3.75284200	-1.51583200
H	0.09460100	2.47672900	1.60499700
H	-0.94565600	-2.30151400	0.41723800
H	-4.84357200	0.75822500	-1.48334400
H	-6.93400200	1.05262100	-0.16309700
H	-7.51062700	-0.57191600	1.63313800
H	-6.00038900	-2.49745400	2.09451100
H	-3.92270700	-2.79509700	0.76335200
H	-2.73479400	-2.26101200	-1.37417900
H	-3.04063800	-0.71008500	-2.20149300

K_2CO_3

Zero-point correction= 0.017210 (Hartree/Particle)

Thermal correction to Energy= 0.024014

Thermal correction to Enthalpy= 0.024958

Thermal correction to Gibbs Free Energy= -0.016365

Sum of electronic and zero-point Energies= -1463.324170

Sum of electronic and thermal Energies= -1463.317366

Sum of electronic and thermal Enthalpies= -1463.316422

Sum of electronic and thermal Free Energies= -1463.357746

C	-0.00020300	0.82703200	0.00223000
O	1.11756000	1.44339300	0.00317100
O	-1.11806800	1.44339500	-0.00486100
O	0.00000600	-0.49180500	0.00746200
K	2.48779900	-0.63468300	-0.00232700
K	-2.48752400	-0.63489900	-0.00080800

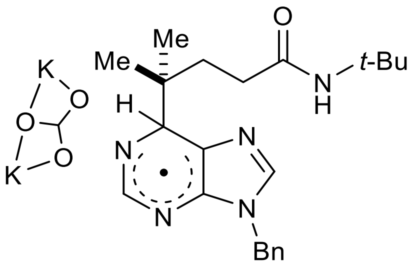

Complex II

Zero-point correction= 0.517247 (Hartree/Particle)

Thermal correction to Energy= 0.551905

Thermal correction to Enthalpy= 0.552850

Thermal correction to Gibbs Free Energy= 0.449026

Sum of electronic and zero-point Energies= -2666.883420

Sum of electronic and thermal Energies= -2666.848762

Sum of electronic and thermal Enthalpies= -2666.847817

Sum of electronic and thermal Free Energies= -2666.951641

N	3.90461800	-0.90000800	0.15374900
C	4.75579600	0.15259500	0.17061400
C	4.21324500	1.48470500	0.67629700
O	5.93963300	0.06284500	-0.12997300
C	2.70914000	1.72870400	0.67313600
C	2.08313200	2.13807800	-0.68140200

C	4.28582900	-2.27078800	-0.22589200
C	3.01570000	-3.11626500	-0.15049100
C	5.32696600	-2.81191000	0.75806300
C	4.83266900	-2.30481400	-1.65678100
H	4.75459000	2.26168600	0.11685500
H	4.58511200	1.55343400	1.71195100
H	2.48378900	2.53747800	1.38454800
H	2.18806800	0.85396600	1.08394300
H	2.91361300	-0.73569200	0.31998200
H	3.25130700	-4.16823800	-0.36514600
H	2.27627800	-2.77149700	-0.88839000
H	2.56324000	-3.06008600	0.85278500
H	5.59418500	-3.84656200	0.49614400
H	4.92343700	-2.80679600	1.78221100
H	6.23495400	-2.19546000	0.73386300
H	5.06357400	-3.34114300	-1.94573400
H	5.74524900	-1.70100700	-1.73542600
H	4.08542400	-1.90945500	-2.36224300
C	2.36331500	3.62512400	-0.91110300
H	1.85253700	4.24091100	-0.15438400
H	3.44452600	3.82026300	-0.83884800
H	2.02125800	3.95193400	-1.90156100
C	2.61971700	1.34237800	-1.87558000
H	2.48026900	0.25925700	-1.75350500
H	2.09210100	1.65361900	-2.79163500
H	3.69368200	1.53320900	-2.03015400
N	-0.20618500	2.67061600	-1.53063600
C	-1.27468800	2.21094500	-2.07378800
N	-1.83860400	0.94984400	-1.93677600
C	-1.06967700	0.14932300	-1.22296700

C	0.11520800	0.49042800	-0.52772800
C	0.54666000	1.91602400	-0.55106400
N	-1.24113600	-1.20391300	-1.01796100
C	-0.20535800	-1.60367900	-0.25009700
N	0.62601800	-0.61395500	0.06671200
C	-3.63341400	-1.80674200	-0.95275000
C	-4.69781400	-1.29349800	-1.70121900
C	-5.93603900	-1.05541000	-1.09767600
C	-6.11487400	-1.32935400	0.26010800
C	-5.05479700	-1.84743100	1.01136600
C	-3.82002900	-2.08425900	0.40722200
C	-2.28528500	-2.03935700	-1.59531200
H	-1.83100800	2.87245500	-2.74851600
H	0.26577700	2.34606400	0.44261500
H	-0.08553700	-2.64676100	0.04621800
H	-4.55127300	-1.06868100	-2.76008400
H	-6.75929000	-0.65038700	-1.68847700
H	-7.07940000	-1.14022200	0.73436000
H	-5.19103100	-2.06250500	2.07254800
H	-2.98794500	-2.47374500	0.99829000
H	-1.96695200	-3.08132100	-1.46076000
H	-2.33626100	-1.82881400	-2.67152700
C	-1.43998400	1.60703100	1.85379900
O	-0.32639000	1.82313700	2.45441700
O	-2.05111400	2.54046300	1.23441600
O	-1.93304800	0.39389200	1.84671600
K	0.16987700	-0.58376600	2.88664600
K	-3.82684600	1.05915200	0.26647500

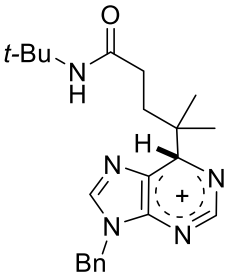

E

Zero-point correction=			0.499286 (Hartree/Particle)
Thermal correction to Energy=			0.525927
Thermal correction to Enthalpy=			0.526871
Thermal correction to Gibbs Free Energy=			0.441884
Sum of electronic and zero-point Energies=			-1203.328003
Sum of electronic and thermal Energies=			-1203.301362
Sum of electronic and thermal Enthalpies=			-1203.300418
Sum of electronic and thermal Free Energies=			-1203.385405
N	-3.50613000	-1.12505700	-0.56317400
C	-4.19512500	0.01401600	-0.31816000
C	-3.89483800	1.16967700	-1.26761600
O	-5.01824000	0.12565400	0.58001900
C	-2.41623200	1.50426600	-1.47810700
C	-1.73684100	2.23820700	-0.30328300
C	-3.61963500	-2.37734800	0.20096200
C	-2.68066200	-3.37914100	-0.46773300
C	-5.05625300	-2.90510100	0.15083800
C	-3.18133100	-2.14514800	1.64970600
H	-4.45701800	2.03391900	-0.88995500
H	-4.32976700	0.90232100	-2.24398300
H	-2.32211000	2.14886000	-2.36609900
H	-1.86230000	0.58650900	-1.71716600
H	-2.87525200	-1.13200800	-1.35776100
H	-2.69171100	-4.33137400	0.08067500

H	-1.64812000	-2.99702100	-0.47842900
H	-2.99031200	-3.57315000	-1.50612300
H	-5.12179200	-3.86870400	0.67763400
H	-5.36915700	-3.05802500	-0.89322000
H	-5.74527900	-2.19415900	0.62382000
H	-3.28498000	-3.07430100	2.22957700
H	-3.79854000	-1.36666400	2.11752800
H	-2.12624300	-1.83289400	1.68657700
C	-2.14515100	3.71048900	-0.30964600
H	-1.76302900	4.22488000	-1.20396200
H	-3.24290000	3.77742000	-0.31620800
H	-1.77480500	4.23637500	0.57935400
C	-2.05195800	1.61259600	1.05732900
H	-1.86826100	0.52763800	1.06206000
H	-1.44249300	2.07950500	1.84657700
H	-3.10927700	1.77381600	1.30997300
N	0.53268900	3.16305400	0.18271200
C	1.54133000	2.92354700	0.92047200
N	2.13405000	1.65583800	1.14211500
C	1.57236700	0.70928900	0.51689200
C	0.39783900	0.80371600	-0.36533300
C	-0.17055000	2.15013200	-0.55261900
N	1.88901900	-0.65314600	0.52988000
C	0.99650200	-1.23604800	-0.24356100
N	0.07688900	-0.38419300	-0.80185000
C	4.31135400	-1.07206500	0.46076000
C	5.16437500	0.00016600	0.73826200
C	6.33313400	0.17417000	-0.00367700
C	6.65221300	-0.72061200	-1.02534700
C	5.80320900	-1.79352800	-1.30389200

C	4.63634700	-1.96949500	-0.56218700
C	3.03014400	-1.25268500	1.23905300
H	2.03273500	3.73977500	1.45475900
H	-0.05760000	2.41529100	-1.62693000
H	0.99750400	-2.31495700	-0.41251900
H	4.91047100	0.69894100	1.53840000
H	6.99732900	1.01096300	0.21886000
H	7.56736800	-0.58453400	-1.60430800
H	6.05347800	-2.49832600	-2.09854900
H	3.97397300	-2.81288500	-0.77298400
H	2.78710700	-2.31225300	1.37802600
H	3.07986200	-0.76027000	2.21777200

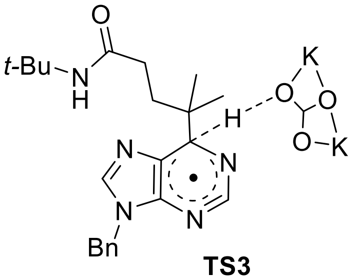

TS3

Zero-point correction= 0.513951 (Hartree/Particle)

Thermal correction to Energy= 0.548015

Thermal correction to Enthalpy= 0.548959

Thermal correction to Gibbs Free Energy= 0.446701

Sum of electronic and zero-point Energies= -2666.881795

Sum of electronic and thermal Energies= -2666.847732

Sum of electronic and thermal Enthalpies= -2666.846788

Sum of electronic and thermal Free Energies= -2666.949046

N	3.73162500	-0.80574200	0.02305100
C	4.52148900	0.23891800	0.38203500
C	3.84540700	1.45562800	1.00569900
O	5.74204300	0.21315900	0.29684900

C	2.34025000	1.63024300	0.85498700
C	1.83864400	2.27127400	-0.45980100
C	4.24139100	-2.05313800	-0.57510900
C	3.02550600	-2.92249300	-0.89073300
C	5.14442300	-2.77991200	0.42506300
C	5.00071600	-1.76112400	-1.87446100
H	4.40139000	2.33204400	0.64054100
H	4.09382700	1.37674200	2.07699800
H	1.95824200	2.24096500	1.68460600
H	1.84202500	0.66703000	1.01185600
H	2.72467600	-0.66115900	-0.04472600
H	3.35364300	-3.88204000	-1.31487600
H	2.36827900	-2.42712100	-1.62052700
H	2.44125000	-3.13630800	0.01917900
H	5.50669500	-3.72294000	-0.01070100
H	4.58485600	-3.01683700	1.34315100
H	6.00821600	-2.15675900	0.68882200
H	5.33444000	-2.70293300	-2.33519800
H	5.87721000	-1.13165900	-1.67707900
H	4.34610000	-1.24165000	-2.59133900
C	2.01200900	3.78806500	-0.35605700
H	1.37888100	4.19927100	0.44576100
H	3.06015700	4.03369600	-0.12472400
H	1.73976900	4.28942300	-1.29408400
C	2.58129100	1.77155700	-1.70624000
H	2.48808700	0.68388000	-1.83670000
H	2.15957900	2.25457300	-2.60209800
H	3.65340900	2.02260700	-1.66477100
N	-0.40016800	2.79861700	-1.44946000
C	-1.51399700	2.41943500	-1.99302800

N	-2.07929900	1.16883600	-1.97772600
C	-1.25332000	0.26618800	-1.44861500
C	-0.02111000	0.50919700	-0.81643900
C	0.33995200	1.92096600	-0.59779800
N	-1.42422700	-1.09920900	-1.42293300
C	-0.32440600	-1.60983100	-0.81829800
N	0.53529300	-0.67698200	-0.43402600
C	-3.79543300	-1.59106400	-1.04719900
C	-4.88787200	-0.85591800	-1.51986100
C	-5.97701100	-0.59307700	-0.68417000
C	-5.97918000	-1.06495100	0.63066100
C	-4.89262600	-1.80626000	1.10563100
C	-3.80607800	-2.06685600	0.27006200
C	-2.58406800	-1.82445800	-1.92295200
H	-2.08724800	3.17083400	-2.54870100
H	-0.19655000	2.14036900	0.46230200
H	-0.19303800	-2.68603800	-0.69478400
H	-4.87764000	-0.47541800	-2.54349700
H	-6.82227100	-0.01405100	-1.05991600
H	-6.82679200	-0.85587700	1.28554500
H	-4.88990100	-2.17660100	2.13210200
H	-2.94990000	-2.63132200	0.64762100
H	-2.31320400	-2.88781700	-1.94283600
H	-2.78415900	-1.49456100	-2.95059900
C	-0.92695700	1.01448300	2.22401400
O	0.07072900	0.99018500	3.00074100
O	-1.26907500	2.11688800	1.60682300
O	-1.60739800	-0.05853200	1.97717400
K	0.55341100	-1.40711500	2.36173900
K	-3.44746900	1.08902000	0.64190800

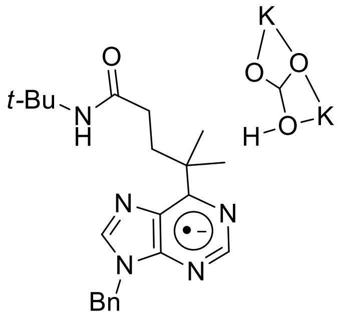

Complex III

Zero-point correction=	0.516056 (Hartree/Particle)		
Thermal correction to Energy=	0.551143		
Thermal correction to Enthalpy=	0.552087		
Thermal correction to Gibbs Free Energy=	0.448129		
Sum of electronic and zero-point Energies=	-2666.913150		
Sum of electronic and thermal Energies=	-2666.878063		
Sum of electronic and thermal Enthalpies=	-2666.877119		
Sum of electronic and thermal Free Energies=	-2666.981076		
N	3.75502700	-0.87602700	0.12465100
C	4.58543800	0.14577200	0.46729600
C	3.95498000	1.43544200	0.98341300
O	5.80497700	0.04569300	0.45868400
C	2.46693500	1.67871500	0.76801200
C	2.02603600	2.27404400	-0.59522200
C	4.23762000	-2.18045200	-0.36852400
C	3.00436800	-3.03423800	-0.66019400
C	5.08366100	-2.87019000	0.70497800
C	5.04331900	-2.00481200	-1.66050600
H	4.57084900	2.25611200	0.58568800
H	4.16425000	1.41135400	2.06592600
H	2.09263700	2.35138100	1.55183600
H	1.91720000	0.74730900	0.94548200
H	2.77397800	-0.66867000	-0.07730300

H	3.31552100	-4.02429100	-1.02265600
H	2.37281600	-2.56469200	-1.42734400
H	2.39935500	-3.18559700	0.24922200
H	5.39754700	-3.86396700	0.35234900
H	4.49583200	-3.00088500	1.62687200
H	5.97654700	-2.27783200	0.93803800
H	5.36773300	-2.98508400	-2.04081100
H	5.92986400	-1.38417900	-1.47994500
H	4.42487200	-1.52374500	-2.43414300
C	2.25532200	3.78561500	-0.56482500
H	1.64841100	4.25990000	0.22116900
H	3.31566400	4.00658900	-0.36418700
H	1.98106700	4.25028300	-1.52254700
C	2.83740100	1.68746000	-1.77052600
H	2.66310400	0.60827100	-1.88899000
H	2.53398800	2.17970000	-2.70782900
H	3.92078700	1.85110000	-1.64457200
N	-0.33847900	2.90954400	-1.17755100
C	-1.55912800	2.60520700	-1.54853200
N	-2.12323200	1.35353900	-1.66813900
C	-1.22094200	0.39851500	-1.36212300
C	0.08852600	0.58998900	-0.89170500
C	0.55862800	1.94848500	-0.80813100
N	-1.39448300	-0.95793200	-1.43508700
C	-0.23182900	-1.54380700	-0.99537300
N	0.67471200	-0.62744200	-0.65797600
C	-3.74807000	-1.60135300	-1.04302700
C	-4.96868000	-1.06901400	-1.47313500
C	-6.05843700	-0.99886900	-0.60025500
C	-5.93288600	-1.46049900	0.71225900

C	-4.71779200	-2.00140400	1.14493500
C	-3.63248800	-2.07204800	0.27097900
C	-2.55605400	-1.64202300	-1.97303800
H	-2.23169500	3.43818300	-1.78685400
H	-0.99973900	2.92955300	1.46564500
H	-0.10168100	-2.62499700	-0.97472400
H	-5.06030700	-0.69251900	-2.49456300
H	-7.00327000	-0.57476600	-0.94432400
H	-6.77952500	-1.39940200	1.39805100
H	-4.61462600	-2.36251600	2.16961600
H	-2.67777200	-2.47685600	0.61614300
H	-2.25842600	-2.68244100	-2.16415800
H	-2.82115200	-1.18190200	-2.93489600
C	-1.10331200	1.12533000	2.09789100
O	0.00643200	1.24659700	2.63073500
O	-1.69163900	2.25958000	1.57713600
O	-1.78911200	0.08604000	1.96437300
K	0.52223100	-1.24540700	2.07732700
K	-3.77747300	1.05037400	0.52094800

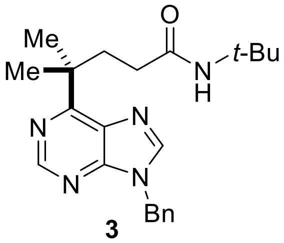

3

Zero-point correction= 0.488726 (Hartree/Particle)

Thermal correction to Energy= 0.515021

Thermal correction to Enthalpy= 0.515965

Thermal correction to Gibbs Free Energy= 0.431066

Sum of electronic and zero-point Energies= -1202.990003

Sum of electronic and thermal Energies= -1202.963708

Sum of electronic and thermal Enthalpies= -1202.962764

Sum of electronic and thermal Free Energies= -1203.047663

N	3.21706300	-0.99358200	0.44565700
C	3.98838100	-0.04871700	1.03245600
C	3.27720400	1.05001900	1.81697700
O	5.21278100	-0.07484000	1.01734000
C	1.82557000	1.37385800	1.48513700
C	1.59713500	2.23690900	0.21223400
C	3.74562700	-2.13174800	-0.32637100
C	2.53698800	-2.92371900	-0.82384100
C	4.60501200	-3.02303800	0.57472400
C	4.55993300	-1.63390500	-1.52533200
H	3.92152800	1.93770400	1.74145100
H	3.33624200	0.72591600	2.86907700
H	1.39193100	1.92911500	2.33117900
H	1.24783300	0.44105700	1.43227700
H	2.21156100	-0.84083400	0.38036700
H	2.87333700	-3.80722900	-1.38459300
H	1.91193000	-2.30740100	-1.48685400
H	1.91899100	-3.26465700	0.02151700
H	4.95178900	-3.90317900	0.01285900
H	4.01668100	-3.37225500	1.43699500
H	5.47771900	-2.47073200	0.94484900
H	4.92528500	-2.48779500	-2.11551600
H	5.42081600	-1.04300700	-1.18734000
H	3.93513900	-1.00599800	-2.17932600
C	1.90312400	3.69901400	0.53305000
H	1.24458600	4.07873300	1.32694400
H	2.94614800	3.79453000	0.86939500
H	1.76712100	4.33568900	-0.35212000

C	2.46964500	1.79992400	-0.98070700
H	2.26895200	0.76427200	-1.28302100
H	2.26985300	2.45541500	-1.84196600
H	3.53861100	1.89270300	-0.73363500
N	-0.64938800	3.12307200	-0.34473100
C	-1.91874600	2.96866900	-0.74984100
N	-2.52795800	1.82995000	-1.05115800
C	-1.73740100	0.77200600	-0.91138400
C	-0.39186200	0.79772700	-0.50211100
C	0.14803400	2.06457800	-0.21051500
N	-2.03153200	-0.54654200	-1.13637400
C	-0.88825300	-1.24091000	-0.85833200
N	0.11241700	-0.48650000	-0.48015800
C	-4.30559700	-1.09534800	-0.36725100
C	-5.28543000	-0.10467100	-0.25494800
C	-6.16417800	-0.10231200	0.82944700
C	-6.06598500	-1.08865400	1.81076900
C	-5.08713900	-2.07926000	1.70558700
C	-4.21155500	-2.08215900	0.62094400
C	-3.33113200	-1.08524200	-1.52345200
H	-2.51262000	3.88255100	-0.84114500
H	-0.85874900	-2.32651000	-0.96161700
H	-5.35357000	0.67139900	-1.02056500
H	-6.92762700	0.67387700	0.90724400
H	-6.75394800	-1.08728600	2.65822100
H	-5.00971000	-2.85500600	2.46954000
H	-3.44943300	-2.86129100	0.53558000
H	-3.15552000	-2.10193800	-1.89672800
H	-3.71759000	-0.47203400	-2.34788600

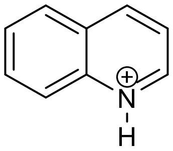

quinolin-1-H+

Zero-point correction= 0.150691 (Hartree/Particle)

Thermal correction to Energy= 0.157467

Thermal correction to Enthalpy= 0.158411

Thermal correction to Gibbs Free Energy= 0.119448

Sum of electronic and zero-point Energies= -401.777673

Sum of electronic and thermal Energies= -401.770897

Sum of electronic and thermal Enthalpies= -401.769953

Sum of electronic and thermal Free Energies= -401.808916

C	-0.01861300	0.74533900	0.00000000
C	-0.01537800	-0.67263200	0.00000000
N	1.19716900	-1.30884600	0.00000000
C	2.36218400	-0.68110400	0.00000000
C	2.40973500	0.71838800	0.00000000
C	1.22602400	1.42378800	0.00000000
C	-1.26923000	1.41894200	0.00000000
C	-2.43784600	0.69737500	0.00000000
C	-2.40986400	-0.72067400	0.00000000
C	-1.21902100	-1.40928600	0.00000000
H	3.25485800	-1.30685800	0.00000000
H	3.37785000	1.21584500	0.00000000
H	1.23000900	2.51580100	0.00000000
H	-1.27507000	2.51005700	0.00000000
H	-3.39850600	1.21337800	0.00000000
H	-3.34976100	-1.27437100	0.00000000
H	-1.18260000	-2.49990000	0.00000000
H	1.19509100	-2.33284000	0.00000000

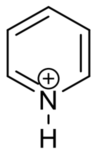

pyridin-1-H^{+}

Zero-point correction= 0.103480 (Hartree/Particle)

Thermal correction to Energy= 0.107831

Thermal correction to Enthalpy= 0.108775

Thermal correction to Gibbs Free Energy= 0.076000

Sum of electronic and zero-point Energies= -248.349381

Sum of electronic and thermal Energies= -248.345030

Sum of electronic and thermal Enthalpies= -248.344086

Sum of electronic and thermal Free Energies= -248.376861

C	-0.71550400	1.20849500	0.00000000
C	0.66767100	1.18239200	0.00000000
N	1.30103800	-0.00000300	0.00000100
C	0.66766700	-1.18239400	0.00000000
C	-0.71550900	-1.20849200	-0.00000100
C	-1.41271700	0.00000300	-0.00000100
H	-1.23334900	2.16650700	0.00000000
H	1.29433900	2.07345200	0.00000100
H	1.29433000	-2.07345700	0.00000000
H	-1.23335700	-2.16650200	-0.00000100
H	-2.50372800	0.00000500	-0.00000100
H	2.32484600	-0.00000500	0.00000100

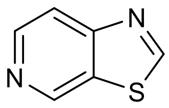

thiazolo[5,4-*c*]pyridine

Zero-point correction= 0.092362 (Hartree/Particle)

Thermal correction to Energy= 0.098402

Thermal correction to Enthalpy= 0.099347

Thermal correction to Gibbs Free Energy= 0.061580

Sum of electronic and zero-point Energies= -738.123909

Sum of electronic and thermal Energies= -738.117869

Sum of electronic and thermal Enthalpies= -738.116924

Sum of electronic and thermal Free Energies= -738.154691

C	-2.44734900	0.41266400	0.00013400
C	-1.41318300	1.33604000	0.00013000
C	-0.10435100	0.83541300	0.00003100
C	0.07534400	-0.56207100	-0.00004100
C	-1.03926100	-1.41020400	-0.00002900
N	1.06381900	1.57363000	-0.00003600
C	2.08802700	0.79233000	-0.00029800
S	1.77258400	-0.92557700	0.00001600
H	-3.48380400	0.76064200	0.00020400
H	-1.60308500	2.40949200	0.00018600
H	-0.91929500	-2.49769800	-0.00004000
H	3.12384500	1.14257400	-0.00030700
N	-2.26872800	-0.92088800	0.00005600

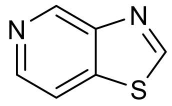

thiazolo[4,5-*c*]pyridine

Zero-point correction= 0.092328 (Hartree/Particle)

Thermal correction to Energy= 0.098371

Thermal correction to Enthalpy= 0.099315

Thermal correction to Gibbs Free Energy= 0.061545

Sum of electronic and zero-point Energies= -738.124257

Sum of electronic and thermal Energies= -738.118214

Sum of electronic and thermal Enthalpies= -738.117270

Sum of electronic and thermal Free Energies= -738.155041

C	1.40521300	1.32894800	-0.00012800
C	0.09720200	0.81885500	-0.00002700

C	-0.07391500	-0.57920400	0.00001800
C	1.04311700	-1.41904400	-0.00004700
N	-1.07020400	1.55843300	0.00002700
C	-2.09339500	0.77986000	0.00006000
S	-1.76935100	-0.94334000	0.00019500
H	1.56220800	2.41232700	-0.00017000
H	0.95822800	-2.50564800	-0.00001000
H	-3.13040500	1.12582800	0.00013200
C	2.28390000	-0.79614000	-0.00015700
H	3.19023900	-1.40803500	-0.00020700
N	2.46400500	0.53718300	-0.00019500

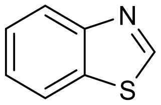

benzothiazole

Zero-point correction= 0.104063 (Hartree/Particle)

Thermal correction to Energy= 0.110230

Thermal correction to Enthalpy= 0.111174

Thermal correction to Gibbs Free Energy= 0.073238

Sum of electronic and zero-point Energies= -722.093818

Sum of electronic and thermal Energies= -722.087651

Sum of electronic and thermal Enthalpies= -722.086706

Sum of electronic and thermal Free Energies= -722.124643

C	2.28446100	-0.88520600	0.00013400
C	2.46920900	0.51065400	-0.00003100
C	1.38411300	1.37339400	-0.00012600
C	0.08849400	0.83199600	-0.00006100
C	-0.08242500	-0.56970800	0.00008100
C	1.01238500	-1.44166200	0.00019100
N	-1.08846600	1.56242700	0.00009900
C	-2.11054500	0.78138200	0.00048800

S	-1.78300800	-0.93605000	-0.00034500
H	3.15524400	-1.54280200	0.00016100
H	3.48196000	0.91653000	-0.00007900
H	1.51228000	2.45677800	-0.00019500
H	0.87126900	-2.52329600	0.00021000
H	-3.14752100	1.12748900	0.00066400

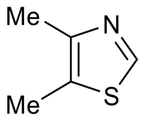

thiazole

Zero-point correction= 0.111305 (Hartree/Particle)

Thermal correction to Energy= 0.118531

Thermal correction to Enthalpy= 0.119476

Thermal correction to Gibbs Free Energy= 0.079652

Sum of electronic and zero-point Energies= -647.141984

Sum of electronic and thermal Energies= -647.134758

Sum of electronic and thermal Enthalpies= -647.133814

Sum of electronic and thermal Free Energies= -647.173638

C	1.26886200	-1.27305700	0.00093900
N	0.02225100	-1.61634000	0.00075000
C	-0.81102000	-0.51479400	-0.00122600
C	-0.16810900	0.69858100	-0.00106100
S	1.54704800	0.43170700	-0.00041100
H	2.10515500	-1.97601800	0.00089000
C	-0.72637900	2.08526700	0.00081300
H	-1.82278200	2.05639400	0.01596500
H	-0.38998800	2.65001400	0.88276100
H	-0.41270800	2.64488300	-0.89282700
C	-2.29053700	-0.74659300	-0.00037900
H	-2.59945300	-1.25586500	0.92496000

H	-2.85490400	0.18931400	-0.08391100
H	-2.57075300	-1.39807900	-0.84102400

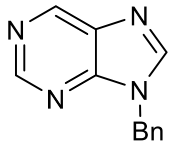

9-benzyl-9*H*-purine

Zero-point correction= 0.207481 (Hartree/Particle)

Thermal correction to Energy= 0.218922

Thermal correction to Enthalpy= 0.219866

Thermal correction to Gibbs Free Energy= 0.167737

Sum of electronic and zero-point Energies= -681.353328

Sum of electronic and thermal Energies= -681.341887

Sum of electronic and thermal Enthalpies= -681.340943

Sum of electronic and thermal Free Energies= -681.393072

N	3.45633500	-1.46479900	-0.54831900
C	2.47950500	-1.93349400	0.24107900
N	1.44334400	-1.25162100	0.71822000
C	1.43481500	0.01901600	0.33999500
C	2.40026000	0.63958200	-0.48301300
C	3.43621500	-0.18655000	-0.91907900
N	0.53235100	0.99802900	0.64653600
C	0.97600500	2.12953000	0.01241800
N	2.08043700	1.96560400	-0.66645500
C	-1.81700900	0.24798300	0.61973100
C	-2.67354000	1.08751200	-0.10052900
C	-3.69952900	0.54965100	-0.87722800
C	-3.87704200	-0.83342900	-0.93925500
C	-3.02643000	-1.67608000	-0.22237000
C	-2.00001000	-1.13760000	0.55398500
C	-0.68527400	0.83316600	1.43391800

H	2.54319000	-2.98896500	0.52014400
H	4.24127300	0.18181300	-1.56245500
H	0.41735600	3.06165400	0.10643200
H	-2.53837200	2.17070400	-0.04620300
H	-4.36568800	1.21259900	-1.43226500
H	-4.68176100	-1.25483300	-1.54444100
H	-3.16446900	-2.75796400	-0.26524900
H	-1.32929300	-1.79399400	1.11283200
H	-0.95655600	1.82266700	1.82168800
H	-0.44073800	0.18010700	2.28174100

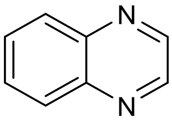

quinoxaline

Zero-point correction= 0.125385 (Hartree/Particle)

Thermal correction to Energy= 0.131831

Thermal correction to Enthalpy= 0.132775

Thermal correction to Gibbs Free Energy= 0.094376

Sum of electronic and zero-point Energies= -417.364447

Sum of electronic and thermal Energies= -417.358001

Sum of electronic and thermal Enthalpies= -417.357057

Sum of electronic and thermal Free Energies= -417.395456

C	-2.37704700	0.70978100	0.00000000
C	-1.19419000	1.40920000	0.00000000
C	0.04261400	0.71176200	0.00000100
C	0.04261400	-0.71176200	0.00000100
C	-1.19419000	-1.40920000	0.00000000
C	-2.37704700	-0.70978100	-0.00000100
H	-3.32744900	1.24578100	-0.00000100
H	-1.16900300	2.50000700	0.00000000

H	-1.16900300	-2.50000700	0.00000000
H	-3.32744900	-1.24578100	-0.00000100
C	2.31812400	-0.71367900	-0.00000200
C	2.31812400	0.71367900	-0.00000300
H	3.27064500	-1.25201400	0.00000200
H	3.27064500	1.25201400	0.00000300
N	1.21268500	-1.40916200	0.00000100
N	1.21268500	1.40916200	0.00000100

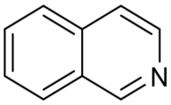

isoquinoline

Zero-point correction= 0.137269 (Hartree/Particle)

Thermal correction to Energy= 0.143889

Thermal correction to Enthalpy= 0.144833

Thermal correction to Gibbs Free Energy= 0.106132

Sum of electronic and zero-point Energies= -401.333259

Sum of electronic and thermal Energies= -401.326640

Sum of electronic and thermal Enthalpies= -401.325696

Sum of electronic and thermal Free Energies= -401.364397

C	-2.41167200	-0.70572300	-0.00002700
C	-1.22647800	-1.40071600	-0.00006800
C	0.00773400	-0.69685800	-0.00003700
C	0.01178600	0.72440300	0.00003900
C	-1.22969800	1.41844000	0.00008000
C	-2.41027000	0.71471200	0.00004700
H	1.27651600	-2.46004700	-0.00013500
H	-3.36231300	-1.24128000	-0.00005100
H	-1.21363300	-2.49297800	-0.00012500
C	1.26654000	-1.36332500	-0.00007800
C	1.27501000	1.37413400	0.00006800

H	-1.22659800	2.51032700	0.00013600
H	-3.36184400	1.24935200	0.00007900
C	2.41927700	0.61550500	0.00002300
H	1.32988500	2.46418600	0.00012600
H	3.40090200	1.09707600	0.00004400
N	2.42053100	-0.74429500	-0.00005100

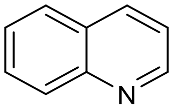

quinoline

Zero-point correction= 0.137206 (Hartree/Particle)

Thermal correction to Energy= 0.143799

Thermal correction to Enthalpy= 0.144744

Thermal correction to Gibbs Free Energy= 0.106113

Sum of electronic and zero-point Energies= -401.335028

Sum of electronic and thermal Energies= -401.328435

Sum of electronic and thermal Enthalpies= -401.327491

Sum of electronic and thermal Free Energies= -401.366121

C	2.39836800	-0.71525800	0.00000000
C	1.20974400	-1.40439600	0.00000000
C	-0.02871100	-0.70415900	0.00000000
C	-0.01552400	0.72064800	0.00000000
C	1.22936100	1.40733400	0.00000000
C	2.40989500	0.70413600	0.00000000
H	3.34425600	-1.25975400	0.00000000
H	1.17968800	-2.49526900	0.00000000
C	-1.26765800	1.39154800	0.00000000
H	1.22596900	2.49957200	0.00000000
H	3.36352400	1.23448500	0.00000000
C	-2.42206100	0.65165900	0.00000000
C	-2.32326500	-0.76537100	0.00000000

H	-1.29074000	2.48387400	0.00000000
H	-3.40422000	1.12500300	0.00000000
H	-3.24142900	-1.36279300	0.00000000
N	-1.18827700	-1.42028100	0.00000000

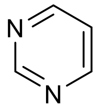

pyrimidine

Zero-point correction= 0.077941 (Hartree/Particle)

Thermal correction to Energy= 0.082095

Thermal correction to Enthalpy= 0.083040

Thermal correction to Gibbs Free Energy= 0.050603

Sum of electronic and zero-point Energies= -263.944314

Sum of electronic and thermal Energies= -263.940159

Sum of electronic and thermal Enthalpies= -263.939215

Sum of electronic and thermal Free Energies= -263.971652

C	-1.31460600	0.00000000	0.00000000
C	0.62079900	-1.18251000	0.00000000
C	1.35478400	0.00000000	0.00000000
C	0.62079900	1.18251000	0.00000000
H	-2.40939800	0.00000000	0.00000000
H	1.12333100	-2.15467500	0.00000000
H	2.44438800	0.00000000	0.00000000
H	1.12333200	2.15467500	0.00000000
N	-0.71230800	1.18623100	0.00000000
N	-0.71230800	-1.18623100	0.00000000

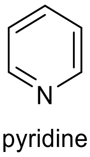

pyridine

Zero-point correction= 0.089654 (Hartree/Particle)

Thermal correction to Energy= 0.093898

Thermal correction to Enthalpy=	0.094842
Thermal correction to Gibbs Free Energy=	0.062268
Sum of electronic and zero-point Energies=	-247.906176
Sum of electronic and thermal Energies=	-247.901932
Sum of electronic and thermal Enthalpies=	-247.900988
Sum of electronic and thermal Free Energies=	-247.933562

C	0.00000000	0.72347200	1.14262900
C	0.00000000	-0.67063600	1.19797600
C	0.00000000	-1.38201100	0.00000000
C	0.00000000	-0.67063600	-1.19797600
C	0.00000000	0.72347200	-1.14262900
H	0.00000000	1.30741400	2.06857100
H	0.00000000	-1.18197500	2.16128400
H	0.00000000	-2.47376800	0.00000000
H	0.00000000	-1.18197500	-2.16128400
H	0.00000000	1.30741400	-2.06857100
N	0.00000000	1.41156000	0.00000000

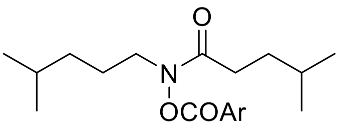

Ar = p-$CF_3C_6H_4$

1y

Zero-point correction=	0.460114 (Hartree/Particle)
Thermal correction to Energy=	0.488375
Thermal correction to Enthalpy=	0.489319
Thermal correction to Gibbs Free Energy=	0.397644
Sum of electronic and zero-point Energies=	-1356.936718
Sum of electronic and thermal Energies=	-1356.908457
Sum of electronic and thermal Enthalpies=	-1356.907513
Sum of electronic and thermal Free Energies=	-1356.999188

N	1.77029200	-0.20717200	0.98516000

C	2.20488000	-1.03534900	2.09797000
C	2.34618800	-2.52421800	1.79351600
C	3.53662500	-2.87731200	0.89589600
C	3.30533600	-2.74784700	-0.61582800
C	4.63949600	-2.77497300	-1.35667300
C	2.37881400	-3.84697600	-1.13038000
H	1.40240100	-2.90699200	1.37055400
H	2.46804900	-3.02326900	2.76748800
H	3.84655800	-3.91775200	1.09866500
H	4.38799400	-2.23957300	1.18751100
H	2.81700500	-1.78219200	-0.81323200
H	4.49470200	-2.73049000	-2.44723000
H	5.19455500	-3.70121200	-1.13122700
H	5.27186700	-1.92294500	-1.06434100
H	2.16550700	-3.71603100	-2.20243300
H	1.41390200	-3.84696400	-0.60338100
H	2.84324800	-4.83889900	-0.99796900
C	2.59218100	0.59474400	0.23640600
C	1.92093800	1.52598500	-0.75289400
H	1.62059600	0.90363100	-1.61279900
H	0.99143300	1.92779300	-0.32735300
C	2.84881400	2.64218700	-1.21632700
H	3.74370500	2.19496800	-1.67543600
H	2.33071900	3.20884100	-2.00826300
C	3.28984300	3.61868600	-0.11860200
H	3.90833100	3.05410700	0.59846900
C	2.10359700	4.21354600	0.63828000
H	2.44162800	4.97501300	1.35744700
H	1.39715900	4.69764300	-0.05695100
H	1.54952900	3.45058200	1.20594500

C	4.15347500	4.72143800	-0.72554200
H	5.01191800	4.30231600	-1.27252600
H	3.56711200	5.32802500	-1.43536000
H	4.54410500	5.39783400	0.04968800
O	3.79932900	0.50131700	0.36024400
H	3.16932000	-0.62436800	2.42594800
H	1.47490600	-0.87420500	2.90439500
O	0.40744400	-0.01915400	0.91082000
C	-0.22953200	-0.69777800	-0.08585900
O	0.33491600	-1.38531300	-0.88401100
C	-1.69755500	-0.44013400	-0.04823600
C	-2.48038200	-1.05470000	-1.02901800
C	-2.28592300	0.38011000	0.92231300
C	-3.85704700	-0.85286200	-1.04607200
H	-2.00400600	-1.69042700	-1.77605100
C	-3.66122900	0.58152900	0.90637400
H	-1.67440200	0.85708800	1.68734000
C	-4.43732000	-0.03552300	-0.07731100
H	-4.47326000	-1.33075700	-1.80779500
H	-4.12995900	1.21659700	1.66012500
C	-5.92024200	0.22463000	-0.08631200
F	-6.57531400	-0.59768400	-0.90562200
F	-6.19565800	1.47315100	-0.47948000
F	-6.45190900	0.08185900	1.13132600

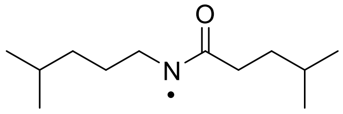

1y-radical

Zero-point correction= 0.344210 (Hartree/Particle)

Thermal correction to Energy= 0.361718

Thermal correction to Enthalpy= 0.362662

Thermal correction to Gibbs Free Energy= 0.296122

Sum of electronic and zero-point Energies= -600.583861

Sum of electronic and thermal Energies= -600.566353

Sum of electronic and thermal Enthalpies= -600.565409

Sum of electronic and thermal Free Energies= -600.631949

N	0.48790600	1.16239000	0.66544600
C	1.08829600	1.97318500	-0.35213500
C	2.60317000	1.73483100	-0.46287800
C	2.95765000	0.32870200	-0.95126700
C	2.98550200	-0.75063600	0.14048500
C	2.89527600	-2.14186500	-0.47952000
C	4.23305900	-0.62610200	1.01308200
H	3.07305800	1.94200500	0.51170500
H	2.98506700	2.48773000	-1.16842200
H	3.94873400	0.34767200	-1.43697600
H	2.23250600	0.03772800	-1.72920900
H	2.10576600	-0.60860900	0.79117100
H	2.94252500	-2.92851700	0.28912200
H	3.72847400	-2.30939100	-1.18231200
H	1.95284800	-2.26267200	-1.03436300
H	4.21929000	-1.35989100	1.83323900
H	4.32372100	0.37433500	1.46288300
H	5.14069800	-0.80815300	0.41419900
C	-0.43514900	0.20336300	0.26587400
C	-1.50020600	-0.09951800	1.29092500
H	-0.98975200	-0.66337100	2.09047000
H	-1.81412000	0.84737100	1.75667500
C	-2.67160300	-0.89735700	0.73536100
H	-2.28301500	-1.83220600	0.30189600
H	-3.32958500	-1.18338400	1.57310000

C	-3.51076200	-0.17139200	-0.32383000
H	-2.85019900	0.05840500	-1.17705200
C	-4.08992800	1.14130500	0.19999100
H	-4.73536900	1.61356400	-0.55605400
H	-4.70160500	0.96429400	1.10056600
H	-3.30577500	1.86717100	0.46181800
C	-4.62395500	-1.08974200	-0.82240500
H	-4.21907500	-2.03358900	-1.21838700
H	-5.31708800	-1.33982000	-0.00229100
H	-5.20940700	-0.61143300	-1.62202900
O	-0.30583200	-0.40126000	-0.78270400
H	0.61217700	1.82265400	-1.33537300
H	0.93333600	3.02281600	-0.04297400

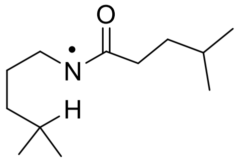

1y-iso1

Zero-point correction= 0.344300 (Hartree/Particle)

Thermal correction to Energy= 0.361795

Thermal correction to Enthalpy= 0.362739

Thermal correction to Gibbs Free Energy= 0.297077

Sum of electronic and zero-point Energies= -600.586151

Sum of electronic and thermal Energies= -600.568656

Sum of electronic and thermal Enthalpies= -600.567712

Sum of electronic and thermal Free Energies= -600.633375

N	-0.15591500	0.95072000	-0.46419100
C	-1.24290000	1.84748600	-0.73943400
C	-2.42402400	1.12701400	-1.39690600
C	-3.27168500	0.30495600	-0.42580900
C	-2.55194700	-0.80874200	0.34318100

C	-3.52699400	-1.50291500	1.29139200
C	-1.89332900	-1.82351000	-0.58870300
H	-2.04180500	0.50151300	-2.21862200
H	-3.06436700	1.89386900	-1.85702000
H	-4.10640700	-0.14812100	-0.98955000
H	-3.73143400	0.99240400	0.30490100
H	-1.76342300	-0.34494100	0.96441700
H	-3.01936500	-2.27174600	1.89331400
H	-4.33241500	-1.99791100	0.72439100
H	-3.99471200	-0.78535000	1.98263100
H	-1.40357800	-2.62680600	-0.01628300
H	-1.13254600	-1.35714800	-1.23095500
H	-2.64841400	-2.29211900	-1.24217100
C	0.56667700	1.12965500	0.70480100
C	1.12427400	-0.13018000	1.32303800
H	0.93535900	-0.07007200	2.40490000
H	0.59109900	-1.00646700	0.92771900
C	2.63355300	-0.24137000	1.07288900
H	3.12654100	0.62359200	1.54522600
H	3.00748600	-1.14204700	1.58824200
C	3.04070000	-0.30760700	-0.40286500
H	2.70825900	0.62588000	-0.89326100
C	2.38222400	-1.48031000	-1.12664100
H	2.74629700	-1.55485300	-2.16260100
H	2.61646300	-2.43066600	-0.61830600
H	1.28825900	-1.37499100	-1.16597900
C	4.56094500	-0.38274400	-0.51920500
H	5.04611300	0.46403400	-0.01030800
H	4.93822400	-1.31147900	-0.06060500
H	4.88136500	-0.37420800	-1.57178700

O	0.80859800	2.24386500	1.13032700
H	-1.56211200	2.40606000	0.15773400
H	-0.84722200	2.58520300	-1.46373800

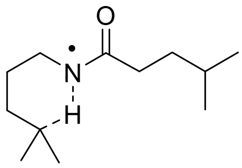

TS4

Zero-point correction=	0.340414 (Hartree/Particle)
Thermal correction to Energy=	0.356988
Thermal correction to Enthalpy=	0.357932
Thermal correction to Gibbs Free Energy=	0.295598
Sum of electronic and zero-point Energies=	-600.578355
Sum of electronic and thermal Energies=	-600.561781
Sum of electronic and thermal Enthalpies=	-600.560836
Sum of electronic and thermal Free Energies=	-600.623170

N	0.39781000	0.78329600	0.41566900
C	1.37537300	1.70632100	0.94785200
C	2.61848400	0.94683500	1.41342200
C	3.22866100	0.15252800	0.25629700
C	2.25400800	-0.88586900	-0.28785400
C	2.38007500	-1.14348700	-1.77472600
C	2.16615500	-2.14942400	0.54719900
H	2.34379200	0.26787600	2.23653300
H	3.34415100	1.66794000	1.81516300
H	4.15684200	-0.34593500	0.58620300
H	3.50721400	0.85353700	-0.54779000
H	1.20455400	-0.32735900	-0.10186800
H	1.63232300	-1.86960600	-2.12684200
H	3.37879400	-1.55652100	-2.00142500
H	2.26571300	-0.21462900	-2.35403900

H	1.34588000	-2.79942000	0.20749100
H	2.00783000	-1.92433300	1.61235200
H	3.10524700	-2.72287700	0.45918800
C	-0.43055300	1.19771200	-0.59821900
C	-1.11988000	0.09813400	-1.37850300
H	-1.03008400	0.34793200	-2.44598900
H	-0.62030700	-0.86627400	-1.21079400
C	-2.60285400	0.01813700	-0.99997100
H	-3.07874200	0.97729300	-1.26071800
H	-3.08522000	-0.75610500	-1.62070000
C	-2.87510400	-0.28964500	0.47681800
H	-2.45637200	0.53585700	1.08113300
C	-2.20552700	-1.58650500	0.92751300
H	-2.48806900	-1.83441700	1.96204000
H	-2.51182200	-2.42818100	0.28374900
H	-1.10851200	-1.51109600	0.89434000
C	-4.38004700	-0.33850600	0.72871300
H	-4.87058600	0.59422200	0.41098000
H	-4.83986900	-1.16807000	0.16684500
H	-4.60246700	-0.49289500	1.79529300
O	-0.67614800	2.37878200	-0.78950400
H	1.64555300	2.47441800	0.20287200
H	0.92591400	2.22792500	1.81108300

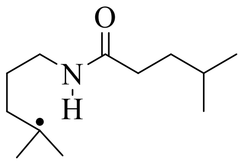

1y-iso1-HAT

Zero-point correction= 0.344332 (Hartree/Particle)

Thermal correction to Energy= 0.362291

Thermal correction to Enthalpy= 0.363235

Thermal correction to Gibbs Free Energy= 0.296018

Sum of electronic and zero-point Energies= -600.610645

Sum of electronic and thermal Energies= -600.592686

Sum of electronic and thermal Enthalpies= -600.591742

Sum of electronic and thermal Free Energies= -600.658959

N	0.28108900	0.70790200	-0.02139200
C	1.13770300	1.80051400	-0.43501300
C	2.45573100	1.82409100	0.33174300
C	3.42222900	0.67755300	-0.01280000
H	2.24194500	1.82152900	1.41505600
H	2.95424900	2.78290300	0.12073700
H	4.37176500	0.88688600	0.51689200
H	3.65201300	0.72457600	-1.09048200
H	0.17780700	0.54643800	0.97527000
C	-0.34224300	-0.13633900	-0.87596600
C	-1.11219600	-1.26565900	-0.21042000
H	-1.58732900	-1.83496500	-1.01944500
H	-0.38079600	-1.93331600	0.27497700
C	-2.14673100	-0.80310700	0.81970100
H	-2.72773200	-1.68408800	1.14200100
H	-1.63939500	-0.43176200	1.72625600
C	-3.11495000	0.27283800	0.31488800
H	-2.52612200	1.18619500	0.11395200
C	-4.14107800	0.60415300	1.39503800
H	-4.80367900	1.42445600	1.08046000
H	-4.77232600	-0.27309600	1.61199600
H	-3.65183700	0.90540300	2.33398700
C	-3.79906900	-0.13956000	-0.98724100
H	-3.07539500	-0.24551000	-1.80888300
H	-4.32378100	-1.10212900	-0.86573800

H	-4.54267200	0.61200000	-1.29307400
O	-0.28180400	-0.01707500	-2.09255200
H	1.31385500	1.68018900	-1.51314600
H	0.61576900	2.76209600	-0.29401200
C	2.95400900	-0.70273500	0.34072900
C	2.63040700	-1.02591700	1.76225000
H	3.20615900	-0.39831900	2.46176700
H	1.55951000	-0.86965200	2.00872000
H	2.84669500	-2.08119700	1.99616900
C	2.65988600	-1.68204200	-0.74845200
H	3.54435500	-1.85320100	-1.38969200
H	2.34803100	-2.65880600	-0.34943300
H	1.86352600	-1.32808300	-1.43091200

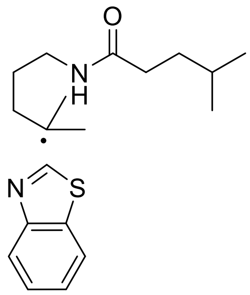

Complex 1-1

Zero-point correction= 0.449266 (Hartree/Particle)

Thermal correction to Energy= 0.475292

Thermal correction to Enthalpy= 0.476236

Thermal correction to Gibbs Free Energy= 0.387518

Sum of electronic and zero-point Energies= -1322.714369

Sum of electronic and thermal Energies= -1322.688343

Sum of electronic and thermal Enthalpies= -1322.687399

Sum of electronic and thermal Free Energies= -1322.776117

N	-2.64444300	0.96438500	-0.31329700
C	-2.25250000	2.06727100	-1.16682100
C	-1.16259800	2.92846900	-0.53781700

C	0.21554600	2.25304400	-0.43853100
H	-1.49378500	3.25254100	0.46443300
H	-1.06489900	3.84582600	-1.13905800
H	0.92659500	3.02328600	-0.07795000
H	0.54911300	1.97827000	-1.45353100
H	-2.80997300	1.16559900	0.66791100
C	-2.75026900	-0.31860500	-0.73083100
C	-3.10471400	-1.31572900	0.36009900
H	-3.14031900	-2.30254400	-0.11856200
H	-2.28130200	-1.33284000	1.09314800
C	-4.42396500	-1.00194600	1.07328800
H	-4.65972100	-1.84194900	1.74891700
H	-4.30066900	-0.11807500	1.72177100
C	-5.61237900	-0.76817800	0.13358300
H	-5.40582400	0.15243400	-0.44243100
C	-6.88774600	-0.54155100	0.94049600
H	-7.73587800	-0.29185800	0.28534400
H	-7.15592300	-1.45003100	1.50441300
H	-6.76563500	0.27919500	1.66386300
C	-5.78796700	-1.91398200	-0.86100800
H	-4.93068100	-1.98847500	-1.54583300
H	-5.89230300	-2.87691500	-0.33339700
H	-6.69155900	-1.76598200	-1.47167900
O	-2.57052400	-0.65750000	-1.89320600
H	-1.90837300	1.63135000	-2.11520400
H	-3.12967100	2.69634400	-1.39386100
C	0.29474800	1.05218700	0.45572700
C	-0.06315100	1.17679700	1.89999900
H	0.00712100	2.21915400	2.25046300
H	-1.09678900	0.83730800	2.11675800

H	0.60202700	0.55972400	2.52819800
C	0.57627600	-0.29176900	-0.13096400
H	1.49261300	-0.27586800	-0.75077500
H	0.71082300	-1.05901700	0.64711100
H	-0.23040100	-0.63797800	-0.80589200
C	3.21205400	1.31877400	1.21010500
N	3.32515200	0.14147800	1.71732000
C	3.94260200	-0.72042600	0.82627100
C	4.31481500	-0.13692600	-0.40508900
S	3.85305300	1.54165700	-0.40185100
C	4.20177000	-2.08069900	1.05577700
C	4.82068700	-2.82144500	0.05997400
C	5.18714200	-2.22737900	-1.16220500
C	4.93980400	-0.88305100	-1.40996500
H	2.76216300	2.16966800	1.72836700
H	3.91051300	-2.52847700	2.00699600
H	5.02699900	-3.88018000	0.22414200
H	5.67279500	-2.83165900	-1.93003700
H	5.22239000	-0.42553600	-2.35905000

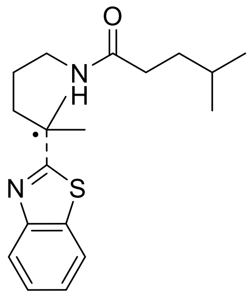

TS6

Zero-point correction= 0.450327 (Hartree/Particle)

Thermal correction to Energy= 0.474751

Thermal correction to Enthalpy= 0.475696

Thermal correction to Gibbs Free Energy= 0.393681

Sum of electronic and zero-point Energies= -1322.703607

Sum of electronic and thermal Energies= -1322.679183

Sum of electronic and thermal Enthalpies= -1322.678238

Sum of electronic and thermal Free Energies= -1322.760253

N	-2.50504600	0.78867400	-0.13648100
C	-1.99389300	1.73093200	-1.11283500
C	-1.00292800	2.72226100	-0.50729900
C	0.45428400	2.24247300	-0.38992500
H	-1.37634700	3.04845100	0.47827100
H	-0.99595400	3.62215400	-1.14027500
H	1.03703100	3.10006500	-0.00647200
H	0.83627300	2.02436800	-1.40043900
H	-2.80425100	1.16129300	0.75986800
C	-2.76983600	-0.51128300	-0.40989300
C	-3.40646800	-1.28716900	0.72952400
H	-3.45092600	-2.33611600	0.41072600
H	-2.74810700	-1.23256100	1.61101900
C	-4.79981200	-0.76217900	1.09444700
H	-5.24868000	-1.45124300	1.83003800
H	-4.70742100	0.21127400	1.60464500
C	-5.75456000	-0.60988700	-0.09506200
H	-5.34669900	0.17977200	-0.75296600
C	-7.12735400	-0.15203900	0.38975100
H	-7.80890800	0.03352400	-0.45392600
H	-7.58720400	-0.92119200	1.03159300
H	-7.05636300	0.77636900	0.97700200
C	-5.85976700	-1.89522000	-0.91323600
H	-4.89877400	-2.15320700	-1.38122600
H	-6.17257900	-2.73921900	-0.27576800
H	-6.60527900	-1.78778200	-1.71593100
O	-2.51617500	-1.02332600	-1.49227200

H	-1.52736900	1.14676200	-1.91901300
H	-2.83340600	2.28745000	-1.56284000
C	0.69724000	1.05020000	0.49963200
C	0.18515200	1.12767600	1.90644500
H	0.28491700	2.14277000	2.32157900
H	-0.88430200	0.85591500	1.94295900
H	0.72760700	0.42345400	2.55465800
C	0.71596200	-0.29971400	-0.14661700
H	1.45596900	-0.33528500	-0.96341600
H	0.95208200	-1.09123900	0.58015200
H	-0.25996300	-0.54012600	-0.60386900
C	2.83294400	1.29223800	0.96644600
N	3.16671100	0.18474800	1.59774500
C	3.90456000	-0.64415500	0.79496000
C	4.27089800	-0.10768100	-0.46732800
S	3.63245700	1.50687600	-0.61352200
C	4.31892000	-1.94788500	1.12839100
C	5.07145300	-2.67432100	0.21683000
C	5.42342900	-2.12812600	-1.03042600
C	5.02517900	-0.84196400	-1.38470900
H	2.54038000	2.19942800	1.50281400
H	4.03794500	-2.36334300	2.09741600
H	5.39370900	-3.68594600	0.46936300
H	6.01594800	-2.71828300	-1.73125200
H	5.29666900	-0.41981500	-2.35361500

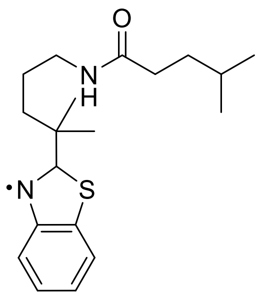

C-1

Zero-point correction= 0.453177 (Hartree/Particle)

Thermal correction to Energy= 0.477296

Thermal correction to Enthalpy= 0.478240

Thermal correction to Gibbs Free Energy= 0.397637

Sum of electronic and zero-point Energies= -1322.733699

Sum of electronic and thermal Energies= -1322.709580

Sum of electronic and thermal Enthalpies= -1322.708636

Sum of electronic and thermal Free Energies= -1322.789239

N	-2.61425900	0.69807900	0.07161200
C	-2.03633400	1.74021400	-0.75535200
C	-1.00723400	2.59611900	-0.01153300
C	0.46533900	2.17602700	-0.08507400
H	-1.32702900	2.71001700	1.03765400
H	-1.05619300	3.60685100	-0.44311900
H	1.04571600	2.99723800	0.37182300
H	0.76102000	2.14418900	-1.14780700
H	-2.90381700	0.95213500	1.01133200
C	-2.95621800	-0.52808300	-0.39238800
C	-3.67027300	-1.41303000	0.61363300
H	-3.75341000	-2.40790600	0.15851800
H	-3.04740100	-1.50710300	1.51694800
C	-5.05002500	-0.86433900	0.99539300
H	-5.56028900	-1.61312100	1.62506000
H	-4.93021100	0.03387600	1.62411300

C	-5.94954300	-0.51790300	-0.19634100
H	-5.47277000	0.31540000	-0.74538900
C	-7.31169900	-0.03694600	0.29662800
H	-7.94770400	0.28966100	-0.53982100
H	-7.84032600	-0.84821600	0.82343900
H	-7.21062200	0.80691700	0.99622000
C	-6.09821300	-1.69230000	-1.16141300
H	-5.13777100	-1.95091700	-1.63010800
H	-6.47714600	-2.58398100	-0.63429900
H	-6.80894500	-1.45038000	-1.96631800
O	-2.71402600	-0.89235400	-1.53498000
H	-1.59077900	1.25273300	-1.63382600
H	-2.84222300	2.39419200	-1.12880300
C	0.91304700	0.85945000	0.58597400
C	0.27113700	0.69780400	1.96736400
H	0.41344000	1.60338100	2.57834000
H	-0.80708500	0.51301400	1.86808900
H	0.71919100	-0.15177600	2.50078800
C	0.62170800	-0.36038100	-0.28953300
H	1.18818600	-0.30824500	-1.23289500
H	0.90743500	-1.28668200	0.23192800
H	-0.44293100	-0.43229600	-0.54465100
C	2.44883700	0.95688600	0.82482700
N	3.01067200	-0.23309500	1.35936800
C	4.03748300	-0.69680400	0.66480300
C	4.42788100	0.04518700	-0.50888300
S	3.40085800	1.42100900	-0.73060100
C	4.77125700	-1.87443000	1.00128400
C	5.82567600	-2.26912100	0.20684300
C	6.19029700	-1.52432900	-0.93765600

C	5.49732700	-0.37004700	-1.29973600
H	2.61969900	1.79231100	1.52965300
H	4.47650400	-2.43655300	1.88849600
H	6.39007900	-3.16766300	0.46101900
H	7.02910800	-1.85828800	-1.55020200
H	5.78715800	0.19516300	-2.18663800

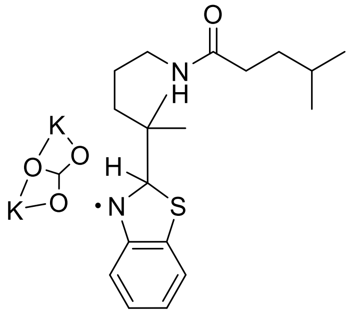

Complex 2-1

Zero-point correction= 0.471440 (Hartree/Particle)

Thermal correction to Energy= 0.503983

Thermal correction to Enthalpy= 0.504927

Thermal correction to Gibbs Free Energy= 0.403281

Sum of electronic and zero-point Energies= -2786.083776

Sum of electronic and thermal Energies= -2786.051233

Sum of electronic and thermal Enthalpies= -2786.050289

Sum of electronic and thermal Free Energies= -2786.151935

N	-4.03080200	0.55887700	0.09391400
C	-3.33275300	1.51283300	-0.74722200
C	-2.09343100	2.11195300	-0.07552700
C	-0.74542000	1.42097500	-0.30750100
H	-2.28975400	2.22361800	1.00385900
H	-1.98105600	3.13703400	-0.45919900
H	0.03089500	2.07114600	0.13481200
H	-0.55440200	1.40214900	-1.39563300
H	-4.17381100	0.80917400	1.06775500
C	-4.64614800	-0.55161100	-0.37844600

C	-5.42346900	-1.34192300	0.65917700
H	-5.72832200	-2.28015300	0.17887900
H	-4.75466700	-1.59464300	1.49681100
C	-6.64042100	-0.57326900	1.18767700
H	-7.22788700	-1.25124900	1.83018300
H	-6.30380900	0.25039300	1.83945200
C	-7.55509000	-0.00077900	0.09920600
H	-6.97910400	0.76393100	-0.45440500
C	-8.75840200	0.68955900	0.73553200
H	-9.39032600	1.17345300	-0.02426300
H	-9.38213100	-0.04157900	1.27547000
H	-8.44433600	1.45973700	1.45656800
C	-7.99824100	-1.06929900	-0.89836700
H	-7.14330100	-1.47325600	-1.45935200
H	-8.49856100	-1.90403300	-0.37921200
H	-8.71094500	-0.65283700	-1.62660500
O	-4.58583800	-0.88996400	-1.55284800
H	-3.07063700	0.99555900	-1.68098700
H	-4.02372000	2.32890300	-1.01801000
C	-0.51165900	-0.00010000	0.25207000
C	-1.04096600	-0.12204300	1.68489200
H	-0.66402200	0.69792500	2.31664300
H	-2.13903200	-0.09057600	1.69213700
H	-0.71971400	-1.07334000	2.13152200
C	-1.11517900	-1.08792900	-0.63762300
H	-0.66838900	-1.06022000	-1.64391200
H	-0.92208400	-2.08320900	-0.20770900
H	-2.20053100	-0.97290100	-0.75065800
C	1.01807700	-0.19391900	0.33787100
N	1.44489900	-1.46385000	0.78623600

C	2.50967700	-1.91926000	0.13928600
C	2.95207900	-1.16555700	-1.00805200
S	1.89645400	0.17343400	-1.29281400
C	3.24622000	-3.08109100	0.51471900
C	4.36135200	-3.45048900	-0.21473500
C	4.77657400	-2.69640300	-1.33582300
C	4.07557300	-1.55660200	-1.73730600
H	1.44802200	0.57018900	1.02379300
H	2.91719000	-3.64896900	1.38610500
H	4.93228500	-4.33321800	0.07735500
H	5.65903500	-3.00762800	-1.89678400
H	4.40525500	-0.98153400	-2.60397300
C	3.53766600	1.63997500	1.22073100
O	3.44867300	0.67838800	2.05921400
O	2.59135000	2.49366000	1.08376600
O	4.59663000	1.72769100	0.45653200
K	5.38323700	-0.58767200	1.09596600
K	3.36587700	3.14860300	-1.20666900

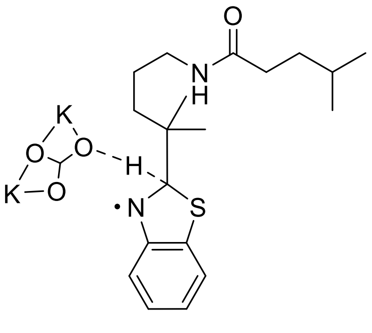

TS8

Zero-point correction= 0.466909 (Hartree/Particle)

Thermal correction to Energy= 0.499050

Thermal correction to Enthalpy= 0.499994

Thermal correction to Gibbs Free Energy= 0.399789

Sum of electronic and zero-point Energies= -2786.084017

Sum of electronic and thermal Energies= -2786.051875

Sum of electronic and thermal Enthalpies= -2786.050931

Sum of electronic and thermal Free Energies= -2786.151136

N	-3.97666600	0.46212800	0.03113500
C	-3.25553900	1.48709300	-0.69963800
C	-1.99677800	1.96825200	0.02768300
C	-0.66866900	1.27071600	-0.28634100
H	-2.18782000	1.96365700	1.11423500
H	-1.85690000	3.02730800	-0.23698100
H	0.12925900	1.85084000	0.20968100
H	-0.48580100	1.35974500	-1.37337600
H	-4.08268400	0.58647500	1.03327800
C	-4.65004800	-0.54985900	-0.56595800
C	-5.41779100	-1.45621200	0.38060200
H	-5.82473000	-2.27288000	-0.22894600
H	-4.71148800	-1.89732300	1.10168700
C	-6.53286000	-0.72381400	1.13520600
H	-7.12381600	-1.47181900	1.69094900
H	-6.09262300	-0.05763400	1.89608500
C	-7.47397400	0.09480200	0.24442800
H	-6.88372800	0.91901400	-0.19727000
C	-8.58668000	0.71432100	1.08536600
H	-9.23033000	1.36842400	0.47811600
H	-9.22390100	-0.07134400	1.52334300
H	-8.17814400	1.31423800	1.91295600
C	-8.04787400	-0.73809200	-0.90034100
H	-7.25901000	-1.06614500	-1.59265800
H	-8.56194100	-1.63322200	-0.51178600
H	-8.78068200	-0.15551200	-1.47922200
O	-4.63881600	-0.72422700	-1.77698200
H	-3.01025000	1.07324500	-1.68810500

H	-3.92436500	2.34748600	-0.87201700
C	-0.47221700	-0.20788500	0.11595700
C	-1.00660900	-0.47104500	1.52834000
H	-0.60077000	0.26009100	2.24539600
H	-2.10328200	-0.40645600	1.54615200
H	-0.71537900	-1.47607400	1.86510100
C	-1.11705300	-1.17185000	-0.88625000
H	-0.66795700	-1.04342800	-1.88404800
H	-0.94684500	-2.21341900	-0.57246700
H	-2.19959400	-1.01385300	-0.98002200
C	1.03972300	-0.47702200	0.16495400
N	1.46691100	-1.74894900	0.48077000
C	2.67168200	-2.03154600	-0.03926400
C	3.17563300	-1.11806700	-1.02622900
S	1.99730500	0.12217700	-1.34322600
C	3.49442000	-3.12251400	0.34631500
C	4.75976000	-3.26364700	-0.20911300
C	5.24255700	-2.34484200	-1.16017600
C	4.44703000	-1.27245300	-1.57802600
H	1.61730100	0.22355000	1.04839300
H	3.12249200	-3.82299400	1.09589800
H	5.39469000	-4.09446300	0.10404100
H	6.24213600	-2.46989600	-1.57855500
H	4.82278800	-0.56195400	-2.31736400
C	3.04881600	1.82923200	1.21078400
O	2.61899300	0.76569900	1.85708400
O	2.23841900	2.72906300	0.86079700
O	4.29868700	1.88056800	0.90894200
K	4.85685300	-0.50522100	1.59090000
K	3.61027000	3.07285200	-1.25262300

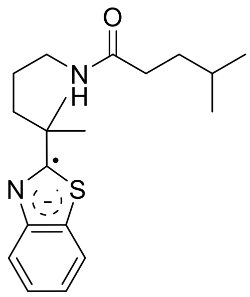

D-1

Zero-point correction=	0.439800 (Hartree/Particle)		
Thermal correction to Energy=	0.464063		
Thermal correction to Enthalpy=	0.465007		
Thermal correction to Gibbs Free Energy=	0.383710		
Sum of electronic and zero-point Energies=	-1322.248066		
Sum of electronic and thermal Energies=	-1322.223804		
Sum of electronic and thermal Enthalpies=	-1322.222860		
Sum of electronic and thermal Free Energies=	-1322.304157		
N	2.70771200	0.53370700	-0.19474400
C	2.09682000	1.77535100	0.24061500
C	1.02472800	2.28098000	-0.72927900
C	-0.43345200	1.89521200	-0.45605700
H	1.31167700	1.99984900	-1.75687000
H	1.06159400	3.38052300	-0.70862800
H	-1.05119000	2.46460000	-1.17152100
H	-0.70705400	2.26924200	0.54701000
H	2.88773700	0.41974200	-1.18737600
C	3.19583700	-0.39546300	0.66068200
C	3.89935500	-1.56859800	0.00042400
H	4.13832900	-2.28226400	0.79887000
H	3.20022900	-2.06226300	-0.69277800
C	5.16374900	-1.14807400	-0.75791300
H	5.69120600	-2.06027900	-1.08548300
H	4.88405200	-0.61115600	-1.67991000

C	6.13240800	-0.27558400	0.04840600
H	5.62755200	0.68721100	0.25129200
C	7.38370900	0.01679300	-0.77495500
H	8.05695200	0.70882900	-0.24711300
H	7.94353200	-0.91237300	-0.97111200
H	7.12950500	0.46647600	-1.74699000
C	6.49368900	-0.90860200	1.39085500
H	5.61494000	-0.98695000	2.04722300
H	6.90975800	-1.91985300	1.24645500
H	7.25153500	-0.30617600	1.91473300
O	3.08378100	-0.29458400	1.87477000
H	1.67968400	1.60467300	1.24343300
H	2.87949500	2.54542900	0.35095000
C	-0.87347100	0.41843100	-0.56190900
C	-0.29388600	-0.23336800	-1.82205600
H	-0.51511000	0.37382500	-2.71484100
H	0.79650900	-0.35184400	-1.74096800
H	-0.74210700	-1.22570600	-1.97032000
C	-0.44750100	-0.38697300	0.67952500
H	-0.88960300	0.05479800	1.58718300
H	-0.82452600	-1.41862000	0.59309900
H	0.64229600	-0.43054700	0.81513500
C	-2.39294500	0.37222900	-0.67054400
N	-3.03755200	-0.79467700	-0.91599500
C	-4.27773200	-0.84124600	-0.41688900
C	-4.68486000	0.27906200	0.39579500
S	-3.35562200	1.42267700	0.48446000
C	-5.24055300	-1.87555600	-0.61504800
C	-6.50038700	-1.78222400	-0.02815700
C	-6.86899200	-0.68681500	0.76328100

C	-5.94030700	0.35664000	0.97471800
H	-4.97101600	-2.73571800	-1.23188700
H	-7.22111000	-2.58755500	-0.19479200
H	-7.86228800	-0.63432600	1.21135500
H	-6.21123700	1.21789100	1.59054200

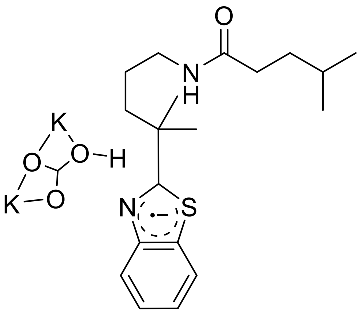

Complex 3-1

Zero-point correction=	0.470508 (Hartree/Particle)
Thermal correction to Energy=	0.503705
Thermal correction to Enthalpy=	0.504649
Thermal correction to Gibbs Free Energy=	0.398852
Sum of electronic and zero-point Energies=	-2786.097179
Sum of electronic and thermal Energies=	-2786.063982
Sum of electronic and thermal Enthalpies=	-2786.063038
Sum of electronic and thermal Free Energies=	-2786.168836

N	-4.00662900	0.42135600	0.06758600
C	-3.29887300	1.33570400	-0.80875200
C	-2.03699300	1.91769800	-0.16306100
C	-0.69890100	1.21812400	-0.42584500
H	-2.20926300	2.02230100	0.92141800
H	-1.92375400	2.94559000	-0.53954400
H	0.08629000	1.84315400	0.03207900
H	-0.51825400	1.22352700	-1.51704100
H	-4.09369200	0.68359900	1.04449300
C	-4.70022000	-0.65600300	-0.37155200
C	-5.48427500	-1.38937000	0.70246100

H	-5.84534100	-2.32259500	0.25175400
H	-4.80863200	-1.65408800	1.53064100
C	-6.65001100	-0.54927100	1.23931000
H	-7.25280000	-1.18018700	1.91428800
H	-6.25681000	0.27422600	1.85891300
C	-7.56405100	0.03687000	0.15793600
H	-6.96317500	0.74598800	-0.44159400
C	-8.70616200	0.81835700	0.80201900
H	-9.33912000	1.30122700	0.04287600
H	-9.34674000	0.14674300	1.39691600
H	-8.32638700	1.60167900	1.47558600
C	-8.09633500	-1.04001200	-0.78532300
H	-7.28217500	-1.51074900	-1.35456800
H	-8.62596200	-1.82596900	-0.22032800
H	-8.80719200	-0.61060400	-1.50826800
O	-4.70670100	-1.00351300	-1.54443100
H	-3.06170800	0.79066500	-1.73343200
H	-3.97422700	2.16128400	-1.09177800
C	-0.46682200	-0.21798100	0.09681200
C	-0.94422700	-0.35290300	1.54707400
H	-0.51551900	0.44492700	2.17510100
H	-2.04063400	-0.29273000	1.60443500
H	-0.62749100	-1.32008300	1.96224700
C	-1.16357300	-1.26975900	-0.78561200
H	-0.78483100	-1.20864000	-1.81890000
H	-0.93732300	-2.27839100	-0.40590900
H	-2.25559500	-1.15274900	-0.81536800
C	1.02679500	-0.49138300	0.07516100
N	1.57893200	-1.58644100	0.63195300
C	2.76985600	-1.91302300	0.12454600

C	3.21964800	-1.13812600	-1.01073800
S	1.97628400	0.04115500	-1.40231800
C	3.66336500	-2.92086500	0.60861300
C	4.87801600	-3.14608600	-0.03551200
C	5.28291700	-2.39078900	-1.14923800
C	4.42983300	-1.37142700	-1.64100100
H	2.16712900	0.36476100	1.97296300
H	3.37111800	-3.51443300	1.47686300
H	5.53938000	-3.92783400	0.34659800
H	6.23695900	-2.58878900	-1.63835900
H	4.72834600	-0.77452800	-2.50564600
C	3.17575100	1.82167800	1.26205100
O	3.09480500	0.66101200	1.99797200
O	2.16209600	2.52345700	1.16142000
O	4.30449800	2.02652100	0.75102000
K	5.37111500	-0.32795500	1.09419600
K	2.96513300	3.15118600	-1.22261800

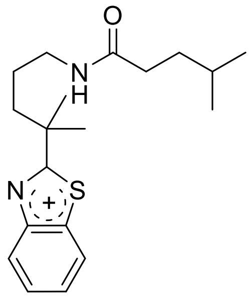

E-1

Zero-point correction= 0.454417 (Hartree/Particle)

Thermal correction to Energy= 0.478518

Thermal correction to Enthalpy= 0.479462

Thermal correction to Gibbs Free Energy= 0.399572

Sum of electronic and zero-point Energies= -1322.547679

Sum of electronic and thermal Energies= -1322.523577

Sum of electronic and thermal Enthalpies= -1322.522633

Sum of electronic and thermal Free Energies= -1322.602523

N	-2.60872200	0.68535700	0.10272100
C	-2.03092700	1.72045300	-0.73300400
C	-0.98249000	2.56698800	-0.00712400
C	0.48212000	2.12421200	-0.09302500
H	-1.28664200	2.69879400	1.04400800
H	-1.01475300	3.57280000	-0.45071000
H	1.07805600	2.94364900	0.34557600
H	0.75865500	2.06751600	-1.16035300
H	-2.93095500	0.95824100	1.02661000
C	-2.95506900	-0.54395500	-0.35282100
C	-3.70696700	-1.40401400	0.64675600
H	-3.79562400	-2.40344000	0.20278100
H	-3.10716900	-1.49577600	1.56578800
C	-5.08618600	-0.82735700	0.98804900
H	-5.62490300	-1.56032100	1.61244900
H	-4.96671500	0.07534600	1.61040600
C	-5.94906000	-0.47837000	-0.22981400
H	-5.44373600	0.33988800	-0.77604400
C	-7.31377100	0.03365700	0.22270500
H	-7.92197100	0.36128300	-0.63375900
H	-7.87057000	-0.76084000	0.74598500
H	-7.21444900	0.88466600	0.91383600
C	-6.09598100	-1.66115700	-1.18479700
H	-5.12897100	-1.94720900	-1.62289800
H	-6.50964100	-2.53740400	-0.65794800
H	-6.77843700	-1.41424100	-2.01232900
O	-2.68665100	-0.92787800	-1.48291700
H	-1.60435100	1.22824900	-1.61801800
H	-2.83445300	2.38417900	-1.09323200

C	0.90393200	0.80623600	0.59293200
C	0.27158700	0.65927100	1.97825000
H	0.41285300	1.57177300	2.57700300
H	-0.80501400	0.47273900	1.87065700
H	0.71371800	-0.18791700	2.52020300
C	0.61666300	-0.41894500	-0.27607800
H	1.20130100	-0.39594600	-1.20981000
H	0.85935000	-1.34660400	0.26380600
H	-0.44281900	-0.46101700	-0.55712000
C	2.45096400	0.89748700	0.83317900
N	3.01968000	-0.28546200	1.32440900
C	4.02961200	-0.71757900	0.65203700
C	4.41582800	0.10338500	-0.51405100
S	3.39795100	1.42323400	-0.67638200
C	4.78370200	-1.91447600	0.96243700
C	5.82812500	-2.23917900	0.16191400
C	6.18246800	-1.41634700	-0.96833400
C	5.51009600	-0.26733900	-1.31493100
H	2.62959700	1.70453100	1.57291100
H	4.48576900	-2.50831500	1.82668900
H	6.42254600	-3.13119200	0.35938500
H	7.03265100	-1.72767700	-1.57871600
H	5.80809300	0.32840200	-2.17729900

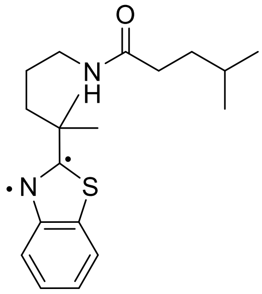

F-1

Zero-point correction= 0.439739 (Hartree/Particle)

Thermal correction to Energy= 0.464045

Thermal correction to Enthalpy= 0.464989

Thermal correction to Gibbs Free Energy= 0.383484

Sum of electronic and zero-point Energies= -1322.068217

Sum of electronic and thermal Energies= -1322.043911

Sum of electronic and thermal Enthalpies= -1322.042967

Sum of electronic and thermal Free Energies= -1322.124472

N	2.55559400	0.67092000	-0.13255400
C	2.14157900	1.92625400	0.46567600
C	0.81288500	2.44815000	-0.08716600
C	-0.48841200	2.04509100	0.61645300
H	0.75629900	2.21047200	-1.16273000
H	0.85453000	3.54658200	-0.03512700
H	-1.28814500	2.64313700	0.15523000
H	-0.43935700	2.35819100	1.67350400
H	2.48064400	0.58625300	-1.14183200
C	3.19534500	-0.31214900	0.54577600
C	3.66029400	-1.47765400	-0.30881000
H	4.02448700	-2.25050400	0.37964300
H	2.79799100	-1.89177900	-0.85457000
C	4.75126200	-1.06820700	-1.30526700
H	5.13178400	-1.97854000	-1.79918900
H	4.30916000	-0.44837800	-2.10326800
C	5.92717900	-0.30779900	-0.68245400
H	5.53789400	0.65219300	-0.29541400
C	6.97385500	0.00753400	-1.74809000
H	7.79453300	0.61307600	-1.33510400
H	7.41088500	-0.92206100	-2.14819500
H	6.53449400	0.56168500	-2.59165400
C	6.54571900	-1.06848900	0.48877000

H	5.83296600	-1.17649700	1.31902200
H	6.86755200	-2.07543900	0.17408500
H	7.43116700	-0.53909400	0.87259000
O	3.39042000	-0.26194200	1.75265200
H	2.09119400	1.77167800	1.55248500
H	2.92291000	2.68448900	0.28863200
C	-0.94291800	0.56254200	0.58302100
C	-0.75643000	-0.05399400	-0.81406200
H	-1.19849600	0.58504400	-1.59359300
H	0.31192400	-0.18686900	-1.03513400
H	-1.23972000	-1.04297100	-0.85999100
C	-0.19841800	-0.26220000	1.63822300
H	-0.47471900	0.06704300	2.65284100
H	-0.43715100	-1.33284000	1.54699300
H	0.88844700	-0.15618900	1.53807000
C	-2.42528400	0.56484900	0.91066100
N	-3.28724900	1.24568900	0.00231900
C	-4.25916300	0.48448300	-0.41807400
C	-4.39679200	-0.82190500	0.22186100
S	-3.19941700	-0.96706400	1.50104400
C	-5.21513800	0.84401600	-1.43552800
C	-6.19418100	-0.05430100	-1.79018100
C	-6.27994300	-1.32246100	-1.17067300
C	-5.37225900	-1.71067700	-0.16122000
H	-5.12567900	1.82199600	-1.90968000
H	-6.91711900	0.20828100	-2.56379800
H	-7.07428600	-2.01009600	-1.46301400
H	-5.47037500	-2.68504800	0.31941200

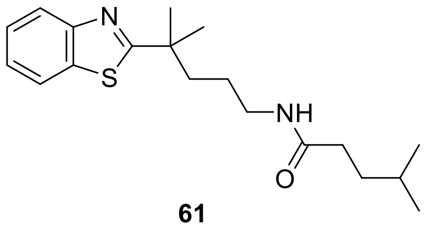

61

Zero-point correction= 0.443900 (Hartree/Particle)

Thermal correction to Energy= 0.467608

Thermal correction to Enthalpy= 0.468552

Thermal correction to Gibbs Free Energy= 0.388500

Sum of electronic and zero-point Energies= -1322.191187

Sum of electronic and thermal Energies= -1322.167480

Sum of electronic and thermal Enthalpies= -1322.166535

Sum of electronic and thermal Free Energies= -1322.246588

N	-2.70168500	-0.01758000	0.57343000
C	-2.04847800	0.92521500	1.46183200
C	-0.83602300	0.32187200	2.17678800
C	0.54706600	0.51997800	1.54855600
H	-1.02473000	-0.74880900	2.36065400
H	-0.77993000	0.78341800	3.17373900
H	1.27924400	0.11361200	2.26598600
H	0.75401600	1.60194100	1.48111000
H	-2.82777000	-0.96694600	0.91165600
C	-3.32278900	0.34141300	-0.57645000
C	-4.07255500	-0.77945500	-1.27395100
H	-4.39431700	-0.38757800	-2.24705000
H	-3.38058700	-1.61563100	-1.46014600
C	-5.27040800	-1.27590300	-0.45540200
H	-5.84086700	-1.99149900	-1.07178300
H	-4.91177000	-1.84407700	0.41918300
C	-6.21452000	-0.16930500	0.02685000
H	-5.64845900	0.47331700	0.72657400

C	-7.38699400	-0.77630200	0.79256500
H	-8.04147800	0.00466900	1.20799700
H	-7.99851000	-1.40795100	0.12751500
H	-7.03927400	-1.40555200	1.62615100
C	-6.70354900	0.70716800	-1.12430200
H	-5.87329800	1.25809700	-1.58944600
H	-7.19097200	0.09426600	-1.90103400
H	-7.43835500	1.44564800	-0.76902400
O	-3.28701200	1.48271100	-1.01499900
H	-1.76737900	1.80354500	0.86395300
H	-2.77327600	1.27407800	2.21662800
C	0.84980500	-0.11888200	0.16618000
C	0.36947100	-1.56908500	0.10320400
H	0.75538700	-2.15374000	0.95083600
H	-0.72791200	-1.59735300	0.12218300
H	0.71412200	-2.04931100	-0.82353600
C	0.24055700	0.69414800	-0.98608700
H	0.55786600	1.74794400	-0.94634900
H	0.55477600	0.28004700	-1.95651300
H	-0.85550400	0.67744100	-0.94912800
C	2.35803600	-0.10706100	0.01244900
N	3.11501600	-1.14851500	0.00705000
C	4.45403400	-0.81764800	-0.11197900
C	4.71879800	0.56602800	-0.20014300
S	3.21487100	1.43728900	-0.13364700
C	5.51807400	-1.73090700	-0.15052800
C	6.81310400	-1.24709300	-0.27387200
C	7.06301000	0.13421100	-0.36073400
C	6.02268500	1.05557200	-0.32609600
H	5.30950300	-2.79963700	-0.08271900

H	7.64987900	-1.94668800	-0.30400600
H	8.08979200	0.49072800	-0.45736100
H	6.21870300	2.12657800	-0.39435700

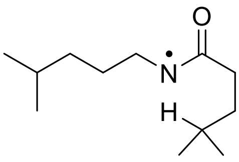

1y-iso2

Zero-point correction= 0.344276 (Hartree/Particle)

Thermal correction to Energy= 0.361687

Thermal correction to Enthalpy= 0.362632

Thermal correction to Gibbs Free Energy= 0.297375

Sum of electronic and zero-point Energies= -600.585395

Sum of electronic and thermal Energies= -600.567984

Sum of electronic and thermal Enthalpies= -600.567040

Sum of electronic and thermal Free Energies= -600.632297

N	0.03625300	-0.02765700	-0.14929800
C	-0.49143000	-0.72232500	-1.28857800
C	-1.63895300	-1.66818500	-0.94154900
C	-2.98486000	-0.96924000	-0.74811900
C	-3.05266200	0.08914800	0.35890200
C	-4.43912600	0.72977500	0.38057700
C	-2.70612700	-0.48765600	1.72961900
H	-1.36025800	-2.24743600	-0.04689400
H	-1.73719500	-2.39310200	-1.76362000
H	-3.75378700	-1.73570100	-0.54452200
H	-3.27104300	-0.49487600	-1.70297200
H	-2.32204600	0.88150300	0.11776500
H	-4.49918300	1.52781000	1.13600700
H	-5.20995000	-0.02047400	0.62270700
H	-4.69417200	1.16901900	-0.59601000

H	-2.80694300	0.27728700	2.51512700
H	-1.67479600	-0.86609200	1.76523000
H	-3.38556000	-1.31919300	1.98236500
C	0.44978200	1.28967400	-0.31699700
C	1.70736400	1.67869600	0.42242700
H	1.80349400	2.76722800	0.31859400
H	1.55717800	1.44925600	1.48885300
C	2.98170400	0.99681200	-0.09328400
H	3.10721800	1.23871900	-1.16164200
H	3.83482400	1.46112700	0.42991700
C	3.08196000	-0.52275900	0.09014300
H	2.30170000	-1.00413100	-0.52400100
C	2.86865900	-0.94677600	1.54112400
H	3.03824000	-2.02763100	1.66070400
H	3.57288700	-0.42118700	2.20767600
H	1.84620800	-0.73256900	1.88446500
C	4.43663500	-1.00687100	-0.42204700
H	4.60227800	-0.70434700	-1.46731300
H	5.25386700	-0.58278100	0.18415800
H	4.51402300	-2.10319900	-0.37087700
O	-0.22729300	2.07928800	-0.94810800
H	-0.78056900	-0.00624500	-2.07959500
H	0.35047300	-1.31543700	-1.69947800

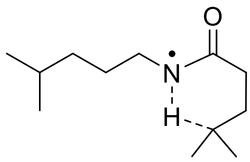

TS5

Zero-point correction= 0.339929 (Hartree/Particle)

Thermal correction to Energy= 0.356650

Thermal correction to Enthalpy= 0.357594

Thermal correction to Gibbs Free Energy= 0.295195

Sum of electronic and zero-point Energies= -600.574216

Sum of electronic and thermal Energies= -600.557494

Sum of electronic and thermal Enthalpies= -600.556550

Sum of electronic and thermal Free Energies= -600.618950

N	0.25571700	-0.00673500	-0.36394400
C	-0.36383800	-0.78542100	-1.41511000
C	-1.48061300	-1.69787300	-0.91308600
C	-2.80872600	-0.99205300	-0.63741000
C	-2.79777100	0.11961000	0.41929500
C	-4.19394000	0.72716400	0.54313900
C	-2.30890100	-0.38089000	1.77646500
H	-1.12454300	-2.23045800	-0.01592500
H	-1.65327800	-2.46976800	-1.67936600
H	-3.55256700	-1.75005600	-0.33232200
H	-3.17917600	-0.56666100	-1.58666400
H	-2.11419400	0.91312200	0.07207900
H	-4.20587900	1.56056900	1.26200000
H	-4.91733000	-0.02762900	0.89397100
H	-4.55300600	1.11023000	-0.42455200
H	-2.37109800	0.41494800	2.53510100
H	-1.26303300	-0.71668600	1.73210400
H	-2.92723100	-1.22498700	2.12653900
C	0.50535100	1.33195400	-0.52084900
C	1.69301500	1.84990700	0.27235900
H	1.83878300	2.90194800	-0.00081700
H	1.42997600	1.81144200	1.34192700
C	2.94470800	1.01060600	-0.00182300
H	3.21820400	1.11997800	-1.06340700
H	3.79413200	1.39072900	0.59057000

C	2.70596200	-0.46400200	0.30763300
H	1.54564100	-0.57233700	-0.00469900
C	2.72467700	-0.79335800	1.78774100
H	2.39549400	-1.82680000	1.97112900
H	3.75039600	-0.69222700	2.18255800
H	2.07456500	-0.11975600	2.36582900
C	3.51649900	-1.41924200	-0.54045500
H	3.36579600	-1.23322800	-1.61422100
H	4.59263400	-1.28877200	-0.32886800
H	3.25970100	-2.46703200	-0.32562200
O	-0.21183400	2.05806900	-1.19185600
H	-0.73831400	-0.09279300	-2.18936100
H	0.42044300	-1.39960200	-1.89291000

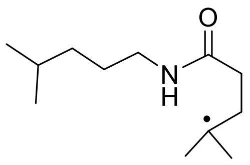

1y-iso2-HAT

Zero-point correction=	0.344715 (Hartree/Particle)
Thermal correction to Energy=	0.362364
Thermal correction to Enthalpy=	0.363308
Thermal correction to Gibbs Free Energy=	0.298850
Sum of electronic and zero-point Energies=	-600.609996
Sum of electronic and thermal Energies=	-600.592347
Sum of electronic and thermal Enthalpies=	-600.591403
Sum of electronic and thermal Free Energies=	-600.655862

N	0.13315500	1.26371700	-0.51124700
C	-1.04607300	1.66144900	-1.25556800
C	-1.83289900	0.47807400	-1.82338100
C	-2.77939500	-0.21057700	-0.83873600
C	-2.16007200	-0.80081900	0.43409800

C	-3.25578000	-1.42427700	1.29700300
C	-1.07352800	-1.82873900	0.12582600
H	-1.12138100	-0.24671600	-2.25529600
H	-2.43281500	0.84939900	-2.66869000
H	-3.30885300	-1.02149100	-1.37091100
H	-3.55587400	0.51548600	-0.54059400
H	-1.71136000	0.02535700	1.01109300
H	-2.84904500	-1.81082200	2.24395400
H	-3.73630700	-2.26520800	0.76987000
H	-4.03990700	-0.69089100	1.54030500
H	-0.68151400	-2.27676200	1.05248200
H	-0.22535900	-1.38214300	-0.41412200
H	-1.47882100	-2.64706400	-0.49356800
C	0.32983800	1.53427700	0.79921900
C	1.60120900	0.98515400	1.43029500
H	1.92990400	1.75660500	2.14067300
H	1.28872900	0.12385100	2.04511600
C	2.74998200	0.58690500	0.49494800
H	2.94139200	1.40701700	-0.21578900
H	3.66228100	0.50245600	1.11695600
C	2.54531700	-0.70400400	-0.24833300
H	0.81890300	0.69737500	-0.99914000
C	2.54395700	-1.97560000	0.53550700
H	2.02726400	-2.78685500	-0.00097200
H	3.58029200	-2.32642700	0.71735600
H	2.07214700	-1.85861800	1.52306700
C	2.89061100	-0.78145400	-1.69971800
H	2.64506500	0.14897000	-2.23665800
H	3.97600500	-0.95529500	-1.84952400
H	2.37056900	-1.61700600	-2.19516700

O	-0.47180600	2.16424700	1.47566700
H	-1.67075100	2.25417600	-0.57294400
H	-0.73721200	2.32227900	-2.08105700

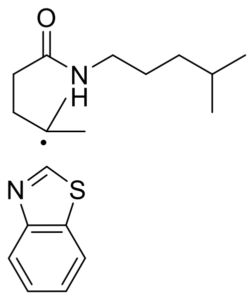

Complex 1-2

Zero-point correction= 0.449263 (Hartree/Particle)

Thermal correction to Energy= 0.475191

Thermal correction to Enthalpy= 0.476135

Thermal correction to Gibbs Free Energy= 0.389174

Sum of electronic and zero-point Energies= -1322.712122

Sum of electronic and thermal Energies= -1322.686195

Sum of electronic and thermal Enthalpies= -1322.685251

Sum of electronic and thermal Free Energies= -1322.772212

N	-2.46649100	0.52732800	-1.31129000
C	-3.35359800	-0.25504900	-2.15067800
C	-3.57205400	-1.68002200	-1.63291300
C	-4.67172600	-1.83064100	-0.58009300
C	-4.54718900	-0.97702500	0.68713200
C	-5.74697800	-1.22848600	1.59853100
C	-3.24398100	-1.23850500	1.43914000
H	-2.61053800	-2.06723300	-1.25385800
H	-3.83390800	-2.31735800	-2.49159900
H	-4.72724600	-2.89262500	-0.28005500
H	-5.63991000	-1.59813200	-1.05691600
H	-4.57133800	0.08423100	0.38649900
H	-5.70781100	-0.59473000	2.49772300

H	-5.76905100	-2.27947700	1.93143900
H	-6.69630400	-1.02153600	1.08084200
H	-3.18872600	-0.62695500	2.35376200
H	-2.36228300	-1.00240300	0.82630200
H	-3.17501200	-2.29729200	1.74270300
C	-2.82532800	1.66169900	-0.66994900
C	-1.77267200	2.31807800	0.21126300
H	-1.83205000	3.39259800	-0.01588600
H	-2.13798000	2.21199800	1.24663100
C	-0.32051800	1.82903100	0.10691400
H	-0.01267200	1.81762100	-0.95157600
H	0.30276800	2.60102800	0.59719300
H	-1.52841800	0.16782100	-1.17889000
O	-3.94287600	2.15249200	-0.75958700
H	-4.30490300	0.29181600	-2.21004300
H	-2.93112600	-0.29607800	-3.16687700
C	-0.05104300	0.49306100	0.73970600
C	-0.12280300	0.35685200	2.22541300
H	-0.76043900	1.12616300	2.68707800
H	-0.50754300	-0.63270100	2.52443500
H	0.88340700	0.44572500	2.68204600
C	0.52063300	-0.65414800	-0.02850800
H	0.61870600	-0.43672600	-1.10337900
H	1.52334500	-0.93838300	0.34725100
H	-0.10003000	-1.56439300	0.08071900
C	3.00253200	1.56317700	1.04482600
N	3.49675900	0.54034800	1.64930700
C	4.11491100	-0.31212400	0.74878400
C	4.06941300	0.11224600	-0.59752100
S	3.22354200	1.62975400	-0.68788100

C	4.74100000	-1.52561700	1.07441000
C	5.30422000	-2.28088900	0.05719100
C	5.25243800	-1.84620800	-1.28082400
C	4.63730300	-0.64954000	-1.62483100
H	2.47235800	2.37563000	1.54925500
H	4.77027100	-1.85099400	2.11519900
H	5.79405800	-3.22649200	0.29443000
H	5.70243500	-2.45964800	-2.06298500
H	4.59705200	-0.31654300	-2.66273000

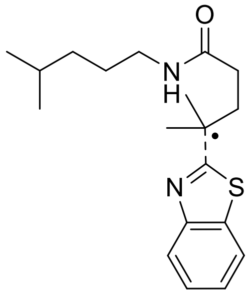

TS7

Zero-point correction=	0.450163 (Hartree/Particle)
Thermal correction to Energy=	0.474614
Thermal correction to Enthalpy=	0.475558
Thermal correction to Gibbs Free Energy=	0.394323
Sum of electronic and zero-point Energies=	-1322.699259
Sum of electronic and thermal Energies=	-1322.674809
Sum of electronic and thermal Enthalpies=	-1322.673865
Sum of electronic and thermal Free Energies=	-1322.755099

N	-2.23692700	0.29635100	-1.33031900
C	-3.07417200	-0.61129300	-2.09199900
C	-3.34207300	-1.93737500	-1.36957400
C	-4.55678700	-1.93894700	-0.43962500
C	-4.58303900	-0.88566500	0.67329300
C	-5.88569000	-0.99803000	1.46314000
C	-3.38163400	-0.99522800	1.60995500

H	-2.42899400	-2.22951300	-0.82287600
H	-3.49767900	-2.71592900	-2.13210300
H	-4.64592900	-2.93853600	0.02254700
H	-5.46220700	-1.80736500	-1.05755200
H	-4.56316900	0.10949900	0.19882300
H	-5.95175700	-0.21777100	2.23670700
H	-5.95422000	-1.97617400	1.96766000
H	-6.76392300	-0.89762600	0.80698200
H	-3.45094700	-0.25654700	2.42413700
H	-2.43436900	-0.82143900	1.07952900
H	-3.33315500	-1.99658000	2.07089200
C	-2.63558300	1.49678300	-0.85810900
C	-1.63246300	2.27282800	-0.01774000
H	-1.79690700	3.32428700	-0.28972200
H	-1.97316200	2.18303300	1.02720400
C	-0.13112800	1.94589700	-0.13507400
H	0.12137200	1.72025400	-1.18346500
H	0.39330900	2.88417200	0.11477600
H	-1.28992500	-0.00930900	-1.14288000
O	-3.75330300	1.95488300	-1.05276000
H	-4.01374600	-0.08219800	-2.30409100
H	-2.58141000	-0.81204100	-3.05562900
C	0.41229700	0.86606000	0.77584300
C	0.10017300	1.00656000	2.23539900
H	0.13529600	2.05729800	2.56225600
H	-0.91407700	0.62321900	2.44893900
H	0.80739300	0.42080900	2.84081500
C	0.52733500	-0.54247900	0.27783400
H	0.81997700	-0.58655000	-0.78311700
H	1.26006800	-1.10782000	0.87457100

H	-0.43111500	-1.08266300	0.39281700
C	2.51897200	1.44939500	0.83909300
N	3.12725700	0.53057900	1.56114900
C	3.85564900	-0.32176600	0.77482300
C	3.91649100	0.01685500	-0.60244100
S	3.01303100	1.47953600	-0.87766600
C	4.52867900	-1.47367500	1.22586400
C	5.23186700	-2.24759200	0.31468700
C	5.27950700	-1.89898500	-1.04745700
C	4.62209100	-0.76632600	-1.51864200
H	2.17716500	2.39213800	1.27579600
H	4.48187800	-1.73591100	2.28397300
H	5.75484100	-3.14201100	0.65737200
H	5.83923100	-2.52335200	-1.74555700
H	4.65882600	-0.49761900	-2.57550000

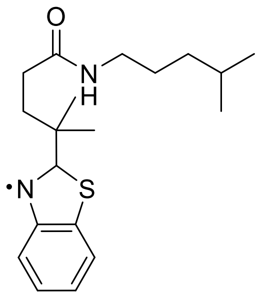

C-2

Zero-point correction= 0.453020 (Hartree/Particle)

Thermal correction to Energy= 0.477390

Thermal correction to Enthalpy= 0.478334

Thermal correction to Gibbs Free Energy= 0.396291

Sum of electronic and zero-point Energies= -1322.735528

Sum of electronic and thermal Energies= -1322.711159

Sum of electronic and thermal Enthalpies= -1322.710215

Sum of electronic and thermal Free Energies= -1322.792257

N	-2.73034000	-0.79029400	-0.74234400

C	-3.77420300	-1.68095400	-1.21051000
C	-4.67050500	-2.21073100	-0.08953000
C	-5.76975100	-1.25575700	0.37948300
C	-5.33231300	0.11059600	0.92214800
C	-6.56328000	0.92573500	1.31456900
C	-4.37807300	-0.01459800	2.10785800
H	-4.03435600	-2.52162400	0.75674600
H	-5.15342800	-3.13044300	-0.45467900
H	-6.36137700	-1.76312600	1.16276800
H	-6.46281400	-1.08581600	-0.46284200
H	-4.82010000	0.65393700	0.11047900
H	-6.28291300	1.93087900	1.66475800
H	-7.11660100	0.42860000	2.12850300
H	-7.25275600	1.04447900	0.46464500
H	-4.09846100	0.97731000	2.49576500
H	-3.45107000	-0.54147300	1.84005300
H	-4.85617100	-0.57173900	2.93135400
C	-2.60047400	0.50297900	-1.12208500
C	-1.45172400	1.24932000	-0.46283800
H	-1.52620200	2.28775800	-0.80684200
H	-1.61012600	1.23590700	0.62806300
C	-0.08721100	0.65185300	-0.81538200
H	-0.08096100	-0.41756400	-0.54758300
H	0.04448800	0.70693500	-1.91030000
H	-2.08427800	-1.15298600	-0.04852700
O	-3.34658100	1.04118600	-1.92699700
H	-4.36435700	-1.11985800	-1.94838600
H	-3.30921300	-2.53024200	-1.73588800
C	1.11521800	1.33284800	-0.13415100
C	1.08667300	2.84661100	-0.37111100

H	0.98412100	3.07699000	-1.44340200
H	0.24283400	3.30659600	0.16240900
H	2.01343600	3.30901400	-0.00579000
C	1.14391200	1.03729800	1.36531300
H	1.20949500	-0.04479700	1.55711100
H	2.00701600	1.52744500	1.84004600
H	0.23473800	1.41627400	1.85420500
C	2.40939600	0.79647900	-0.80593300
N	3.59956500	1.31843000	-0.23070800
C	4.47280100	0.38837000	0.12154300
C	4.09759500	-0.98611400	-0.10199900
S	2.51609400	-1.08154300	-0.80036000
C	5.74738100	0.65966500	0.70489700
C	6.58263500	-0.38668900	1.03122400
C	6.19357700	-1.72536400	0.79847200
C	4.95575700	-2.03073300	0.23425900
H	2.36579400	1.08037300	-1.87458700
H	6.03101400	1.69893000	0.87631300
H	7.55864300	-0.18417100	1.47494600
H	6.87332300	-2.53600200	1.06514400
H	4.66637500	-3.06821900	0.06125100

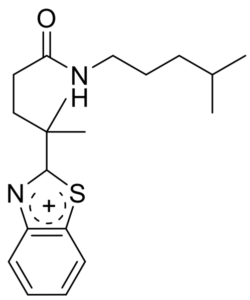

E-2

Zero-point correction= 0.454077 (Hartree/Particle)

Thermal correction to Energy= 0.477639

Thermal correction to Enthalpy= 0.478583

Thermal correction to Gibbs Free Energy= 0.399964

Sum of electronic and zero-point Energies= -1322.548521

Sum of electronic and thermal Energies= -1322.524959

Sum of electronic and thermal Enthalpies= -1322.524015

Sum of electronic and thermal Free Energies= -1322.602634

N	-2.73269400	-0.83412700	-0.69559000
C	-3.77700900	-1.73390600	-1.14645200
C	-4.70729400	-2.19932100	-0.02479500
C	-5.79784900	-1.20617800	0.38049600
C	-5.34745700	0.16946600	0.88822100
C	-6.57229400	1.01753600	1.22620800
C	-4.42185400	0.06677500	2.09851600
H	-4.09615900	-2.48789800	0.84743800
H	-5.19981500	-3.12381500	-0.36413100
H	-6.42037100	-1.67374400	1.16455100
H	-6.46500200	-1.05247100	-0.48546800
H	-4.80870900	0.67767200	0.07119000
H	-6.28286800	2.02899800	1.54983800
H	-7.15148100	0.55638800	2.04327500
H	-7.24078500	1.11924600	0.35751600
H	-4.13188000	1.06582400	2.45956000
H	-3.49958100	-0.48660800	1.87049600
H	-4.92865500	-0.45355600	2.92891700
C	-2.58420300	0.44010400	-1.12723100
C	-1.44193900	1.20619400	-0.47685600
H	-1.50894500	2.23267400	-0.85539100
H	-1.60959700	1.22512400	0.61215000
C	-0.07783700	0.59327800	-0.80186400
H	-0.07722100	-0.46238300	-0.48230800
H	0.05610900	0.60193100	-1.89708700

H	-2.10871400	-1.17137600	0.03066000
O	-3.30678600	0.95135500	-1.96943800
H	-4.34190100	-1.20160700	-1.92424800
H	-3.31002800	-2.61250700	-1.61902300
C	1.11441200	1.31441700	-0.14349100
C	1.09847800	2.81227800	-0.46084100
H	0.99933500	2.98710200	-1.54285900
H	0.25109400	3.29051400	0.04878600
H	2.01998300	3.29533400	-0.11024700
C	1.14792200	1.08817500	1.36786500
H	1.18802000	0.01715100	1.61958800
H	2.01523700	1.59015900	1.82169100
H	0.24408500	1.50828800	1.83058200
C	2.41556200	0.73969600	-0.78898800
N	3.60001800	1.28001400	-0.26740900
C	4.47301000	0.40894600	0.10181400
C	4.07835900	-1.00353400	-0.07512700
S	2.53325700	-1.11401100	-0.71474900
C	5.77442800	0.72325200	0.65451800
C	6.59368000	-0.30371200	0.98778600
C	6.18252000	-1.67271600	0.79802000
C	4.96174300	-2.03785700	0.27765400
H	2.38305600	0.97062800	-1.87435200
H	6.04706800	1.77135700	0.77851100
H	7.58220000	-0.11297500	1.40544700
H	6.88284800	-2.45976300	1.08482500
H	4.68932100	-3.08491800	0.14984800

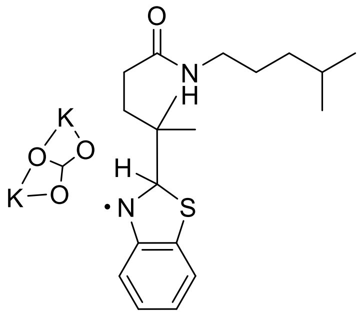

Complex 2-2

Zero-point correction= 0.471757 (Hartree/Particle)

Thermal correction to Energy= 0.504390

Thermal correction to Enthalpy= 0.505334

Thermal correction to Gibbs Free Energy= 0.402776

Sum of electronic and zero-point Energies= -2786.100555

Sum of electronic and thermal Energies= -2786.067922

Sum of electronic and thermal Enthalpies= -2786.066978

Sum of electronic and thermal Free Energies= -2786.169536

N	-3.82374700	-2.11228700	-0.80173900
C	-5.17835400	-1.69192500	-1.11212400
C	-6.00270600	-1.33929300	0.13059300
C	-5.85094800	0.10192600	0.61461800
C	-4.43444100	0.56377300	0.96508800
C	-4.42683600	2.06137500	1.27138600
C	-3.80243000	-0.21250100	2.11754200
H	-5.75386200	-2.05342100	0.93357900
H	-7.06339000	-1.51122800	-0.10772400
H	-6.49799700	0.25437200	1.49679500
H	-6.24595200	0.76883100	-0.17193900
H	-3.79429800	0.38258700	0.08692300
H	-3.40214600	2.39988000	1.48827600
H	-5.05858900	2.27647900	2.14929900
H	-4.83747300	2.65032600	0.43292900
H	-2.78927400	0.18031400	2.29696400

H	-3.72235600	-1.28748200	1.89292000
H	-4.39505300	-0.10114200	3.04157900
C	-2.70157900	-1.50915900	-1.21503800
C	-1.41538500	-2.19854000	-0.81333200
H	-1.62209700	-2.90663200	0.00152600
H	-1.09491400	-2.79894800	-1.68052500
C	-0.32599500	-1.19872200	-0.42552300
H	0.05246200	-0.73161900	-1.35047300
H	-0.75395600	-0.38189400	0.18275600
H	-3.72114900	-2.97030700	-0.26834500
O	-2.70041700	-0.47770200	-1.86976300
H	-5.10806700	-0.83401900	-1.79551500
H	-5.67220700	-2.51021400	-1.65777600
C	0.84373000	-1.82840700	0.35647300
C	0.39410900	-2.16076700	1.78552100
H	0.12575900	-1.24284300	2.33154800
H	-0.48431100	-2.82243600	1.77592200
H	1.19672000	-2.66635200	2.33932300
C	1.37891400	-3.08596000	-0.33016600
H	1.58527000	-2.90700800	-1.39653300
H	2.31168700	-3.41805200	0.15161100
H	0.65352400	-3.90942100	-0.25497300
C	1.95947800	-0.76594700	0.49079800
N	3.05877600	-1.17364200	1.28513700
C	4.24210300	-0.90732700	0.74775500
C	4.24923700	-0.34584500	-0.57876800
S	2.62568000	-0.18698700	-1.18138900
C	5.49242000	-1.14207600	1.39179400
C	6.66534400	-0.81411600	0.74333100
C	6.64579200	-0.25432300	-0.55216600

C	5.44216400	-0.01977900	-1.21871200
H	1.51480100	0.15544200	0.94232500
H	5.49008200	-1.57161200	2.39447700
H	7.62326700	-0.98567400	1.23678600
H	7.58652300	-0.00286000	-1.04419300
H	5.43898700	0.40479000	-2.22391800
C	-0.05014800	2.15540500	0.68648200
O	0.98984200	2.01124200	1.42943500
O	-1.19457000	1.72533400	1.05951800
O	0.06986300	2.73666000	-0.47621700
K	2.56850600	2.92431100	-0.31181400
K	-2.26759200	2.00844800	-1.22409600

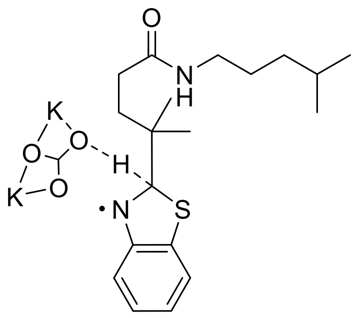

TS9

Zero-point correction=	0.466683 (Hartree/Particle)
Thermal correction to Energy=	0.498739
Thermal correction to Enthalpy=	0.499683
Thermal correction to Gibbs Free Energy=	0.401236
Sum of electronic and zero-point Energies=	-2786.100376
Sum of electronic and thermal Energies=	-2786.068320
Sum of electronic and thermal Enthalpies=	-2786.067376
Sum of electronic and thermal Free Energies=	-2786.165823

N	-4.02354100	-1.79848800	-1.13540700
C	-5.21084700	-1.13438100	-1.64116700
C	-6.12611300	-0.58644300	-0.54578100
C	-5.62775600	0.65956100	0.19069900

C	-4.38876400	0.52897100	1.09025700
C	-4.13076400	1.85092100	1.81521000
C	-4.50687100	-0.61431700	2.09495600
H	-6.35406400	-1.39355700	0.17038100
H	-7.08369600	-0.33262500	-1.02700500
H	-6.45317900	1.03129300	0.82372300
H	-5.44237100	1.45583100	-0.55358800
H	-3.50759900	0.31954800	0.46315800
H	-3.14334400	1.84814200	2.29877000
H	-4.90208500	2.02797700	2.58251100
H	-4.17569400	2.71266100	1.12654000
H	-3.63750300	-0.63094300	2.77050500
H	-4.56398100	-1.59386200	1.59816200
H	-5.41138000	-0.49470800	2.71467300
C	-2.78530100	-1.27868600	-1.18288900
C	-1.72987500	-2.06987600	-0.42900200
H	-2.08599200	-2.12633600	0.61276700
H	-1.72402100	-3.10309800	-0.81147600
C	-0.36023400	-1.41365600	-0.49800500
H	-0.00218700	-1.45480600	-1.54021400
H	-0.48729600	-0.35560300	-0.22812500
H	-4.14986200	-2.67060900	-0.63021800
O	-2.53595000	-0.21124000	-1.74622400
H	-4.87495000	-0.32316600	-2.30119500
H	-5.77163700	-1.85292400	-2.25713600
C	0.70978100	-2.00038800	0.44141900
C	0.20185200	-2.00249200	1.89000300
H	-0.21065500	-1.01022800	2.13506000
H	-0.57520000	-2.76629600	2.04246400
H	1.02974200	-2.22204300	2.57936900

C	1.12261300	-3.40907200	0.00900100
H	1.52976800	-3.39858800	-1.01467300
H	1.89939200	-3.80428400	0.68103000
H	0.26636600	-4.10109900	0.03164000
C	1.92437400	-1.05812100	0.41692800
N	3.03422800	-1.40285900	1.15409600
C	4.17803400	-0.92687500	0.63179900
C	4.10038600	-0.38044800	-0.69156600
S	2.47083600	-0.52480100	-1.30296400
C	5.43736400	-0.91884100	1.28202500
C	6.54280800	-0.37761900	0.64200700
C	6.44250700	0.16438500	-0.65066200
C	5.21823600	0.16031000	-1.32619400
H	1.57547700	0.08194700	0.90747400
H	5.51098800	-1.33328200	2.28888800
H	7.50678400	-0.36606100	1.15421100
H	7.32426900	0.58607700	-1.13521100
H	5.14394700	0.56531700	-2.33840800
C	0.28392700	1.85414100	0.84304300
O	1.43408300	1.37397300	1.28367200
O	-0.80331900	1.38486200	1.27359900
O	0.33116300	2.77658500	-0.05006100
K	2.85221300	2.59866500	-0.46474600
K	-2.06369700	2.17163400	-0.84093000

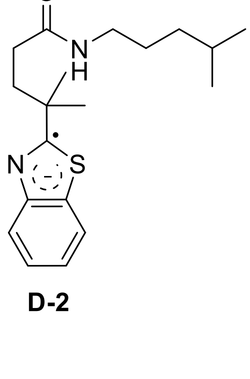

D-2

Zero-point correction= 0.439897 (Hartree/Particle)

Thermal correction to Energy= 0.464150

Thermal correction to Enthalpy= 0.465094

Thermal correction to Gibbs Free Energy= 0.384234

Sum of electronic and zero-point Energies= -1322.254105

Sum of electronic and thermal Energies= -1322.229852

Sum of electronic and thermal Enthalpies= -1322.228908

Sum of electronic and thermal Free Energies= -1322.309768

N	-3.97536400	-1.00249800	0.20509100
C	-5.12152300	-0.13746600	0.00592900
C	-5.06870800	1.16295500	0.80902700
C	-4.14861700	2.25078200	0.25168700
C	-2.64337500	1.95577900	0.21163700
C	-1.89532900	3.15291100	-0.37159600
C	-2.09141500	1.60439100	1.59186600
H	-4.80924400	0.92950000	1.85568200
H	-6.09231700	1.56851900	0.83813300
H	-4.29561800	3.16769400	0.85073700
H	-4.48382900	2.49899900	-0.77053400
H	-2.47549300	1.10106300	-0.46504300
H	-0.82099300	2.93670400	-0.47449900
H	-2.00136900	4.03585400	0.28033500
H	-2.28272300	3.42183300	-1.36640700
H	-0.99535100	1.49457900	1.56580700
H	-2.51069100	0.66169800	1.97497800
H	-2.33013300	2.40076000	2.31709300
C	-3.12718400	-1.37097500	-0.78469300
C	-1.93372900	-2.19285600	-0.33713300
H	-2.12764600	-2.65749500	0.63991200
H	-1.77826100	-2.98868900	-1.07987000

C	-0.72048600	-1.26122200	-0.27179000
H	-0.58738200	-0.81397200	-1.27166300
H	-0.95865200	-0.43469300	0.41614800
H	-3.80118900	-1.35159300	1.14152200
O	-3.25769100	-1.01185400	-1.94635500
H	-5.17093100	0.07664100	-1.07071900
H	-6.03572800	-0.69033500	0.27529800
C	0.60902700	-1.88476200	0.18603200
C	0.43198400	-2.59830700	1.53187200
H	-0.04208000	-1.92940700	2.26852500
H	-0.18813700	-3.50168900	1.42958100
H	1.41186600	-2.89735500	1.92868500
C	1.12054800	-2.88980300	-0.86003900
H	1.27176900	-2.39024600	-1.83012100
H	2.08833000	-3.30444900	-0.53772800
H	0.42171300	-3.72906600	-1.00790300
C	1.62435700	-0.76834900	0.37470100
N	2.83628700	-1.01037600	0.93019800
C	3.78931000	-0.15984900	0.53313700
C	3.42067000	0.78457100	-0.49258200
S	1.74338400	0.50788900	-0.93675400
C	5.12553900	-0.08560700	1.02704500
C	6.00984700	0.86011100	0.51449200
C	5.62901600	1.76362200	-0.48657800
C	4.31188300	1.71816600	-0.99409700
H	5.43712200	-0.78145700	1.80913600
H	7.02903200	0.89841100	0.90888900
H	6.33838900	2.49761100	-0.87151600
H	3.99958600	2.41477300	-1.77607400

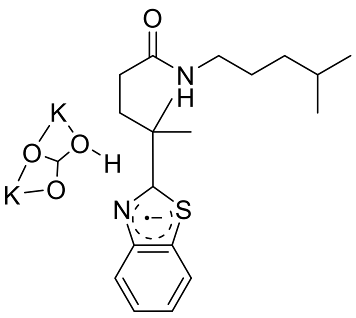

Complex 3-2

Zero-point correction= 0.471086 (Hartree/Particle)

Thermal correction to Energy= 0.503931

Thermal correction to Enthalpy= 0.504875

Thermal correction to Gibbs Free Energy= 0.405471

Sum of electronic and zero-point Energies= -2786.116889

Sum of electronic and thermal Energies= -2786.084044

Sum of electronic and thermal Enthalpies= -2786.083100

Sum of electronic and thermal Free Energies= -2786.182504

N	-4.51683300	-1.45343600	-0.32591900
C	-5.59563900	-0.64308900	-0.86492000
C	-6.11889200	0.40887200	0.11812100
C	-5.33175400	1.72027800	0.15213500
C	-3.84391600	1.62880800	0.50801000
C	-3.19141100	3.00702300	0.40349000
C	-3.59801400	1.02381700	1.88822300
H	-6.17609700	-0.04229900	1.12300700
H	-7.15645300	0.64260500	-0.16564700
H	-5.81738400	2.40473600	0.87042500
H	-5.42209500	2.20193200	-0.83726100
H	-3.36926400	0.97181700	-0.23953400
H	-2.11785100	2.94872900	0.64202900
H	-3.65491200	3.70905100	1.11647500
H	-3.31256100	3.43797000	-0.60396700
H	-2.51851400	1.00431800	2.10183100

H	-3.98251800	-0.00475700	1.96075400
H	-4.09507200	1.62525100	2.66855700
C	-3.25981300	-1.47508000	-0.79298100
C	-2.29159400	-2.39270500	-0.06058200
H	-2.66111800	-2.59017400	0.95648800
H	-2.26966500	-3.35510400	-0.59763700
C	-0.92375600	-1.72437300	-0.06550300
H	-0.63069500	-1.64654800	-1.12516300
H	-1.02934300	-0.70625100	0.33865000
H	-4.73219000	-2.04727200	0.46919600
O	-2.88370100	-0.77288300	-1.73676000
H	-5.21859800	-0.16826800	-1.78105500
H	-6.41703500	-1.31631200	-1.15248200
C	0.23874700	-2.36289900	0.71241100
C	-0.03128400	-2.28075600	2.21757500
H	-0.18794700	-1.23241700	2.51687100
H	-0.92654900	-2.86178800	2.48660400
H	0.82011600	-2.67835900	2.78753100
C	0.43908300	-3.83038900	0.29719300
H	0.60799100	-3.91046400	-0.78850900
H	1.31670100	-4.25335200	0.81024400
H	-0.43648800	-4.44750300	0.55640500
C	1.46926900	-1.54028600	0.39898500
N	2.52608200	-1.36227900	1.20309300
C	3.59288300	-0.84829100	0.58157400
C	3.46950000	-0.65342400	-0.84620300
S	1.85770000	-1.15655700	-1.33691400
C	4.81951500	-0.41091200	1.18480000
C	5.83769300	0.10965100	0.39096500
C	5.70870200	0.24895100	-1.00299300

C	4.49363700	-0.14624500	-1.62605700
H	1.65803200	0.64703600	2.03445100
H	4.94201500	-0.50309900	2.26565800
H	6.76691000	0.42741400	0.87163700
H	6.52816100	0.64759900	-1.60144400
H	4.36449700	-0.03261100	-2.70498400
C	0.75981800	1.74080000	0.73952900
O	1.82699000	1.48777200	1.57630900
O	-0.33963600	1.25997100	1.04549900
O	1.04402300	2.44185100	-0.25846900
K	3.67333400	2.31653000	-0.07227600
K	-0.92059900	1.07983100	-1.54582800

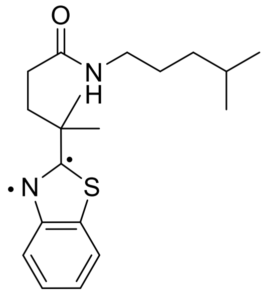

F-2

Zero-point correction= 0.439740 (Hartree/Particle)

Thermal correction to Energy= 0.463934

Thermal correction to Enthalpy= 0.464879

Thermal correction to Gibbs Free Energy= 0.385563

Sum of electronic and zero-point Energies= -1322.077031

Sum of electronic and thermal Energies= -1322.052836

Sum of electronic and thermal Enthalpies= -1322.051892

Sum of electronic and thermal Free Energies= -1322.131208

N	-3.54346600	-0.81350300	0.18013700
C	-4.51097900	0.19043200	-0.21899900
C	-4.27899600	1.56465700	0.41175800
C	-3.12917400	2.38330400	-0.17959100

C	-1.71309500	1.81226500	-0.03218500
C	-0.68889700	2.75958700	-0.65326300
C	-1.35960200	1.53069000	1.42691900
H	-4.14977400	1.44261000	1.50064400
H	-5.20862200	2.14125300	0.28319600
H	-3.14134200	3.38447700	0.28805200
H	-3.33138900	2.54572000	-1.25266200
H	-1.66404600	0.87016100	-0.60122200
H	0.31273900	2.30114300	-0.66076500
H	-0.62430400	3.70208000	-0.08344100
H	-0.95181400	3.01047100	-1.69276900
H	-0.30361100	1.23500400	1.53163600
H	-1.97424400	0.72358800	1.85268700
H	-1.51295400	2.43402200	2.04159500
C	-2.67501800	-1.40312100	-0.67369500
C	-1.65748600	-2.32758900	-0.03013400
H	-1.93701900	-2.55328000	1.00848500
H	-1.65356300	-3.26598100	-0.60413200
C	-0.28614800	-1.65031700	-0.10073600
H	-0.08092600	-1.38945100	-1.15156300
H	-0.33751900	-0.70521900	0.46357700
H	-3.49428300	-1.06343900	1.16199800
O	-2.65181300	-1.17059400	-1.87399500
H	-4.46319900	0.25697300	-1.31472700
H	-5.51938100	-0.16251000	0.04853700
C	0.90566000	-2.47060900	0.44443600
C	0.64719300	-2.92365500	1.88847200
H	0.35161200	-2.07699400	2.52443700
H	-0.15009300	-3.68030800	1.91422100
H	1.55499600	-3.37395100	2.31832300

C	1.17964600	-3.69248500	-0.43656700
H	1.33065700	-3.40520600	-1.48886000
H	2.07706200	-4.22684000	-0.08895800
H	0.33346200	-4.39376900	-0.39891800
C	2.11742100	-1.56538600	0.44910900
N	2.11683900	-0.46482100	1.35480200
C	2.43356700	0.65456100	0.77039800
C	2.83268000	0.57947500	-0.63595000
S	2.87540600	-1.10726500	-1.13423400
C	2.43804400	1.95151000	1.40763600
C	2.78465300	3.06262800	0.67645100
C	3.13111600	2.95716100	-0.69056500
C	3.15090000	1.70657200	-1.35175400
H	2.15963900	2.01365800	2.46031000
H	2.78861400	4.04514000	1.15055600
H	3.40972400	3.85395500	-1.24498000
H	3.44506700	1.64787500	-2.40055900

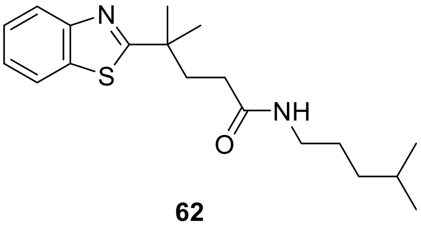

62

Zero-point correction= 0.443591 (Hartree/Particle)

Thermal correction to Energy= 0.467508

Thermal correction to Enthalpy= 0.468452

Thermal correction to Gibbs Free Energy= 0.387500

Sum of electronic and zero-point Energies= -1322.193546

Sum of electronic and thermal Energies= -1322.169629

Sum of electronic and thermal Enthalpies= -1322.168685

Sum of electronic and thermal Free Energies= -1322.249637

N	-2.54193300	0.94527600	0.59510900
C	-3.44950700	1.96746200	1.07943600
C	-4.52312700	2.36029600	0.06244700
C	-5.72439400	1.41674200	-0.02329800
C	-5.44498300	-0.04752200	-0.38440900
C	-6.75308500	-0.83709600	-0.38796000
C	-4.73588500	-0.18744500	-1.72975100
H	-4.04814100	2.48150300	-0.92613000
H	-4.89557600	3.35868300	0.33988500
H	-6.43623000	1.82434400	-0.76374700
H	-6.25002700	1.43810600	0.94725200
H	-4.80130300	-0.47593500	0.40190200
H	-6.57656600	-1.90373400	-0.59446200
H	-7.43777600	-0.45418900	-1.16285100
H	-7.26981500	-0.76123600	0.58116400
H	-4.55983600	-1.24649000	-1.97468500
H	-3.76243900	0.32360900	-1.73943000
H	-5.35076000	0.24432300	-2.53756500
C	-2.38819000	-0.27285200	1.16583000
C	-1.41273000	-1.19521300	0.45222400
H	-1.43765000	-2.14644300	0.99747800
H	-1.78955800	-1.37069400	-0.56820900
C	0.00685200	-0.62824100	0.40480000
H	0.01001600	0.32107000	-0.15501300
H	0.34009500	-0.39411100	1.42928200
H	-2.02597900	1.14604100	-0.25546500
O	-2.98974500	-0.62030100	2.17140300
H	-3.90813700	1.57690400	1.99852500
H	-2.86575000	2.86018000	1.35453600
C	1.05398500	-1.57128200	-0.24010000

C	1.20418200	-2.86235100	0.56850100
H	1.42428700	-2.64620000	1.62297100
H	0.27969500	-3.45336400	0.51461600
H	2.02426400	-3.47488900	0.16659400
C	0.67429000	-1.90193300	-1.68860100
H	0.46722300	-0.99195500	-2.27459400
H	1.48197800	-2.45886500	-2.18627200
H	-0.22970400	-2.52646100	-1.71343100
C	2.37034400	-0.82245500	-0.20725700
N	3.27865100	-0.96811700	0.69361100
C	4.34181400	-0.10284700	0.50401300
C	4.22001300	0.75103700	-0.61356800
S	2.71775800	0.41693500	-1.42344500
C	5.48447900	-0.02533900	1.31453900
C	6.47248000	0.89502800	0.99404800
C	6.33912400	1.73891300	-0.12347300
C	5.21572800	1.67752700	-0.93931600
H	5.57511700	-0.68618000	2.17785400
H	7.36630800	0.96634100	1.61569700
H	7.12991700	2.45395300	-0.35577900
H	5.11480600	2.33242600	-1.80589100
